# Global, regional, and national burden of chronic hepatitis C including cirrhosis from 1990 to 2021, alongside projections for the next 15 years

**DOI:** 10.1097/MD.0000000000051118

**Published:** 2026-10-16

**Authors:** Shicheng Gu, Yaojian Shao, Chenyu Jiang, Zhenjun Yu, Mingqi Hong

**Affiliations:** a Department of Gastroenterology, Taizhou Central Hospital (Taizhou University Hospital), Taizhou, Zhejiang, China; b Department of Geriatric, Taizhou Central Hospital (Taizhou University Hospital), Taizhou, Zhejiang, China.

**Keywords:** chronic hepatitis C, cirrhosis, Global Burden of Disease, health inequality, trends

## Abstract

Chronic hepatitis C (CHC), including cirrhosis, remains a major global health challenge. While direct-acting antivirals have improved prognoses, barriers to diagnosis and equitable access hinder World Health Organization’s 2030 eradication target. This study systematically assessed the global burden and trends to inform prevention strategies. Based on the Global Burden of Disease Study 2021 database, this analysis quantified the absolute numbers and age-standardized rates (ASRs) of 4 disease burden indicators, including incidence, prevalence, deaths, and disability-adjusted life years (DALYs), for CHC, including cirrhosis, across global, regional, and national levels from 1990 to 2021. Temporal trends were quantified using the estimated annual percent change and Joinpoint regression. Decomposition, health inequality, and frontier analyses were used to characterize drivers and socioeconomic gradients, with Bayesian age-period-cohort projections to 2036. Analyses were stratified by socio-demographic index (SDI), sex, and age. In 2021, the global numbers of incidence, prevalence, deaths, and DALYs for CHC, including cirrhosis, were substantially higher than in 1990, though the ASRs declined. Significant socioeconomic inequalities persisted, with the highest burden in low SDI regions. South Asia, Southeast Asia, East Asia, Western Sub-Saharan Africa, and North Africa and the Middle East showed the highest absolute numbers for disease burden indicators; Sub-Saharan Africa, Central Asia, Southeast Asia, and North Africa and the Middle East had the highest ASRs. Males exhibited higher deaths and DALYs than females. Decomposition analysis identified population growth and aging as drivers of increased disease burden, partially countered by epidemiological improvements. Frontier analysis revealed substantial unrealized potential in low SDI settings. Projections indicated a continued decline in ASRs, but the number of prevalence may reach approximately 150 million by 2036 under the continuation of current trends. Despite declining ASRs of CHC, including cirrhosis, from 1990 to 2021, the absolute burden increased due to demographic shifts. Pronounced socioeconomic inequalities persist, with the highest burden concentrated in low SDI regions.

## 1. Introduction

Chronic hepatitis C (CHC), a chronic liver disease caused by the hepatitis C virus (HCV), has garnered considerable attention as a major public health challenge due to the substantial burden it imposes. Among patients with CHC virus infection, the risk of liver cirrhosis is 15 to 30% in 20 years.^[1]^ Otherwise, it will pose a severe risk of liver failure and death.^[2,3]^ No protective vaccine is currently present, even though the dissemination of DAAs has greatly improved the course of the disease as of 2014,^[4]^ the effectiveness of the use of the DAAs has not led to a rapid decrease in the burden of disease.^[5]^ Underdiagnosis and inequitable treatment availability remain issues that render HCV a persistent global public health challenge.^[6]^

In 2015, the HCV prevalence was estimated to be 1.0% globally. The number of HCV-infected people is 71 million.^[7]^ It is unfortunate that in the period between 2015 and 2022, only 36% of hepatitis C patients were diagnosed, and 20% were cured.^[8]^ In 2016, the World Health Organization (WHO) embraced the global hepatitis strategy to eliminate hepatitis C as a health challenge to the population by the year 2030. The plan will seek to eliminate or at a minimum lower the rates of hepatitis C by 80% and death rates by 65%.^[9]^ However, by the end of 2022, the global target of hepatitis C cure elimination by 2030 had been met by just 12.5 million people, well short of the global goal to eliminate hepatitis C.^[8]^

While previous Global Burden of Disease (GBD) analyses have described hepatitis C epidemiology, most relied on earlier GBD iterations and focused primarily on descriptive trends. The primary contribution of the present study is an integrated analytical framework that combines decomposition analysis, health inequality analysis, and frontier analysis to systematically examine the drivers of disease, reveal health disparities, and quantify unrealized health potential across socioeconomic contexts, as well as Bayesian age-period-cohort (BAPC) projections through 2036 to explore plausible future trajectories under continuation-of-trends assumptions. These analyses are built upon updated GBD 2021 estimates. The core objective is to deliver a comprehensive, evidence-based framework to inform strategic prioritization in global HCV elimination.

## 2. Methods

### 2.1. Data sources

Based on the GBD 2021 data, this systematic analysis estimated the burden of CHC including cirrhosis worldwide between 1990 and 2021. The definition of CHC including cirrhosis was based on International Classification of Diseases, 10th Revision codes (B18.2, K74.6). CHC including cirrhosis in the GBD report is characterized as a disease that encompasses cirrhosis and other chronic liver diseases caused by hepatitis C, such as the progressive destruction of functional hepatocytes and the substitution of liver fibrosis by the hepatitis virus. All data can be accessed via the Global Health Data Exchange platform (https://ghdx.healthdata.org), which is an open-access global health data repository. Data were obtained from a range of sources, including vital registration systems, censuses, disease registries, healthcare utilization records, verbal autopsies, household surveys, and other sources. The GBD data collection procedures, methods, and statistical modeling methods were described in detail in previous reports.^[10,11]^ This study gathered and examined data on CHC, including cirrhosis, in 204 countries and territories grouped into 5 socio-demographic index (SDI) regions and 21 GBD geographic regions between 1990 and 2021. The analysis included absolute numbers and rates for 4 disease indicators: incidence, prevalence, deaths, and disability-adjusted life years (DALYs). All the rates mentioned are per 100,000 population. DALYs provide a comprehensive measure of disease burden by summing years of life lost and years lived with disabilities. Years of life lost is calculated by subtracting the actual age at death from a standard life expectancy. Years lived with disability is calculated by multiplying the number of cases by the duration of the disease and a disability weight.^[10]^ All data were stratified systematically by sex, age, and geographical region. The SDI classified the countries and regions into 5 levels: high, high-middle, middle, low-middle, and low. The SDI was computed as a product of gross domestic product, educational attainment among those under 25 years, and years of schooling among those over 15 years. The index indicates the general socioeconomic standing of a nation or region and is scaled from 0 (lowest) to 1 (highest), with higher values denoting a better degree of socioeconomic development.^[12]^

### 2.2. Statistical analysis

Descriptive analyses were conducted at global, regional, and country levels to characterize the burden of CHC, including cirrhosis. The age-standardized rates (ASRs) of incidence, prevalence, deaths, and DALYs were calculated from the WHO standard population, allowing comparisons across countries and regions with different population age structures. Age standardization was performed using the following formula:


Age-standardized rate(ASR)=[∑(ai*wi)/∑(wi)]*100,000.

Where ai represents the age-specific rate for the i-th age group, and wi refers to the number of people in each i-th age group in the standard population.^[13]^ All indicators were derived from the GBD 2021 estimation framework, in which 500 posterior draws were generated to quantify uncertainty. The mean of these draws was used as the final point estimate, and the 2.5th and 97.5th percentiles defined the 95% uncertainty intervals (UIs). The precision of these estimates varies across regions and indicators. The 95% UIs provide a plausible range to facilitate a better contextual understanding of the reported trends and rankings. The changes in the burden of disease from 1990 to 2021 were described using the estimated annual percentage change (EAPC) and its 95% confidence interval (CI). The calculation of EAPC used the logarithmic linear regression model:


$$\begin{array}{c} ln\left(ASR\right)=\alpha +\beta x+\varepsilon \end{array}$$

$$\begin{array}{c} EAPC=\left(exp\left(\beta \right)-1\right)*100 \end{array}$$

Where ln(ASR) represents the natural logarithm of the ASR, *x* is the calendar year, α is the intercept, β is the slope (the regression coefficient), and ε is the error term. The linear regression model derived a 95% CI. The EAPC is the average change in the disease burden indicators on an annual basis over a specific time. When EAPC and 95% CI were both negative, it meant that there was a decline in ASR with time. When the lower limit of EAPC and 95% CI were positive, the ASR rose over time. Otherwise, the ASR was considered stable.^[14]^

### 2.3. Joinpoint regression analysis

The joinpoint regression program (version 5.0) was used to analyze the change trend of diseases worldwide and in the 5 SDI regions. The model partitions the long-term trend into segments and identifies the optimal trend within each segment. The average annual percent change, annual percent change, and its 95% CI can be estimated using the model, thus testing the overall direction of change across the entire time interval and the characteristic change in different time periods comprehensively.^[15]^ The default method for model selection in the joinpoint software is the Monte Carlo permutation test.

### 2.4. Decomposition analysis

We applied the Das Gupta decomposition framework to quantify the relative contributions of population growth, population aging, and epidemiological changes to differences in the absolute numbers of incidence, prevalence, deaths, and DALYs between 1990 and 2021. In this framework, the total number of each burden indicator is expressed as the product of 3 components: total population size, age structure, and age-specific rates. Estimates were constructed by sequentially replacing the 1990 values of these components with the corresponding 2021 values, and the contribution of each component was calculated by averaging across all possible replacement orders to reduce dependence on the sequence of decomposition. Population growth therefore represents the change attributable to expansion of the total population while holding age structure and age-specific rates constant; aging represents the effect of shifts in the population age distribution; and epidemiological change represents the residual effect of changes in age-specific disease rates, which may reflect changes in transmission, diagnosis, treatment access, prevention, and disease management.^[16–18]^ The analysis was carried out at a global level and was further stratified by SDI region, which made it possible to explore the variation in these aspects across socioeconomic contexts.

### 2.5. Health inequality analysis

The application of this technique is a means of examining how the burden of CHC, including cirrhosis, varies across countries and regions, providing evidence to guide public health policy development. Absolute and relative health inequalities were measured using the slope index of inequality (SII) and the concentration index. Regression analysis determined SII, and it represents the relationship between disease burden indicators and socioeconomic status. The concentration index, derived by numerically integrating the area under the Lorenz concentration curve, ranges from −1 to 1, with values indicating the degree to which health outcomes are economically dependent. The index values are interpreted as follows: a value near 0 denotes minimal inequality; a positive value indicates a concentration of the burden in high SDI regions; and a negative value reflects a concentration in low SDI regions.^[19,20]^

### 2.6. Frontier analysis

The unrealized potential to eliminate the burden of CHC, including cirrhosis, was measured through this methodology in terms of the level of socioeconomic development of countries and territories. This analysis constructs a theoretical model of the best health results and measures the difference between health results in the regions and the theoretical model, which is the unrealized health potential at the present level of development. For each indicator, we constructed a nonparametric frontier curve representing the lowest ASRs observed among countries at a given SDI level. The “effective difference” for a given country or territory was defined as the vertical distance between its observed ASR and the frontier curve at its corresponding SDI value. This difference indicates the statistical shortfall in burden reduction relative to the best-performing countries with similar socioeconomic resources, but it does not imply that the frontier is immediately attainable, operationally comparable, or causally attributable to any single policy or health system feature. Interpretation of frontier results should consider differences in surveillance quality, diagnostic coverage, healthcare infrastructure, policy environments, and data uncertainty across countries and territories.^[21,22]^ We identified countries and territories with the largest effective differences to highlight regions with substantial unfulfilled potential. We also identified countries and territories with lower SDI that appear to be maximizing outcomes within their resource constraints, as well as those with higher SDI that still have considerable room for improvement.

### 2.7. Predictive analysis

Lastly, we projected the trends in the burden of CHC, including cirrhosis, over 15 years, using the BAPC model. This approach integrates prior information and accounts for age, period, and cohort effects, providing a flexible framework for extrapolation. Projections were treated as scenario-based extrapolations under a continuation-of-trends assumption and are presented with uncertainty bounds (credible intervals), which widened progressively with increasing forecast horizon.^[23,24]^ These projections do not account for potential disruptions such as the COVID-19 pandemic, which may have affected hepatitis C screening and treatment access during 2020 to 2021 and could influence near-term trajectories.

Data from the GBD database were processed using Microsoft Excel and R software (version 4.5.0). All statistical analyses and visualizations were performed in R, utilizing packages including dplyr, ggplot2, reshape2, and other software packages. Statistical significance was established during the analysis with a *P*-value <.05. For the ecological analyses examining the relationship between ASRs and SDI, Spearman correlation coefficients *R* were calculated separately across the 21 GBD super-regions and the 204 countries and territories. These correlation analyses were computed in R software using the cor.test function. Although this function returns a *P*-value under the null hypothesis of no correlation, we did not use these *P*-values for inferential testing. GBD locations represent a modeled census of the global population rather than a random sample drawn from a larger population; therefore, conventional sampling-based significance testing is not conceptually appropriate. Instead, we interpreted the correlation results primarily by the magnitude and direction of *R*. The magnitude was described qualitatively as weak (|*R*| < 0.30), moderate (0.30 ≤ |*R*| < 0.50), strong (0.50 ≤ |*R*| < 0.70), or very strong (|*R*| ≥ 0.70). A negative *R* indicated that ASRs tended to be lower in locations with higher SDI, whereas a positive *R* indicated that ASRs tended to be higher in locations with higher SDI.

The GBD 2021 study is based on the Guidelines of Accurate and Transparent Health Estimates Reporting. The Guidelines of Accurate and Transparent Health Estimates Reporting guidelines provide optimal practices regarding health estimates reporting, such as using standardized data sources and reporting methodological pathways.^[25]^ Data from this study were accessed in accordance with the GBD protocol, which permits ethical utilization of publicly accessible data. No secondary use of GBD estimation data required ethical approval, and no individual-level data was involved. Clinical trial number: not applicable.

## 3. Results

### 3.1. Descriptive analysis of CHC, including cirrhosis burden at global, regional, and national levels

#### 3.1.1. Global level

The global trends of the absolute numbers and ASRs of incidence, prevalence, deaths, and DALYs differed significantly. The global number of incidences in 2021 was 4,477,374 (95% UI: 3,691,726 to 5,356,718), an increase of 20% compared to 3,718,034 (95% UI: 3,088,275to 4,416,139) in 1990. However, the age-standardized incidence rate (ASIR) decreased from 68.55 (95% UI: 56.69 to 81.14) per 100,000 population to 59.37 (95% UI: 48.9 to 70.74) per 100,000 population from 1990 to 2021, and the EAPC was −0.51% (95% UI: −0.54 to −0.48; Table 1). In 2021, the global number of prevalences was 138,559,102 (95% UI: 111,487,772 to 167,350,517), an increase of 27% from 109,293,619 (95% UI: 89,259,257 to 131,707,609) in 1990. The age-standardized prevalence rate (ASPR) decreased from 2174.78 (95% UI: 1776.45 to 2606.11) per 100,000 population to 1704.79 (95% UI: 1373.46 to 2061.7) per 100,000 population from 1990 to 2021, with an EAPC of −0.85% (95% UI: −0.93 to −0.77; Table S1, Supplemental Digital Content 1). The global number of deaths in 2021 was 368,273 (95% UI: 314,031 to 427,548), an increase of 43% from 257,010 (95% UI: 214,870 to 315,977) in 1990. The age-standardized mortality rate (ASMR) decreased from 6.26 (95% UI: 5.23 to 7.65) per 100,000 population to 4.29 (95% UI: 3.67 to 4.98) per 100,000 population from 1990 to 2021, with an EAPC of −1.22% (95% UI: −1.29 to −1.15; Table S2, Supplemental Digital Content 2). In 2021, the global number of DALYs was 11,822,890 (95% UI: 10,109,233 to 13,698,292), an increase of 37% compared with 8,630,240 (95% UI: 7,148,331 to 10,496,175) in 1990. The age-standardized DALYs rate (ASDR) fell from 195.3 (95% UI: 162.76 to 236.7) per 100,000 population to 137.99 (95% UI: 117.99 to 159.91) per 100,000 population from 1990 to 2021, with an EAPC of −1.14% (95% UI: −1.22 to −1.06; Table S3, Supplemental Digital Content 3).

**Table 1:** The number of incidence cases and the age-standardized incidence rate of chronic hepatitis C including cirrhosis in 1990 and 2021 at the global and regional levels, and their trends from 1990 to 2021.

Location	Number of incidence cases (95% UI) in 1990	Number of incidence cases (95% UI) in 2021	1990–2021 Percentage change (100%)	The age-standardized incidence rate/100,000 (95% UI) in 1990	The age-standardized incidence rate/100,000 (95% UI) in 2021	1990–2021 EAPC (95% CI)
Global	3,718,034 (3,088,275 to 4,416,139)	44,777,374 (36,911,726 to 53,566,718)	0.2	68.55 (56.69 to 81.14)	59.37 (48.9 to 70.74)	−0.51 (−0.54 to −0.48)
Female	1,865,149 (1,547,684 to 2,217,901)	2,213,066 (1,829,390 to 2,650,152)	0.19	69 (57.03 to 81.78)	58.94 (48.47 to 70.02)	−0.54 (−0.58 to −0.5)
Male	1,852,885 (1,539,769 to 2,205,577)	2,264,309 (1,857,326 to 2,714,624)	0.22	68.24 (56.66 to 80.87)	59.82 (49.3 to 71.61)	−0.49 (−0.52 to −0.46)
Low SDI	597,263 (488,397 to 715,087)	1,043,136 (849,077 to 1,246,135)	0.75	107.15 (88.49 to 127.8)	93.28 (77.38 to 110.68)	−0.5 (−0.56 to −0.43)
Low-middle SDI	1,060,137 (879,868 to 1,261,097)	1,296,827 (1,065,648 to 1,558,210)	0.22	86.99 (72.4 to 103)	71.77 (58.78 to 86.29)	−0.5 (−0.58 to −0.42)
Middle SDI	1,115,988 (918,345 to 1,336,501)	1,159,906 (945,979 to 1,387,756)	0.04	63.8 (52.44 to 75.81)	52.01 (42.69 to 61.99)	−0.75 (−0.84 to −0.65)
High-middle SDI	558,712 (462,337 to 663,267)	518,070 (425,294 to 625,667)	−0.07	56.87 (47.04 to 67.5)	43.4 (35.61 to 51.7)	−1.08 (−1.2 to −0.97)
High SDI	383,024 (315,188 to 459,541)	456,001 (363,876 to 560,040)	0.19	42.86 (35.38 to 50.73)	35.83 (29.29 to 42.94)	−0.78 (−0.88 to −0.67)
Andean Latin America	14,261 (11,748 to 16,979)	21,207 (17,465 to 25,028)	0.49	37.93 (31.49 to 45)	33.42 (27.38 to 39.61)	−0.41 (−0.46 to −0.36)
Australasia	11,140 (9125 to 13,239)	14,392 (11,381 to 18,010)	0.29	56.86 (46.77 to 67.34)	44.79 (35.77 to 54.55)	−0.59 (−0.75 to −0.43)
Caribbean	22,626 (18,833 to 26,873)	26,135 (21,539 to 30,843)	0.16	63.86 (53.37 to 75.81)	57.09 (47.33 to 67.46)	−0.43 (−0.49 to −0.38)
Central Asia	110,560 (91,635 to 130,878)	137,526 (113,812 to 161,685)	0.24	134.04 (111.29 to 157.98)	142.32 (117.98 to 167.12)	0.17 (0.04 to 0.3)
Central Europe	43,995 (35,849 to 52,620)	35,652 (28,508 to 43,709)	−0.19	37.03 (30.63 to 44.05)	30.21 (25.02 to 35.9)	−0.59 (−0.63 to −0.54)
Central Latin America	106,577 (87,046 to 127,619)	122,524 (100,420 to 145,993)	0.15	61.12 (50.68 to 72.6)	50.87 (42.08 to 60.54)	−0.28 (−0.44 to −0.11)
Central Sub-Saharan Africa	100,250 (82,024 to 119,618)	192,688 (158,172 to 228,806)	0.92	171.61 (142.04 to 204.61)	147.23 (120.8 to 173.31)	−0.56 (−0.62 to −0.51)
East Asia	670,256 (551,909 to 802,451)	452,527 (367,064 to 554,219)	−0.32	59.07 (48.85 to 70.57)	36.72 (29.82 to 44.33)	−1.99 (−2.25 to −1.73)
Eastern Europe	118,637 (97,189 to 141,669)	119,699 (97,621 to 145,354)	0.01	56.7 (46.6 to 67.7)	66.8 (54.55 to 79.94)	0.7 (0.51 to 0.89)
Eastern Sub-Saharan Africa	212,820 (173,075 to 255,428)	374,138 (304,760 to 448,560)	0.76	112.94 (92.66 to 134.51)	97.29 (80.19 to 115.82)	−0.56 (−0.6 to −0.52)
High-income Asia Pacific	125,041 (100,521 to 153,436)	123,287 (93,511 to 157,610)	−0.01	65.66 (53.47 to 79.15)	41.55 (32.85 to 50.98)	−2.58 (−3.23 to −1.91)
High-income North America	92,706 (75,133 to 112,658)	128,067 (102,785 to 155,229)	0.38	32.35 (26.55 to 39.01)	31.66 (25.76 to 37.56)	−0.17 (−0.24 to −0.09)
North Africa and Middle East	350,118 (290,991 to 416,297)	461,086 (369,907 to 559,148)	0.32	111.15 (92.89 to 130.79)	77.81 (62.72 to 93.88)	−0.97 (−1.06 to −0.87)
Oceania	5092 (4198 to 6186)	10,598 (8700 to 12,725)	1.08	62.75 (52.54 to 74.75)	65.41 (54.23 to 77.54)	0.03 (−0.02 to 0.08)
South Asia	641,395 (527,897 to 765,706)	819,351 (664,879 to 984,014)	0.28	59.99 (49.36 to 71.4)	50.58 (41.08 to 60.69)	−0.5 (−0.58 to −0.43)
Southeast Asia	437,035 (359,694 to 517,448)	459,610 (379,902 to 546,083)	0.05	85.95 (71.32 to 101.94)	72.77 (60.35 to 86.51)	−0.55 (−0.62 to −0.49)
Southern Latin America	14,877 (12,363 to 17,707)	21,518 (17,664 to 25,949)	0.45	30.96 (25.61 to 36.86)	29.38 (24.38 to 34.93)	0.02 (−0.07 to 0.11)
Southern Sub-Saharan Africa	32,242 (26,411 to 38,829)	49,305 (40,253 to 59,277)	0.53	65.22 (53.42 to 77.77)	65.84 (53.98 to 79.24)	−0.02 (−0.13 to 0.1)
Tropical Latin America	108,817 (86,916 to 132,371)	138,556 (110,955 to 167,823)	0.27	73.39 (59.34 to 88.4)	62.77 (50.62 to 76.59)	−0.14 (−0.4 to 0.12)
Western Europe	152,680 (124,618 to 182,412)	169,184 (135,162 to 206,493)	0.11	36.59 (30.25 to 43.64)	30.33 (24.75 to 36.06)	−0.64 (−0.78 to −0.49)
Western Sub-Saharan Africa	346,909 (283,189 to 419,738)	600,323 (487,409 to 721,558)	0.73	142.89 (117.82 to 169.75)	109.75 (91.02 to 130.16)	−0.94 (−1.05 to −0.84)

CI = confidence interval, EAPC = estimated annual percent change, SDI = socio-demographic index, UIs = uncertainty intervals.

#### 3.1.2. Regional level

In the socioeconomic level of development, the five SDI regions showed great variation. From 1990 to 2021, low SDI regions experienced the largest increases in absolute numbers across all indicators, with a 75% increase in the number of incidences, an 85% increase in the number of prevalences, a 60% increase in the number of deaths, and a 66% increase in the number of DALYs. The best outcomes in terms of changes in the numbers of incidence, prevalence, and deaths were reported in high-middle SDI regions, with a 9% reduction in the number of incidences, no changes in the number of prevalences, and an increase in the number of deaths. High SDI regions registered the least percentage growth in the number of DALYs (12%). In terms of ASRs, in 2021, the highest values for each indicator were found in low SDI regions. ASIR, ASPR, ASMR, and ASDR were 93.28 (95% UI: 77.38 to 110.68) per 100,000 population, 2683.12 (95% UI: 2168.26 to 3222.34) per 100,000 population, 8.29 (95% UI: 6.82 to 10.13) per 100,000 population, and 249.3 (95% UI: 203.4 to 302.86) per 100,000 population, respectively. The regions with the lowest ASIR and ASPR were high SDI regions, with an ASIR of 35.83 (95% UI: 29.29 to 42.94) per 100,000 population and an ASPR of 949.64 (95% UI: 768.19 to 1152.89) per 100,000 population, respectively. The regions with the lowest ASMR and ASDR were the high-middle SDI regions, with ASMR and ASDR of 2.12 (95% UI: 1.77 to 2.56) per 100,000 population and 67.79 (95% UI: 55.41 to 83.95) per 100,000 population, respectively. The time trend analysis from 1990 to 2021 showed that the ASRs for all indicators across all SDI regions exhibited a downward trend, among which ASIR (EAPC = −1.08, 95% CI: −1.2 to −0.97) and ASPR (EAPC = −1.3, 95% CI: −1.43 to −1.16) in the high-middle SDI regions decreased most significantly, while ASMR (EAPC = −1.57, 95% CI: −1.6 to −1.54) and ASDR (EAPC = −1.58, 95% CI: −1.61 to −1.55) in the middle SDI regions decreased most significantly (Tables S1–S3, Supplemental Digital Content 1).

In 2021, from the perspective of 21 GBD regions, the highest numbers of incidence and prevalence were in South Asia, Southeast Asia, East Asia, and Western Sub-Saharan Africa. South Asia, Southeast Asia, North Africa and the Middle East had the highest numbers of deaths and DALYs. Regarding ASRs, in 2021, the ASIRs and ASPRs topped in Central Sub-Saharan Africa, Western Sub-Saharan Africa, and Central Asia. Central Sub-Saharan Africa, Eastern Sub-Saharan Africa, North Africa and the Middle East, and Southeast Asia were the regions with the highest ASMRs. Central Sub-Saharan Africa, Eastern Sub-Saharan Africa, and Southeast Asia had the highest ASDRs. The time-trend analysis from 1990 to 2021 showed that the EAPCs for ASRs of all indicators were negative in nearly all regions, with the exception that Eastern Europe exhibited positive values on EAPC and 95% CI for all indicators. The ASIR and ASPR of EAPC and 95% CI in Central Asia showed positive values. The ASDR of EAPC and 95% CI in High-income North America showed a positive value (Tables S1–S3, Supplemental Digital Content 1).

#### 3.1.3. National level

Relative to 204 countries and territories, in 2021, India and China had the highest numbers of incidence and prevalence of CHC, including cirrhosis, with an incidence of 554,629 (95% UI: 449,890 to 665,964) and 430,365 (95% UI: 347,696 to 528,079), respectively, and a prevalence of 16,514,074 (95% UI: 13,256,709 to 19,990,525) and 19,829,779 (95% UI: 15,664,995 to 24,260,962), respectively, reflecting their large population bases as well as epidemiological factors. India and Indonesia had the highest numbers of deaths and DALYs, with deaths of 54,503 (95% UI: 36,577 to 72,823) and 39,997 (95% UI: 31,857 to 49,460), respectively, and DALYs of 1,894,750 (95% UI: 1,278,047 to 2,539,841) and 1,266,654 (95% UI: 1,020,553 to 1,608,138), respectively. These rankings should be interpreted cautiously: absolute burden is heavily influenced by population size and does not alone indicate poor system performance. By the ASRs, in 2021, Mongolia recorded the highest ASIR and ASPR, with 239.82 (95% UI: 191.28 to 291.87) per 100,000 population and 8589.89 (95% UI: 6259.61 to 11,024.28) per 100,000 population, respectively. Egypt had the highest ASMR and ASDR of 45.2 (95% UI: 34.58 to 56.19) per 100,000 population and 976.16 (95% UI: 737.55 to 1228.58) per 100,000 population, respectively. In terms of the time trend analysis conducted from 1990 to 2021, Ukraine experienced the greatest increase in ASIR and ASPR, with EAPC of 1.01 (95% CI: 0.77 to 1.24) and 1.06 (95% CI: 0.77 to 1.34), respectively. Kazakhstan experienced the greatest increase in ASMR with an EAPC of 3.19 (95% CI: 2.61 to 3.79). The largest increase of ASDR was in the Russian Federation with an EAPC at 3.57 (95% CI: 2.5 to 4.65; Tables S4–S7, Supplemental Digital Content 4, Fig. 1).

**Figure 1.**
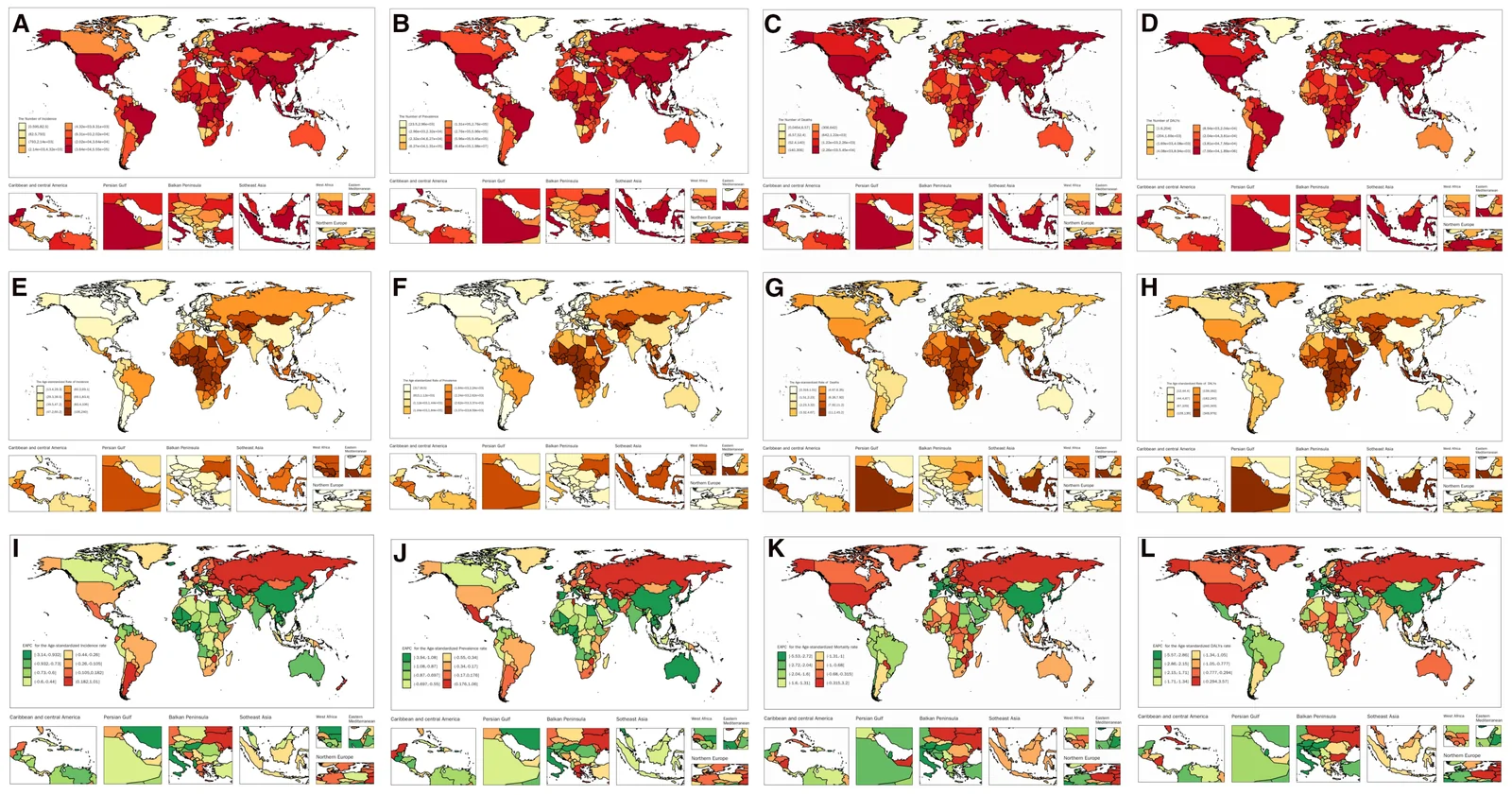
Geographical distribution and trends of chronic hepatitis C, including cirrhosis burden, in 204 countries and territories. (A–D) The number of incidence, prevalence, deaths, and disability-adjusted life years (DALYs) in 2021. (E–H) The ASIR, ASPR, ASMR, and ASDR in 2021. (I–L) The EAPC of ASIR, ASPR, ASMR, and ASDR from 1990 to 2021. ASDR = age-standardized DALYs rate, ASIR = age-standardized incidence rate, ASMR = age-standardized mortality rate, ASPR = age-standardized prevalence rate, EAPC = estimated annual percentage change.

### 3.2. Temporal trends in the burden of CHC, including cirrhosis, by sex from 1990 to 2021

The numbers of incidence and prevalence increased over time for both genders, whereas the ASIR and ASPR declined. Both the absolute number and ASR of prevalence were consistently observed to be slightly higher in females compared to males. In males, both the numbers of deaths and DALYs had a general upward trend. In females, the number of deaths exhibited a sustained upward trend, while the number of DALYs increased until 2016 and thereafter remained stable. The numbers of deaths and DALYs in males were consistently higher than those in females. By contrast, ASMR and ASDR demonstrated a declining trend in both genders alongside a persistent disparity, with males showing higher rates (Fig. 2).

**Figure 2.**
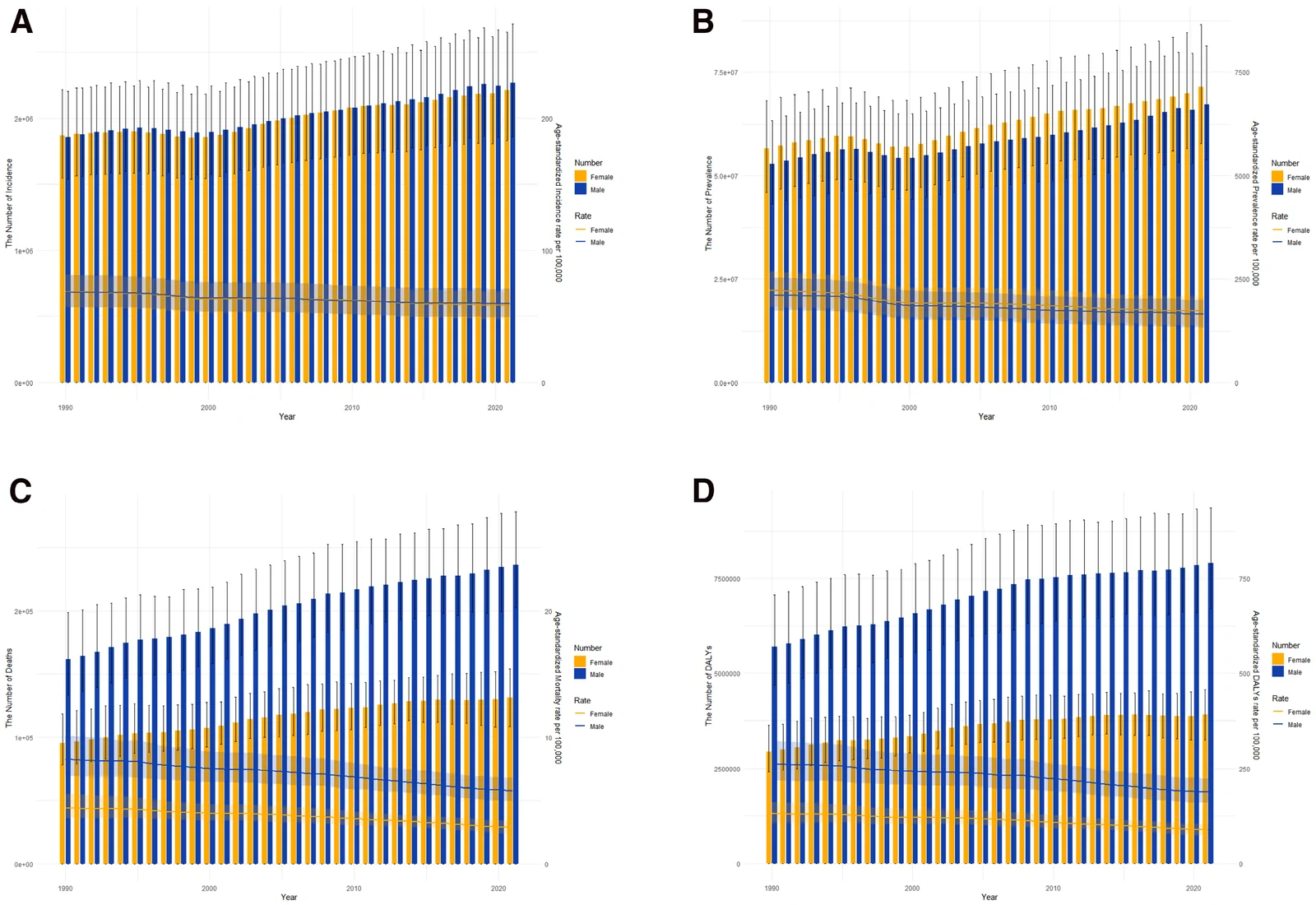
Temporal trend changes in the global number and rate of chronic hepatitis C, including cirrhosis burden, from 1990 to 2021, stratified by sex. Shading indicates the 95% UIs for the rate. (A) Number of incidence and age-standardized incidence rates. (B) Number of prevalence and age-standardized prevalence rates. (C) Number of deaths and age-standardized mortality rates. (D) Number of disability-adjusted life years (DALYs) and age-standardized DALYs rates. UIs = uncertainty intervals.

### 3.3. Burden of CHC, including cirrhosis, by age and sex in 2021

The number of incidence for both genders was highest in the < 5 years group, remained at comparable levels from young to older adulthood, and gradually declined after age 75. The incidence rates for both genders peaked in the < 5 years age group, decreased to the lowest in the adolescent group, and then gradually began to increase. The number of prevalence increased with age for both genders, peaked in the 30 to 39 years old, and then gradually declined. The prevalence rates rose steadily with age in both genders, peaking at the 90 years and older age groups. Both the number and rate of prevalence were slightly higher in females compared to males. The number of deaths for both males and females followed an inverted U-shaped curve, peaking at ages 55 to 59 years in males and at ages 65 to 69 years in females. The number of deaths in males was generally higher than in females, and it was surpassed by females in the ultra-high age group (> 90 years old). The deaths rate of both genders increased exponentially with age, and the deaths rate of males was markedly higher than that of females. The number of DALYs for both genders followed an inverted U-shaped curve, peaking at ages 50 to 54 years in males and at ages 55 to 59 years in females. The number of DALYs in males was higher than that in females, but females exceeded males in the ultra-high age group (> 90 years old). The DALYs rate exhibited a bimodal distribution, with peaks observed at ages 60 to 64 years and over 95 years in males, and at ages 70 to 74 years and over 95 years in females, respectively. The second peak in the ≥ 95 years stratum should be interpreted with caution, as it may partly reflect denominator instability in very small population strata rather than a genuine epidemiological signal, given the extremely small number of survivors and the correspondingly wide UIs in this age group. The DALYs rate for males was markedly higher than that for females (Fig. 3).

**Figure 3.**
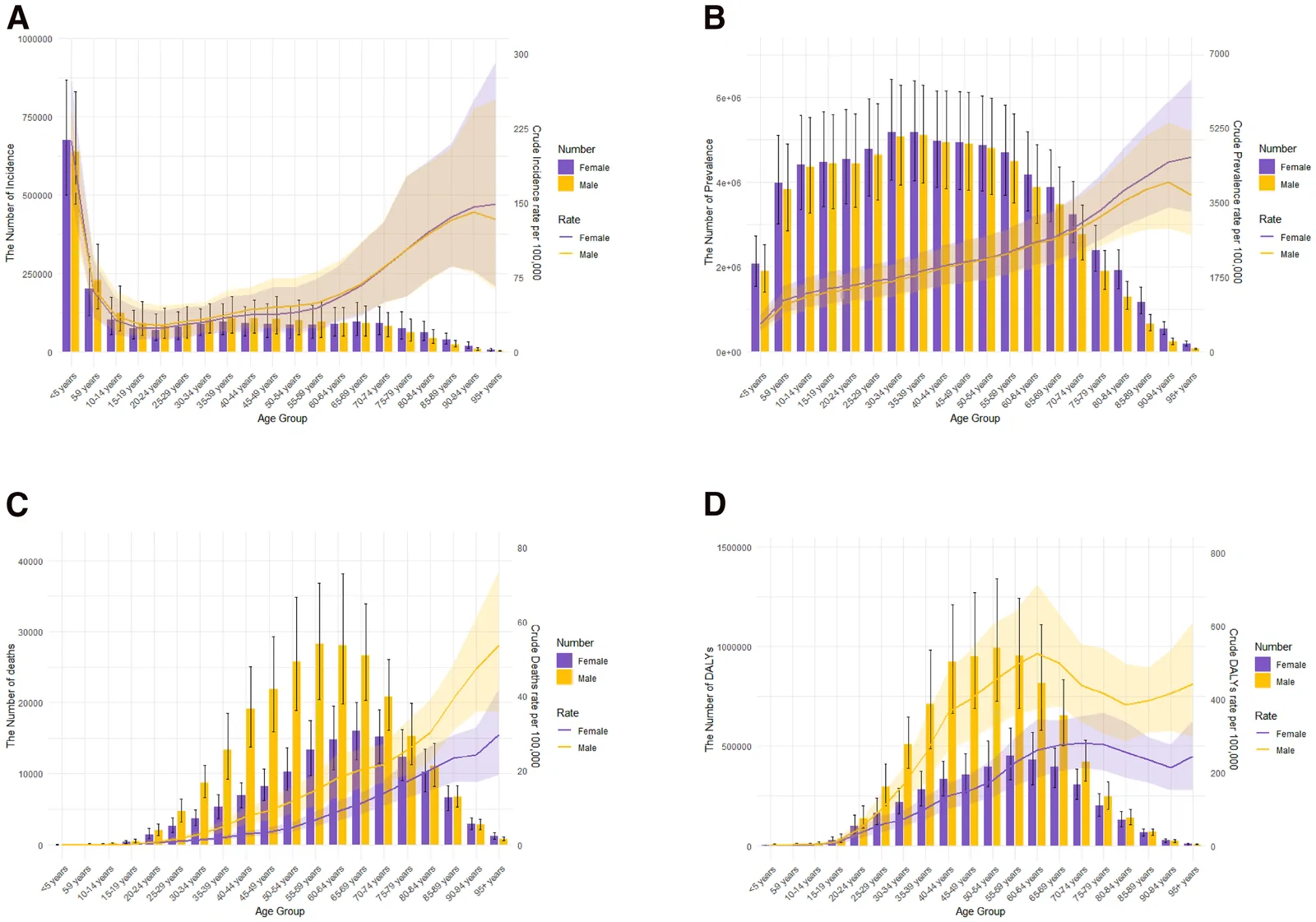
Global number and rate of chronic hepatitis C, including cirrhosis, in 2021, stratified by age and sex. Shading indicates the 95% UIs for the rate. (A) Number of incidence and age-specific incidence rates. (B) Number of prevalence and age-specific prevalence rates. (C) Number of deaths and age-specific mortality rates. (D) Number of disability-adjusted life years (DALYs) and age-specific DALYs rates. UIs = uncertainty intervals.

### 3.4. Joinpoint regression analysis

Joinpoint regression analysis results indicated that from 1990 to 2021, the ASRs of incidence, prevalence, deaths, and DALYs of CHC including cirrhosis demonstrated a negative trend on average across the world and across regions with varying SDI (average annual percent change and 95% CI were negative). ASIR and ASPR in high SDI regions reflected a slight reversal from decreasing to increasing after 2015. ASIR exhibited a slight reversal from decreasing to increasing after 2014, and ASPR remained stable after 2014. Moreover, the ASRs of all indicators in the high SDI, high-middle SDI, and middle SDI regions were generally lower than those at the global level. In contrast, the above 4 indicators in the low-middle SDI and low SDI regions were higher than those at the global level (Tables S8–S11, Supplemental Digital Content 5, Fig. 4).

**Figure 4.**
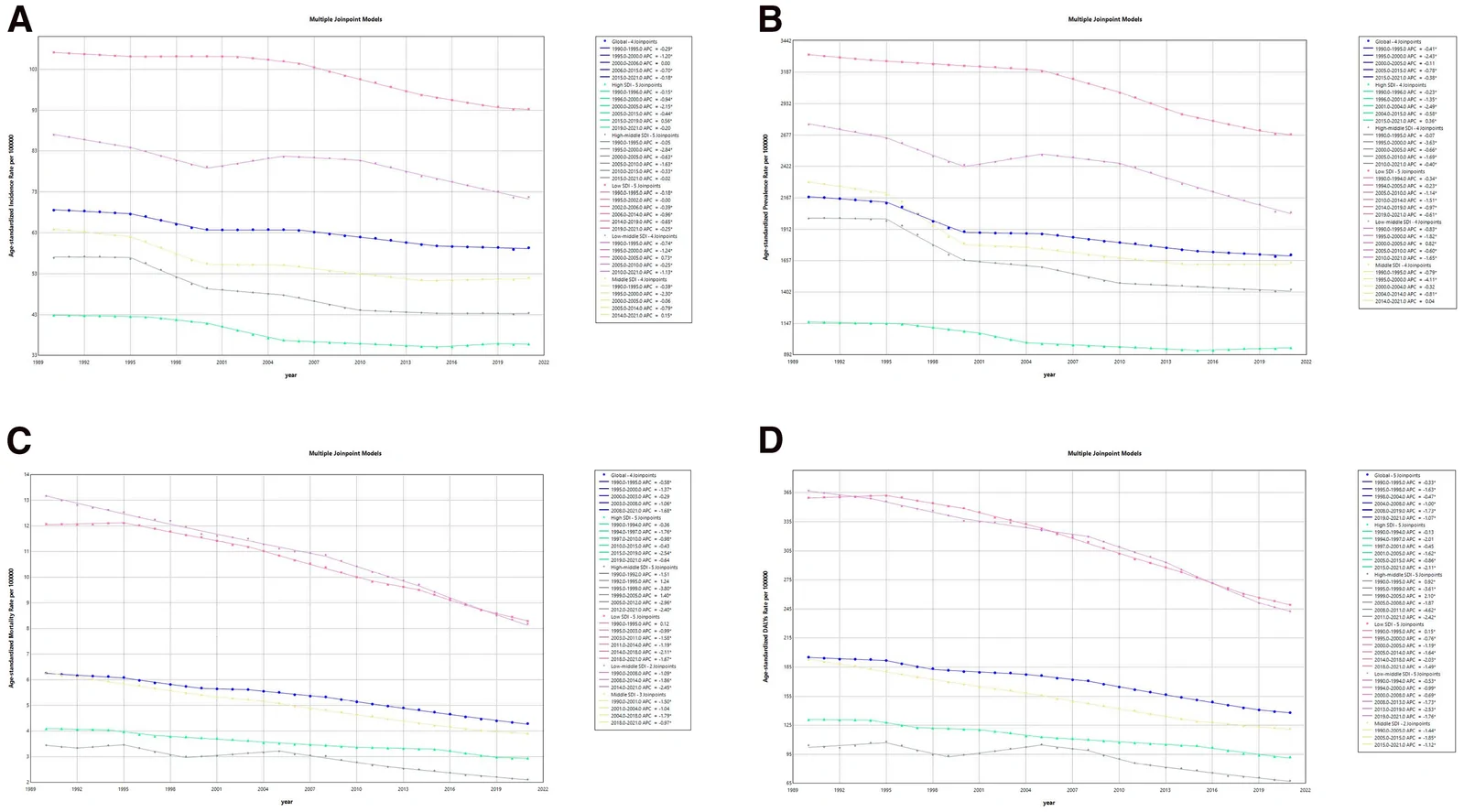
Joinpoint regression analysis on age-standardized rates for chronic hepatitis C, including cirrhosis, at global level and 5 socio-demographic index (SDI) regions from 1990 to 2021. (A) Age-standardized incidence rate; (B) age-standardized prevalence rate; (C) age-standardized mortality rate; and (D) age-standardized disability-adjusted life years (DALYs) rate.

### 3.5. Burden of CHC including cirrhosis and correlation with SDI

The ASRs of incidence, prevalence, deaths, and DALYs of CHC including cirrhosis in 21 GBD super regions were found to be negatively related to SDI in 2021. ASRs decreased with the rise of SDI. The Spearman correlation coefficients R between incidence, prevalence, deaths, DALYs, and SDI were −0.619, −0.655, −0.571, and −0.575, respectively. In line with their SDI, the ASIRs and ASPRs in Central Saharan Africa and Central Asia were significantly higher than expected. The ASMRs and ASDRs in Central Saharan Africa, North Africa and the Middle East, and Southeast Asia were significantly higher than the expected levels of their SDI (Fig. 5A, C, E, G). The ASRs of incidence, prevalence, deaths, and DALYs of CHC including cirrhosis in 204 countries and territories were also negatively correlated with SDI. The burden is less in high SDI countries and territories than in low SDI countries and territories. The Spearman correlation coefficients *R* between incidence, prevalence, deaths, DALYs, and SDI were −0.723, −0.713, −0.721, and −0.72, respectively. The ASIR and ASPR of Mongolia were markedly elevated beyond what would be anticipated according to its SDI, and the ASMR and ASDR of Egypt were significantly higher than would be anticipated according to its SDI (Fig. 5B, D, F, H).

**Figure 5.**
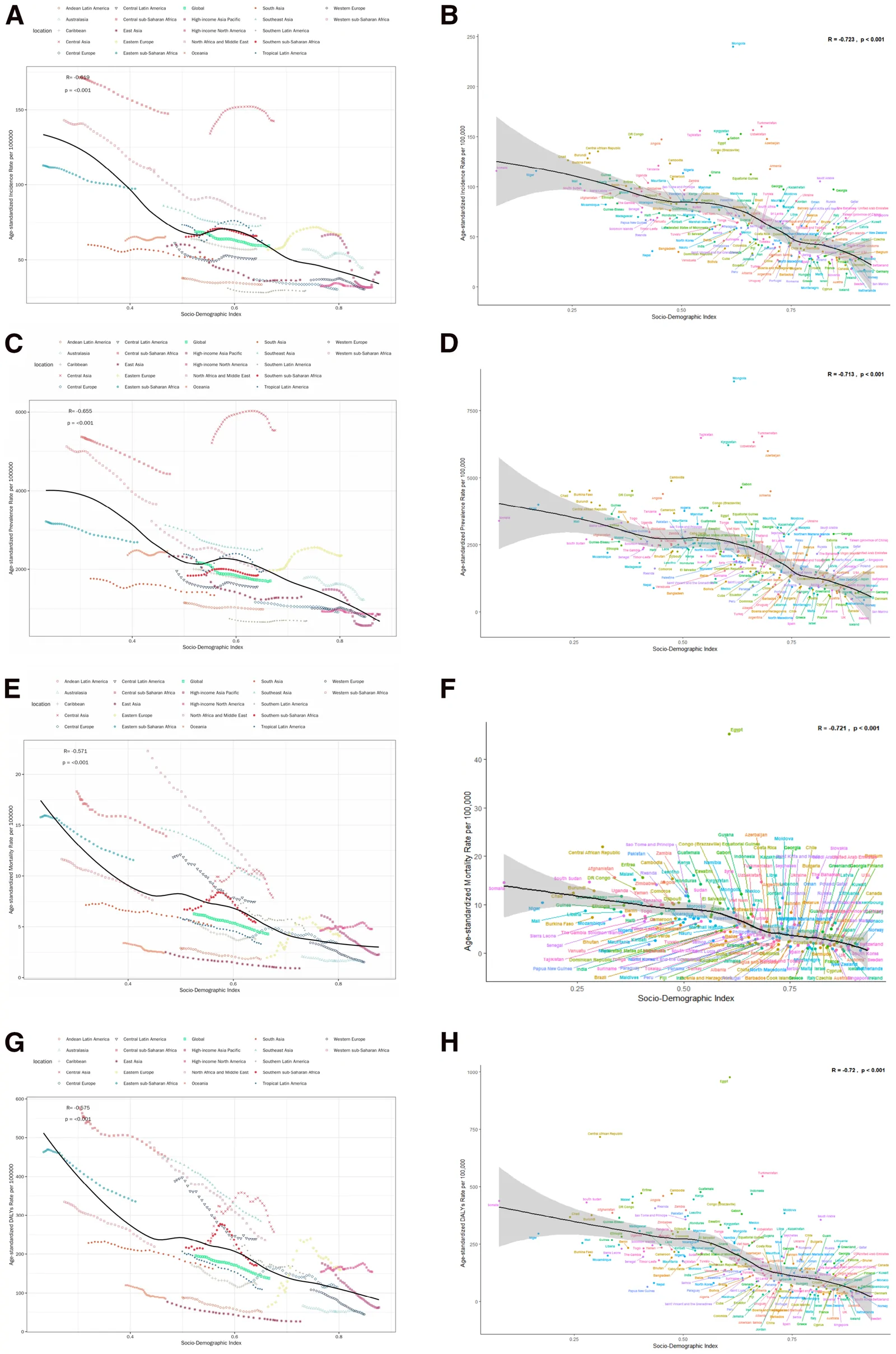
The associations between the age-standardized rates and socio-demographic index (SDI) of chronic hepatitis C, including cirrhosis, in 21 GBD regions and 204 countries and territories. (A) ASIR of 21 regions; (B) ASIR of 204 countries and territories; (C) ASPR of 21 regions; (D) ASPR of 204 countries and territories; (E) ASMR of 21 regions; (F) ASMR of 204 countries and territories; (G) ASDR of 21 regions; and (H) ASDR of 204 countries and territories. ASDR = age-standardized disability-adjusted life years (DALYs) rate, ASIR = age-standardized incidence rate, ASMR = age-standardized mortality rate, ASPR = age-standardized prevalence rate.

### 3.6. Cross-country health inequality analysis

CHC including cirrhosis demonstrated pronounced absolute and relative health inequalities linked to the SDI, with the disease burden predominantly centralized in countries with low SDI. From 1990 to 2021, the SII of incidence, prevalence, deaths, and DALYs improved from −84.72, −2838.15, −11.64, and −332.29 to −65.03, −2204.93, −8.21, and −240.55, respectively (Fig. 6A, C, E, G). These developments indicated that global health inequities involving CHC including cirrhosis have decreased over time; the disparity between the burden of CHC including cirrhosis in low and high SDI countries has shortened. However, the concentration index did not significantly improve between 1990 and 2021 (Fig. 6B, D, F, H), which means that the burden of CHC including cirrhosis remains greater in socioeconomically disadvantaged countries worldwide.

**Figure 6.**
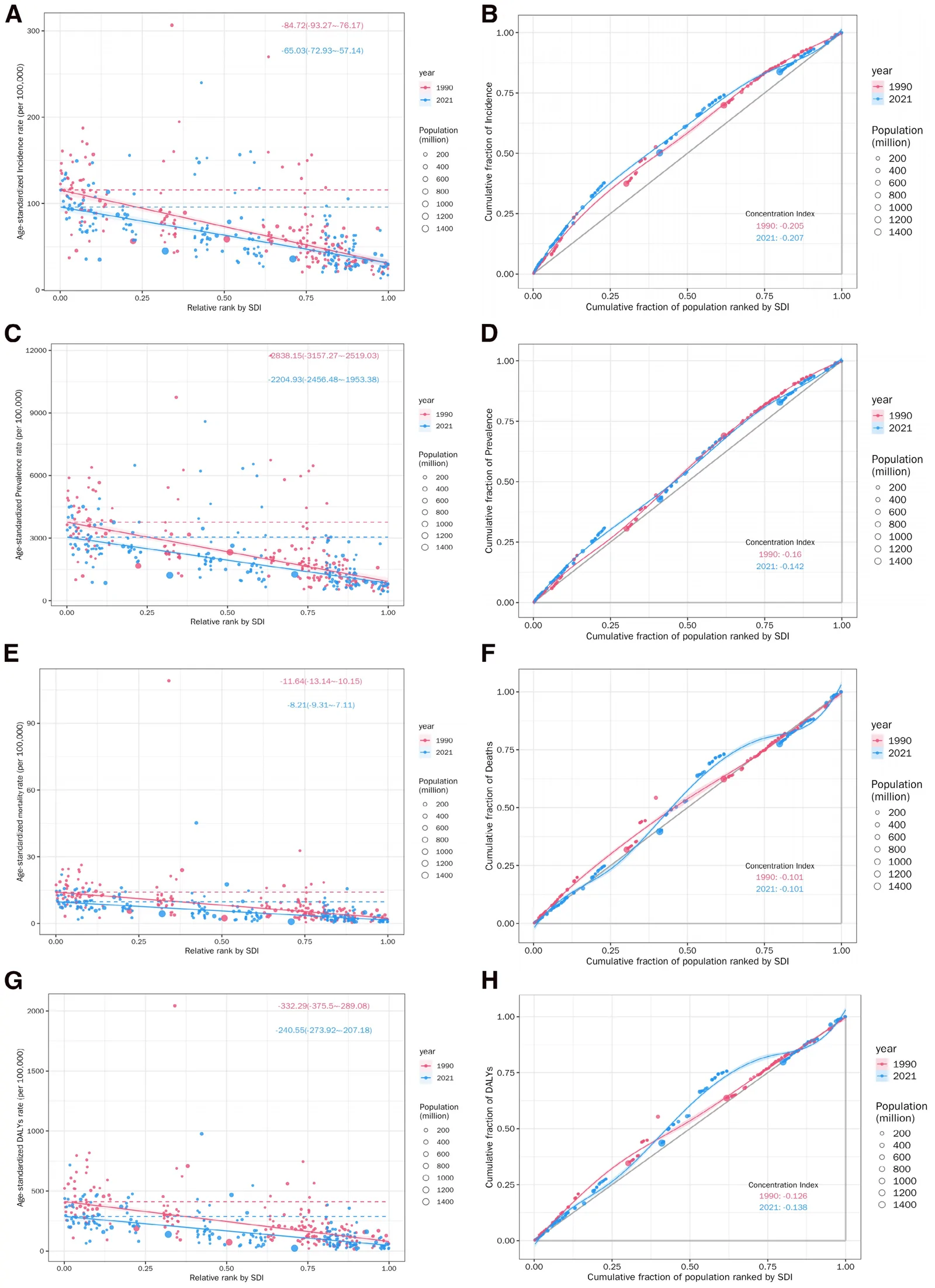
Health inequality analysis of chronic hepatitis C, including cirrhosis burden, at a global level in 1990 and 2021. A, C, E, G illustrate the inequality slope index, depicting the relationship between the socio-demographic index (SDI) and age-standardized rates for each condition, with points representing individual countries and territories sized by population. B, D, F, H present the concentration index, which quantifies relative inequalities by integrating the area under the Lorenz curve, aligning disease burden distribution with population distribution by SDI. (A,B) Health inequality analysis of age-standardized incidence rate. (C,D) Health inequality analysis of age-standardized prevalence rate. (E,F) Health inequality analysis of age-standardized mortality rate. (G,H) Health inequality analysis of age-standardized disability-adjusted life years (DALYs) rate.

### 3.7. Decomposition analysis

Decomposition analysis revealed that population growth was the primary driving force behind the increases in incidence, prevalence, deaths, and DALYs, while aging contributed to the rises in prevalence, deaths, and DALYs. In contrast, epidemiological changes substantially offset the growth in disease burden. Besides, the drivers of disease burden vary between SDI regions. In regions with a lower SDI, population growth demonstrated a stronger contribution to the increasing disease burden. In regions with a higher SDI, the impact of population aging on the increase in disease burden was greater. Epidemiological changes have consistently negatively counteracted the rise of disease burden, with their negative influence being particularly pronounced in regions with higher SDI. Notably, in the case of incidence, the contribution of aging to incidence growth was found to be negative in most regions. Separate analyses by sex showed similar patterns of results for males and females (Fig. 7).

**Figure 7.**
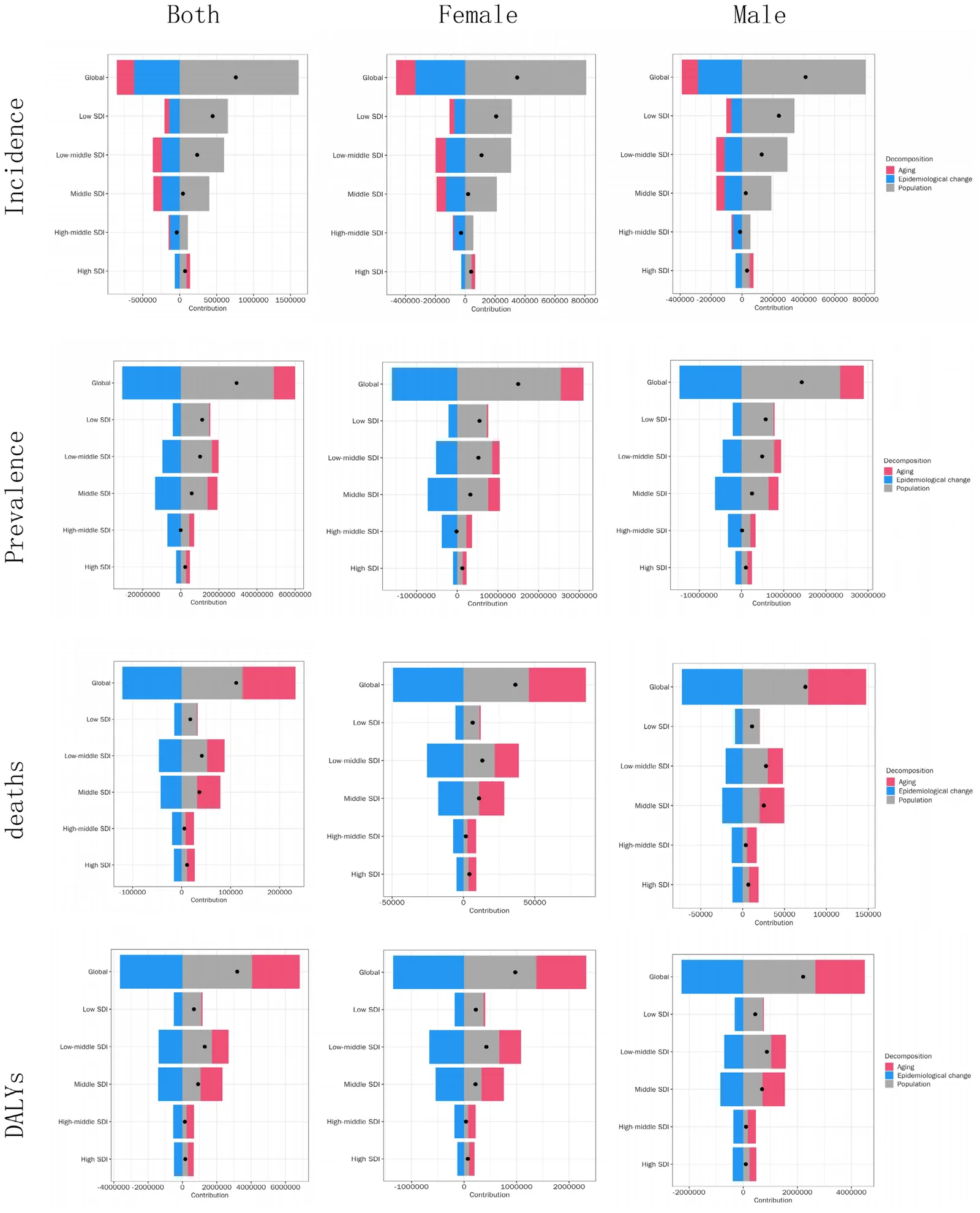
Impact of age, population growth, and epidemiological changes on the chronic hepatitis C including cirrhosis burden across global and 5 socio-demographic index (SDI) regions.

### 3.8. Frontier analysis

To assess the possibility of reducing the burden of CHC, including cirrhosis, and adjusting it by considering the socioeconomic development levels in different countries and territories, frontier analysis of the ASRs of incidence, prevalence, deaths, and DALYs was done. The boundary curve represents the lowest ASRs (best-performing or ideal burden) possible at a given SDI, and the effective difference lies in the gap between a country or territory’s observed ASRs at its current SDI level and the optimal ASRs achievable. Overall, as SDI increases, ASRs decline, and the effective differences also decline. Countries and territories with higher socioeconomic development were generally clustered closer to the frontier, whereas those with low-to-medium development exhibited greater distance from it. The largest effective differences occurred in the ASIR and ASPR in Mongolia. The largest effective differences occurred in ASMR and ASDR in Egypt. Bangladesh, Nepal, Somalia, and other low SDI countries and territories demonstrated low effective differences despite their low socioeconomic development levels. Quite to the contrary, Lithuania, Japan, Taiwan (Province of China), and other high SDI countries and territories exhibited relatively high effective differences (Tables S12–S15, Supplemental Digital Content 6, Fig. 8).

**Figure 8.**
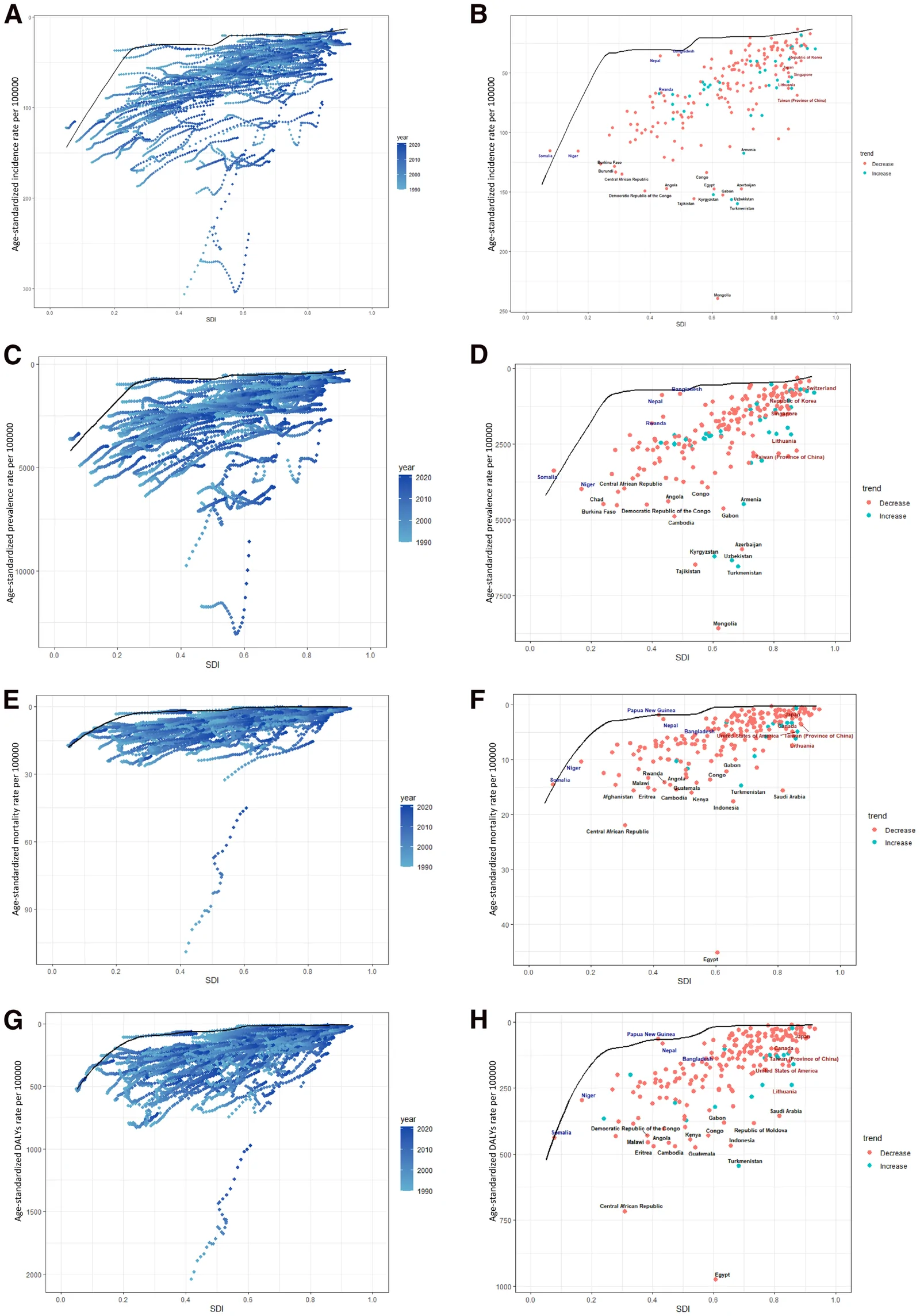
Frontier analysis based on socio-demographic index (SDI) and age-standardized rates of chronic hepatitis C, including cirrhosis. (A,B) Frontier analysis of age-standardized incidence rate. (C,D) Frontier analysis of age-standardized prevalence rate. (E,F) Frontier analysis of age-standardized mortality rate. (G,H) Frontier analysis of age-standardized disability-adjusted life years (DALYs) rate. The frontier is delineated in solid black; countries and territories are represented as dots. A, C, E, G are frontier analyses of SDI and age-standardized rates from 1990 to 2021. The trend of SDI and the age-standardized rate for each country or territory is shown in the form of a blue gradient. B, D, F, H are frontier analyses of SDI and age-standardized rates in 2021. The “effective difference” for each country or territory is the vertical distance between its observed ASR and the frontier at its corresponding SDI, indicating the statistical shortfall relative to the best-performing countries with similar socioeconomic resources. The top 15 countries and territories with the largest effective difference are labeled in black; examples of frontier countries and territories with low SDI (SDI < 0.5) and low effective difference are labeled in blue, and examples of countries and territories with high SDI (SDI > 0.85) and relatively high effective difference for their level of development are labeled in red. The orange dots indicate that the age-standardized rate in 2021 has decreased compared with 1990, while the green dots indicate that it has increased. ASR = age-standardized rate.

### 3.9. Predictive analysis

Predictive analysis indicates that the global ASIR, ASPR, ASMR, and ASDR all demonstrated a continuous decline and were expected to decrease to 51.97 (95% CI: 35.98 to 67.95) per 100,000 population, 1670.31 (95% CI: 1072.42 to 2268.21) per 100,000 population, 3.61 (95% CI: 2.51 to 4.71) per 100,000 population, and 116.32 (95% CI: 80.28 to 152.37) per 100,000 population, respectively, by 2036. Since 2022, the number of incidences has maintained its previous upward trend, but the upward trend has gradually leveled off and began to decline slowly after 2032, and is expected to be 4,664,723 (95% CI: 3,230,130 to 6,099,315) by 2036. The number of prevalences is on an increasing trend and was projected to reach 150,105,557 (95% CI: 96,613,219 to 203,597,896) by 2036. The numbers of deaths and DALYs demonstrated a consistent downward trend and were expected to drop to 324,202 (95% CI: 225,552 to 422,851) and 10,441,447 (95% CI: 7,205,844 to 13,677,050), respectively, by 2036 (Tables S16–S19, Supplemental Digital Content 7, Fig. 9).

**Figure 9.**
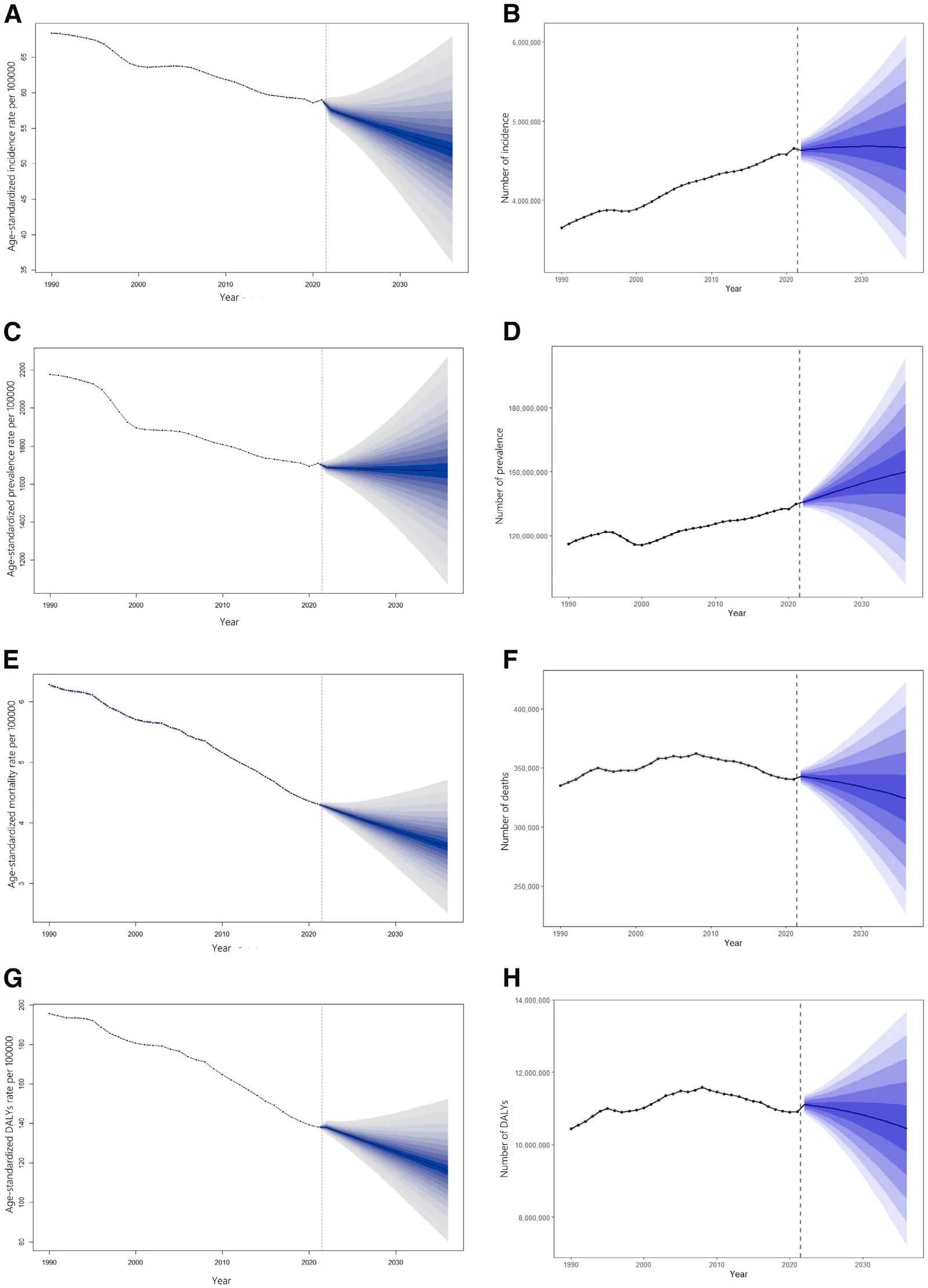
Predictions of the global burden for chronic hepatitis C, including cirrhosis, trending to 2036. (A) Age-standardized incidence rate. (B) Number of incidences. (C) Age-standardized prevalence rate. (D) Number of prevalences. (E) Age-standardized mortality rate. (F) Number of deaths. (G) Age-standardized disability-adjusted life years (DALYs) rate. (H) Number of DALYs.

## 4. Discussion

This study thoroughly examined the spatiotemporal evolution of the global burden of CHC including cirrhosis from 1990 to 2021 based on the GBD 2021 database. The results revealed that the ASRs of incidence, prevalence, deaths, and DALYs of CHC including cirrhosis experienced a global decline. The overall progress in managing CHC globally may be due to the popularization of DAAs, the improvement of blood transfusion safety and injection equipment intervention, and the improvement of access to screening and treatment.^[26]^ Nevertheless, the numbers of incidence, prevalence, deaths, and DALYs in 2021 showed an increase compared to 1990, with a concerning growth situation, especially in low SDI regions. This “growth and decline” paradox is not uncommon in epidemiology, it is attributable to the rise of the global population and the aggravation of aging.^[27,28]^ As well as enhancing our knowledge of the global epidemiological pattern of CHC including cirrhosis, the discovery also offers valuable data support and policy implications in the achievement of the strategic objective of the WHO, which is to eliminate viral hepatitis by 2030.^[8]^

### 4.1. Socioeconomic inequality and regional differences

This study discovered enormous inequalities in the burden of CHC, including cirrhosis, in various regions. The analysis of health inequality and SDI correlation revealed that the burden of CHC, including cirrhosis, was significantly correlated with SDI. Our study shows that in 2021, low SDI regions exhibited the highest ASRs across all indicators, and the increase in absolute numbers from 1990 was the greatest among all SDI regions. With a proper health system, widespread use of DAAs, and a high screening rate, the ASRs of all indicators were kept at low levels in high SDI regions such as North America, Western Europe, and Oceania.^[29,30]^ The burden of CHC, including cirrhosis, remains high in certain regions with lower SDI, such as sub-Saharan Africa, Central Asia, South Asia, and Southeast Asia. In 2021, India led the world in terms of the numbers of incidence, deaths, and DALYs. The prevalence of HCV infection in most developing countries such as India, Pakistan, and other South Asian nations is sustained by socioeconomic development, poverty, and lack of education, as well as ineffective public health strategies.^[31]^ In these regions, unsafe medical injections, blood transfusion practices, and limited drug rehabilitation measures are the primary modes of HCV transmission.^[32–34]^ Furthermore, decomposition analysis indicates that in regions with lower SDI, such as South Asia, Southeast Asia, and East Asia, population growth is the primary driver of the increase in absolute numbers. In these regions, a large population base can lead to a rise in case numbers even when age-specific risks remain stable or decline. Among the 7 WHO regions, Southeast Asia has the largest population, accounting for 25% of the global population, while its HCV disease burden represents 30% of the global total.^[35]^ Southeast Asian countries with a heavy burden of CHC, including cirrhosis, include Indonesia, Myanmar, and Vietnam. These regions are characterized by high population density, relatively young demographics, marked resource constraints, and low government health expenditure – particularly on preventive services – resulting in a substantial burden of infectious diseases including HCV.^[35]^ The numbers of incidence and prevalence in East Asia were also among the highest regions in the world, and that too might be explained by a huge role played by China. This study found that in 2021, China had one of the highest numbers of prevalence in the world. This ranking reflects its population size of over 1.4 billion, and its ASR is considerably lower than that of many countries with relatively smaller populations but higher per-capita burdens. Weak oversight of blood products early on made transfusion the dominant HCV transmission route in China; this was subsequently reversed through donor screening and voluntary donation policies,^[36]^ though recent increases in unsafe injection practices have contributed to sustained incidence.^[37]^ Several countries in Central Asia also rank among the highest in the world in terms of ASIR and ASPR, likely related to the use of inadequately sterilized medical equipment and blood transfusions in the region.^[38]^

This study found that the burden of CHC, including cirrhosis, in many countries in sub-Saharan Africa ranks among the highest in the world. Insufficient infrastructure, equipment, medical products, and financial resources have contributed to the region’s persistently high disease burden. Additionally, it is estimated that Africa currently faces a demand-based shortage of approximately 5.6 million health workers, a deficit that hinders the equitable delivery of healthcare services, including those for viral hepatitis. International migration, workforce attrition, inadequate training relative to population growth, as well as poor remuneration and working conditions, are factors contributing to this shortage.^[39,40]^ The burden of deaths and DALYs in North Africa and the Middle East was also among the highest in the world. By 2021, Egypt had the highest ASMR and ASDR in the world, a situation linked to historical HCV transmission resulting from large-scale intravenous antischistosomal injections.^[41]^ In recent years, the country has achieved remarkable progress through universal screening and large-scale DAA treatment, advancing toward the goal of eliminating hepatitis C by 2030 or even earlier, thereby setting an exemplary model for low- and middle-income countries.^[42,43]^

Disparities in disease burden across regions should also be considered in the context of substantial gaps across the HCV cascade of care, particularly in lower-income settings. The WHO Global Hepatitis Report 2024 indicates that, between 2015 and 2022, only 36% of people living with hepatitis C worldwide had been diagnosed and 20% had received curative treatment, with coverage falling markedly along the diagnosis-treatment cascade in lower-income settings. Coverage was considerably lower in the WHO African Region, where approximately 13% of people living with HCV had been diagnosed and only 3% had received treatment, while the corresponding proportions in the South-East Asia Region were 26% and 15%, respectively.^[8]^ These regional estimates are not directly equivalent to SDI categories, but they provide relevant context for the larger burden and unrealized health gains observed in many lower-SDI settings in our analysis. Recent evidence further suggests that HCV care cascades are generally less complete in lower-income settings, with deficiencies occurring across screening, confirmatory diagnosis, linkage to care, treatment initiation, and posttreatment assessment.^[44,45]^ However, these service-coverage differences should be regarded as contextual factors rather than causal explanations for the SDI-related patterns observed in the present ecological analysis.

The surprising results showed that, between 1990 and 2021, the ASRs of all indicators declined in nearly all GBD super regions, whereas Eastern Europe exhibited positive EAPCs and 95% CIs across all indicators. The EAPC and 95% CI of ASDR in High-income North America were also positive. Joinpoint analysis revealed that the ASIRs in high and middle SDI regions had a slight upward trend in recent years. This may be attributable to the high prevalence of injecting drug use and the aging population.^[46,47]^ In Europe, almost half of HCV cases are in Eastern European countries. The hepatitis C epidemic in Eastern Europe was primarily caused by intravenous drug use.^[48,49]^ It is reported that people who inject drugs contribute between 21.5 to 40.4% of the total HCV infections in Russia, Ukraine, and Georgia. Prescription opioid injections have been associated with increased incidence and prevalence of HCV infection in North America.^[50]^ New HCV infections rose significantly in the United States between 2010 and 2015, with the most significant percentage of those infected being young adults who had histories of injection drug use and use of opioid agonists such as oxycodone.^[51]^ This highlights the need to incorporate harm reduction services into elimination strategies.^[52]^ Overall, the trajectories of countries differ significantly due to variations in transmission dynamics, healthcare system capacity, economic level, and population structure. Although the ASRs of CHC, including cirrhosis, have declined globally, attention remains warranted for regions with a high burden, and disease intervention and management should be tailored to local conditions in each region.

Notably, although available data indicate a considerable disease burden in low SDI countries and regions, the actual burden may be even more severe than current statistical estimates suggest. This is due to weak primary healthcare systems, low screening coverage, and limited access to antiviral drugs, which have left a large number of potential infections undiagnosed and untreated. For example, with the exception of Egypt, nearly all African countries have yet to implement large-scale hepatitis C screening programs, leaving most infected individuals undiagnosed.^[29,53]^ Meanwhile, due to sparse surveillance data, underdiagnosis, and incomplete vital registration, GBD estimates for CHC, including cirrhosis – particularly in low SDI regions – are subject to greater uncertainty. These limitations may lead to an underestimation of the true burden in these settings. In addition, all estimates are accompanied by UIs, which represent potential variability in the data. Especially in regions with limited data, when interpreting national rankings and the degree of burden, these uncertain intervals should be taken into account. In addition, GBD mortality estimates for CHC, including cirrhosis, are subject to redistribution, in which deaths assigned to ill-defined or intermediate causes are algorithmically reallocated across underlying etiologies. The proportion and distribution of these codes vary by national vital registration quality, which may differentially affect mortality rankings in low-SDI settings with less complete cause-of-death certification. Consequently, national-level mortality comparisons should be interpreted as modeled estimates rather than precise measurements of true underlying cause-specific mortality.

### 4.2. Gender and age differences

The burden of CHC, including cirrhosis, varies by gender and age. In line with previous natural history and cohort studies, male gender is an independent predictor of more rapid fibrosis, and other factors, including increased alcohol exposure, are also associated with disease progression in hepatitis C.^[54–56]^ Comparatively, estrogen can inhibit liver fibrosis, act as an endogenous antioxidant by reducing reactive oxidant species, and regulate the production of cytokines to protect females against inflammation-induced liver injury.^[57]^ Estrogen helps to protect females during progression; fibrosis develops more slowly, whereas after menopause, the protective effect becomes less powerful, and the risk of fibrosis and complications increases.^[58]^ From 1990 to 2021, females had a somewhat higher prevalence level than males, and females had fewer deaths and DALYs than males. However, in the ultra-high age group, there was a narrowing of deaths and DALYs disparities between males and females, and finally, females surpassed males. Females are reported to have a stronger immune response than males. It is also believed that this is why females have a much longer life expectancy compared to males.^[59]^ The relative clustering of complications and deaths was observed in females at older ages because of their longer life expectancy and the cumulative burden of disease.^[46]^ The incidence numbers in both genders showed higher levels starting from the < 5 years age group, which may be related to vertical transmission from mother to child. The overall proportion of mother-to-child transmission was generally small, but in certain areas and among human immunodeficiency virus/high viral load pregnant women, the risk was greatly exaggerated.^[60,61]^ Therefore, enhanced antenatal screening and mother-to-child transmission prevention should be promoted alongside early intervention in childhood. Furthermore, as populations age, the accumulation of previously untreated infections in older cohorts has shifted the peaks of prevalence, deaths, and DALYs toward advanced age. This indicates that future hepatitis C control strategies may need to simultaneously address the dual challenges of high-risk younger populations and elderly patients while strengthening surveillance for cirrhosis and management of related complications.^[46]^

### 4.3. Decomposition analysis

This study quantified the driving forces behind changes in the global burden of CHC, including cirrhosis, from 1990 to 2021 using factor decomposition. The results suggested that there was an overall increase in the burden due to population growth and aging, but epidemiological changes (possibly due to medical advances, preventive measures, or better disease management) were important in alleviating the burden. This study found that, regarding incidence, population growth was the source of an increase in incidence in most regions, and the effects of aging and epidemiological changes on incidence had a negative relationship. This finding implies that the elderly are not the primary group for new infections, and incidence growth is primarily spurred by population expansion, particularly in the presence of continuous transmission among young or middle-aged groups, such as through exposure to risk behaviors, including mother-to-child vertical transmission, injection drug use, dangerous medical practices, and unsafe blood transfusions. However, indicators such as prevalence, deaths, and DALYs are highly influenced by aging, since uncured infections are more prevalent in older age groups, with disease progression, complications, and deaths being more common in middle-aged and older age groups. The “entrance” of HCV infection is in young and middle-aged groups, and the “exit” of deaths is in elderly groups. Such a demographic phenomenon of “asynchronous entry and exit” implies that prevention and control measures should be undertaken in 2 respects: regulate the risk of new exposure and infection among young and middle-aged groups, and mitigate complications and deaths in the elderly. The aging of the population with CHC including cirrhosis shows that although DAAs have reduced HCV-related liver transplantation and improved outcomes, patients with advanced fibrosis, cirrhosis, or persistent hepatocellular carcinoma risk will continue to require specialist follow-up and, in some cases, transplant assessment.^[62,63]^ In addition, multimorbidity, including cardiovascular, metabolic, and renal diseases, is increasingly common among older HCV patients and may complicate long-term management.^[64]^ Even after viral cure, patients with established cirrhosis require ongoing surveillance for hepatocellular carcinoma and cirrhosis-related complications. Therefore, CHC, including cirrhosis, management strategies should be accompanied by strengthened chronic liver disease care pathways, multidisciplinary management, and long-term surveillance systems to address the evolving healthcare needs of aging patient populations.^[65,66]^ Moreover, our observation of substantial regional variations in the impacts of population growth, aging, and epidemiology highlights the need for optimized, region-specific global strategies for prevention and control. In low SDI regions, population growth was the primary driver behind the rising disease burden, and the offsetting effect of epidemiological progress was comparatively weak. This could be indicative of the absence of HCV medical intervention, prevention strategies, or disease management capacity in low SDI or low-income countries, which makes it hard to effectively control the burden of HCV.^[33,67]^ On the contrary, the contribution of aging to the rise of disease burden was larger in regions with higher SDI, which could be linked to the prolonged natural survival time of HCV-infected people, indicating that the fibrosis surveillance and complication management of elderly patients with long-term infection is stronger in these regions. The epidemiological factors had a greater reduction impact on disease burden in regions with middle and high SDI as compared to regions with low SDI. It demonstrates the advantages of medical intervention and prevention systems in these areas.^[68]^ Furthermore, the results of this study indicated that despite the fact that the numbers of incidence, prevalence, deaths, and DALYs of CHC, including cirrhosis, have been increasing throughout the world in the last 30 years, the ASRs of those indicators have been decreasing. The contribution of improved epidemiology was indirectly validated. Patterns of high concordance between males and females in drivers of disease burden can indicate that sex does not significantly contribute to variation in drivers of disease burden.

### 4.4. Frontier analysis

Frontier analysis revealed significant disparities in health gains for CHC, including cirrhosis, between 1990 and 2021 across countries and regions with different levels of socioeconomic development. High SDI countries exhibited relatively smaller unrealized health gains, whereas low SDI countries showed larger unrealized health gains. However, certain high SDI countries and regions demonstrated high effectiveness differences, suggesting a mismatch between their disease burden and level of economic development. In Lithuania, the primary transmission routes are intravenous drug use, tattooing, and blood transfusions and donations prior to 1993.^[69]^ In Taiwan (province of China), transmission is mainly iatrogenic, with a high incidence observed in remote rural areas lacking medical resources.^[70]^ These findings highlight the importance of considering region-specific transmission patterns, healthcare accessibility, and health-system organization when interpreting frontier gaps. Because the frontier compares observed ASRs with the best-performing countries at similar SDI levels, differences in effective gaps may partly reflect variation in how available resources are translated into screening, diagnosis, linkage to care, and treatment delivery, rather than socioeconomic resources alone. In this context, simplified and decentralized HCV service-delivery models may offer practical approaches to reducing losses along the care cascade, particularly in resource-constrained settings. A global systematic review and meta-analysis found that fully decentralized HCV care was associated with higher linkage to care and treatment uptake than partially decentralized or centralized models in several key populations.^[71]^ WHO also recommended decentralization, integration of HCV services into primary care and other existing services, task sharing to trained nonspecialist providers, and simplified diagnostic pathways, including point-of-care and reflex HCV RNA testing.^[72]^ For countries with large unrealized health gains at relatively low SDI levels, such approaches may help narrow diagnosis and treatment gaps and support movement toward better outcomes achievable at comparable levels of socioeconomic development. However, these interpretations should remain cautious. The frontier is derived from the best-performing countries with available data and is therefore sensitive to uncertainty in the underlying estimates, differences in surveillance quality, policy environments, and local epidemiological conditions. The effective difference should consequently be regarded as a descriptive benchmark rather than a precise measure of attainable improvement, and the present ecological analysis cannot establish that differences in service-delivery models caused the observed frontier gaps or that implementing any specific model would necessarily reduce them.

### 4.5. Predictive analysis

BAPC projections indicate that the ASRs for all indicators of CHC, including cirrhosis burden, will continue to decline globally through 2036, suggesting a gradual reduction in the relative risk of the disease and reflecting the progress made in global efforts to control this condition. This positive shift may be strongly connected with the advancement of the disease’s prevention and management system, the extensive use of DAAs, the promotion of screening programs, and the improvement of access to treatment in recent years.^[26,68,73]^ However, even as the ASRs remained on a downward trend, the number of incidences is still on an upward trend in the short term, then decreasing gradually after 2032. Furthermore, the prevalence remained on an upward trend to 150 (95% CI: 97 to 204) million, under the assumption that historical trends continue. This indicates that despite the remarkable outcomes of the prevention and control measures, the absolute burden of CHC, including cirrhosis, remains high as the population base and the aging population, along with the accumulation of previously infected people, increase. There is a need to enhance screening and treatment of this disease, paying attention to the management of those already affected and the prevention of complications, especially in older people and densely populated areas. However, it is worth noting that BAPC projections should be interpreted as scenario-based extrapolations, assuming the continuation of historical age, period, and cohort patterns. They do not account for potential future disruptions, such as policy shifts, changes in screening coverage, migration, crises, or the introduction of novel therapies. Furthermore, the CIs widen as the projection horizon extends. Therefore, the projected trajectories are best interpreted as indicative of the direction and magnitude under a status quo scenario, rather than as precise forecasts.

## 5. Limitations

There are several limitations to this study. First, the quality and completeness of GBD data vary across countries, with limited primary data in some regions introducing uncertainty into model estimates. Second, as BAPC projections cannot account for future unforeseen factors, long-term projections should be interpreted with caution. Third, this study only offers disease burden estimates at the national and regional level, and the GBD 2021 database exhibits insufficient data at the provincial or municipal level. Consequently, the unassessable nature of subregional differences in disease burden limits the relevance of the results for local public health policy. Fourth, the GBD 2021 database does not provide relevant data regarding the distribution of HCV genotypes, and this study failed to distinguish the epidemiological characteristics of different genotypes of HCV in different regions. Fifth, the SDI-ASR correlation and inequality analyses are ecological analyses based on country- or region-level estimates. The observed associations cannot be interpreted as individual-level relationships or as evidence that socioeconomic development causally determines individual HCV risk. More thorough studies are needed in the future.

## 6. Conclusions

In conclusion, between 1990 and 2021, the ASRs of all indicators for CHC, including cirrhosis, declined globally, yet the absolute burden increased because of demographic change. Pronounced regional inequalities persist, with a greater disease burden observed in regions with lower SDI. Given gaps in surveillance data, underdiagnosis, and variable quality of vital registration systems across countries, uncertainty remains in future estimates.

## Acknowledgments

We would like to thank the Global Burden of Disease Study 2021 and its contributors for providing the data.

## Author contributions

**Conceptualization:** Shicheng Gu, Mingqi Hong.

**Formal analysis:** Shicheng Gu, Chenyu Jiang.

**Investigation:** Yaojian Shao.

**Methodology:** Shicheng Gu, Mingqi Hong.

**Software:** Yaojian Shao, Chenyu Jiang.

**Validation:** Mingqi Hong.

**Visualization:** Zhenjun Yu, Mingqi Hong.

**Writing – original draft:** Shicheng Gu, Yaojian Shao, Chenyu Jiang, Zhenjun Yu, Mingqi Hong.

**Writing – review & editing:** Zhenjun Yu, Mingqi Hong.






































