# Supplementary material for: Global, regional, and national burden of chronic hepatitis C including cirrhosis from 1990 to 2021, alongside projections for the next 15 years
Source: Medicine (Baltimore). 2026 Oct 16;105(42):e51118. doi: 10.1097/MD.0000000000051118 (PMC13681144; doi:10.1097/MD.0000000000051118)
Supplement: Supplementary file 1 [file medi-105-e51118-s001.docx]

Supplementary Table S1. The number of prevalence cases and the age-standardized prevalence rate of chronic hepatitis C including cirrhosis in 1990 and 2021 at the global and regional levels, and their trends from 1990 to 2021.

| location | Number of prevalence cases (95%UI) in 1990 | Number of prevalence cases (95%UI) in 2021 | 1990-2021 Percentage change(100%) | The age-standardized prevalence rate/100000 (95%UI) in 1990 | The age-standardized prevalence rate/100000 (95%UI) in 2021 | 1990-2021 EAPC (95%CI) |
| --- | --- | --- | --- | --- | --- | --- |
| Global | 109,293,619 (89,259,257~131,707,609) | 138,559,102 (111,487,772~167,350,517) | 0.27 | 2174.78(1776.45~2606.11) | 1704.79(1373.46~2061.7) | -0.85 (-0.93 ~ -0.77) |
| Female | 56,479,943 (46,083,024~68,141,656) | 71,464,835 (57,740,639~86,597,045) | 0.27 | 2231.96(1819.69~2681.75) | 1743.87(1405.21~2114.22) | -0.85 (-0.93 ~ -0.76) |
| Male | 52,813,676 (43,097,935~63,364,844) | 67,094,266 (53,944,911~81,297,199) | 0.27 | 2119.43(1737.49~2535.49) | 1665(1341.37~2014.37) | -0.86 (-0.93 ~ -0.78) |
| Low SDI | 13,158,818 (10,563,489~16,020,102) | 24,368,711 (19,489,866~29,532,069) | 0.85 | 3330.63(2696.09~3992.91) | 2683.12(2168.26~3222.34) | -0.74 (-0.82 ~ -0.66) |
| Low-middle SDI | 27,063,767 (22,038,281~32,622,570) | 37,168,572 (29,650,823~45,276,583) | 0.37 | 2762.59(2271.7~3298.04) | 2049.35(1640.24~2497.14) | -0.83 (-0.93 ~ -0.72) |
| Middle SDI | 36,428,055 (29,550,685~44,288,641) | 42,134,763 (34,092,245~50,945,753) | 0.16 | 2294.46(1875.41~2761.49) | 1642.8(1328.41~1986.5) | -1.19 (-1.37 ~ -1.01) |
| High-middle SDI | 21,286,599 (17,391,906~25,735,253) | 21,221,625 (17,156,141~25,601,651) | 0 | 1999.99(1638.33~2417.47) | 1423.33(1151.43~1729.23) | -1.3 (-1.43 ~ -1.16) |
| High SDI | 11,269,372 (9,289,703~13,445,239) | 13,555,119 (11,109,422~16,382,616) | 0.2 | 1160.93(952.52~1385.79) | 949.64(768.19~1152.89) | -0.85 (-0.95 ~ -0.74) |
| Andean Latin America | 363,871 (292,826~443,577) | 640,882 (514,895~773,911) | 0.76 | 1153.27(942.75~1394.04) | 988.79(796.8~1190.43) | -0.5 (-0.57 ~ -0.43) |
| Australasia | 380,373 (312,628~457,193) | 460,849 (354,761~582,992) | 0.21 | 1770.63(1449.62~2132.91) | 1229.43(942~1560.02) | -1.06 (-1.25 ~ -0.86) |
| Caribbean | 651,261 (532,271~793,391) | 869,065 (707,876~1,044,090) | 0.33 | 2003.89(1635.7~2418.89) | 1749.73(1416.62~2101.86) | -0.52 (-0.57 ~ -0.47) |
| Central Asia | 3,408,787 (2,807,991~4,097,931) | 5,273,965 (4,297,899~6,360,151) | 0.55 | 5220.36(4291.03~6234.12) | 5540.23(4521.76~6669.24) | 0.18 (0.03 ~ 0.33) |
| Central Europe | 1,549,723 (1,254,551~1,877,805) | 1,300,809 (1,061,970~1,572,461) | -0.16 | 1168.32(946.85~1419.85) | 916.41(740.49~1115.53) | -0.67 (-0.74 ~ -0.6) |
| Central Latin America | 2,755,724 (2,215,394~3,374,301) | 4,029,510 (3,251,311~4,874,999) | 0.46 | 1953.97(1583.18~2366.46) | 1556.81(1257.02~1876.92) | -0.35 (-0.53 ~ -0.17) |
| Central Sub-Saharan Africa | 2,176,594 (1,754,006~2,614,275) | 4,529,861 (3,639,300~5,447,119) | 1.08 | 5372.94(4350.83~6418.79) | 4425.9(3620.38~5235.63) | -0.69 (-0.76 ~ -0.61) |
| East Asia | 27,870,133 (22,718,692~34,101,820) | 21,190,085 (16,816,248~25,854,833) | -0.24 | 2329.45(1909.15~2846.18) | 1303.5(1026.62~1607.35) | -2.31 (-2.7 ~ -1.92) |
| Eastern Europe | 4,743,560 (3,778,149~5,770,219) | 5,637,244 (4,515,845~6,828,699) | 0.19 | 1969.27(1566.36~2396.77) | 2352.89(1899.27~2869.08) | 0.77 (0.54 ~ 1) |
| Eastern Sub-Saharan Africa | 4,392,529 (3,516,341~5,326,195) | 8,539,709 (6,772,184~10,331,267) | 0.94 | 3218.05(2606.13~3847.48) | 2692.33(2187.22~3235.64) | -0.64 (-0.69 ~ -0.59) |
| High-income Asia Pacific | 2,917,499 (2,349,999~3,583,610) | 2,780,548 (2,148,988~3,471,822) | -0.05 | 1480.19(1196.63~1804.47) | 859.73(688.5~1064.84) | -3.14 (-4.08 ~ -2.2) |
| High-income North America | 2,877,819 (2,310,391~3,503,563) | 4,034,923 (3,286,972~4,881,104) | 0.4 | 937.82(750.26~1141.23) | 870(700.67~1054.2) | -0.35 (-0.44 ~ -0.26) |
| North Africa and Middle East | 8,335,193 (6,828,618~9,948,287) | 11,334,889 (8,892,038~13,932,605) | 0.36 | 3224.85(2680.68~3826.14) | 1954.55(1545.67~2399) | -1.4 (-1.51 ~ -1.3) |
| Oceania | 139,052 (111,595~170,240) | 317,726 (256,980~386,384) | 1.28 | 2367.21(1929.45~2887.02) | 2463.94(2006.22~2958.65) | -0.01 (-0.07 ~ 0.06) |
| South Asia | 16,242,896 (13,063,796~19,885,115) | 23,569,088 (18,844,147~28,778,964) | 0.45 | 1762.05(1434.94~2118.95) | 1342.99(1079.5~1630.58) | -0.74 (-0.88 ~ -0.61) |
| Southeast Asia | 13,418,339 (10,934,145~16,196,020) | 17,974,141 (14,592,605~21,571,085) | 0.34 | 3124.65(2541.21~3758.45) | 2525.14(2053.43~3029.61) | -0.74 (-0.83 ~ -0.66) |
| Southern Latin America | 357,437 (293,098~427,129) | 532,452 (437,336~638,326) | 0.49 | 749.51(615.69~895.52) | 687.84(563.56~823.46) | 0 (-0.13 ~ 0.12) |
| Southern Sub-Saharan Africa | 769,980 (610,012~939,928) | 1,360,465 (1,088,685~1,653,311) | 0.77 | 1838.8(1481.93~2217.63) | 1850.62(1486.32~2254.4) | -0.03 (-0.16 ~ 0.11) |
| Tropical Latin America | 3,180,104 (2,488,997~3,921,308) | 4,775,027 (3,742,108~5,919,385) | 0.5 | 2391.79(1880.84~2928.3) | 1955.45(1528.87~2414.49) | -0.26 (-0.54 ~ 0.03) |
| Western Europe | 4,674,378 (3,860,841~5,569,966) | 4,956,342 (4,046,493~6,004,086) | 0.06 | 1011.21(831.66~1208.56) | 800.3(646.89~973.17) | -0.7 (-0.87 ~ -0.53) |
| Western Sub-Saharan Africa | 8,088,367 (6,503,908~9,882,807) | 14,451,524 (11,552,770~17,733,962) | 0.79 | 5131.49(4145.89~6173.3) | 3661.6(2961.15~4426.18) | -1.2 (-1.32 ~ -1.07) |
