# Supplementary material for: Global, regional, and national burden of chronic hepatitis C including cirrhosis from 1990 to 2021, alongside projections for the next 15 years
Source: Medicine (Baltimore). 2026 Oct 16;105(42):e51118. doi: 10.1097/MD.0000000000051118 (PMC13681144; doi:10.1097/MD.0000000000051118)
Supplement: Supplementary file 2 [file medi-105-e51118-s002.docx]

Supplementary Table S2. The number of deaths cases and the age-standardized mortality rate of chronic hepatitis C including cirrhosis in 1990 and 2021 at the global and regional levels, and their trends from 1990 to 2021.

| location | Number of deaths cases (95%UI) in 1990 | Number of deaths cases (95%UI) in 2021 | 1990-2021 Percentage change(100%) | The age-standardized mortality rate/100000 (95%UI) in 1990 | The age-standardized mortality rate/100000 (95%UI) in 2021 | 1990-2021 EAPC (95%CI) |
| --- | --- | --- | --- | --- | --- | --- |
| Global | 257,010 (214,870~315,977) | 368,273 (314,031~427,548) | 0.43 | 6.26(5.23~7.65) | 4.29(3.67~4.98) | -1.22 (-1.29 ~ -1.15) |
| Female | 95,195 (78,461~118,608) | 131,572 (108,940~154,124) | 0.38 | 4.43(3.62~5.52) | 2.91(2.42~3.41) | -1.33 (-1.41 ~ -1.24) |
| Male | 161,816 (133,742~198,484) | 236,701 (202,384~278,361) | 0.46 | 8.3(6.94~10.19) | 5.79(4.96~6.78) | -1.18 (-1.24 ~ -1.12) |
| Low SDI | 29,235 (23,599~36,319) | 46,862 (38,342~56,930) | 0.6 | 12.07(9.66~15.35) | 8.29(6.82~10.13) | -1.28 (-1.38 ~ -1.19) |
| Low-middle SDI | 80,899 (65,080~105,165) | 121,991 (100,164~143,856) | 0.51 | 13.17(10.58~17.13) | 8.19(6.71~9.71) | -1.46 (-1.56 ~ -1.37) |
| Middle SDI | 68,875 (57,100~85,882) | 105,003 (91,090~121,292) | 0.52 | 6.24(5.15~7.87) | 3.9(3.36~4.49) | -1.57 (-1.6 ~ -1.54) |
| High-middle SDI | 34,486 (29,213~42,034) | 39,903 (33,316~48,531) | 0.16 | 3.45(2.95~4.18) | 2.12(1.77~2.56) | -1.54 (-1.73 ~ -1.34) |
| High SDI | 43,259 (37,658~50,183) | 54,179 (47,670~62,791) | 0.25 | 4.09(3.56~4.77) | 2.95(2.59~3.41) | -1.05 (-1.1 ~ -1) |
| Andean Latin America | 643 (441~918) | 1,088 (719~1,555) | 0.69 | 2.91(1.98~4.21) | 1.81(1.19~2.59) | -1.71 (-1.84 ~ -1.58) |
| Australasia | 477 (396~569) | 731 (595~884) | 0.53 | 2.08(1.73~2.5) | 1.53(1.26~1.83) | -0.7 (-0.91 ~ -0.49) |
| Caribbean | 1,482 (1,089~1,914) | 2,032 (1,438~2,810) | 0.37 | 5.56(4.12~7.25) | 3.81(2.71~5.27) | -1.31 (-1.52 ~ -1.1) |
| Central Asia | 3,593 (2,736~4,666) | 6,957 (5,199~9,034) | 0.94 | 7.35(5.64~9.51) | 7.88(6.03~10.13) | 0.22 (-0.19 ~ 0.62) |
| Central Europe | 6,016 (4,646~7,921) | 6,517 (5,092~8,375) | 0.08 | 4.05(3.15~5.31) | 3.43(2.7~4.39) | -1.07 (-1.3 ~ -0.84) |
| Central Latin America | 11,225 (10,112~12,465) | 19,227 (16,092~22,423) | 0.71 | 11.83(10.63~13.18) | 7.46(6.26~8.68) | -1.82 (-1.94 ~ -1.7) |
| Central Sub-Saharan Africa | 4,732 (3,396~6,255) | 9,440 (6,653~13,044) | 0.99 | 18.29(13.3~23.82) | 13.92(9.81~19.4) | -0.79 (-0.84 ~ -0.75) |
| East Asia | 22,641 (18,388~28,232) | 18,976 (14,746~24,269) | -0.16 | 2.52(2.07~3.1) | 0.9(0.71~1.14) | -3.26 (-3.38 ~ -3.14) |
| Eastern Europe | 5,400 (4,504~6,620) | 12,239 (9,959~15,010) | 1.27 | 1.95(1.65~2.37) | 4.1(3.29~5.09) | 2.32 (1.46 ~ 3.19) |
| Eastern Sub-Saharan Africa | 12,704 (10,238~15,556) | 21,941 (17,571~26,586) | 0.73 | 15.78(12.71~19.23) | 11.56(9.44~13.84) | -1.18 (-1.28 ~ -1.07) |
| High-income Asia Pacific | 12,265 (11,074~13,736) | 10,180 (8,541~11,664) | -0.17 | 6.1(5.52~6.81) | 2.29(2~2.6) | -3.3 (-3.42 ~ -3.19) |
| High-income North America | 15,185 (13,777~16,774) | 27,500 (24,400~30,698) | 0.81 | 4.61(4.18~5.09) | 4.78(4.29~5.33) | 0.24 (0.11 ~ 0.37) |
| North Africa and Middle East | 32,519 (25,990~40,757) | 42,596 (33,773~52,747) | 0.31 | 22.29(17.69~28.09) | 9.84(7.77~12.07) | -2.49 (-2.56 ~ -2.41) |
| Oceania | 128 (80~201) | 231 (159~323) | 0.8 | 3.41(2.11~5.29) | 2.43(1.71~3.39) | -1.13 (-1.24 ~ -1.02) |
| South Asia | 46,091 (35,583~62,362) | 79,486 (61,041~97,783) | 0.72 | 7.13(5.4~9.86) | 5.14(3.93~6.34) | -1.07 (-1.17 ~ -0.98) |
| Southeast Asia | 40,807 (30,757~57,867) | 64,061 (52,886~76,863) | 0.57 | 14.68(10.96~21.36) | 9.63(7.91~11.39) | -1.4 (-1.47 ~ -1.32) |
| Southern Latin America | 3,858 (3,111~4,711) | 3,938 (3,143~4,887) | 0.02 | 8.3(6.74~10.1) | 4.64(3.69~5.76) | -1.34 (-1.52 ~ -1.16) |
| Southern Sub-Saharan Africa | 2,029 (1,594~2,669) | 3,412 (2,755~4,079) | 0.68 | 6.7(5.19~9.04) | 5.42(4.41~6.43) | -0.71 (-1.15 ~ -0.26) |
| Tropical Latin America | 6,261 (5,742~6,775) | 8,689 (7,734~9,595) | 0.39 | 5.87(5.4~6.36) | 3.32(2.96~3.67) | -1.72 (-1.87 ~ -1.57) |
| Western Europe | 18,249 (14,785~22,507) | 12,482 (9,876~15,789) | -0.32 | 3.44(2.78~4.25) | 1.51(1.2~1.94) | -2.73 (-2.79 ~ -2.67) |
| Western Sub-Saharan Africa | 10,704 (7,883~14,582) | 16,551 (12,305~21,851) | 0.55 | 11.65(8.66~15.96) | 7.64(5.91~9.75) | -1.29 (-1.4 ~ -1.18) |
