# Supplementary material for: Global, regional, and national burden of chronic hepatitis C including cirrhosis from 1990 to 2021, alongside projections for the next 15 years
Source: Medicine (Baltimore). 2026 Oct 16;105(42):e51118. doi: 10.1097/MD.0000000000051118 (PMC13681144; doi:10.1097/MD.0000000000051118)
Supplement: Supplementary file 3 [file medi-105-e51118-s003.docx]

Supplementary Table S3. The number of DALYs cases and the age-standardized DALYs rate of chronic hepatitis C including cirrhosis in 1990 and 2021 at the global and regional levels, and their trends from 1990 to 2021.

| location | Number of DALYs cases (95%UI) in 1990 | Number of DALYs cases (95%UI) in 2021 | 1990-2021 Percentage change(100%) | The age-standardized DALYs rate/100000 (95%UI) in 1990 | The age-standardized DALYs rate/100000 (95%UI) in 2021 | 1990-2021 EAPC (95%CI) |
| --- | --- | --- | --- | --- | --- | --- |
| Global | 8,630,240 (7,148,331~10,496,175) | 11,822,890 (10,109,233~13,698,292) | 0.37 | 195.3(162.76~236.7) | 137.99(117.99~159.91) | -1.14 (-1.22 ~ -1.06) |
| Female | 2,935,716 (2,403,732~3,641,032) | 3,912,392 (3,244,590~4,571,732) | 0.33 | 130.46(106.45~162.5) | 89.1(74.32~104) | -1.24 (-1.33 ~ -1.14) |
| Male | 5,694,525 (4,694,239~7,066,575) | 7,910,498 (6,701,838~9,357,874) | 0.39 | 262.2(218.36~323.09) | 188.38(160.14~222.56) | -1.09 (-1.17 ~ -1.01) |
| Low SDI | 1,022,629 (823,190~1,257,887) | 1,692,538 (1,348,268~2,069,725) | 0.66 | 359.79(290.94~444.06) | 249.3(203.4~302.86) | -1.28 (-1.39 ~ -1.18) |
| Low-middle SDI | 2,706,779 (2,153,791~3,502,695) | 4,007,016 (3,316,643~4,732,831) | 0.48 | 367.14(294.57~475.08) | 242.65(200.78~285.44) | -1.27 (-1.39 ~ -1.15) |
| Middle SDI | 2,466,408 (2,045,841~3,035,415) | 3,380,258 (2,940,707~3,954,950) | 0.37 | 192.57(159.35~238.99) | 121.26(106.04~141.19) | -1.58 (-1.61 ~ -1.55) |
| High-middle SDI | 1,085,284 (901,230~1,328,130) | 1,225,200 (1,002,377~1,534,016) | 0.13 | 104.34(86.94~127.07) | 67.79(55.41~83.95) | -1.36 (-1.62 ~ -1.11) |
| High SDI | 1,340,690 (1,158,653~1,592,075) | 1,507,410 (1,314,609~1,755,618) | 0.12 | 130.79(112.7~155.27) | 92.46(80.7~107.79) | -1.13 (-1.19 ~ -1.07) |
| Andean Latin America | 21,778 (14,582~31,914) | 31,764 (20,265~46,087) | 0.46 | 88.82(59.56~128.5) | 50.94(33.1~74.48) | -2.06 (-2.22 ~ -1.91) |
| Australasia | 14,880 (12,397~17,727) | 20,387 (16,808~24,533) | 0.37 | 66.33(54.82~78.94) | 46.82(38.8~55.84) | -0.77 (-0.96 ~ -0.58) |
| Caribbean | 48,367 (34,746~64,179) | 64,346 (44,889~89,693) | 0.33 | 172.05(124.58~228.13) | 122.32(85.42~169.9) | -1.16 (-1.41 ~ -0.92) |
| Central Asia | 119,606 (87,659~157,972) | 246,089 (175,287~326,544) | 1.06 | 228.25(169.46~300.86) | 257.73(188.86~338.82) | 0.28 (-0.16 ~ 0.73) |
| Central Europe | 194,861 (145,164~261,686) | 200,259 (155,796~258,666) | 0.03 | 133.02(99.21~178.66) | 115.17(88.63~149.02) | -1.08 (-1.34 ~ -0.82) |
| Central Latin America | 412,866 (370,397~456,929) | 631,857 (530,978~744,429) | 0.53 | 388.33(348.67~431.54) | 239.06(201.02~281.24) | -1.96 (-2.11 ~ -1.81) |
| Central Sub-Saharan Africa | 174,876 (126,179~232,894) | 360,496 (252,248~497,549) | 1.06 | 577.75(417.11~759.42) | 445.67(314.98~615.06) | -0.74 (-0.79 ~ -0.69) |
| East Asia | 776,746 (615,082~973,361) | 550,972 (426,863~720,282) | -0.29 | 76.68(61.46~95.48) | 26.3(20.33~34.17) | -3.44 (-3.56 ~ -3.33) |
| Eastern Europe | 174,195 (143,790~213,699) | 449,119 (355,304~562,245) | 1.58 | 64.06(52.96~77.58) | 159.76(125.97~201.32) | 2.79 (1.79 ~ 3.8) |
| Eastern Sub-Saharan Africa | 440,540 (356,143~534,211) | 784,754 (623,033~958,980) | 0.78 | 462.84(375.97~563.7) | 335.66(267.63~407.23) | -1.22 (-1.33 ~ -1.11) |
| High-income Asia Pacific | 363,416 (327,198~411,282) | 218,220 (192,548~248,174) | -0.4 | 176.24(158.82~199.39) | 62.3(55.5~71.71) | -3.56 (-3.65 ~ -3.46) |
| High-income North America | 489,191 (444,344~537,007) | 804,105 (719,044~896,718) | 0.64 | 154.08(139.51~169.5) | 154.32(138.13~171.24) | 0.11 (-0.02 ~ 0.25) |
| North Africa and Middle East | 858,925 (699,454~1,068,493) | 1,164,034 (911,163~1,445,364) | 0.36 | 487.51(394.05~606.41) | 236.24(186.2~293.56) | -2.13 (-2.25 ~ -2.01) |
| Oceania | 5,232 (3,199~8,297) | 9,220 (6,185~13,219) | 0.76 | 119.96(74.55~188.59) | 84.6(58.42~118.39) | -1.18 (-1.3 ~ -1.07) |
| South Asia | 1,742,040 (1,357,468~2,309,428) | 2,794,838 (2,149,583~3,510,327) | 0.6 | 228.34(176.85~306.02) | 162.57(125.39~203.57) | -1.11 (-1.22 ~ -0.99) |
| Southeast Asia | 1,458,177 (1,100,775~2,016,678) | 2,069,600 (1,726,076~2,523,445) | 0.42 | 450.26(342.12~638.41) | 285.01(237.65~344.04) | -1.54 (-1.6 ~ -1.47) |
| Southern Latin America | 120,788 (95,753~147,459) | 111,567 (88,207~138,497) | -0.08 | 257.52(204.3~314.33) | 136.1(108.36~167.94) | -1.48 (-1.68 ~ -1.28) |
| Southern Sub-Saharan Africa | 75,332 (60,184~96,249) | 120,547 (95,093~146,253) | 0.6 | 216.71(171.77~283.61) | 170.54(136.03~204.72) | -0.79 (-1.29 ~ -0.28) |
| Tropical Latin America | 244,797 (222,872~267,600) | 288,268 (256,845~319,925) | 0.18 | 206.82(188.08~225.24) | 109.21(97.61~121.03) | -2.02 (-2.19 ~ -1.86) |
| Western Europe | 534,560 (424,986~676,758) | 316,937 (246,559~407,628) | -0.41 | 108.44(85.62~138.51) | 44.57(34.33~57.53) | -2.98 (-3.06 ~ -2.9) |
| Western Sub-Saharan Africa | 359,066 (264,272~493,722) | 585,511 (418,698~786,923) | 0.63 | 334.52(244.58~456.24) | 218.87(161.27~289.08) | -1.3 (-1.4 ~ -1.2) |
