# Supplementary material for: Global, regional, and national burden of chronic hepatitis C including cirrhosis from 1990 to 2021, alongside projections for the next 15 years
Source: Medicine (Baltimore). 2026 Oct 16;105(42):e51118. doi: 10.1097/MD.0000000000051118 (PMC13681144; doi:10.1097/MD.0000000000051118)
Supplement: Supplementary file 4 [file medi-105-e51118-s004.docx]

Supplementary Table S4. The number of incidence cases and the age-standardized incidence rate of chronic hepatitis C including cirrhosis in 1990 and 2021 at 204 countries and territories levels, and their trends from 1990 to 2021.

| location | Number of incidence cases (95%UI) in 1990 | Number of incidence cases (95%UI) in 2021 | 1990-2021 Percentage change(100%) | The age-standardized incidence rate/100000 (95%UI) in 1990 | The age-standardized incidence rate/100000 (95%UI) in 2021 | 1990-2021 EAPC (95%CI) |
| --- | --- | --- | --- | --- | --- | --- |
| Afghanistan | 10847.58(8835.04~12954.56) | 26493.44(21621.36~32096.85) | 1.44 | 108.99(88.77~131.95) | 93.55(76.73~110.65) | -0.62 (-0.69 ~ -0.56) |
| Albania | 1172.44(962.84~1391.93) | 854.04(691.17~1039.7) | -0.27 | 37.98(31.45~44.87) | 30.8(25.44~36.97) | -0.94 (-1.02 ~ -0.87) |
| Algeria | 16217.63(13237.42~19607.17) | 27052.47(22120.21~32379.18) | 0.67 | 73.15(59.86~87.76) | 63.78(52.63~76.23) | -0.45 (-0.5 ~ -0.41) |
| American Samoa | 19.44(15.95~23.6) | 17.82(14.5~21.04) | -0.08 | 36.08(29.81~42.91) | 40.16(33.39~47.35) | 0.26 (0.23 ~ 0.3) |
| Andorra | 18.04(14.62~21.72) | 36.16(28.35~45.1) | 1 | 34.13(28.21~40.62) | 34.1(27.9~41.33) | -0.09 (-0.16 ~ -0.03) |
| Angola | 20675.3(16819.57~24814.8) | 46777.8(37935.16~56067.55) | 1.26 | 187.36(154.59~224.1) | 147.26(120.95~174.8) | -0.86 (-0.89 ~ -0.83) |
| Antigua and Barbuda | 31.82(26.22~37.82) | 37.35(29.58~45.11) | 0.17 | 54.53(44.89~65.01) | 43.83(35.43~51.97) | -0.73 (-0.83 ~ -0.64) |
| Argentina | 8601.88(7054.59~10305.23) | 12456.74(10208.67~15075.38) | 0.45 | 26.48(21.85~31.76) | 25.7(21.17~30.75) | 0.19 (0.08 ~ 0.29) |
| Armenia | 3975.18(3250.6~4781.23) | 2988.21(2468.03~3515.22) | -0.25 | 111.88(91.95~133.97) | 117.7(97.76~138.2) | 0.4 (0.3 ~ 0.5) |
| Australia | 9949.08(8162.55~11823.31) | 12169.7(9530.57~15364.26) | 0.22 | 61.52(50.52~72.93) | 46.12(36.69~56.91) | -0.75 (-0.93 ~ -0.58) |
| Austria | 2686.46(2137.15~3272.49) | 3071.28(2440.08~3756.31) | 0.14 | 31.91(25.81~38.01) | 26.98(22.38~31.68) | -0.69 (-0.76 ~ -0.63) |
| Azerbaijan | 12704.04(10403.7~15206.02) | 12708.46(10446.94~15100.56) | 0 | 156.25(129.16~185.57) | 147.56(121.68~175.52) | -0.29 (-0.41 ~ -0.17) |
| Bahamas | 129.11(105.53~153.98) | 155.46(124.93~185.85) | 0.2 | 54.16(44.8~63.99) | 44.17(36.22~52.67) | -0.67 (-0.76 ~ -0.58) |
| Bahrain | 316.1(256.79~383.18) | 856.77(670.31~1050.22) | 1.71 | 73.38(60.24~86.96) | 62.32(50.93~75.29) | -0.58 (-0.64 ~ -0.52) |
| Bangladesh | 46572.99(38479.06~55398.06) | 51000.93(42030.82~61865.12) | 0.1 | 45.39(37.42~53.53) | 34.98(28.85~42.61) | -1.06 (-1.27 ~ -0.85) |
| Barbados | 115.03(94.53~138.87) | 125.4(100.35~151.65) | 0.09 | 47.24(38.99~56.41) | 41.54(34.17~48.9) | -0.42 (-0.45 ~ -0.38) |
| Belarus | 5241.6(4292.31~6243) | 4941.9(4054.39~6000.67) | -0.06 | 54.5(44.99~64.73) | 60.52(49.82~71.84) | 0.49 (0.31 ~ 0.66) |
| Belgium | 3044.02(2489.47~3721.36) | 4247.95(3416.37~5166.7) | 0.4 | 27.32(22.7~32.58) | 29.28(24.09~34.97) | 0.27 (0.21 ~ 0.33) |
| Belize | 128.24(104.3~155.57) | 217.55(178.22~257.75) | 0.7 | 63.98(52.24~76.59) | 54.86(44.92~64.34) | -0.52 (-0.55 ~ -0.49) |
| Benin | 7651.95(6183.76~9335.45) | 15181.3(12230.06~18274.26) | 0.98 | 124.1(102.51~147.7) | 100.59(82.49~119.79) | -0.71 (-0.73 ~ -0.69) |
| Bermuda | 27.11(22.01~32.28) | 24.32(19.37~30.07) | -0.1 | 48.07(39.47~57.46) | 35.35(28.87~42.04) | -1.06 (-1.14 ~ -0.97) |
| Bhutan | 379.92(312.05~452.99) | 498.66(412.66~591.31) | 0.31 | 63.81(52.51~76.46) | 76.4(63.54~91.72) | 0.79 (0.69 ~ 0.89) |
| Bolivia (Plurinational State of) | 2818.78(2302.21~3407.96) | 4282.11(3544.02~5136.79) | 0.52 | 43.22(35.81~51.64) | 38.38(31.74~45.92) | -0.38 (-0.41 ~ -0.36) |
| Bosnia and Herzegovina | 860.99(698.16~1026.67) | 820.4(654.58~995.45) | -0.05 | 20.67(16.98~24.57) | 20.59(16.9~24.13) | 0 (-0.09 ~ 0.09) |
| Botswana | 980.89(800.83~1182.47) | 1565.65(1286.99~1861.33) | 0.6 | 77.55(63.57~93.23) | 71.65(59~84.85) | -0.35 (-0.39 ~ -0.31) |
| Brazil | 106123.77(84706.51~129240.77) | 134563.84(107771.27~163082.42) | 0.27 | 73.65(59.47~88.77) | 62.93(50.7~76.77) | -0.14 (-0.41 ~ 0.13) |
| Brunei Darussalam | 105.4(86.08~127.27) | 235.78(187.04~287.29) | 1.24 | 53.76(43.63~64.34) | 56.02(45.15~67.76) | 0.02 (-0.03 ~ 0.07) |
| Bulgaria | 2775.28(2257.1~3323.11) | 2135.11(1715.35~2614.62) | -0.23 | 33.26(27.57~39.13) | 30.02(24.97~35.66) | -0.47 (-0.57 ~ -0.37) |
| Burkina Faso | 20498.78(16468.21~24786.73) | 35303.81(28396.92~42734.68) | 0.72 | 160.82(132.8~191.16) | 128.51(106.16~152.18) | -0.74 (-0.79 ~ -0.69) |
| Burundi | 9619.05(7797.09~11499.64) | 17441.64(14164.83~20908.74) | 0.81 | 156.14(128.32~185.38) | 133.49(108.4~159) | -0.57 (-0.6 ~ -0.54) |
| Cabo Verde | 440.82(357.36~533.1) | 426.57(350.22~508.09) | -0.03 | 107.22(88.04~126.93) | 83.65(69.2~99.36) | -0.9 (-0.95 ~ -0.86) |
| Cambodia | 23494.06(19067.79~28226.67) | 20974.05(17115.7~24983.43) | -0.11 | 153.61(126.24~182.56) | 123.41(101.41~146.43) | -0.8 (-0.87 ~ -0.74) |
| Cameroon | 19408.02(15932.23~23594.59) | 36997.21(29605.66~44676.49) | 0.91 | 145.48(121.41~174.15) | 107.48(88.31~127.21) | -1 (-1.02 ~ -0.97) |
| Canada | 7631.77(6226.24~9198.83) | 9266.74(7057.92~11765.15) | 0.21 | 27.38(22.5~32.98) | 21.84(17.26~26.97) | -0.49 (-0.69 ~ -0.29) |
| Central African Republic | 4124.75(3399.42~4963.54) | 7062.84(5826.96~8389.5) | 0.71 | 148.13(123.67~174.94) | 135.28(111.33~161.5) | -0.37 (-0.43 ~ -0.31) |
| Chad | 11938.58(9714.37~14516.72) | 29101.05(23706.28~35356.73) | 1.44 | 146.51(120.66~173.63) | 126.6(104.42~149.98) | -0.53 (-0.55 ~ -0.5) |
| Chile | 5364.76(4501.2~6344.06) | 7956.74(6438.73~9573.96) | 0.48 | 43.47(36.39~51.76) | 38.58(31.82~45.68) | -0.27 (-0.37 ~ -0.16) |
| China | 645186.12(530373.23~773476.49) | 430364.74(347696.41~528079.24) | -0.33 | 58.87(48.62~70.36) | 35.85(29.1~43.4) | -2.06 (-2.34 ~ -1.78) |
| Colombia | 18925.29(15375.91~22496.73) | 20369.85(16295.68~24491.24) | 0.08 | 55.84(46.08~66.34) | 44.72(36.18~53.39) | -0.67 (-0.82 ~ -0.52) |
| Comoros | 446.69(362.98~531.96) | 563.74(463.83~665.89) | 0.26 | 100.25(81.99~120.08) | 83.24(68.86~98.76) | -0.62 (-0.65 ~ -0.58) |
| Congo | 3754.28(3064.8~4484.05) | 6694.48(5448.78~8096.55) | 0.78 | 160.26(130.65~191.21) | 133.8(108.31~160.2) | -0.67 (-0.7 ~ -0.63) |
| Cook Islands | 8.04(6.55~9.77) | 6.24(5.08~7.49) | -0.22 | 42.44(34.8~51.04) | 36.88(30.54~43.95) | -0.46 (-0.49 ~ -0.42) |
| Costa Rica | 1795.26(1479.82~2154.12) | 2202.75(1819.78~2587.01) | 0.23 | 56.71(47.25~66.98) | 49.62(40.97~58.07) | -0.44 (-0.48 ~ -0.4) |
| Croatia | 1539.77(1240.08~1840.38) | 1262.17(1009.27~1568.44) | -0.18 | 34.25(28.28~40.79) | 27.41(22.6~32.43) | -0.81 (-0.85 ~ -0.76) |
| Cuba | 5201.19(4282.43~6179.86) | 4886.54(3893.21~5902.77) | -0.06 | 51.47(42.41~61.21) | 43.55(36.13~51.59) | -0.56 (-0.6 ~ -0.52) |
| Cyprus | 217.63(176.96~264.21) | 391.32(314.63~485.3) | 0.8 | 28(22.74~33.6) | 24.85(20.22~29.81) | -0.23 (-0.3 ~ -0.17) |
| Czechia | 3420.81(2807.57~4121.86) | 3484.45(2822.06~4173.59) | 0.02 | 35.15(29.22~41.41) | 31.01(25.84~36.54) | -0.33 (-0.46 ~ -0.21) |
| C么te d'Ivoire | 16439.36(13288.5~20028.27) | 27570.69(22506.3~33256.62) | 0.68 | 112.93(92.42~134.97) | 92.2(75.67~109.27) | -0.62 (-0.64 ~ -0.6) |
| Democratic People's Republic of Korea | 12233.35(9883.32~14863.72) | 11197.72(9271.58~13221.57) | -0.08 | 56.27(46.07~67.6) | 57.82(47.7~69.02) | -0.11 (-0.17 ~ -0.04) |
| Democratic Republic of the Congo | 68915.92(56288.91~82450.13) | 128104.73(105009.41~151544.49) | 0.86 | 169.07(139.28~201.03) | 149.24(122.56~175.45) | -0.46 (-0.53 ~ -0.39) |
| Denmark | 1481.14(1202.8~1794.44) | 1879.77(1493.82~2290.43) | 0.27 | 26.48(21.86~31.65) | 26.07(21.38~30.73) | -0.17 (-0.27 ~ -0.08) |
| Djibouti | 346.67(280~413.77) | 931.02(758.91~1116.58) | 1.69 | 93.42(76.45~112.1) | 82.99(68.28~98.67) | -0.44 (-0.46 ~ -0.42) |
| Dominica | 41.14(34.03~49) | 27.13(21.69~32.61) | -0.34 | 56.77(46.99~67.8) | 43.34(35.27~51.55) | -0.92 (-1.01 ~ -0.83) |
| Dominican Republic | 4627.47(3812.93~5502.77) | 6086.32(5048.39~7191.95) | 0.32 | 64.54(53.54~76.64) | 57.1(47.33~67.4) | -0.42 (-0.51 ~ -0.33) |
| Ecuador | 3409.79(2796.27~4050.09) | 5782.13(4751.9~6822.07) | 0.7 | 34.87(28.9~41.41) | 33.45(27.57~39.59) | -0.08 (-0.13 ~ -0.04) |
| Egypt | 167136.66(139675.28~196652.84) | 148343.76(107554.89~189153.2) | -0.11 | 306.5(257.61~358.02) | 147.55(107.69~187.04) | -1.69 (-2.04 ~ -1.35) |
| El Salvador | 4089.33(3371.59~4859.83) | 3607.91(2963.5~4266.16) | -0.12 | 68.79(57.23~81.16) | 57.42(47.11~67.89) | -0.58 (-0.65 ~ -0.51) |
| Equatorial Guinea | 796.44(650.46~956.21) | 1440.96(1166.52~1719.17) | 0.81 | 171.81(141.91~203.69) | 112.21(90.86~133.38) | -1.54 (-1.62 ~ -1.45) |
| Eritrea | 3694.86(2990.19~4448.34) | 5848.02(4805.57~6942.13) | 0.58 | 112.58(91.99~134.47) | 99.55(82.02~117.84) | -0.37 (-0.4 ~ -0.34) |
| Estonia | 783.37(648.02~940.4) | 717.55(575.55~861.43) | -0.08 | 53.46(44.31~63.3) | 57.41(47.31~68.02) | 0 (-0.12 ~ 0.13) |
| Eswatini | 597.42(485.12~727.99) | 797.27(650.07~951.69) | 0.33 | 76.53(62.35~92) | 76.88(63.34~91.68) | 0.05 (-0.02 ~ 0.11) |
| Ethiopia | 54615.9(44068.84~65378.45) | 87628.37(71514.73~105717.73) | 0.6 | 115.38(94.42~138.53) | 93.35(76.49~112.92) | -0.86 (-0.95 ~ -0.78) |
| Fiji | 331.48(268.37~401.78) | 369.28(303.07~443.78) | 0.11 | 42.84(35.2~51.57) | 41.87(34.51~50.11) | -0.03 (-0.09 ~ 0.02) |
| Finland | 1484.94(1209.72~1800.31) | 2029.12(1617.89~2504.01) | 0.37 | 27.33(22.52~32.49) | 28.16(23.08~33.6) | 0.03 (-0.07 ~ 0.13) |
| France | 17954.01(14491.07~21747.24) | 20397.99(16091.84~25124.65) | 0.14 | 28.26(23.11~33.81) | 23.47(18.8~28.13) | -0.28 (-0.5 ~ -0.06) |
| Gabon | 1983.6(1624.18~2361.42) | 2607.64(2151~3068.89) | 0.31 | 194.71(157.88~232.01) | 152.74(125.44~180.63) | -0.9 (-1.04 ~ -0.77) |
| Gambia | 996.16(809.38~1210.39) | 1842.13(1502.65~2213.78) | 0.85 | 93.02(76.86~110.62) | 79.27(65.25~93.92) | -0.5 (-0.58 ~ -0.42) |
| Georgia | 5959.03(4888.12~7050.04) | 2789.37(2142.24~3411.57) | -0.53 | 118.53(96.66~140.54) | 97.12(75.74~118.75) | -0.99 (-1.26 ~ -0.72) |
| Germany | 27280.34(22035.16~33021.92) | 30983.83(24728.4~37998.63) | 0.14 | 30.96(25.35~36.8) | 27.52(22.75~32.63) | 0.03 (-0.15 ~ 0.2) |
| Ghana | 27527.38(22870.6~32953.52) | 39470.12(32133.73~47223.41) | 0.43 | 149(123.9~175.04) | 111.18(91.46~131.38) | -1.03 (-1.09 ~ -0.98) |
| Greece | 2490.91(2017.85~3067.96) | 3015.87(2331.03~3727.99) | 0.21 | 21.39(17.76~25.7) | 21.05(16.95~25.19) | 0.14 (0.04 ~ 0.24) |
| Greenland | 17.44(14.09~20.92) | 16.96(13.67~20.36) | -0.03 | 32.47(26.8~38.58) | 30.61(25.14~35.99) | -0.27 (-0.3 ~ -0.25) |
| Grenada | 60.37(49.16~71.8) | 45.34(37~53.73) | -0.25 | 65.41(53.73~77.97) | 47.16(38.91~55.91) | -1.07 (-1.17 ~ -0.96) |
| Guam | 52.72(42.95~63.06) | 61.81(50.85~74.08) | 0.17 | 39.05(32~46.46) | 40.12(33.04~47.98) | -0.04 (-0.14 ~ 0.06) |
| Guatemala | 10261.48(8398.92~12149.79) | 11561.71(9474.44~13657.23) | 0.13 | 96.22(79.36~112.67) | 75.22(62.47~88.22) | -0.87 (-0.93 ~ -0.8) |
| Guinea | 9866.35(8035.78~11889.76) | 16234.3(13220.52~19445.3) | 0.65 | 129.84(107.37~153.98) | 108.21(88.76~127.95) | -0.6 (-0.63 ~ -0.58) |
| Guinea-Bissau | 1224.08(981.33~1479.09) | 1771.25(1428.86~2118.46) | 0.45 | 105.13(85.76~125.21) | 83.46(68.38~99.03) | -0.77 (-1.07 ~ -0.46) |
| Guyana | 609.47(497.82~729.2) | 450.99(368.46~530.18) | -0.26 | 75.08(61.57~88.75) | 61(49.88~71.65) | -0.64 (-0.72 ~ -0.57) |
| Haiti | 6599.94(5359.1~7935.73) | 9573.88(7774.55~11413.29) | 0.45 | 88.72(73~106.53) | 73.73(60.92~87.14) | -0.75 (-0.79 ~ -0.7) |
| Honduras | 4722.69(3838.29~5712.71) | 6976.94(5838.42~8335.67) | 0.48 | 81.77(67.17~97.13) | 70.79(58.64~83.39) | -0.49 (-0.5 ~ -0.48) |
| Hungary | 4034.84(3314.43~4849.86) | 2842.4(2276.25~3461.94) | -0.3 | 41.73(35.1~49.15) | 28.24(23.36~33.45) | -1.39 (-1.44 ~ -1.34) |
| Iceland | 59.12(47.99~71.06) | 49.88(33.51~66.28) | -0.16 | 22.99(18.71~27.6) | 13.42(9.51~17.67) | -1.15 (-1.54 ~ -0.76) |
| India | 458850.56(375994.34~549028.05) | 554628.66(449889.67~665963.53) | 0.21 | 56.44(46.11~67.46) | 45(36.76~54.18) | -0.79 (-0.92 ~ -0.65) |
| Indonesia | 175747.75(143624.81~210717.7) | 195609.5(160577.13~231815.75) | 0.11 | 89.45(74.28~105.92) | 78.88(65.08~93.7) | -0.4 (-0.43 ~ -0.38) |
| Iran (Islamic Republic of) | 22080.5(17535.4~26872.21) | 32270.14(25981.27~39238.68) | 0.46 | 44.7(36.65~53.94) | 39.69(32.41~47.7) | -1.01 (-1.53 ~ -0.49) |
| Iraq | 15942.4(12997.39~19293.2) | 30906.76(24993.86~36806.24) | 0.94 | 89.97(73.72~107.78) | 83.34(67.79~99.08) | -0.22 (-0.46 ~ 0.03) |
| Ireland | 970.6(780.28~1171.58) | 1378.72(1099.24~1678.46) | 0.42 | 26.95(21.97~32.26) | 24.58(19.85~29.61) | -0.19 (-0.28 ~ -0.09) |
| Israel | 1232.97(1014.84~1481.64) | 2584.17(2125.26~3077.56) | 1.1 | 25.26(20.76~30.33) | 24.6(20.1~29.48) | -0.24 (-0.37 ~ -0.1) |
| Italy | 43670.54(34995.67~53013.95) | 38536.02(29519.61~48453.27) | -0.12 | 71.39(58.59~86.48) | 51.71(41.76~62) | -1.97 (-2.31 ~ -1.62) |
| Jamaica | 1158.35(953.17~1383.26) | 1072.83(867.45~1282.94) | -0.07 | 48.97(40.32~58.24) | 41.67(34.37~49.2) | -0.49 (-0.57 ~ -0.41) |
| Japan | 105406.39(84220~130429.51) | 89690.23(67201.02~118008.35) | -0.15 | 71.12(57.66~86.53) | 41.72(32.58~51.98) | -3.14 (-3.99 ~ -2.29) |
| Jordan | 2357.45(1903.84~2846.94) | 7024.78(5695.18~8376.44) | 1.98 | 72.56(59.48~86.43) | 65.43(53.96~77.55) | -0.79 (-1.06 ~ -0.51) |
| Kazakhstan | 12780.43(10519.08~15097.43) | 16270.91(13412.3~19194.25) | 0.27 | 75.38(62.6~88.98) | 85.86(71.17~100.7) | 0.52 (0.4 ~ 0.63) |
| Kenya | 20185.45(16217.63~24357.34) | 38048.16(30927.14~46087.25) | 0.88 | 91.73(74.74~110.44) | 87.7(71.67~105.55) | -0.12 (-0.13 ~ -0.1) |
| Kiribati | 58.82(48.15~70.92) | 88.11(71.79~106.06) | 0.5 | 64.76(53.67~76.67) | 69.57(57.12~82.12) | 0.22 (0.15 ~ 0.29) |
| Kuwait | 800.83(646.16~992.9) | 2169.37(1708.19~2660.72) | 1.71 | 53.55(43.74~64.46) | 49.93(41.16~59.65) | -0.25 (-0.3 ~ -0.19) |
| Kyrgyzstan | 7991.91(6511.19~9599.64) | 11298.21(9190.6~13475.03) | 0.41 | 145.69(118.96~173.51) | 152.37(124.64~179.98) | 0.19 (0.03 ~ 0.35) |
| Lao People's Democratic Republic | 5274.12(4288.69~6362.88) | 5180.31(4268~6169.69) | -0.02 | 93.28(76.56~110.57) | 69.36(57.39~82.44) | -1.12 (-1.2 ~ -1.04) |
| Latvia | 1234.74(997.3~1473.11) | 820.2(650.73~999.74) | -0.34 | 49.65(40.95~59.23) | 47.57(38.78~56.85) | -0.04 (-0.15 ~ 0.07) |
| Lebanon | 1549.68(1281.32~1846.62) | 2495.63(2014.25~3013.52) | 0.61 | 54.76(44.81~65.49) | 43.87(35.6~52.86) | -0.77 (-1.16 ~ -0.39) |
| Lesotho | 1179.87(959.92~1418.29) | 1393.49(1137.74~1665.99) | 0.18 | 76.96(63.42~92.38) | 82.4(67.9~98.03) | 0.24 (0.22 ~ 0.25) |
| Liberia | 3795.42(3041.94~4591.28) | 5922.28(4747.86~7146.78) | 0.56 | 126.82(104.08~151.93) | 106.19(86.58~127.25) | -0.7 (-0.74 ~ -0.65) |
| Libya | 2502.06(2048.11~3035.3) | 3781.55(3000.69~4576.46) | 0.51 | 69.14(56.88~83.22) | 58.6(48.11~69.75) | -0.55 (-1.2 ~ 0.1) |
| Lithuania | 2056.32(1683.58~2420.24) | 1543.85(1242.34~1838.22) | -0.25 | 60.55(49.77~71.88) | 63.17(52.37~74.43) | 0.16 (0.06 ~ 0.26) |
| Luxembourg | 139.21(112.78~169.06) | 205.14(162.78~252.98) | 0.47 | 33.68(27.52~40.27) | 27.48(22.47~32.62) | -0.79 (-0.86 ~ -0.72) |
| Madagascar | 8668.79(7123.25~10392.79) | 16959.43(13922.65~20125.04) | 0.96 | 78.69(64.2~93.78) | 69.92(58.04~83.32) | -0.32 (-0.51 ~ -0.12) |
| Malawi | 11574.01(9412.93~13749.38) | 16446.83(13485.23~19480.09) | 0.42 | 117.02(95.88~139.49) | 97.95(80.04~116.62) | -0.69 (-0.74 ~ -0.63) |
| Malaysia | 17886.1(14663.75~21371.39) | 20946.92(17250.62~25002.28) | 0.17 | 87.58(72.53~103.4) | 76.16(62.64~90.68) | -0.44 (-0.57 ~ -0.31) |
| Maldives | 361.9(294.56~440.97) | 293.91(238.45~351.38) | -0.19 | 110.48(91.73~131.83) | 74.69(61.76~88.85) | -1.39 (-1.49 ~ -1.29) |
| Mali | 15331.35(12462.09~18902.95) | 29529.11(23937.57~35709.41) | 0.93 | 134.9(111.28~161.91) | 102.55(84.69~121.47) | -0.99 (-1.02 ~ -0.96) |
| Malta | 102.88(82.14~124.09) | 140.72(111.78~174.38) | 0.37 | 27.35(22.16~32.88) | 24.85(20.58~29.59) | -0.35 (-0.41 ~ -0.28) |
| Marshall Islands | 34.89(28.24~42.52) | 34.5(28.2~41.25) | -0.01 | 61.56(51.02~72.95) | 64.18(52.93~76.54) | 0.12 (0.09 ~ 0.15) |
| Mauritania | 2671.13(2163.61~3237.3) | 3899.92(3175.14~4686.86) | 0.46 | 112(92.08~133.62) | 85.4(70.23~101.76) | -0.94 (-0.96 ~ -0.92) |
| Mauritius | 1013.82(838.3~1209.76) | 779.35(639.8~923.12) | -0.23 | 95.44(78.89~113.73) | 81.12(67.21~95.88) | -0.62 (-0.66 ~ -0.58) |
| Mexico | 50594.69(40194.94~62237.21) | 59233.18(47932.38~71142.21) | 0.17 | 57.87(46.79~70.1) | 47.63(38.61~57.49) | 0.16 (-0.21 ~ 0.53) |
| Micronesia (Federated States of) | 71.13(57.93~86.37) | 59.04(48.41~71.06) | -0.17 | 58.07(47.49~68.94) | 62.06(51.6~74.13) | 0.17 (0.14 ~ 0.21) |
| Monaco | 10.26(8.19~12.81) | 16.31(12.87~20.57) | 0.59 | 24.74(20.24~29.55) | 29.71(24.49~35.32) | 0.65 (0.57 ~ 0.73) |
| Mongolia | 7968.55(6476.02~9605.65) | 8869.78(6965.6~10885.99) | 0.11 | 269.91(223.12~320.64) | 239.82(191.28~291.87) | 0.02 (-0.2 ~ 0.25) |
| Montenegro | 173.26(142.18~206.89) | 178.08(144.37~215.17) | 0.03 | 29.31(24~35.09) | 28.64(23.51~33.94) | -0.18 (-0.24 ~ -0.11) |
| Morocco | 18290.28(14981.3~22015.66) | 24274.24(19566.74~29266.56) | 0.33 | 78.56(64.31~94.62) | 67.55(55.02~81.04) | -0.42 (-0.78 ~ -0.06) |
| Mozambique | 11654.45(9530.51~13924.21) | 22140.76(18096.89~26500.78) | 0.9 | 89.77(74.18~107.67) | 82.21(68.21~97.91) | -0.26 (-0.28 ~ -0.23) |
| Myanmar | 44743.39(36786.97~53274.87) | 46361.9(38305.06~54349.28) | 0.04 | 98.81(81.1~117.09) | 86.72(71.78~101.92) | -0.52 (-0.56 ~ -0.49) |
| Namibia | 969.29(784.9~1181.94) | 1519.06(1241.4~1823.4) | 0.57 | 71.3(58.22~85.81) | 68.55(56.09~81.52) | -0.3 (-0.36 ~ -0.25) |
| Nauru | 6.35(5.14~7.71) | 6.82(5.53~8.22) | 0.07 | 52.84(43.35~62.98) | 58.34(48.03~69.4) | 0.31 (0.27 ~ 0.34) |
| Nepal | 7005.57(5744.83~8405.42) | 9592.61(7981.67~11378.15) | 0.37 | 37.21(30.57~44.34) | 35.65(29.5~42.3) | -0.19 (-0.44 ~ 0.07) |
| Netherlands | 3193.34(2552.95~3939.12) | 4357.86(3469.35~5341.99) | 0.36 | 19.71(15.87~23.88) | 18.95(15.37~22.71) | -0.28 (-0.49 ~ -0.08) |
| New Zealand | 1191.37(968.34~1438.85) | 2222.16(1785.5~2706.19) | 0.87 | 34.64(28.38~41.67) | 38.51(31.47~45.97) | 0.49 (0.43 ~ 0.55) |
| Nicaragua | 4460.95(3648.84~5316.17) | 4843.69(4001.7~5703.28) | 0.09 | 92.6(77.2~108.43) | 75.87(62.89~89.19) | -0.6 (-0.76 ~ -0.44) |
| Niger | 14842.44(11897.81~18249.53) | 36186.51(29258.65~44093.83) | 1.44 | 138.11(114.05~163.99) | 115.93(95.65~136.91) | -0.65 (-0.68 ~ -0.62) |
| Nigeria | 176329.12(142361.17~212817.24) | 291323.03(235991.48~353488.14) | 0.65 | 156.86(128.92~186.72) | 113.42(93.3~135.8) | -1.18 (-1.36 ~ -0.99) |
| Niue | 1.09(0.9~1.32) | 0.68(0.55~0.81) | -0.38 | 45.17(37.08~54.12) | 43.68(36.13~52.12) | -0.21 (-0.24 ~ -0.17) |
| North Macedonia | 602.73(496.92~718.16) | 667.75(533.13~814.5) | 0.11 | 32.24(26.67~38.3) | 31.15(25.58~36.82) | -0.18 (-0.2 ~ -0.15) |
| Northern Mariana Islands | 19.02(15.3~23.12) | 20.03(16.23~24.07) | 0.05 | 46.03(38.01~55.1) | 47.85(39.62~56.96) | 0.12 (0.09 ~ 0.15) |
| Norway | 1008.53(797.54~1231.43) | 1172.99(920.06~1464.98) | 0.16 | 20.85(16.87~25.13) | 17.14(13.85~20.63) | -0.22 (-0.4 ~ -0.04) |
| Oman | 1379.15(1107.7~1668.55) | 2509.04(2005.37~3097.37) | 0.82 | 76.25(62.15~91.39) | 60.83(49.37~72.85) | -0.7 (-0.73 ~ -0.67) |
| Pakistan | 128585.92(106981.89~153783.56) | 203629.76(163727.48~246211.03) | 0.58 | 103.38(85.58~123.5) | 87.09(69.03~105.54) | -0.13 (-0.46 ~ 0.19) |
| Palau | 6.5(5.28~7.87) | 7.32(5.94~8.82) | 0.13 | 45.66(37.33~54.64) | 49.64(41.23~58.94) | 0.23 (0.21 ~ 0.25) |
| Palestine | 1537.95(1247.1~1856.89) | 3057.29(2526.19~3656.67) | 0.99 | 82.92(68.3~99.82) | 70.71(57.75~84) | -0.47 (-0.51 ~ -0.44) |
| Panama | 1188.69(970.12~1423.05) | 1774.22(1469.68~2103.91) | 0.49 | 49.51(40.54~58.75) | 42.69(35.38~50.36) | -0.4 (-0.44 ~ -0.36) |
| Papua New Guinea | 3604.66(2961.74~4376.01) | 8452.92(6919.04~10155.33) | 1.34 | 68.14(57.02~81.13) | 67.08(55.3~79.8) | -0.19 (-0.26 ~ -0.13) |
| Paraguay | 2693.59(2200~3250.58) | 3992.46(3316.2~4712.76) | 0.48 | 63.8(52.28~75.87) | 58.95(48.83~69.35) | -0.2 (-0.24 ~ -0.17) |
| Peru | 8032.18(6664.13~9564.07) | 11142.83(9137.3~13191.73) | 0.39 | 37.76(31.29~44.81) | 31.84(26.21~37.87) | -0.58 (-0.68 ~ -0.48) |
| Philippines | 49896.16(40187.97~60798.81) | 64270.11(52053.92~77129.85) | 0.29 | 68.71(56.43~82.09) | 58.87(48.2~70.6) | -0.46 (-0.51 ~ -0.41) |
| Poland | 13501.08(10951.58~16384.38) | 11362.75(9061.01~13799.7) | -0.16 | 36.37(29.75~43.48) | 29.15(23.85~34.8) | -0.56 (-0.71 ~ -0.41) |
| Portugal | 4001.62(3241.17~4888.4) | 5067.16(4011~6286.95) | 0.27 | 38.48(31.5~46.16) | 40.41(33.19~48.49) | 0.32 (0.24 ~ 0.4) |
| Puerto Rico | 2028.44(1674.97~2389.83) | 1486.88(1188.8~1824.65) | -0.27 | 57.84(47.74~68.18) | 42.27(34.81~50.34) | -1.14 (-1.21 ~ -1.07) |
| Qatar | 278.82(225.11~340.37) | 1572.09(1195.53~1950.56) | 4.64 | 73.2(60.69~87.21) | 63.02(52.14~75.69) | -0.62 (-0.71 ~ -0.53) |
| Republic of Korea | 18191.7(14913.55~21615.3) | 29530.21(22969.03~36201.2) | 0.62 | 48.6(40.43~58.18) | 40.06(32.15~48.18) | -1.17 (-1.48 ~ -0.85) |
| Republic of Moldova | 3706.16(3047.26~4396.89) | 2454.66(1992.27~2962.63) | -0.34 | 85.29(70.08~101.06) | 80.89(66.78~95.86) | -0.04 (-0.22 ~ 0.14) |
| Romania | 9858.47(8015.7~11794.24) | 6288.09(4818.44~7800.37) | -0.36 | 45.77(37.59~54.55) | 34.99(27.95~42.33) | -0.63 (-0.86 ~ -0.4) |
| Russian Federation | 73319.99(59743.05~87771.17) | 80469.08(65653.81~98048.76) | 0.1 | 52.28(42.67~62.27) | 62.59(51.1~75.03) | 0.74 (0.55 ~ 0.93) |
| Rwanda | 8889.81(7313.36~10544.64) | 8900.08(6943.24~11012.79) | 0 | 127.87(104.64~152.27) | 66.8(51.33~83.91) | -1.82 (-2.08 ~ -1.57) |
| Saint Kitts and Nevis | 26.94(22.57~31.91) | 25.26(20.35~30.27) | -0.06 | 65.54(54.82~78.1) | 45.66(37.51~54.08) | -1.26 (-1.41 ~ -1.12) |
| Saint Lucia | 86.71(70.86~104.13) | 79.21(62.29~96.1) | -0.09 | 63.35(52.29~75.33) | 47.3(38.55~56.26) | -1 (-1.14 ~ -0.86) |
| Saint Vincent and the Grenadines | 62.94(51.66~75.28) | 50.31(40.5~60.52) | -0.2 | 58.74(49.12~69.64) | 46.53(38.46~55.13) | -0.75 (-0.86 ~ -0.64) |
| Samoa | 105.3(85.54~126.89) | 147.18(120.45~176.3) | 0.4 | 53(43.37~63.59) | 60.38(50.14~71.99) | 0.37 (0.27 ~ 0.47) |
| San Marino | 7.06(5.67~8.66) | 12.01(9.52~15.02) | 0.7 | 26.68(21.62~31.91) | 26.97(21.97~32.5) | 0.03 (0 ~ 0.07) |
| Sao Tome and Principe | 158.86(128.47~194.69) | 192.72(158.18~230.33) | 0.21 | 113.32(93.16~135.81) | 92.38(76.3~110.14) | -0.75 (-0.8 ~ -0.69) |
| Saudi Arabia | 17884.56(14901.42~21347.34) | 36553.24(28911.89~44631.87) | 1.04 | 129.05(106.64~154.97) | 105.47(86.86~125.43) | -0.54 (-0.59 ~ -0.48) |
| Senegal | 6546.54(5347.9~7886.72) | 11798.57(9654.5~13971.83) | 0.8 | 78.01(64.26~92.5) | 74.62(61.6~88.3) | -0.15 (-0.18 ~ -0.11) |
| Serbia | 2951.62(2377.11~3547.75) | 2907.3(2367.69~3511.25) | -0.02 | 32.1(26.15~38.19) | 30.61(25.42~36.18) | -0.26 (-0.33 ~ -0.18) |
| Seychelles | 52.38(42.88~62.57) | 59.58(49.02~70.78) | 0.14 | 69.64(57.1~83.36) | 63.36(52.01~74.84) | -0.25 (-0.27 ~ -0.22) |
| Sierra Leone | 6207.63(5073.44~7494.6) | 9312.35(7525.55~11171.5) | 0.5 | 121.37(100.23~143.85) | 98.13(80.3~116.25) | -0.78 (-0.85 ~ -0.72) |
| Singapore | 1337.31(1060.37~1623.17) | 3831.12(2989.72~4726.64) | 1.86 | 50.79(41.02~60.71) | 53.72(43.73~64.89) | 0.1 (0.06 ~ 0.13) |
| Slovakia | 1774.71(1469.33~2115.78) | 1684.66(1362.15~2039.09) | -0.05 | 35.25(29.12~41.87) | 30.83(25.47~36.51) | -0.23 (-0.32 ~ -0.14) |
| Slovenia | 624.95(509.93~746.1) | 646.04(518.67~786.2) | 0.03 | 33.79(28.12~40.27) | 27.19(22.71~32.14) | -0.68 (-0.72 ~ -0.63) |
| Solomon Islands | 245.29(201.27~297.49) | 526.9(429.99~630.48) | 1.15 | 56.42(46.26~67.1) | 67.43(55.71~79.34) | 0.64 (0.58 ~ 0.7) |
| Somalia | 9971.83(8028.12~12061.55) | 24525.08(19639.12~29452.54) | 1.46 | 122.59(100.18~146.74) | 115.6(94.86~137.56) | -0.22 (-0.26 ~ -0.18) |
| South Africa | 20732.95(16781.58~25024.77) | 31424.61(25443.3~38108.32) | 0.52 | 59.95(48.55~72.18) | 59.13(47.97~71.76) | -0.12 (-0.27 ~ 0.04) |
| South Sudan | 5955.23(4865.75~7163.17) | 8653.05(7120.28~10293.52) | 0.45 | 105.55(86.04~126.19) | 96.04(78.83~114.34) | -0.32 (-0.34 ~ -0.31) |
| Spain | 17237.83(14287.27~20792.45) | 20186.4(15874.45~24638.64) | 0.17 | 42.45(35.94~50.12) | 33.67(27.68~39.59) | -0.76 (-0.79 ~ -0.74) |
| Sri Lanka | 11721.68(9657.84~14006.42) | 11983.92(9852.32~14270.75) | 0.02 | 69.44(57.12~82.86) | 60.66(50.23~71.99) | -0.62 (-0.74 ~ -0.5) |
| Sudan | 17577.18(14335.64~21204.19) | 28329.58(23204.64~34016.77) | 0.61 | 91.76(74.89~109.7) | 75.49(61.97~90.54) | -0.7 (-0.72 ~ -0.68) |
| Suriname | 235.79(194.88~281.87) | 293.48(237.94~351.99) | 0.24 | 62.32(51.41~74.02) | 52.66(42.96~63.18) | -0.62 (-0.68 ~ -0.57) |
| Sweden | 1870.3(1479.08~2290.89) | 2510.25(1976.29~3162.8) | 0.34 | 17.86(14.56~21.59) | 18.19(14.65~22.05) | 0.12 (0.08 ~ 0.16) |
| Switzerland | 2155.26(1737.18~2634.26) | 3318.44(2644.69~4057.38) | 0.54 | 28.66(23.27~34.14) | 29.88(24.43~35.48) | 0 (-0.08 ~ 0.08) |
| Syrian Arab Republic | 9122.51(7394.24~10960.42) | 9316.24(7411.12~11241.06) | 0.02 | 80.78(65.78~97.16) | 71.42(58.38~85.03) | -0.33 (-0.39 ~ -0.26) |
| Taiwan (Province of China) | 12836.78(11012.2~14799.74) | 10964.5(8978.37~13073.49) | -0.15 | 74.55(63.75~86.29) | 68.99(57.3~81.54) | -0.59 (-0.79 ~ -0.38) |
| Tajikistan | 12531.6(10301.82~15058.82) | 18560.14(15212.42~21986.85) | 0.48 | 159.37(131.35~188.66) | 155.8(127.06~183.73) | -0.14 (-0.2 ~ -0.08) |
| Thailand | 38290.52(31974.98~45392.58) | 32655.03(26114.34~39493.12) | -0.15 | 71.8(60.28~84.73) | 58.57(48.11~69.1) | -1 (-1.21 ~ -0.78) |
| Timor-Leste | 894.19(731.16~1088.67) | 1067.27(870.6~1281.79) | 0.19 | 83.93(69.44~100.4) | 69.08(56.64~82.14) | -0.73 (-0.81 ~ -0.65) |
| Togo | 5023.61(4023.77~6091.08) | 8253.28(6675.07~9899.29) | 0.64 | 115.74(95.39~137.92) | 95.25(78.14~112.54) | -0.69 (-0.72 ~ -0.65) |
| Tokelau | 0.87(0.71~1.05) | 0.59(0.49~0.71) | -0.32 | 50.41(41.18~60.37) | 47.78(39.6~56.9) | -0.25 (-0.29 ~ -0.21) |
| Tonga | 69.73(56.84~84.55) | 75.69(62.57~89.24) | 0.09 | 60.07(49.56~71.67) | 64.1(53.04~76.29) | 0.15 (0.12 ~ 0.18) |
| Trinidad and Tobago | 634.44(518.3~754.23) | 571.07(456.87~687.89) | -0.1 | 54.28(44.6~64.85) | 42.41(34.68~50.27) | -0.85 (-0.95 ~ -0.74) |
| Tunisia | 4875.01(4040.61~5845.93) | 7894.39(6342.59~9425.23) | 0.62 | 66.44(55.08~78.68) | 65.49(53.32~77.54) | 0.39 (0.21 ~ 0.57) |
| Turkmenistan | 7354.22(6025.42~8787.75) | 8378.61(6903.21~9935.38) | 0.14 | 149.86(123.6~177.33) | 160.04(132.22~189.04) | 0.28 (0.23 ~ 0.33) |
| Tuvalu | 6.32(5.19~7.6) | 7.36(6.04~8.69) | 0.16 | 55.12(45.89~66.01) | 59.97(49.65~70.69) | 0.29 (0.26 ~ 0.31) |
| T眉rkiye | 22392.84(18401.08~26587.44) | 33079.37(26478.82~39774.31) | 0.48 | 44.91(37.15~53.65) | 39.08(31.73~46.58) | -0.51 (-0.93 ~ -0.09) |
| Uganda | 23997.08(19378.55~29018.8) | 41569.04(33489.45~49674.16) | 0.73 | 129.07(105.85~155.53) | 103.92(85.51~124.19) | -0.79 (-0.85 ~ -0.73) |
| Ukraine | 32294.47(26077.37~38945.02) | 28752.06(23326.74~35076.65) | -0.11 | 67.81(55.1~81.75) | 85.68(69.42~102.82) | 1.01 (0.77 ~ 1.24) |
| United Arab Emirates | 979.31(779.64~1205.24) | 5050.7(3841.66~6366.78) | 4.16 | 60.49(49.91~72.22) | 53.35(43.58~63.24) | -0.4 (-0.48 ~ -0.32) |
| United Kingdom | 20237.43(16344.49~24575.84) | 23445.32(18366.16~29174.27) | 0.16 | 32.89(26.96~39.34) | 29.45(23.54~35.63) | 0.39 (-0.3 ~ 1.08) |
| United Republic of Tanzania | 34229.65(27367.21~41130.18) | 67996.7(55623.83~81114.73) | 0.99 | 126.9(103.36~150.64) | 121.13(99.21~143.17) | -0.27 (-0.42 ~ -0.12) |
| United States Virgin Islands | 55.59(45.41~66.72) | 41.24(32.87~50.95) | -0.26 | 53.57(44~64.05) | 44.19(35.93~52.29) | -0.67 (-0.73 ~ -0.62) |
| United States of America | 85054.79(68674.27~103615.44) | 118781.02(95472.33~143522.92) | 0.4 | 32.88(26.87~39.73) | 32.75(26.73~38.96) | -0.14 (-0.22 ~ -0.06) |
| Uruguay | 909.22(746.32~1093.61) | 1103.45(891.67~1320.49) | 0.21 | 27.17(22.34~32.25) | 27.03(22.08~32.34) | -0.06 (-0.13 ~ 0.02) |
| Uzbekistan | 39294.79(32517.11~46521.57) | 55662.43(46239.76~65692.28) | 0.42 | 142.3(118.16~165.69) | 156.53(130.47~184.71) | 0.3 (0.17 ~ 0.43) |
| Vanuatu | 125.2(100.14~153.69) | 239.59(195.64~285.91) | 0.91 | 63.11(52.31~75.65) | 68.37(56.41~81.74) | 0.22 (0.2 ~ 0.24) |
| Venezuela (Bolivarian Republic of) | 10539.01(8733.75~12578.44) | 11954.03(9808.78~14254.96) | 0.13 | 54.06(44.86~64) | 46.08(37.67~54.84) | -0.77 (-0.88 ~ -0.66) |
| Viet Nam | 67026.84(54687.24~79845) | 58786.81(48469.49~69255.17) | -0.12 | 85.23(70.38~100.91) | 64.45(53.77~76.15) | -0.83 (-1.09 ~ -0.57) |
| Yemen | 15857.9(13041.32~19034.03) | 27625.57(22373.35~33211.53) | 0.74 | 117.97(97.29~140.78) | 92.54(75.66~110.8) | -0.67 (-0.83 ~ -0.5) |
| Zambia | 8818.78(7168.06~10471.09) | 16160.78(13183.14~19182.33) | 0.83 | 115.36(94.89~136.6) | 96.68(79.11~114.71) | -0.7 (-0.77 ~ -0.63) |
| Zimbabwe | 7781.13(6329.98~9340.68) | 12604.89(10365.58~14972.97) | 0.62 | 80.87(66.73~96.36) | 88.98(73.29~105.84) | 0.36 (0.3 ~ 0.42) |
