# Supplementary material for: Global, regional, and national burden of chronic hepatitis C including cirrhosis from 1990 to 2021, alongside projections for the next 15 years
Source: Medicine (Baltimore). 2026 Oct 16;105(42):e51118. doi: 10.1097/MD.0000000000051118 (PMC13681144; doi:10.1097/MD.0000000000051118)
Supplement: Supplementary file 5 [file medi-105-e51118-s005.docx]

Supplementary Table S8. Joinpoint regression analysis on age-standardized incidence rates for chronic hepatitis C including cirrhosis at global level and 5 SDI regions from 1990 to 2021.

| location | Model | Segment | Segment Start | Segment End | APC | APC 95% LCL | APC 95% UCL | APC Significant | Test Statistic | P-Value | location | Start Obs | End Obs | AAPC | AAPC C.I. Low | AAPC C.I. High | Statistically Significant (0=No 1=Yes) | Test Statistic | P-Value |
| --- | --- | --- | --- | --- | --- | --- | --- | --- | --- | --- | --- | --- | --- | --- | --- | --- | --- | --- | --- |
| Global | 4 | 0 | 1990 | 1995 | -0.2857 | -0.4119 | -0.1593 | 1 | -4.7451 | 0.000162 | Global | 1990 | 2021 | -0.4797 | -0.5261 | -0.4334 | 1 | -20.2418 | 0 |
| Global | 4 | 1 | 1995 | 2000 | -1.2039 | -1.3774 | -1.03 | 1 | -14.4729 | 0 |  |  |  |  |  |  |  |  |  |
| Global | 4 | 2 | 2000 | 2006 | 0.0017 | -0.1232 | 0.1267 | 0 | 0.0283 | 0.977774 |  |  |  |  |  |  |  |  |  |
| Global | 4 | 3 | 2006 | 2015 | -0.7021 | -0.7634 | -0.6406 | 1 | -23.9326 | 0 |  |  |  |  |  |  |  |  |  |
| Global | 4 | 4 | 2015 | 2021 | -0.1816 | -0.2797 | -0.0833 | 1 | -3.8818 | 0.001093 |  |  |  |  |  |  |  |  |  |
| High SDI | 5 | 0 | 1990 | 1996 | -0.1546 | -0.2239 | -0.0852 | 1 | -4.7481 | 0.000259 | High SDI | 1990 | 2021 | -0.5837 | -0.6337 | -0.5337 | 1 | -22.821 | 0 |
| High SDI | 5 | 1 | 1996 | 2000 | -0.9357 | -1.1403 | -0.7307 | 1 | -9.6924 | 0 |  |  |  |  |  |  |  |  |  |
| High SDI | 5 | 2 | 2000 | 2005 | -2.1532 | -2.2819 | -2.0244 | 1 | -35.2603 | 0 |  |  |  |  |  |  |  |  |  |
| High SDI | 5 | 3 | 2005 | 2015 | -0.4388 | -0.4768 | -0.4008 | 1 | -24.5881 | 0 |  |  |  |  |  |  |  |  |  |
| High SDI | 5 | 4 | 2015 | 2019 | 0.5572 | 0.3471 | 0.7677 | 1 | 5.6635 | 0.000045 |  |  |  |  |  |  |  |  |  |
| High SDI | 5 | 5 | 2019 | 2021 | -0.1999 | -0.6276 | 0.2295 | 0 | -0.9934 | 0.336277 |  |  |  |  |  |  |  |  |  |
| High-middle SDI | 5 | 0 | 1990 | 1995 | -0.0455 | -0.1944 | 0.1036 | 0 | -0.6511 | 0.524851 | High-middle SDI | 1990 | 2021 | -0.8913 | -0.9596 | -0.823 | 1 | -25.4638 | 0 |
| High-middle SDI | 5 | 1 | 1995 | 2000 | -2.8442 | -3.0456 | -2.6424 | 1 | -29.6402 | 0 |  |  |  |  |  |  |  |  |  |
| High-middle SDI | 5 | 2 | 2000 | 2005 | -0.6264 | -0.8346 | -0.4178 | 1 | -6.3873 | 0.000012 |  |  |  |  |  |  |  |  |  |
| High-middle SDI | 5 | 3 | 2005 | 2010 | -1.6253 | -1.8315 | -1.4187 | 1 | -16.645 | 0 |  |  |  |  |  |  |  |  |  |
| High-middle SDI | 5 | 4 | 2010 | 2015 | -0.3288 | -0.5371 | -0.12 | 1 | -3.3544 | 0.004346 |  |  |  |  |  |  |  |  |  |
| High-middle SDI | 5 | 5 | 2015 | 2021 | -0.0197 | -0.134 | 0.0947 | 0 | -0.3667 | 0.718968 |  |  |  |  |  |  |  |  |  |
| Low SDI | 5 | 0 | 1990 | 1995 | -0.1833 | -0.2261 | -0.1405 | 1 | -9.1189 | 0 | Low SDI | 1990 | 2021 | -0.4496 | -0.4706 | -0.4287 | 1 | -41.9961 | 0 |
| Low SDI | 5 | 1 | 1995 | 2002 | -0.0029 | -0.0349 | 0.029 | 0 | -0.1953 | 0.847769 |  |  |  |  |  |  |  |  |  |
| Low SDI | 5 | 2 | 2002 | 2006 | -0.3906 | -0.4836 | -0.2975 | 1 | -8.9319 | 0 |  |  |  |  |  |  |  |  |  |
| Low SDI | 5 | 3 | 2006 | 2014 | -0.9583 | -0.9831 | -0.9334 | 1 | -81.7448 | 0 |  |  |  |  |  |  |  |  |  |
| Low SDI | 5 | 4 | 2014 | 2019 | -0.6488 | -0.7075 | -0.5901 | 1 | -23.4891 | 0 |  |  |  |  |  |  |  |  |  |
| Low SDI | 5 | 5 | 2019 | 2021 | -0.2542 | -0.4405 | -0.0677 | 1 | -2.9038 | 0.010909 |  |  |  |  |  |  |  |  |  |
| Low-middle SDI | 4 | 0 | 1990 | 1995 | -0.7426 | -0.9144 | -0.5706 | 1 | -9.0436 | 0 | Low-middle SDI | 1990 | 2021 | -0.6432 | -0.7133 | -0.5731 | 1 | -17.9302 | 0 |
| Low-middle SDI | 4 | 1 | 1995 | 2000 | -1.2375 | -1.4757 | -0.9988 | 1 | -10.836 | 0 |  |  |  |  |  |  |  |  |  |
| Low-middle SDI | 4 | 2 | 2000 | 2005 | 0.7328 | 0.4881 | 0.9782 | 1 | 6.3057 | 0.000006 |  |  |  |  |  |  |  |  |  |
| Low-middle SDI | 4 | 3 | 2005 | 2010 | -0.2507 | -0.4908 | -0.0101 | 1 | -2.1887 | 0.042043 |  |  |  |  |  |  |  |  |  |
| Low-middle SDI | 4 | 4 | 2010 | 2021 | -1.1251 | -1.1786 | -1.0715 | 1 | -43.8603 | 0 |  |  |  |  |  |  |  |  |  |
| Middle SDI | 4 | 0 | 1990 | 1995 | -0.5865 | -0.7431 | -0.4297 | 1 | -7.8427 | 0 | Middle SDI | 1990 | 2021 | -0.6739 | -0.7334 | -0.6143 | 1 | -22.1056 | 0 |
| Middle SDI | 4 | 1 | 1995 | 2000 | -2.2988 | -2.514 | -2.0831 | 1 | -22.1574 | 0 |  |  |  |  |  |  |  |  |  |
| Middle SDI | 4 | 2 | 2000 | 2005 | -0.0599 | -0.2809 | 0.1617 | 0 | -0.5681 | 0.577 |  |  |  |  |  |  |  |  |  |
| Middle SDI | 4 | 3 | 2005 | 2014 | -0.7867 | -0.8624 | -0.7109 | 1 | -21.7271 | 0 |  |  |  |  |  |  |  |  |  |
| Middle SDI | 4 | 4 | 2014 | 2021 | 0.1451 | 0.0508 | 0.2394 | 1 | 3.2344 | 0.004601 |  |  |  |  |  |  |  |  |  |
