# Supplementary material for: Global, regional, and national burden of chronic hepatitis C including cirrhosis from 1990 to 2021, alongside projections for the next 15 years
Source: Medicine (Baltimore). 2026 Oct 16;105(42):e51118. doi: 10.1097/MD.0000000000051118 (PMC13681144; doi:10.1097/MD.0000000000051118)
Supplement: Supplementary file 6 [file medi-105-e51118-s006.docx]

Supplementary Table S12. Frontier analysis based on SDI and age-standardized incidence rate of chronic hepatitis C including cirrhosis at 204 countries and territories.

| location | The age-standardized incidence rate/100000 in 2021 | SDI in 2021 | frontier | effective difference | trend |
| --- | --- | --- | --- | --- | --- |
| Afghanistan | 93.55 | 0.34 | 30.62 | 62.93 | Decrease |
| Albania | 30.80 | 0.71 | 19.44 | 11.36 | Decrease |
| Algeria | 63.78 | 0.66 | 19.48 | 44.30 | Decrease |
| American Samoa | 40.16 | 0.72 | 19.50 | 20.66 | Increase |
| Andorra | 34.10 | 0.87 | 16.33 | 17.77 | Decrease |
| Angola | 147.26 | 0.45 | 30.50 | 116.76 | Decrease |
| Antigua and Barbuda | 43.83 | 0.75 | 19.44 | 24.39 | Decrease |
| Argentina | 25.70 | 0.72 | 19.42 | 6.28 | Decrease |
| Armenia | 117.70 | 0.70 | 19.48 | 98.22 | Increase |
| Australia | 46.12 | 0.84 | 17.15 | 28.97 | Decrease |
| Austria | 26.98 | 0.85 | 17.07 | 9.92 | Decrease |
| Azerbaijan | 147.56 | 0.69 | 19.45 | 128.11 | Decrease |
| Bahamas | 44.17 | 0.81 | 17.75 | 26.42 | Decrease |
| Bahrain | 62.32 | 0.75 | 19.46 | 42.86 | Decrease |
| Bangladesh | 34.98 | 0.49 | 30.46 | 4.52 | Decrease |
| Barbados | 41.54 | 0.75 | 19.43 | 22.11 | Decrease |
| Belarus | 60.52 | 0.78 | 19.47 | 41.05 | Increase |
| Belgium | 29.28 | 0.85 | 16.93 | 12.35 | Increase |
| Belize | 54.86 | 0.61 | 20.68 | 34.18 | Decrease |
| Benin | 100.59 | 0.37 | 30.50 | 70.10 | Decrease |
| Bermuda | 35.35 | 0.82 | 17.72 | 17.62 | Decrease |
| Bhutan | 76.40 | 0.47 | 30.47 | 45.92 | Increase |
| Bolivia (Plurinational State of) | 38.38 | 0.60 | 20.67 | 17.71 | Decrease |
| Bosnia and Herzegovina | 20.59 | 0.72 | 19.44 | 1.15 | Decrease |
| Botswana | 71.65 | 0.64 | 19.89 | 51.76 | Decrease |
| Brazil | 62.93 | 0.65 | 19.74 | 43.19 | Decrease |
| Brunei Darussalam | 56.02 | 0.81 | 17.73 | 38.30 | Increase |
| Bulgaria | 30.02 | 0.77 | 19.41 | 10.62 | Decrease |
| Burkina Faso | 128.51 | 0.29 | 34.39 | 94.12 | Decrease |
| Burundi | 133.49 | 0.29 | 34.45 | 99.04 | Decrease |
| Cabo Verde | 83.65 | 0.53 | 30.67 | 52.98 | Decrease |
| Cambodia | 123.41 | 0.47 | 30.49 | 92.92 | Decrease |
| Cameroon | 107.48 | 0.48 | 30.66 | 76.82 | Decrease |
| Canada | 21.84 | 0.87 | 15.88 | 5.96 | Decrease |
| Central African Republic | 135.28 | 0.31 | 31.08 | 104.21 | Decrease |
| Chad | 126.60 | 0.24 | 36.72 | 89.89 | Decrease |
| Chile | 38.58 | 0.77 | 19.47 | 19.12 | Decrease |
| China | 35.85 | 0.72 | 19.42 | 16.44 | Decrease |
| Colombia | 44.72 | 0.66 | 19.44 | 25.29 | Decrease |
| Comoros | 83.24 | 0.48 | 30.64 | 52.61 | Decrease |
| Congo | 133.80 | 0.58 | 20.67 | 113.13 | Decrease |
| Cook Islands | 36.88 | 0.78 | 19.45 | 17.43 | Decrease |
| Costa Rica | 49.62 | 0.70 | 19.42 | 30.20 | Decrease |
| Croatia | 27.41 | 0.80 | 17.76 | 9.65 | Decrease |
| Cuba | 43.55 | 0.67 | 19.41 | 24.15 | Decrease |
| Cyprus | 24.85 | 0.84 | 17.37 | 7.48 | Decrease |
| Czechia | 31.01 | 0.83 | 17.49 | 13.52 | Decrease |
| C么te d'Ivoire | 92.20 | 0.43 | 30.47 | 61.73 | Decrease |
| Democratic People's Republic of Korea | 57.82 | 0.57 | 20.67 | 37.14 | Increase |
| Democratic Republic of the Congo | 149.24 | 0.38 | 30.50 | 118.74 | Decrease |
| Denmark | 26.07 | 0.90 | 14.94 | 11.14 | Decrease |
| Djibouti | 82.99 | 0.49 | 30.48 | 52.51 | Decrease |
| Dominica | 43.34 | 0.75 | 19.46 | 23.88 | Decrease |
| Dominican Republic | 57.10 | 0.62 | 20.64 | 36.46 | Decrease |
| Ecuador | 33.45 | 0.66 | 19.41 | 14.04 | Decrease |
| Egypt | 147.55 | 0.61 | 20.67 | 126.88 | Decrease |
| El Salvador | 57.42 | 0.56 | 20.68 | 36.74 | Decrease |
| Equatorial Guinea | 112.21 | 0.66 | 19.82 | 92.39 | Decrease |
| Eritrea | 99.55 | 0.40 | 30.64 | 68.91 | Decrease |
| Estonia | 57.41 | 0.84 | 17.14 | 40.27 | Increase |
| Eswatini | 76.88 | 0.59 | 20.68 | 56.20 | Increase |
| Ethiopia | 93.35 | 0.36 | 30.50 | 62.85 | Decrease |
| Fiji | 41.87 | 0.68 | 19.41 | 22.46 | Decrease |
| Finland | 28.16 | 0.86 | 16.81 | 11.35 | Increase |
| France | 23.47 | 0.84 | 17.26 | 6.20 | Decrease |
| Gabon | 152.74 | 0.63 | 20.14 | 132.60 | Decrease |
| Gambia | 79.27 | 0.41 | 30.48 | 48.79 | Decrease |
| Georgia | 97.12 | 0.73 | 19.47 | 77.65 | Decrease |
| Germany | 27.52 | 0.90 | 13.42 | 14.10 | Decrease |
| Ghana | 111.18 | 0.56 | 20.68 | 90.51 | Decrease |
| Greece | 21.05 | 0.79 | 17.80 | 3.25 | Decrease |
| Greenland | 30.61 | 0.83 | 17.51 | 13.09 | Decrease |
| Grenada | 47.16 | 0.67 | 19.42 | 27.74 | Decrease |
| Guam | 40.12 | 0.80 | 17.80 | 22.32 | Increase |
| Guatemala | 75.22 | 0.54 | 20.72 | 54.50 | Decrease |
| Guinea | 108.21 | 0.34 | 30.50 | 77.70 | Decrease |
| Guinea-Bissau | 83.46 | 0.35 | 30.47 | 52.99 | Decrease |
| Guyana | 61.00 | 0.65 | 19.51 | 41.49 | Decrease |
| Haiti | 73.73 | 0.45 | 30.47 | 43.26 | Decrease |
| Honduras | 70.79 | 0.51 | 30.51 | 40.29 | Decrease |
| Hungary | 28.24 | 0.79 | 17.97 | 10.27 | Decrease |
| Iceland | 13.42 | 0.88 | 13.42 | 0.00 | Decrease |
| India | 45.00 | 0.58 | 20.67 | 24.33 | Decrease |
| Indonesia | 78.88 | 0.66 | 19.93 | 58.95 | Decrease |
| Iran (Islamic Republic of) | 39.69 | 0.70 | 19.48 | 20.20 | Decrease |
| Iraq | 83.34 | 0.66 | 19.42 | 63.93 | Decrease |
| Ireland | 24.58 | 0.87 | 16.03 | 8.55 | Decrease |
| Israel | 24.60 | 0.81 | 17.72 | 6.88 | Decrease |
| Italy | 51.71 | 0.81 | 17.74 | 33.97 | Decrease |
| Jamaica | 41.67 | 0.68 | 19.44 | 22.23 | Decrease |
| Japan | 41.72 | 0.87 | 16.32 | 25.39 | Decrease |
| Jordan | 65.43 | 0.73 | 19.41 | 46.02 | Decrease |
| Kazakhstan | 85.86 | 0.73 | 19.43 | 66.43 | Increase |
| Kenya | 87.70 | 0.52 | 30.46 | 57.24 | Decrease |
| Kiribati | 69.57 | 0.53 | 30.47 | 39.10 | Increase |
| Kuwait | 49.93 | 0.85 | 17.03 | 32.90 | Decrease |
| Kyrgyzstan | 152.37 | 0.60 | 20.67 | 131.70 | Increase |
| Lao People's Democratic Republic | 69.36 | 0.49 | 30.48 | 38.88 | Decrease |
| Latvia | 47.57 | 0.83 | 17.53 | 30.04 | Decrease |
| Lebanon | 43.87 | 0.74 | 19.46 | 24.41 | Decrease |
| Lesotho | 82.40 | 0.51 | 30.47 | 51.93 | Increase |
| Liberia | 106.19 | 0.35 | 30.46 | 75.72 | Decrease |
| Libya | 58.60 | 0.73 | 19.41 | 39.18 | Decrease |
| Lithuania | 63.17 | 0.86 | 17.00 | 46.16 | Increase |
| Luxembourg | 27.48 | 0.88 | 14.97 | 12.51 | Decrease |
| Madagascar | 69.92 | 0.40 | 30.47 | 39.45 | Decrease |
| Malawi | 97.95 | 0.38 | 30.48 | 67.47 | Decrease |
| Malaysia | 76.16 | 0.74 | 19.49 | 56.67 | Decrease |
| Maldives | 74.69 | 0.65 | 19.51 | 55.17 | Decrease |
| Mali | 102.55 | 0.27 | 35.43 | 67.12 | Decrease |
| Malta | 24.85 | 0.80 | 17.81 | 7.04 | Decrease |
| Marshall Islands | 64.18 | 0.57 | 20.67 | 43.50 | Increase |
| Mauritania | 85.40 | 0.50 | 30.63 | 54.78 | Decrease |
| Mauritius | 81.12 | 0.72 | 19.48 | 61.64 | Decrease |
| Mexico | 47.63 | 0.66 | 19.42 | 28.21 | Decrease |
| Micronesia (Federated States of) | 62.06 | 0.59 | 20.67 | 41.38 | Increase |
| Monaco | 29.71 | 0.91 | 13.81 | 15.90 | Increase |
| Mongolia | 239.82 | 0.62 | 20.65 | 219.17 | Decrease |
| Montenegro | 28.64 | 0.80 | 17.76 | 10.87 | Decrease |
| Morocco | 67.55 | 0.56 | 20.69 | 46.86 | Decrease |
| Mozambique | 82.21 | 0.33 | 30.63 | 51.58 | Decrease |
| Myanmar | 86.72 | 0.53 | 28.39 | 58.34 | Decrease |
| Namibia | 68.55 | 0.62 | 20.64 | 47.91 | Decrease |
| Nauru | 58.34 | 0.63 | 20.59 | 37.75 | Increase |
| Nepal | 35.65 | 0.43 | 30.51 | 5.15 | Decrease |
| Netherlands | 18.95 | 0.89 | 13.50 | 5.44 | Decrease |
| New Zealand | 38.51 | 0.85 | 17.09 | 21.41 | Increase |
| Nicaragua | 75.87 | 0.52 | 30.50 | 45.38 | Decrease |
| Niger | 115.93 | 0.17 | 113.52 | 2.42 | Decrease |
| Nigeria | 113.42 | 0.50 | 30.60 | 82.82 | Decrease |
| Niue | 43.68 | 0.73 | 19.43 | 24.25 | Decrease |
| North Macedonia | 31.15 | 0.75 | 19.45 | 11.69 | Decrease |
| Northern Mariana Islands | 47.85 | 0.77 | 19.47 | 28.38 | Increase |
| Norway | 17.14 | 0.92 | 13.42 | 3.73 | Decrease |
| Oman | 60.83 | 0.77 | 19.42 | 41.41 | Decrease |
| Pakistan | 87.09 | 0.50 | 30.47 | 56.62 | Decrease |
| Palau | 49.64 | 0.75 | 19.50 | 30.14 | Increase |
| Palestine | 70.71 | 0.63 | 20.46 | 50.25 | Decrease |
| Panama | 42.69 | 0.71 | 19.45 | 23.24 | Decrease |
| Papua New Guinea | 67.08 | 0.42 | 30.46 | 36.61 | Decrease |
| Paraguay | 58.95 | 0.64 | 20.16 | 38.79 | Decrease |
| Peru | 31.84 | 0.66 | 19.41 | 12.43 | Decrease |
| Philippines | 58.87 | 0.65 | 19.51 | 39.36 | Decrease |
| Poland | 29.15 | 0.81 | 17.72 | 11.43 | Decrease |
| Portugal | 40.41 | 0.74 | 19.47 | 20.93 | Increase |
| Puerto Rico | 42.27 | 0.83 | 17.72 | 24.55 | Decrease |
| Qatar | 63.02 | 0.85 | 17.04 | 45.98 | Decrease |
| Republic of Korea | 40.06 | 0.89 | 14.07 | 25.99 | Decrease |
| Republic of Moldova | 80.89 | 0.73 | 19.43 | 61.46 | Decrease |
| Romania | 34.99 | 0.77 | 19.49 | 15.51 | Decrease |
| Russian Federation | 62.59 | 0.81 | 17.73 | 44.86 | Increase |
| Rwanda | 66.80 | 0.44 | 30.51 | 36.29 | Decrease |
| Saint Kitts and Nevis | 45.66 | 0.75 | 19.41 | 26.25 | Decrease |
| Saint Lucia | 47.30 | 0.67 | 19.43 | 27.87 | Decrease |
| Saint Vincent and the Grenadines | 46.53 | 0.64 | 20.22 | 26.31 | Decrease |
| Samoa | 60.38 | 0.59 | 20.67 | 39.71 | Increase |
| San Marino | 26.97 | 0.89 | 14.55 | 12.42 | Increase |
| Sao Tome and Principe | 92.38 | 0.51 | 30.50 | 61.87 | Decrease |
| Saudi Arabia | 105.47 | 0.82 | 17.74 | 87.73 | Decrease |
| Senegal | 74.62 | 0.41 | 30.51 | 44.11 | Decrease |
| Serbia | 30.61 | 0.79 | 17.83 | 12.78 | Decrease |
| Seychelles | 63.36 | 0.73 | 19.45 | 43.92 | Decrease |
| Sierra Leone | 98.13 | 0.36 | 30.47 | 67.66 | Decrease |
| Singapore | 53.72 | 0.86 | 16.94 | 36.78 | Increase |
| Slovakia | 30.83 | 0.81 | 17.72 | 13.11 | Decrease |
| Slovenia | 27.19 | 0.84 | 17.18 | 10.00 | Decrease |
| Solomon Islands | 67.43 | 0.43 | 30.49 | 36.94 | Increase |
| Somalia | 115.60 | 0.08 | 115.56 | 0.04 | Decrease |
| South Africa | 59.13 | 0.68 | 19.43 | 39.70 | Decrease |
| South Sudan | 96.04 | 0.28 | 35.15 | 60.89 | Decrease |
| Spain | 33.67 | 0.77 | 19.47 | 14.20 | Decrease |
| Sri Lanka | 60.66 | 0.70 | 19.46 | 41.19 | Decrease |
| Sudan | 75.49 | 0.54 | 20.68 | 54.81 | Decrease |
| Suriname | 52.66 | 0.63 | 20.20 | 32.46 | Decrease |
| Sweden | 18.19 | 0.89 | 15.38 | 2.81 | Increase |
| Switzerland | 29.88 | 0.93 | 14.16 | 15.73 | Increase |
| Syrian Arab Republic | 71.42 | 0.62 | 20.46 | 50.96 | Decrease |
| Taiwan (Province of China) | 68.99 | 0.87 | 14.32 | 54.67 | Decrease |
| Tajikistan | 155.80 | 0.54 | 20.72 | 135.08 | Decrease |
| Thailand | 58.57 | 0.68 | 19.41 | 39.16 | Decrease |
| Timor-Leste | 69.08 | 0.44 | 30.49 | 38.59 | Decrease |
| Togo | 95.25 | 0.41 | 30.62 | 64.63 | Decrease |
| Tokelau | 47.78 | 0.69 | 19.48 | 28.30 | Decrease |
| Tonga | 64.10 | 0.63 | 20.49 | 43.61 | Increase |
| Trinidad and Tobago | 42.41 | 0.77 | 19.46 | 22.96 | Decrease |
| Tunisia | 65.49 | 0.68 | 19.49 | 46.00 | Decrease |
| Turkey | 39.08 | 0.71 | 19.49 | 19.59 | Decrease |
| Turkmenistan | 160.04 | 0.68 | 19.43 | 140.62 | Increase |
| Tuvalu | 59.97 | 0.58 | 20.69 | 39.28 | Increase |
| Uganda | 103.92 | 0.42 | 30.47 | 73.44 | Decrease |
| Ukraine | 85.68 | 0.76 | 19.44 | 66.24 | Increase |
| United Arab Emirates | 53.35 | 0.85 | 17.03 | 36.32 | Decrease |
| United Kingdom | 29.45 | 0.86 | 16.74 | 12.71 | Decrease |
| United Republic of Tanzania | 121.13 | 0.45 | 30.58 | 90.56 | Decrease |
| United States Virgin Islands | 44.19 | 0.82 | 17.70 | 26.49 | Decrease |
| United States of America | 32.75 | 0.86 | 16.67 | 16.09 | Decrease |
| Uruguay | 27.03 | 0.72 | 19.48 | 7.55 | Decrease |
| Uzbekistan | 156.53 | 0.66 | 19.43 | 137.10 | Increase |
| Vanuatu | 68.37 | 0.47 | 30.47 | 37.89 | Increase |
| Venezuela (Bolivarian Republic of) | 46.08 | 0.60 | 20.67 | 25.40 | Decrease |
| Viet Nam | 64.45 | 0.63 | 20.49 | 43.96 | Decrease |
| Yemen | 92.54 | 0.45 | 30.47 | 62.07 | Decrease |
| Zambia | 96.68 | 0.51 | 30.46 | 66.22 | Decrease |
| Zimbabwe | 88.98 | 0.47 | 30.46 | 58.52 | Increase |
