# Supplementary material for: Global, regional, and national burden of chronic hepatitis C including cirrhosis from 1990 to 2021, alongside projections for the next 15 years
Source: Medicine (Baltimore). 2026 Oct 16;105(42):e51118. doi: 10.1097/MD.0000000000051118 (PMC13681144; doi:10.1097/MD.0000000000051118)
Supplement: Supplementary file 7 [file medi-105-e51118-s007.docx]

Supplementary Table S16. Prediction of global incidence of chronic hepatitis C including cirrhosis trend from 2022 to 2036.

| year | Age-standardized incidence rate | sd | 0.025Q | 0.975Q | Number of incidence cases | sd | 0.025Q | 0.975Q |
| --- | --- | --- | --- | --- | --- | --- | --- | --- |
| 2022 | 57.59 | 0.90 | 55.82 | 59.36 | 4633026.61 | 72689.63 | 4490557.55 | 4775495.67 |
| 2023 | 57.17 | 1.14 | 54.92 | 59.41 | 4643417.13 | 92940.31 | 4461257.46 | 4825576.79 |
| 2024 | 56.75 | 1.47 | 53.88 | 59.62 | 4652787.84 | 120271.63 | 4417059.78 | 4888515.89 |
| 2025 | 56.34 | 1.85 | 52.71 | 59.97 | 4661076.37 | 153256.78 | 4360698.61 | 4961454.14 |
| 2026 | 55.93 | 2.29 | 51.45 | 60.41 | 4667855.79 | 190892.25 | 4293713.85 | 5041997.73 |
| 2027 | 55.52 | 2.76 | 50.11 | 60.93 | 4672956.12 | 232504.71 | 4217255.27 | 5128656.98 |
| 2028 | 55.11 | 3.27 | 48.70 | 61.52 | 4676772.27 | 277572.67 | 4132739.84 | 5220804.69 |
| 2029 | 54.71 | 3.81 | 47.25 | 62.17 | 4679554.66 | 325741.90 | 4041112.27 | 5317997.05 |
| 2030 | 54.31 | 4.37 | 45.75 | 62.88 | 4681282.34 | 376753.32 | 3942859.39 | 5419705.28 |
| 2031 | 53.92 | 4.96 | 44.20 | 63.63 | 4681594.68 | 430387.34 | 3838050.98 | 5525138.37 |
| 2032 | 53.52 | 5.56 | 42.62 | 64.42 | 4680376.43 | 486458.26 | 3726935.75 | 5633817.10 |
| 2033 | 53.12 | 6.19 | 41.00 | 65.25 | 4677959.40 | 544786.62 | 3610197.25 | 5745721.56 |
| 2034 | 52.73 | 6.83 | 39.35 | 66.12 | 4674605.44 | 605234.45 | 3488367.72 | 5860843.17 |
| 2035 | 52.35 | 7.48 | 37.68 | 67.02 | 4670319.92 | 667674.02 | 3361702.88 | 5978936.96 |
| 2036 | 51.97 | 8.15 | 35.98 | 67.95 | 4664722.57 | 731948.37 | 3230130.12 | 6099315.02 |
