# Supplementary material for: Global, regional, and national burden of chronic hepatitis C including cirrhosis from 1990 to 2021, alongside projections for the next 15 years
Source: Medicine (Baltimore). 2026 Oct 16;105(42):e51118. doi: 10.1097/MD.0000000000051118 (PMC13681144; doi:10.1097/MD.0000000000051118)
Supplement: Supplementary file 8 [file medi-105-e51118-s008.docx]

Supplementary Table S5. The number of prevalence cases and the age-standardized prevalence rate of chronic hepatitis C including cirrhosis in 1990 and 2021 at 204 countries and territories levels, and their trends from 1990 to 2021.

| location | Number of prevalence cases (95%UI) in 1990 | Number of prevalence cases (95%UI) in 2021 | 1990-2021 Percentage change(100%) | The age-standardized prevalence rate/100000 (95%UI) in 1990 | The age-standardized prevalence rate/100000 (95%UI) in 2021 | 1990-2021 EAPC (95%CI) |
| --- | --- | --- | --- | --- | --- | --- |
| Afghanistan | 260298.93(209499.38~317515.86) | 562061.39(452728.84~681427.99) | 1.16 | 3204.47(2588.51~3911.55) | 2697.34(2214.56~3271.36) | -0.7 (-0.78 ~ -0.62) |
| Albania | 32448.9(25900.47~39238.69) | 29072.01(23720.42~35128.16) | -0.1 | 1109.65(902.02~1330.82) | 919.99(745.64~1115.89) | -0.86 (-0.94 ~ -0.78) |
| Algeria | 384347.02(307387.59~468634.99) | 722008.48(587450.94~867013.54) | 0.88 | 2045.26(1651.4~2476.76) | 1734.95(1418.72~2083.35) | -0.55 (-0.59 ~ -0.51) |
| American Samoa | 498.35(394.32~621.1) | 683.11(554.52~829.55) | 0.37 | 1203.59(979.63~1480.39) | 1365.91(1108.92~1656.1) | 0.31 (0.26 ~ 0.35) |
| Andorra | 505.25(409.55~609.93) | 1030.08(840.07~1239.52) | 1.04 | 866.28(702.28~1045.27) | 862.3(704.89~1038.47) | -0.17 (-0.25 ~ -0.08) |
| Angola | 444233.14(354208.17~538230.1) | 1028770.04(828253.76~1249124.8) | 1.32 | 5890.7(4789.23~7131.22) | 4401.35(3581.77~5268.03) | -1.02 (-1.06 ~ -0.98) |
| Antigua and Barbuda | 983.18(797.09~1200.46) | 1335.27(1069.59~1628.54) | 0.36 | 1711.16(1392.41~2080.87) | 1351.88(1078.08~1649.54) | -0.78 (-0.85 ~ -0.7) |
| Argentina | 204928.86(167759.97~246789.46) | 297625.23(240038.75~359958.53) | 0.45 | 634.61(519.85~764.14) | 585.16(472.54~707.77) | 0.11 (-0.04 ~ 0.25) |
| Armenia | 147627.1(118456.41~178387.17) | 147216.75(120110.11~175231.75) | 0 | 4450.23(3586.85~5367.87) | 4492.5(3669.34~5366.26) | 0.24 (0.14 ~ 0.34) |
| Australia | 345851.57(284354.08~415758.81) | 393149.3(298862.16~504592.73) | 0.14 | 1935.09(1585.23~2329.81) | 1263.71(956.84~1602.94) | -1.25 (-1.47 ~ -1.04) |
| Austria | 80976.38(65733.89~96703.73) | 88634.9(73186.34~105249.05) | 0.09 | 861.22(702.02~1042.49) | 700.99(576.88~835.46) | -0.87 (-0.95 ~ -0.79) |
| Azerbaijan | 449493.37(361159.09~548838.88) | 650756.62(528427.76~788416.87) | 0.45 | 6465.96(5212.22~7824.2) | 5983.99(4867.06~7272.94) | -0.38 (-0.52 ~ -0.24) |
| Bahamas | 3863.94(3098.27~4666.68) | 5706.69(4616.43~6912.27) | 0.48 | 1698.52(1382.61~2043.37) | 1382.74(1124.46~1664.63) | -0.67 (-0.75 ~ -0.59) |
| Bahrain | 7234.97(5852.92~8826.87) | 22933.88(18522.24~27873.46) | 2.17 | 1934.16(1573.97~2314.98) | 1599.53(1309.37~1915.01) | -0.66 (-0.71 ~ -0.62) |
| Bangladesh | 990431.88(808182.99~1196114.35) | 1320319.14(1081592.37~1584884.37) | 0.33 | 1182.16(967.88~1413.72) | 851.1(700.77~1021.91) | -1.36 (-1.74 ~ -0.98) |
| Barbados | 3985.82(3244.2~4818.65) | 4795.09(3924~5744.44) | 0.2 | 1515.2(1227.22~1837.56) | 1299.27(1061.8~1581.91) | -0.48 (-0.52 ~ -0.45) |
| Belarus | 213981.51(172761.64~259390.28) | 228725.21(186379.43~275268.05) | 0.07 | 1934.52(1559.35~2342.67) | 2117.33(1723.41~2547.64) | 0.43 (0.25 ~ 0.62) |
| Belgium | 87946.42(72557.95~104960.45) | 122748.02(99364.61~147825) | 0.4 | 723.37(595.58~866.73) | 766.92(622.37~925.68) | 0.22 (0.16 ~ 0.28) |
| Belize | 3157.62(2496.08~3875.73) | 7039.84(5686.77~8536.26) | 1.23 | 2086.34(1672.42~2539.14) | 1752.71(1427.37~2107.33) | -0.57 (-0.6 ~ -0.55) |
| Benin | 161520.84(128801.29~198417.65) | 354704.9(283597.29~431905.3) | 1.2 | 4279.12(3427.8~5203.47) | 3347.43(2689.14~4030.25) | -0.82 (-0.85 ~ -0.79) |
| Bermuda | 935.89(759.95~1124.18) | 945.03(766.51~1139.01) | 0.01 | 1493.52(1211.14~1795.77) | 1109.13(910.25~1356.66) | -1.02 (-1.09 ~ -0.95) |
| Bhutan | 9296.76(7457.26~11325) | 16176.29(12993.51~19415.69) | 0.74 | 1850.36(1507.12~2217.54) | 2217.06(1795.32~2673.93) | 0.81 (0.68 ~ 0.95) |
| Bolivia (Plurinational State of) | 67473.28(53789.67~83430.97) | 123178.03(98361.56~149262.03) | 0.83 | 1313.61(1058.15~1599.61) | 1117.18(893.96~1349.63) | -0.53 (-0.55 ~ -0.51) |
| Bosnia and Herzegovina | 24914.22(20142.22~30276.03) | 23008.98(18682.57~27529.13) | -0.08 | 554.65(449.4~668.37) | 533.51(434.91~639.43) | -0.2 (-0.33 ~ -0.07) |
| Botswana | 22174.4(17771.28~27149.56) | 42949.66(34487.31~52076.97) | 0.94 | 2251.52(1814.4~2727.46) | 2017.04(1640.16~2401.97) | -0.44 (-0.47 ~ -0.41) |
| Brazil | 3111479.7(2431260.15~3839651.64) | 4647945.48(3643109.16~5767170.59) | 0.49 | 2400(1885.96~2939.81) | 1958.8(1529.05~2424.65) | -0.26 (-0.55 ~ 0.03) |
| Brunei Darussalam | 2258.32(1817.59~2728.9) | 5202.29(4213.66~6265.06) | 1.3 | 1273.48(1044.06~1527.55) | 1253.45(1033.08~1500.86) | -0.18 (-0.23 ~ -0.13) |
| Bulgaria | 99796.78(80574.33~119642.94) | 76701.01(62200.64~92289.8) | -0.23 | 1029.45(827.61~1242.32) | 896.58(717.85~1092.46) | -0.58 (-0.7 ~ -0.46) |
| Burkina Faso | 455117.15(359992.96~554016.09) | 830759.88(655908.26~1019019.52) | 0.83 | 5889.82(4710.95~7102.77) | 4523.41(3619.55~5430.06) | -0.83 (-0.9 ~ -0.76) |
| Burundi | 207656.36(165517.18~251381.4) | 406646.59(326155.46~497805.08) | 0.96 | 4952.16(4007.37~5904.92) | 4085.57(3312.77~4908.32) | -0.67 (-0.71 ~ -0.63) |
| Cabo Verde | 10868.67(8672.7~13298.75) | 14568.3(11691.57~17687.69) | 0.34 | 3646.56(2930.52~4395.92) | 2668.08(2152.43~3236.39) | -1.1 (-1.14 ~ -1.07) |
| Cambodia | 591885.71(474424.31~717101.33) | 815775.81(659112.34~989758.84) | 0.38 | 6387.54(5158.66~7642.15) | 4885(3950.93~5895.69) | -1 (-1.09 ~ -0.91) |
| Cameroon | 441627.63(358686.9~537149.91) | 949391.7(751076.27~1175951.12) | 1.15 | 5247.44(4293.29~6328.93) | 3670.91(2939.89~4433.65) | -1.15 (-1.18 ~ -1.11) |
| Canada | 223808.98(182312.3~271266.06) | 276976.92(212073.59~347995.77) | 0.24 | 750.16(610.61~913.49) | 565.97(435.4~705.92) | -0.57 (-0.81 ~ -0.32) |
| Central African Republic | 88994.31(72587.7~107285.83) | 163357.49(131635.13~197847.14) | 0.84 | 4440.61(3650.15~5309.8) | 3974.34(3249.59~4779.46) | -0.42 (-0.46 ~ -0.37) |
| Chad | 255120.53(205298.17~313593.7) | 608797.18(490401.22~744345.44) | 1.39 | 5266.51(4283.22~6349.8) | 4486.02(3649.57~5408.71) | -0.57 (-0.6 ~ -0.54) |
| Chile | 129877.97(106691.41~155376.13) | 206555.84(169910.79~246935.89) | 0.59 | 1082.97(886.89~1285.93) | 932.22(766.33~1109.86) | -0.29 (-0.43 ~ -0.15) |
| China | 26803824.86(21798633.26~32869400.76) | 19829778.81(15664995.19~24260961.57) | -0.26 | 2318.48(1894.98~2835.06) | 1260.41(985.1~1556.49) | -2.42 (-2.83 ~ -2) |
| Colombia | 554679.78(443614.97~678753.73) | 779376.45(632383.7~946348.52) | 0.41 | 1935.26(1556.73~2330.9) | 1497.37(1213.38~1828.87) | -0.76 (-0.97 ~ -0.55) |
| Comoros | 9600.74(7624.59~11647.31) | 14597.06(11842.97~17492.23) | 0.52 | 2852.29(2297.33~3412.17) | 2261.85(1846.39~2698.15) | -0.76 (-0.79 ~ -0.72) |
| Congo | 86917.99(69413.07~105070.39) | 173372.7(139388.26~209704.29) | 0.99 | 4859.14(3934.14~5876.21) | 3937.04(3207.64~4770.42) | -0.76 (-0.78 ~ -0.73) |
| Cook Islands | 248.71(196.01~307.89) | 239.64(194.46~287.74) | -0.04 | 1433.33(1144.59~1759.34) | 1215.17(984.07~1464.64) | -0.54 (-0.59 ~ -0.49) |
| Costa Rica | 50447.82(40852.02~61251.49) | 81181.08(66127.06~97119.2) | 0.61 | 1888.27(1541.83~2289.97) | 1588.94(1288~1899.32) | -0.57 (-0.6 ~ -0.53) |
| Croatia | 57146.8(46288.28~69256.83) | 44296.37(36107.71~53169.67) | -0.22 | 1078.37(873.22~1310.58) | 821.93(663.43~1000.21) | -0.99 (-1.04 ~ -0.94) |
| Cuba | 179171.53(145547.87~216909) | 193463.89(159613.57~231892.07) | 0.08 | 1651.03(1345.54~1998.9) | 1391.18(1145.53~1667.6) | -0.58 (-0.61 ~ -0.55) |
| Cyprus | 5526.51(4458.89~6659.66) | 10671.16(8644.31~12934.77) | 0.93 | 693.61(560.58~834.99) | 627.41(507.65~763.56) | -0.12 (-0.21 ~ -0.02) |
| Czechia | 126787.02(102258.4~152488.68) | 128909.35(105418.94~152501.18) | 0.02 | 1123.93(907.9~1357.04) | 975.66(793.39~1169.45) | -0.33 (-0.5 ~ -0.15) |
| C么te d'Ivoire | 365502.83(290363.53~449970.01) | 695912.26(554365.37~847150.21) | 0.9 | 3885.69(3131.93~4682.42) | 3078.79(2510.85~3722.75) | -0.7 (-0.73 ~ -0.67) |
| Democratic People's Republic of Korea | 456981.48(362445.77~555436.11) | 647076.4(522092.66~784239.45) | 0.42 | 2276.08(1806.56~2763.16) | 2323.34(1875.3~2836.25) | -0.17 (-0.25 ~ -0.09) |
| Democratic Republic of the Congo | 1489126.89(1201792.55~1783905.08) | 3057637.44(2458623.41~3653949.38) | 1.05 | 5307.66(4303.49~6338.68) | 4508.05(3699.06~5308.31) | -0.58 (-0.68 ~ -0.49) |
| Denmark | 46176.7(38207.14~55754.74) | 56150.94(45965.52~67530.21) | 0.22 | 732.89(602.76~889.32) | 699.21(571.82~833.65) | -0.33 (-0.43 ~ -0.23) |
| Djibouti | 7786.87(6188.9~9493.36) | 23307.29(18552.14~28138.98) | 1.99 | 2627.4(2128.96~3159.74) | 2246.4(1816.33~2710.9) | -0.56 (-0.59 ~ -0.53) |
| Dominica | 1190.59(953.87~1462.49) | 1000.25(807.25~1211.8) | -0.16 | 1801(1456.49~2191.64) | 1351.51(1086.1~1627.72) | -0.96 (-1.05 ~ -0.88) |
| Dominican Republic | 121263.87(98215.52~147661.55) | 189739.92(154278.97~228697.56) | 0.56 | 2031.64(1657.44~2445.88) | 1745.19(1424~2103.69) | -0.52 (-0.61 ~ -0.44) |
| Ecuador | 88161.04(70539.84~106804.42) | 172804.28(140930.64~207988.93) | 0.96 | 1062.35(857.7~1277.05) | 977.8(798.75~1175.51) | -0.25 (-0.29 ~ -0.21) |
| Egypt | 4150314.17(3450059.63~4877761.11) | 3107266.84(2153167.08~4027520.22) | -0.25 | 9749.13(8169.68~11475.06) | 3456.12(2354.06~4554.74) | -2.51 (-2.97 ~ -2.05) |
| El Salvador | 109266.67(88869.47~131866.09) | 122063.38(98033.32~146653.48) | 0.12 | 2373.76(1944.3~2839.95) | 1906.92(1532.86~2291.74) | -0.72 (-0.78 ~ -0.65) |
| Equatorial Guinea | 17277.32(13841.79~20953.99) | 35736.35(28741.82~43513.58) | 1.07 | 5431.72(4421.62~6516.93) | 3260.23(2635.83~3896.59) | -1.83 (-1.91 ~ -1.74) |
| Eritrea | 76745.04(61099.6~93503.14) | 136257.4(111010.61~165167.87) | 0.78 | 3210.25(2605.27~3845.38) | 2709.91(2210.05~3238.82) | -0.51 (-0.55 ~ -0.48) |
| Estonia | 31331.78(25630.21~37700.15) | 30998.97(25341.29~37131.81) | -0.01 | 1859.82(1513.9~2234.51) | 1967.35(1590.82~2369.17) | -0.09 (-0.22 ~ 0.04) |
| Eswatini | 12631.59(10044.59~15544.08) | 20048.58(15912.08~24277.1) | 0.59 | 2208.37(1765.41~2702.58) | 2152.09(1744.91~2590.11) | -0.05 (-0.12 ~ 0.02) |
| Ethiopia | 1087251.95(858496.48~1317695.11) | 1971188.6(1570066.12~2405537) | 0.81 | 3098.33(2489.66~3723.14) | 2472.58(2002.73~3013.81) | -0.88 (-0.97 ~ -0.8) |
| Fiji | 10123.93(7991.75~12446.36) | 12918.54(10432.1~15704.6) | 0.28 | 1500.24(1197.48~1826.29) | 1436.88(1160.06~1735.8) | -0.09 (-0.16 ~ -0.03) |
| Finland | 44093.22(36265.53~53254.09) | 59761.66(48760.25~71426.94) | 0.36 | 740.24(611.02~892.84) | 740.76(604.49~888.07) | -0.09 (-0.2 ~ 0.02) |
| France | 493699.43(410309.16~588112.9) | 543282.47(428717.04~657309.25) | 0.1 | 725(597.66~870.48) | 573.75(455.06~698.86) | -0.36 (-0.6 ~ -0.11) |
| Gabon | 50044.41(40522.96~60795.47) | 70987.16(57359.23~85350.75) | 0.42 | 6261.85(5066.9~7550) | 4640.18(3785.88~5533.67) | -1.08 (-1.18 ~ -0.97) |
| Gambia | 21484.7(17064.02~26465.54) | 44296.26(35555.36~54250.67) | 1.06 | 2959.13(2389.16~3578.57) | 2389.66(1949.88~2876.07) | -0.62 (-0.76 ~ -0.48) |
| Georgia | 264760.99(215460.18~316073.88) | 110507.3(77139.84~144982.58) | -0.58 | 4660.93(3792.77~5559.66) | 2900.36(2062.39~3795.61) | -1.7 (-2.19 ~ -1.21) |
| Germany | 834407.43(684664.45~1002217.27) | 906330.88(740686.16~1087735.15) | 0.09 | 840.92(689.75~1011.23) | 721.89(586.47~860.26) | -0.01 (-0.22 ~ 0.2) |
| Ghana | 668877.18(551921.77~807479.36) | 1104059.35(889844.34~1346436) | 0.65 | 5426.24(4495.47~6513.97) | 3765.69(3049.48~4534.13) | -1.31 (-1.39 ~ -1.23) |
| Greece | 67345.67(56472.65~79556.65) | 85207.93(68376.43~102264.91) | 0.27 | 539.81(454.99~634.08) | 547.32(440.69~659.54) | 0.33 (0.18 ~ 0.47) |
| Greenland | 471(383.02~565.94) | 537.35(437.47~640.35) | 0.14 | 915.75(745.79~1091.75) | 851.89(693.73~1014.12) | -0.35 (-0.39 ~ -0.31) |
| Grenada | 1667.75(1331.99~2029.2) | 1620.56(1311.41~1952.59) | -0.03 | 2129.58(1705.34~2580.31) | 1486.99(1203.69~1788.8) | -1.16 (-1.26 ~ -1.05) |
| Guam | 1644.09(1313.53~2002.03) | 2355.63(1915.12~2829.34) | 0.43 | 1312.61(1055.91~1586.89) | 1376.69(1114.15~1665.17) | 0 (-0.11 ~ 0.11) |
| Guatemala | 230995.7(186824.84~276674.82) | 363303.39(290102.65~437228.99) | 0.57 | 3355.69(2725.33~4008.6) | 2459.15(1980.32~2950.31) | -1.1 (-1.17 ~ -1.03) |
| Guinea | 224295.38(178527.74~272062.42) | 388791(311451.73~476161.42) | 0.73 | 4533.13(3642.31~5430.7) | 3635.47(2952.73~4369.47) | -0.72 (-0.76 ~ -0.69) |
| Guinea-Bissau | 26876.19(21362.69~32922.69) | 41479.18(32767.93~50544.43) | 0.54 | 3473.76(2780.99~4193.51) | 2615.43(2120.86~3135.27) | -0.93 (-1.24 ~ -0.62) |
| Guyana | 15892.28(12730.13~19457.42) | 14238.06(11496.96~17046.32) | -0.1 | 2432.16(1961.12~2939.46) | 1920.9(1552.78~2292.36) | -0.74 (-0.81 ~ -0.67) |
| Haiti | 164952.04(131700.47~202690.55) | 283697.87(226165.19~344700.42) | 0.72 | 3077.63(2467.31~3738.38) | 2475.05(1988.31~3001.84) | -0.89 (-0.94 ~ -0.83) |
| Honduras | 111076.35(88583.1~136715.47) | 213076.66(170939.57~258359.26) | 0.92 | 2865.83(2295.14~3493.21) | 2314.45(1878.88~2781.18) | -0.75 (-0.77 ~ -0.73) |
| Hungary | 152579.69(124726.89~182731.98) | 104987.17(85731.88~126080.8) | -0.31 | 1328.81(1083.76~1610.3) | 870.84(701.04~1055.43) | -1.49 (-1.54 ~ -1.44) |
| Iceland | 1688.19(1376.61~2050.61) | 1349.31(877.58~1866.33) | -0.2 | 633.2(512.59~769.46) | 316.78(205.25~437.44) | -1.45 (-1.95 ~ -0.95) |
| India | 12053478.58(9654898.75~14812582.84) | 16514073.83(13256708.69~19990525.01) | 0.37 | 1665.08(1350.4~2010.54) | 1215.8(983.03~1468.54) | -1 (-1.2 ~ -0.8) |
| Indonesia | 5418571.97(4375405.91~6614404.13) | 7491223.45(6025938.96~9044334.45) | 0.38 | 3168.55(2572.52~3839.25) | 2637.44(2142.85~3177.61) | -0.61 (-0.65 ~ -0.57) |
| Iran (Islamic Republic of) | 493594.16(391569.61~607987.08) | 902989.58(724781.68~1113857.21) | 0.83 | 1187.37(943.51~1466.67) | 1039.36(834.15~1288.46) | -1.08 (-1.64 ~ -0.52) |
| Iraq | 359732.6(289031.95~440392.59) | 862787.19(696506.31~1053319.79) | 1.4 | 2676.46(2164.15~3238.06) | 2459.79(2004.29~2960.41) | -0.24 (-0.55 ~ 0.08) |
| Ireland | 28280.42(22751.64~34345.61) | 39664.73(31861.3~48388.15) | 0.4 | 754.98(607.25~913.62) | 647.36(519.12~791.78) | -0.38 (-0.48 ~ -0.28) |
| Israel | 30936.47(25218.3~37076.74) | 66982.82(54517.12~80522.23) | 1.17 | 642.52(523.1~773.57) | 633.83(514.64~766.07) | -0.21 (-0.36 ~ -0.06) |
| Italy | 1397865.81(1135058.7~1691287.9) | 1294172.14(1043613.68~1581572.5) | -0.07 | 1985.84(1603.22~2404.35) | 1479.63(1190.62~1800.89) | -1.72 (-2.03 ~ -1.42) |
| Jamaica | 34246.02(27397.98~42010.72) | 39816.58(32295.75~48378.25) | 0.16 | 1603.34(1290.05~1942.26) | 1334.44(1080.92~1628.23) | -0.57 (-0.64 ~ -0.49) |
| Japan | 2487313.16(1994850.87~3100821.52) | 2001536.95(1507480.05~2564054.19) | -0.2 | 1584.3(1269.36~1955.87) | 842.34(666.06~1046.11) | -3.94 (-5.18 ~ -2.69) |
| Jordan | 52095.71(41275.84~63378.07) | 195093.32(156976.99~236667.89) | 2.74 | 2027.1(1640.16~2453.98) | 1818.29(1476.06~2206.82) | -0.91 (-1.28 ~ -0.55) |
| Kazakhstan | 436340.78(353818.54~520839.01) | 591788.81(480160.85~710249.41) | 0.36 | 2790.6(2265.07~3330.38) | 3122(2537.36~3741.96) | 0.42 (0.3 ~ 0.54) |
| Kenya | 417474.9(329014.47~510727) | 938530.27(747090.82~1141316.63) | 1.25 | 2599.04(2089.35~3136.65) | 2402.42(1941.71~2927.71) | -0.21 (-0.23 ~ -0.2) |
| Kiribati | 1566.92(1254.41~1923.85) | 2905.42(2315.36~3571.42) | 0.85 | 2365.71(1912.44~2885.44) | 2563.75(2068.31~3152.75) | 0.24 (0.16 ~ 0.32) |
| Kuwait | 19740.79(15861.83~24302.96) | 61547.43(49587.09~74591.88) | 2.12 | 1479.58(1200.32~1818.14) | 1335.55(1082.12~1608.04) | -0.34 (-0.41 ~ -0.28) |
| Kyrgyzstan | 247181.06(200089.43~301540.79) | 410382.31(333842.38~494310.68) | 0.66 | 5970.43(4845.19~7231.36) | 6214.92(5051.32~7460.44) | 0.17 (-0.02 ~ 0.35) |
| Lao People's Democratic Republic | 133462.49(107758.79~162825.16) | 175257.84(142662.39~211598.95) | 0.31 | 3600.37(2905.35~4377.59) | 2509.47(2038.93~3011.68) | -1.36 (-1.46 ~ -1.26) |
| Latvia | 49029.31(39669.33~59287.14) | 34711.38(27651.71~42645.1) | -0.29 | 1704.14(1368.18~2085.4) | 1539.69(1209.31~1896.61) | -0.15 (-0.29 ~ -0.01) |
| Lebanon | 39454.85(31725.06~47550.23) | 67311.19(55242.28~81581.37) | 0.71 | 1517.55(1233.71~1829.54) | 1142.55(936.12~1382.17) | -1 (-1.47 ~ -0.53) |
| Lesotho | 27555.89(21943.45~33573.86) | 37316.13(29998.13~45545.06) | 0.35 | 2266.73(1826.62~2742.65) | 2361.69(1894.73~2843.01) | 0.17 (0.15 ~ 0.2) |
| Liberia | 86366.14(67956.47~106002.52) | 157576.7(125650.92~193552.66) | 0.82 | 4375.71(3499.96~5296.16) | 3524.79(2823.27~4268.85) | -0.84 (-0.89 ~ -0.78) |
| Libya | 56429.34(45422.18~69200.65) | 103074.51(83428.65~124150.11) | 0.83 | 1860.87(1510.77~2257.54) | 1533.27(1251.43~1833.7) | -0.66 (-1.4 ~ 0.09) |
| Lithuania | 83929.62(67346.68~102016.52) | 71673.55(58911.17~85139.72) | -0.15 | 2161.48(1731.8~2640.62) | 2194.28(1788.29~2633.59) | 0.04 (-0.06 ~ 0.14) |
| Luxembourg | 4136.82(3406.31~4940.94) | 6041.82(4960.95~7236.77) | 0.46 | 906.46(743.81~1092.23) | 728.61(600.85~869.93) | -0.86 (-0.93 ~ -0.79) |
| Madagascar | 179661.66(144791.66~217737.91) | 378223.52(302632.27~457132.21) | 1.11 | 2119.26(1730.97~2576.62) | 1822.85(1484.53~2177.98) | -0.36 (-0.65 ~ -0.08) |
| Malawi | 237830.33(189816.05~288643.67) | 383348(307732.14~466241.32) | 0.61 | 3364.95(2713~4045.56) | 2692.2(2181.62~3210.64) | -0.85 (-0.91 ~ -0.79) |
| Malaysia | 562206.25(450627.45~682551.08) | 927987.27(744263.04~1122160.18) | 0.65 | 3453.98(2794.85~4149.09) | 2888.68(2325.37~3487.17) | -0.55 (-0.69 ~ -0.41) |
| Maldives | 8629.06(6971.17~10550.63) | 14731.85(11851.33~17762.52) | 0.71 | 4430.09(3600.17~5332.13) | 2810.97(2277.86~3410.67) | -1.63 (-1.74 ~ -1.53) |
| Mali | 336157.54(268744.17~419644.89) | 652022(521719.06~798908.93) | 0.94 | 4805.39(3873.73~5890.95) | 3505.67(2840.02~4217.78) | -1.12 (-1.16 ~ -1.09) |
| Malta | 3005.91(2428.16~3645.75) | 4297.75(3515.46~5184.27) | 0.43 | 752.69(607.44~912.69) | 682.46(554.09~827.51) | -0.34 (-0.42 ~ -0.27) |
| Marshall Islands | 861.65(682.36~1059.97) | 1250.1(1004.96~1510.62) | 0.45 | 2237.17(1799.15~2719.32) | 2335.05(1885.85~2811.98) | 0.13 (0.1 ~ 0.16) |
| Mauritania | 62024.59(49354.59~76463.38) | 99361.33(79769.6~121837.28) | 0.6 | 3755.67(3017.36~4571.66) | 2772.03(2240.75~3349.74) | -1.03 (-1.04 ~ -1.02) |
| Mauritius | 39753.82(31887.88~48467.14) | 43652.57(35892.17~52239.38) | 0.1 | 3735.42(3009.51~4530.95) | 3136.22(2572.29~3791.51) | -0.67 (-0.72 ~ -0.62) |
| Mexico | 1266507.51(982719.35~1597005.72) | 1823854.43(1448752.54~2248689.76) | 0.44 | 1755.77(1364~2188.24) | 1371.55(1090.16~1687.81) | 0.19 (-0.27 ~ 0.64) |
| Micronesia (Federated States of) | 1882.5(1497.91~2301.01) | 2220.27(1769.26~2706.51) | 0.18 | 2088.54(1694.83~2530.88) | 2241.9(1800.86~2720.48) | 0.19 (0.15 ~ 0.22) |
| Monaco | 292.14(236.37~352.36) | 456.31(366.33~549.74) | 0.56 | 633.06(516.29~763.5) | 755.93(617.54~904.13) | 0.62 (0.56 ~ 0.68) |
| Mongolia | 232667.9(186923.59~284317.31) | 282414.48(204654.68~364663.41) | 0.21 | 11749.08(9511.14~14254.44) | 8589.89(6259.61~11024.28) | -0.29 (-0.62 ~ 0.05) |
| Montenegro | 5733.61(4558~7035.69) | 6349.99(5195.38~7648.28) | 0.11 | 904.6(719.57~1107.26) | 883.85(723.81~1069.52) | -0.19 (-0.27 ~ -0.12) |
| Morocco | 446832.39(357671.46~549291.02) | 686070.47(552432.18~834511.35) | 0.54 | 2228.36(1799.46~2722.88) | 1856.36(1499.59~2249.53) | -0.55 (-0.98 ~ -0.12) |
| Mozambique | 247991.91(198653.27~301434.39) | 476098.94(381461.39~573451.52) | 0.92 | 2521.01(2047.99~3024.32) | 2208.35(1794.59~2648.38) | -0.39 (-0.42 ~ -0.35) |
| Myanmar | 1445985.02(1167699.14~1753451.68) | 1834982.51(1488747.43~2187382.09) | 0.27 | 3832.99(3117.77~4617.31) | 3268.64(2658.28~3897.59) | -0.63 (-0.67 ~ -0.59) |
| Namibia | 22292.34(17616.01~27432.42) | 40160.81(32079.35~48926.52) | 0.8 | 2081.54(1665.3~2548.8) | 1957.51(1588.58~2366.95) | -0.38 (-0.44 ~ -0.32) |
| Nauru | 164.59(132.23~201.8) | 209.46(167.9~257.65) | 0.27 | 1855.24(1495.75~2249.9) | 2089.72(1676.44~2562.64) | 0.36 (0.31 ~ 0.4) |
| Nepal | 154634.56(124256.49~187204.44) | 245875.29(200203.48~295619.24) | 0.59 | 1004.14(819.86~1212.93) | 880.93(721.29~1051.43) | -0.53 (-0.91 ~ -0.15) |
| Netherlands | 87607.19(69695.82~106431.25) | 116697.63(94736.76~139607.97) | 0.33 | 503.72(400.46~610.63) | 475.16(384.34~575.09) | -0.38 (-0.61 ~ -0.14) |
| New Zealand | 34521.81(27657.17~41844.71) | 67699.2(54448.5~82020.73) | 0.96 | 953.31(764.68~1155) | 1065.33(855.03~1294.22) | 0.54 (0.47 ~ 0.61) |
| Nicaragua | 107662.33(87275.35~130260.86) | 161237.69(129545.08~195091.44) | 0.5 | 3355.6(2763.83~3989.04) | 2540.95(2056.9~3039.37) | -0.88 (-1.06 ~ -0.69) |
| Niger | 304840.44(239448.65~377694.79) | 753997.29(595167.78~927456.28) | 1.47 | 4917.82(3957.28~5971.03) | 3996.76(3241.03~4835.46) | -0.76 (-0.79 ~ -0.73) |
| Nigeria | 4268886.16(3413821.18~5251385.22) | 6987086.87(5567040.63~8590050.34) | 0.64 | 5662.32(4536.73~6871.81) | 3756.8(3025.23~4545.76) | -1.48 (-1.71 ~ -1.25) |
| Niue | 34.8(27.95~42.72) | 26.54(21.53~31.82) | -0.24 | 1556.11(1248.7~1904.03) | 1480.98(1198.53~1783.29) | -0.27 (-0.31 ~ -0.23) |
| North Macedonia | 19326.51(15425.34~23608.59) | 23529.34(19438.65~28297.19) | 0.22 | 972.56(780.29~1186.81) | 919.6(755.25~1119.58) | -0.28 (-0.31 ~ -0.24) |
| Northern Mariana Islands | 633.26(502.9~778.77) | 822.68(661.71~990.5) | 0.3 | 1529.98(1228.08~1864.87) | 1617.28(1303.27~1934.11) | 0.18 (0.14 ~ 0.23) |
| Norway | 29011.04(23632.33~35422.22) | 31625.15(25649.79~38672.47) | 0.09 | 557.21(445.6~680.7) | 432.7(345.59~530.14) | -0.44 (-0.62 ~ -0.26) |
| Oman | 29165.91(23249.89~35763.26) | 61383.43(49809.51~75285.72) | 1.1 | 2094.48(1681.56~2529.25) | 1576.9(1288.52~1895.84) | -0.89 (-0.92 ~ -0.85) |
| Pakistan | 3035053.83(2454185.53~3705355.08) | 5472643.67(4224168.8~6895781.81) | 0.8 | 3213.36(2645.19~3876.73) | 2614.04(2034.33~3239.76) | -0.14 (-0.55 ~ 0.27) |
| Palau | 223.61(177.17~276.22) | 341.13(277.86~411.51) | 0.53 | 1563.26(1248.81~1908.73) | 1723.12(1409.52~2073.1) | 0.27 (0.25 ~ 0.29) |
| Palestine | 32007.53(25684.44~39092.39) | 74451.32(60590.95~89834.91) | 1.33 | 2295.72(1856.4~2773.57) | 1879.98(1543.33~2251.08) | -0.61 (-0.65 ~ -0.57) |
| Panama | 35341.07(28397.1~43088.9) | 60165.68(48522.93~72106.39) | 0.7 | 1651.19(1342.32~2000.09) | 1377.05(1109.06~1652) | -0.5 (-0.55 ~ -0.46) |
| Papua New Guinea | 98607.07(79035.52~121178.49) | 249961.84(202308.27~304391.54) | 1.53 | 2677.65(2178.22~3266.27) | 2613.84(2128.07~3133.27) | -0.27 (-0.35 ~ -0.18) |
| Paraguay | 68624.05(55220.44~83885.8) | 127081.83(103075.04~152363.89) | 0.85 | 2064.45(1674.43~2496.22) | 1854.73(1511.77~2215.29) | -0.29 (-0.33 ~ -0.26) |
| Peru | 208236.45(167933.57~252887.58) | 344899.35(275786.69~420900.03) | 0.66 | 1149.88(938.08~1386.34) | 955.52(769.26~1160.71) | -0.61 (-0.74 ~ -0.49) |
| Philippines | 1363733.9(1080034.8~1685532.48) | 2209270.79(1765500.42~2698562.22) | 0.62 | 2461.45(1959~3000.92) | 2035.81(1628.78~2476.68) | -0.57 (-0.63 ~ -0.51) |
| Poland | 443650.84(354920.23~543223.26) | 428900.42(345659.16~521917.94) | -0.03 | 1106.42(883.02~1354.01) | 911.45(726.78~1109.05) | -0.36 (-0.52 ~ -0.2) |
| Portugal | 125883.07(103104.87~152788.48) | 175635.78(142532.02~212371.18) | 0.4 | 1086.83(885.6~1326.61) | 1173.8(936.32~1434.54) | 0.48 (0.36 ~ 0.6) |
| Puerto Rico | 65237.37(53663.29~79194.95) | 57267.22(46851.08~68471.75) | -0.12 | 1799.01(1480.48~2182.39) | 1297.85(1051.19~1559.27) | -1.17 (-1.23 ~ -1.12) |
| Qatar | 6092.17(4910.57~7420.3) | 37665.76(30065.1~45904.83) | 5.18 | 1846.83(1507.25~2219.24) | 1515.14(1231.08~1831.67) | -0.81 (-0.88 ~ -0.73) |
| Republic of Korea | 393656.44(327476.17~465950.37) | 677942.01(543976.07~821984.88) | 0.72 | 1077.38(898.39~1265.02) | 870.27(708.36~1049.19) | -1.12 (-1.46 ~ -0.79) |
| Republic of Moldova | 133220.86(106555.57~161496.52) | 117183.54(94989.06~140813.81) | -0.12 | 2980.66(2381.65~3614.6) | 2800.5(2249.94~3387.44) | -0.03 (-0.22 ~ 0.17) |
| Romania | 379511.34(306974.04~461907.56) | 232364.6(178180.53~290698.15) | -0.39 | 1546.83(1250.39~1886.5) | 1022.15(770.33~1290.33) | -0.99 (-1.27 ~ -0.72) |
| Russian Federation | 2865520.94(2278033.84~3487819.8) | 3623938.83(2893605.7~4403504.65) | 0.26 | 1792.58(1421.42~2183.95) | 2165.48(1742.2~2644.12) | 0.79 (0.56 ~ 1.02) |
| Rwanda | 182733.35(148248.19~220942.48) | 192358.7(142602.11~244192.53) | 0.05 | 3564.44(2910.62~4244.69) | 1606.8(1189.8~2072.39) | -2.14 (-2.5 ~ -1.78) |
| Saint Kitts and Nevis | 783.72(639.31~945.45) | 915.88(734.83~1110.79) | 0.17 | 2040.42(1674.05~2460.93) | 1394.82(1123.86~1683) | -1.32 (-1.45 ~ -1.2) |
| Saint Lucia | 2399.09(1907.8~2909.24) | 3057.1(2495.71~3705.06) | 0.27 | 2038.11(1630.03~2462.08) | 1497.63(1217.3~1812.64) | -1.03 (-1.17 ~ -0.9) |
| Saint Vincent and the Grenadines | 1815.08(1461.98~2212.03) | 1851.13(1512.31~2216.73) | 0.02 | 1906.08(1540.72~2309.64) | 1481.19(1215.46~1777.52) | -0.81 (-0.91 ~ -0.72) |
| Samoa | 2829.57(2255.39~3499.92) | 4416.12(3560.91~5351.6) | 0.56 | 1911.27(1535.64~2332.57) | 2237.87(1809.07~2703.8) | 0.45 (0.33 ~ 0.57) |
| San Marino | 196.15(158.9~235.29) | 343.53(276.44~417.8) | 0.75 | 679.69(548.4~815.8) | 699.24(569.09~851.7) | 0.07 (0.04 ~ 0.11) |
| Sao Tome and Principe | 3818.91(3028.03~4696.11) | 5588.89(4455.04~6744.36) | 0.46 | 3852.29(3106.65~4676.49) | 2988.78(2412.06~3556.98) | -0.92 (-0.99 ~ -0.85) |
| Saudi Arabia | 395818.08(324702.93~471642.12) | 969770.18(790741.35~1174309.53) | 1.45 | 3551.8(2942.4~4276.99) | 2821.26(2305.27~3354.62) | -0.57 (-0.64 ~ -0.5) |
| Senegal | 140385.5(113636.75~172058.29) | 300919.37(243649.27~364821.5) | 1.14 | 2445.89(1978.18~2951.32) | 2334.1(1899.43~2793.39) | -0.15 (-0.18 ~ -0.11) |
| Serbia | 99145.61(79313.6~119963.9) | 100191.55(81989.55~119643.67) | 0.01 | 966.43(773.81~1174.45) | 905(726.37~1083.06) | -0.34 (-0.43 ~ -0.26) |
| Seychelles | 1738.42(1412.98~2114.32) | 2485.34(2034.65~3006.95) | 0.43 | 2505.68(2036.44~3041.37) | 2237.41(1827.56~2697.14) | -0.3 (-0.33 ~ -0.27) |
| Sierra Leone | 143780.48(115865.26~174531.66) | 241171.01(192318.71~292065.61) | 0.68 | 4229.34(3429.4~5125.71) | 3316.72(2681.1~3972.79) | -0.87 (-0.95 ~ -0.79) |
| Singapore | 34270.8(27591.67~41733.14) | 95866.54(77312.87~114792) | 1.8 | 1250.12(1013.55~1507.53) | 1284.09(1041.02~1532.47) | 0 (-0.04 ~ 0.04) |
| Slovakia | 61317.1(49697.48~74190.89) | 62053.64(50632.33~74407.09) | 0.01 | 1114.29(902.71~1349.3) | 949.72(775.45~1139.73) | -0.31 (-0.41 ~ -0.22) |
| Slovenia | 22581.45(18272.5~27492.69) | 21510.86(17652.28~25768.7) | -0.05 | 1051.4(849.95~1283.27) | 797.51(653.04~966.06) | -0.87 (-0.93 ~ -0.8) |
| Solomon Islands | 5814.61(4665.8~7133.21) | 15369.86(12383.54~18603.07) | 1.64 | 2018.83(1631.8~2455.43) | 2474.69(2000.5~2983.68) | 0.74 (0.66 ~ 0.82) |
| Somalia | 202766.13(160293.42~249985.91) | 503935.05(399396.93~617829.69) | 1.49 | 3640.12(2907.33~4392.35) | 3386.71(2751.42~4052.11) | -0.25 (-0.29 ~ -0.21) |
| South Africa | 517052.8(407712.4~634539.36) | 920606.98(733509.41~1132732.09) | 0.78 | 1698.55(1359.61~2067.66) | 1688.96(1354.29~2071.12) | -0.09 (-0.27 ~ 0.09) |
| South Sudan | 130852.38(104674.35~159632.97) | 192354.88(154432.32~233066.37) | 0.47 | 3023.78(2440.63~3656.31) | 2705.04(2208.72~3257.94) | -0.38 (-0.4 ~ -0.37) |
| Spain | 516257.77(436714.87~605334.94) | 465889.32(359823.29~571671.42) | -0.1 | 1151.44(977.56~1347.23) | 707.76(549.95~872.23) | -1.37 (-1.51 ~ -1.23) |
| Sri Lanka | 403712.01(329571.01~497149.35) | 505151.27(410439.6~610043.83) | 0.25 | 2485.33(2027.53~3036.05) | 2151.4(1752.83~2585.84) | -0.66 (-0.8 ~ -0.52) |
| Sudan | 386068.1(306995.79~469234.87) | 679393.44(547617.92~828147) | 0.76 | 2627.37(2120.55~3171.9) | 2066.97(1682.25~2487.67) | -0.86 (-0.89 ~ -0.84) |
| Suriname | 6983.37(5637.65~8554.2) | 10161.28(8206~12262.76) | 0.46 | 2015.58(1640.16~2450.05) | 1671.61(1349.36~2019.88) | -0.68 (-0.74 ~ -0.63) |
| Sweden | 51516.17(41781.79~62836.12) | 67614.67(54437.05~82369.72) | 0.31 | 461.84(370.25~568.13) | 461.5(370.05~562.32) | 0.04 (0 ~ 0.07) |
| Switzerland | 65479.17(52851.18~78778.84) | 100386.75(81957.14~120610.77) | 0.53 | 783.29(630.18~953.48) | 811.58(661.05~989.59) | -0.03 (-0.1 ~ 0.05) |
| Syrian Arab Republic | 199388.94(160216.16~244335.87) | 266807.41(215823.74~321693.04) | 0.34 | 2238.59(1803.5~2698.05) | 1891.97(1542.23~2281.18) | -0.48 (-0.56 ~ -0.39) |
| Taiwan (Province of China) | 609326.66(523603.43~708581.45) | 713230.17(579648.15~858591.12) | 0.17 | 2993.15(2569.97~3491.95) | 2726.09(2200.28~3297.53) | -0.7 (-0.93 ~ -0.48) |
| Tajikistan | 326034.17(265373.83~391302.66) | 621029.83(500574.32~749659.85) | 0.9 | 6738.72(5490.23~8081.58) | 6485.9(5251.78~7784.37) | -0.21 (-0.29 ~ -0.14) |
| Thailand | 1438161.21(1174424.73~1734441.02) | 1566826.57(1273958.39~1878669.79) | 0.09 | 2633.57(2157.65~3169.25) | 2032.52(1658.02~2438.74) | -1.34 (-1.63 ~ -1.05) |
| Timor-Leste | 21592.63(17239.73~26515.6) | 31554.66(25115.77~38337.08) | 0.46 | 3171.97(2560.74~3851.34) | 2488.9(1993.26~3019.81) | -0.9 (-0.99 ~ -0.81) |
| Togo | 110549.09(87463.85~136493.54) | 220871.4(176896.77~268669.38) | 1 | 3947.57(3171.35~4765.08) | 3150.2(2546.42~3791.78) | -0.78 (-0.82 ~ -0.74) |
| Tokelau | 26.61(21.32~32.76) | 23.55(18.99~28.44) | -0.11 | 1783.71(1432.15~2189.05) | 1680.61(1358.02~2033.8) | -0.29 (-0.33 ~ -0.24) |
| Tonga | 1832.44(1465.73~2264.26) | 2205.24(1777.36~2653.46) | 0.2 | 2103.09(1691.55~2567.86) | 2230.03(1809.68~2672.34) | 0.13 (0.09 ~ 0.17) |
| Trinidad and Tobago | 18967.8(15280.08~23100.26) | 21465.24(17613.99~25945) | 0.13 | 1739.76(1401.32~2107.73) | 1347.97(1099.93~1634.86) | -0.88 (-0.97 ~ -0.78) |
| Tunisia | 121044.45(97340.68~145458.09) | 230690.7(188536.08~275728.48) | 0.91 | 1807.06(1473.92~2180.35) | 1793.92(1460.3~2143.42) | 0.46 (0.26 ~ 0.67) |
| Turkmenistan | 208809.37(170182.83~251954.14) | 332241.68(274111.13~400177.73) | 0.59 | 6215.6(5078.31~7439.58) | 6547.41(5392.82~7882.83) | 0.22 (0.17 ~ 0.28) |
| Tuvalu | 173.35(138.37~210.95) | 260.91(210.01~313.66) | 0.51 | 1991.44(1596.61~2418.02) | 2171.88(1755.78~2599.71) | 0.3 (0.27 ~ 0.32) |
| T眉rkiye | 550004.76(448005.89~661662.43) | 918332.98(740979.5~1105045.59) | 0.67 | 1176.76(957.49~1409.5) | 1008.9(818.02~1213.19) | -0.44 (-0.85 ~ -0.02) |
| Uganda | 483368.99(385978.57~593410.28) | 913264.45(722346.89~1112247.73) | 0.89 | 3909.91(3133.61~4680.43) | 2974.37(2414.2~3575.51) | -0.96 (-1.06 ~ -0.85) |
| Ukraine | 1366546.28(1072192.31~1670336.6) | 1530012.22(1234745.39~1872300.75) | 0.12 | 2401.19(1891.65~2944.89) | 3056.46(2455.29~3748.28) | 1.06 (0.77 ~ 1.34) |
| United Arab Emirates | 21990.14(17477.79~27030.51) | 132896.95(106503.31~161917.16) | 5.04 | 1611.88(1307.1~1955.26) | 1353.43(1093.75~1622.89) | -0.53 (-0.67 ~ -0.39) |
| United Kingdom | 667702.87(538344.52~807764.91) | 707002.21(560531.47~877102.43) | 0.06 | 977.56(784.45~1191.02) | 794.26(627.46~980.02) | 0.15 (-0.6 ~ 0.91) |
| United Republic of Tanzania | 737747.61(585997.47~897131.43) | 1645041.92(1329423.96~1974746.38) | 1.23 | 3839(3089.68~4586.51) | 3624.57(2955.39~4358.03) | -0.32 (-0.48 ~ -0.14) |
| United States Virgin Islands | 1711.69(1376.43~2104.43) | 1537.68(1245.17~1832.84) | -0.1 | 1689.5(1371.32~2062.49) | 1350.1(1094.67~1626.94) | -0.79 (-0.84 ~ -0.74) |
| United States of America | 2653473.26(2122903.39~3238471.5) | 3757345.49(3060950.28~4542040.77) | 0.42 | 958.4(762.59~1165.86) | 904.83(730.35~1094.16) | -0.33 (-0.43 ~ -0.23) |
| Uruguay | 22613.16(18319.78~27015.64) | 28241.4(23000.96~34204.28) | 0.25 | 661.61(539.04~794.3) | 655.46(534.81~791.6) | -0.07 (-0.15 ~ 0.02) |
| Uzbekistan | 1095872.27(906910.75~1304307.22) | 2127626.88(1730791.27~2551214.3) | 0.94 | 5801.75(4799.45~6867.2) | 6338.28(5177.37~7590) | 0.27 (0.13 ~ 0.41) |
| Vanuatu | 3015.12(2349.5~3741.93) | 7250.51(5855.95~8762.97) | 1.4 | 2303.8(1846.38~2822.57) | 2531.11(2054.88~3051.15) | 0.26 (0.23 ~ 0.29) |
| Venezuela (Bolivarian Republic of) | 289747.08(234415.91~352168.72) | 425251.41(340909.04~513205.38) | 0.47 | 1788.44(1458.17~2151.99) | 1513.99(1211.81~1825.73) | -0.83 (-0.95 ~ -0.72) |
| Viet Nam | 1969495.4(1583361.25~2382794.99) | 2330171.18(1889056.68~2784828.45) | 0.18 | 3190.94(2578.75~3838.89) | 2262.94(1838.44~2699.77) | -1.01 (-1.31 ~ -0.7) |
| Yemen | 318977.82(260139.82~381710.25) | 659780.56(527484.42~804213.73) | 1.07 | 3485.52(2868.04~4181.41) | 2641.61(2123.53~3189.14) | -0.76 (-0.98 ~ -0.54) |
| Zambia | 179922.01(144300.01~217379.29) | 357121.44(283572.47~431833.05) | 0.98 | 3240.08(2608.27~3863.5) | 2564.36(2087.03~3047.35) | -0.89 (-0.97 ~ -0.8) |
| Zimbabwe | 168273.16(134091.2~203974.37) | 299382.41(239660.51~362659.49) | 0.78 | 2265.48(1833.4~2731.88) | 2493.53(2037.34~2975.3) | 0.38 (0.31 ~ 0.45) |
