# Supplementary material for: Global, regional, and national burden of chronic hepatitis C including cirrhosis from 1990 to 2021, alongside projections for the next 15 years
Source: Medicine (Baltimore). 2026 Oct 16;105(42):e51118. doi: 10.1097/MD.0000000000051118 (PMC13681144; doi:10.1097/MD.0000000000051118)
Supplement: Supplementary file 9 [file medi-105-e51118-s009.docx]

Supplementary Table S6. The number of deaths cases and the age-standardized mortality rate of chronic hepatitis C including cirrhosis in 1990 and 2021 at 204 countries and territories levels, and their trends from 1990 to 2021.

| location | Number of deaths cases (95%UI) in 1990 | Number of deaths cases (95%UI) in 2021 | 1990-2021 Percentage change(100%) | The age-standardized mortality rate/100000 (95%UI) in 1990 | The age-standardized mortality rate/100000 (95%UI) in 2021 | 1990-2021 EAPC (95%CI) |
| --- | --- | --- | --- | --- | --- | --- |
| Afghanistan | 1551.93(962.76~2341.12) | 1493.72(866.43~2269.7) | -0.04 | 24.33(14.92~36.94) | 15.66(9.65~23) | -1.65 (-1.83 ~ -1.47) |
| Albania | 57.98(41.13~77.79) | 51.11(34.07~75.07) | -0.12 | 2.75(1.98~3.75) | 1.25(0.84~1.78) | -2.34 (-2.56 ~ -2.12) |
| Algeria | 801.2(547.93~1151.78) | 1616.44(1163.34~2264.4) | 1.02 | 8.1(5.85~10.94) | 5.41(4~7.48) | -1.12 (-1.2 ~ -1.05) |
| American Samoa | 0.96(0.64~1.37) | 1.34(0.91~1.93) | 0.4 | 3.33(2.23~4.77) | 2.66(1.85~3.73) | -0.79 (-1.05 ~ -0.54) |
| Andorra | 1.62(1.01~2.52) | 2.09(1.27~3.11) | 0.29 | 2.77(1.75~4.29) | 1.39(0.85~2.06) | -2 (-2.2 ~ -1.79) |
| Angola | 1121.99(761.74~1537.16) | 2115.05(1497.04~2940.24) | 0.89 | 24.23(16.6~33.43) | 14.68(10.42~20.18) | -1.59 (-1.67 ~ -1.52) |
| Antigua and Barbuda | 2.25(1.7~2.94) | 2.64(1.95~3.56) | 0.17 | 4.29(3.26~5.61) | 2.46(1.82~3.25) | -1.96 (-2.4 ~ -1.52) |
| Argentina | 2198.79(1737.58~2672.02) | 2265.54(1778.84~2825.18) | 0.03 | 6.84(5.47~8.31) | 4.15(3.28~5.17) | -1.05 (-1.32 ~ -0.78) |
| Armenia | 111.83(83.69~150.06) | 154.53(112.33~206.42) | 0.38 | 3.91(2.95~5.14) | 3.71(2.72~4.94) | 0.72 (-0.22 ~ 1.67) |
| Australia | 428.98(351.61~519.07) | 658.9(529.98~803.89) | 0.54 | 2.25(1.84~2.72) | 1.65(1.34~2) | -0.68 (-0.89 ~ -0.47) |
| Austria | 486.69(355.11~666.08) | 301.97(222.38~408.69) | -0.38 | 4.66(3.39~6.44) | 1.86(1.36~2.52) | -2.97 (-3.11 ~ -2.83) |
| Azerbaijan | 440.38(325.9~575.3) | 638.93(430.04~909.75) | 0.45 | 8.75(6.51~11.31) | 6.41(4.33~9.21) | -1.28 (-1.68 ~ -0.88) |
| Bahamas | 9.81(7.04~13.15) | 13.52(9.23~20.12) | 0.38 | 5.68(4.14~7.52) | 3.15(2.22~4.64) | -2.03 (-2.41 ~ -1.65) |
| Bahrain | 21.84(16.11~27.4) | 55.23(41.52~74.4) | 1.53 | 16.19(11.99~20.39) | 8.43(6.04~10.96) | -2.9 (-3.39 ~ -2.42) |
| Bangladesh | 6348.61(4630.77~8516.98) | 7640.14(5456.94~10286.05) | 0.2 | 12.12(8.8~16.47) | 5.74(4.14~7.66) | -2.62 (-2.83 ~ -2.41) |
| Barbados | 7.97(5.9~10.45) | 6.51(4.25~9.55) | -0.18 | 2.98(2.18~3.99) | 1.34(0.89~1.95) | -2.81 (-3.06 ~ -2.55) |
| Belarus | 199.03(145.68~266.07) | 478.51(332.77~668.27) | 1.4 | 1.55(1.14~2.05) | 3.41(2.32~4.79) | 2.93 (1.65 ~ 4.23) |
| Belgium | 379.24(287.98~494.08) | 347.58(252.72~463.55) | -0.08 | 2.69(2.06~3.52) | 1.72(1.27~2.28) | -1.42 (-1.56 ~ -1.28) |
| Belize | 4.79(3.66~6.2) | 14.75(10.67~19.81) | 2.08 | 4.93(3.7~6.45) | 4.47(3.21~5.92) | -0.54 (-0.87 ~ -0.21) |
| Benin | 259.32(177.71~372.8) | 463.57(306.72~669.04) | 0.79 | 12.34(8.41~17.62) | 7.91(5.5~11.25) | -1.38 (-1.47 ~ -1.3) |
| Bermuda | 1.94(1.45~2.6) | 1.37(0.98~1.9) | -0.29 | 3.07(2.31~4.1) | 1.1(0.77~1.51) | -3.4 (-4.03 ~ -2.77) |
| Bhutan | 25.46(14~45.1) | 41(26.17~69.31) | 0.61 | 8.51(5.01~14.53) | 6.38(4.05~10.58) | -0.96 (-0.99 ~ -0.93) |
| Bolivia (Plurinational State of) | 130.44(65.67~212.24) | 232.76(130.72~373.02) | 0.78 | 3.82(1.94~6.04) | 2.52(1.42~3.96) | -1.43 (-1.51 ~ -1.36) |
| Bosnia and Herzegovina | 150.11(103.01~214.7) | 104.39(66.34~150.73) | -0.3 | 3.36(2.35~4.68) | 1.8(1.15~2.54) | -2.16 (-2.38 ~ -1.93) |
| Botswana | 68.95(39.21~121.86) | 90.43(63.47~127.95) | 0.31 | 11.45(6.85~19.33) | 5.51(4.03~7.56) | -2.46 (-2.71 ~ -2.21) |
| Brazil | 6187.21(5676.4~6686.48) | 8487.04(7573.14~9384.9) | 0.37 | 5.94(5.46~6.43) | 3.32(2.97~3.68) | -1.76 (-1.91 ~ -1.61) |
| Brunei Darussalam | 4.21(3.06~5.63) | 8.38(5.97~11.38) | 0.99 | 3.39(2.42~4.64) | 2.07(1.52~2.69) | -1.2 (-1.43 ~ -0.97) |
| Bulgaria | 364.73(258.85~518.95) | 470.67(321.92~660.52) | 0.29 | 3.03(2.21~4.14) | 4.01(2.76~5.54) | 0.85 (0.6 ~ 1.1) |
| Burkina Faso | 409.42(281.62~561.67) | 710.63(483.34~1022.55) | 0.74 | 9.62(6.69~13.28) | 7.31(5.07~10.17) | -0.76 (-0.87 ~ -0.66) |
| Burundi | 484.21(314.24~687.03) | 719.15(491.57~992.81) | 0.49 | 19.3(12.87~27.22) | 12.9(8.99~17.68) | -1.73 (-1.88 ~ -1.58) |
| Cabo Verde | 16.22(10.6~26.19) | 26.37(16.82~38.06) | 0.63 | 7.26(4.75~11.76) | 5.53(3.5~7.9) | -1.37 (-1.58 ~ -1.16) |
| Cambodia | 1322.56(856.83~2155.27) | 2025.24(1337.02~2876.29) | 0.53 | 26.3(16.66~44.38) | 15.43(10.34~21.98) | -1.93 (-2.01 ~ -1.86) |
| Cameroon | 479.22(308.32~677.54) | 1055.73(626.19~1611.55) | 1.2 | 10.12(6.58~14.34) | 7.16(4.19~10.87) | -0.99 (-1.13 ~ -0.85) |
| Canada | 1323.65(1080.4~1577.65) | 2215.54(1825.36~2673.57) | 0.67 | 4.15(3.39~4.94) | 3.44(2.85~4.11) | -0.5 (-0.66 ~ -0.33) |
| Central African Republic | 333.5(230.11~470.16) | 623.31(417.65~891.98) | 0.87 | 24.5(17.26~33.81) | 21.97(15.13~30.36) | -0.34 (-0.42 ~ -0.25) |
| Chad | 368.86(249.44~573.09) | 817.24(516.74~1255.39) | 1.22 | 12.74(8.56~19.64) | 12.49(8.07~19.21) | -0.04 (-0.17 ~ 0.09) |
| Chile | 1481.28(1203.38~1800.38) | 1532.92(1195.04~1909.98) | 0.03 | 13.96(11.37~17) | 6.11(4.79~7.6) | -2.09 (-2.29 ~ -1.89) |
| China | 20747.23(16692.16~25755.2) | 16665.36(13022.56~21553.13) | -0.2 | 2.38(1.93~2.94) | 0.82(0.64~1.05) | -3.37 (-3.52 ~ -3.23) |
| Colombia | 574.71(451.36~708.33) | 1065.04(785.4~1398.85) | 0.85 | 3.03(2.35~3.76) | 1.94(1.43~2.53) | -2 (-2.16 ~ -1.84) |
| Comoros | 28.82(18.96~43.94) | 52.14(36.47~71.51) | 0.81 | 13.64(9.03~20.12) | 10.12(6.94~13.99) | -1.17 (-1.32 ~ -1.03) |
| Congo | 255.59(176.44~349.76) | 450.39(286.46~654.95) | 0.76 | 21.32(14.5~28.51) | 13.64(8.93~19.24) | -1.55 (-1.63 ~ -1.46) |
| Cook Islands | 0.08(0.05~0.12) | 0.07(0.04~0.11) | -0.12 | 0.55(0.36~0.83) | 0.32(0.19~0.49) | -1.84 (-2.14 ~ -1.55) |
| Costa Rica | 107.11(84.62~132.11) | 262.62(198.43~341.52) | 1.45 | 5.68(4.48~7.08) | 4.77(3.61~6.17) | -1.21 (-1.47 ~ -0.96) |
| Croatia | 309.4(221.45~437.97) | 177.13(124.83~247.78) | -0.43 | 4.93(3.56~6.85) | 2.25(1.6~3.11) | -2.62 (-2.82 ~ -2.41) |
| Cuba | 258.19(192.99~331.8) | 404.26(291.38~560.34) | 0.57 | 2.52(1.89~3.23) | 2.19(1.57~3) | -0.6 (-1.01 ~ -0.18) |
| Cyprus | 23.45(15.97~32.92) | 23.1(17.07~31.13) | -0.01 | 3.21(2.24~4.46) | 1.19(0.88~1.59) | -3.32 (-3.42 ~ -3.22) |
| Czechia | 304.89(222.47~417.46) | 310.08(215.26~435.23) | 0.02 | 2.33(1.69~3.22) | 1.73(1.19~2.42) | -0.68 (-0.85 ~ -0.51) |
| C么te d'Ivoire | 537.73(367.2~760.14) | 991.34(662.48~1451.2) | 0.84 | 11.81(8.3~16.59) | 7.49(5.15~10.54) | -1.43 (-1.55 ~ -1.31) |
| Democratic People's Republic of Korea | 625.25(391.6~974.68) | 856.34(519.55~1392.1) | 0.37 | 3.69(2.35~5.68) | 2.57(1.57~4.12) | -1.13 (-1.18 ~ -1.07) |
| Democratic Republic of the Congo | 2858.8(1963.86~3942.83) | 6051.6(3947.88~8793.73) | 1.12 | 16.03(11.11~22.04) | 13.36(8.82~19.44) | -0.46 (-0.53 ~ -0.39) |
| Denmark | 167.46(120.86~225.71) | 149.07(107.94~209.73) | -0.11 | 2.48(1.74~3.38) | 1.47(1.06~2.05) | -2.2 (-2.64 ~ -1.76) |
| Djibouti | 20.75(12.92~35.9) | 76.38(47.24~113.8) | 2.68 | 12.9(8.45~20.95) | 10.42(6.4~15.31) | -0.83 (-0.98 ~ -0.68) |
| Dominica | 2.43(1.82~3.21) | 2.03(1.4~2.87) | -0.16 | 4.21(3.16~5.59) | 2.45(1.7~3.43) | -1.88 (-2.12 ~ -1.65) |
| Dominican Republic | 331.83(239.54~431.98) | 564.02(333.07~810.63) | 0.7 | 8.66(6.42~11.1) | 5.52(3.27~8.03) | -1.37 (-1.54 ~ -1.2) |
| Ecuador | 148.37(105.59~205.97) | 254.44(165.66~387.88) | 0.71 | 2.59(1.85~3.57) | 1.56(1.03~2.34) | -1.34 (-1.59 ~ -1.08) |
| Egypt | 20498.69(16315.69~24146.05) | 23791.57(17694.2~30282.45) | 0.16 | 109.15(83.25~129.58) | 45.2(34.58~56.19) | -2.49 (-2.67 ~ -2.3) |
| El Salvador | 324.06(252.52~396.31) | 462.18(329.83~631.28) | 0.43 | 10.07(7.94~12.49) | 7.52(5.38~10.28) | -1.01 (-1.23 ~ -0.79) |
| Equatorial Guinea | 44.29(27.43~61.53) | 54.31(29.82~88.94) | 0.23 | 19.85(12.17~27.58) | 8.02(4.25~12.93) | -3.36 (-3.67 ~ -3.04) |
| Eritrea | 291.09(196.07~412.32) | 513.58(334.61~724.49) | 0.76 | 21.28(14.46~29.67) | 15.58(10.56~21.46) | -1.11 (-1.18 ~ -1.05) |
| Estonia | 38.63(28.34~51.86) | 65.71(47.12~90.6) | 0.7 | 1.99(1.46~2.66) | 3.3(2.27~4.58) | 1.07 (0.33 ~ 1.81) |
| Eswatini | 40.42(25.52~65.94) | 69.89(42.43~103.03) | 0.73 | 12.58(7.93~20.3) | 10.31(6.37~15) | -0.23 (-0.72 ~ 0.26) |
| Ethiopia | 4060.54(2592.28~5372) | 4798.88(3850.04~5811.11) | 0.18 | 18.72(12.16~24.56) | 9.85(7.81~12.1) | -2.52 (-2.7 ~ -2.34) |
| Fiji | 13.28(8.57~20.17) | 16.35(10.24~24.58) | 0.23 | 2.76(1.78~4.22) | 1.92(1.23~2.79) | -1.27 (-1.41 ~ -1.13) |
| Finland | 153.3(113.28~205.91) | 203.46(148.87~279.9) | 0.33 | 2.34(1.72~3.2) | 2.13(1.56~2.93) | 0.34 (-0.11 ~ 0.8) |
| France | 2680.46(1972.79~3587.09) | 1765.41(1294.36~2365.26) | -0.34 | 3.62(2.63~4.86) | 1.48(1.09~2) | -2.84 (-2.96 ~ -2.72) |
| Gabon | 117.56(82.81~157.2) | 144.93(91.96~207.21) | 0.23 | 19.83(14.01~26.39) | 12.2(7.75~17.25) | -1.48 (-1.61 ~ -1.36) |
| Gambia | 36.27(23.4~53.98) | 80.67(51.1~117.21) | 1.22 | 9.27(6.01~13.53) | 7.17(4.39~10.67) | -0.83 (-1.06 ~ -0.61) |
| Georgia | 382.55(281.7~512.75) | 262.04(185.31~360.85) | -0.32 | 6.16(4.59~8.22) | 4.95(3.47~6.77) | -0.1 (-0.42 ~ 0.23) |
| Germany | 4415.12(3297.54~5812.43) | 3245.28(2398.52~4477.84) | -0.26 | 3.86(2.81~5.14) | 2(1.49~2.77) | -2.48 (-2.63 ~ -2.33) |
| Ghana | 690.9(463.89~982.54) | 1400.47(951.08~1965.85) | 1.03 | 10.17(6.84~14.75) | 7.94(5.45~10.84) | -0.57 (-0.68 ~ -0.47) |
| Greece | 267.56(197.1~352.71) | 221.27(163.62~292.77) | -0.17 | 1.83(1.39~2.38) | 1.05(0.79~1.42) | -1.73 (-1.89 ~ -1.57) |
| Greenland | 2.76(2.12~3.47) | 3.33(2.33~4.4) | 0.21 | 5.78(4.51~7.25) | 4.43(3.21~5.76) | -0.53 (-0.64 ~ -0.43) |
| Grenada | 3.35(2.51~4.4) | 3.36(2.35~4.57) | 0 | 5.03(3.76~6.67) | 2.87(2.05~3.89) | -2 (-2.34 ~ -1.67) |
| Guam | 4.8(3.31~6.75) | 6.68(4.63~9.04) | 0.39 | 5.44(3.93~7.3) | 3.4(2.37~4.63) | -1.17 (-1.49 ~ -0.84) |
| Guatemala | 721.77(576.62~880.12) | 1707.44(1290.75~2182.43) | 1.37 | 17.41(14.24~21) | 14.26(10.77~18.18) | -0.9 (-1.17 ~ -0.63) |
| Guinea | 377.46(254.26~577.5) | 563.52(374.85~823.49) | 0.49 | 11.19(7.55~17.04) | 9.11(6.13~13.04) | -0.49 (-0.59 ~ -0.38) |
| Guinea-Bissau | 74.7(47.98~111.01) | 105.27(67.65~154.76) | 0.41 | 17.11(11.19~24.64) | 11.79(7.64~16.9) | -1.06 (-1.17 ~ -0.96) |
| Guyana | 49.35(36.14~67.4) | 50.82(33.9~74.22) | 0.03 | 11.42(8.58~15.35) | 7.29(4.98~10.49) | -0.99 (-1.27 ~ -0.71) |
| Haiti | 440.48(278.26~638.16) | 660.78(403.27~1039.57) | 0.5 | 12.55(8.21~18.16) | 8.2(5.08~12.82) | -1.33 (-1.38 ~ -1.28) |
| Honduras | 306.62(234.82~400.96) | 725.97(404.64~1168.83) | 1.37 | 13.06(10.02~16.92) | 10.8(5.8~17.63) | -0.62 (-0.72 ~ -0.53) |
| Hungary | 986.06(694.62~1373.97) | 510.39(352.39~739.52) | -0.48 | 7.3(5.04~10.23) | 3.08(2.13~4.45) | -4.25 (-4.71 ~ -3.79) |
| Iceland | 2.1(1.55~2.79) | 2.43(1.77~3.3) | 0.16 | 0.8(0.59~1.07) | 0.48(0.35~0.66) | -1.9 (-2.08 ~ -1.71) |
| India | 30506.67(23733.05~39624.52) | 54503.26(36577.34~72822.94) | 0.79 | 5.68(4.39~7.34) | 4.38(2.9~5.77) | -0.76 (-0.89 ~ -0.62) |
| Indonesia | 25233.88(18807.92~35690.56) | 39996.84(31856.71~49459.79) | 0.59 | 24.04(17.55~34.69) | 17.61(14.08~21.12) | -0.95 (-1.04 ~ -0.86) |
| Iran (Islamic Republic of) | 903.32(671.38~1273.28) | 1409.51(1151.58~1823.84) | 0.56 | 3.83(2.8~5.29) | 1.87(1.52~2.43) | -2.22 (-2.31 ~ -2.13) |
| Iraq | 657.26(460.12~947.29) | 1267.05(864.78~1763.68) | 0.93 | 8.11(5.57~12.1) | 5.58(3.89~7.65) | -1.56 (-1.68 ~ -1.44) |
| Ireland | 40.82(30.41~54.27) | 56.2(40.14~73.9) | 0.38 | 1.06(0.8~1.42) | 0.79(0.57~1.05) | -0.63 (-1.07 ~ -0.2) |
| Israel | 92.94(70.1~122.39) | 111.27(83.28~145.41) | 0.2 | 1.96(1.48~2.57) | 0.94(0.71~1.24) | -2.58 (-2.82 ~ -2.35) |
| Italy | 5290.79(4786.9~5828.01) | 2952.61(2556.07~3382.78) | -0.44 | 6.22(5.67~6.76) | 2.08(1.82~2.34) | -3.56 (-3.61 ~ -3.5) |
| Jamaica | 31.76(23.99~42.42) | 31.26(20.11~45.95) | -0.02 | 1.83(1.37~2.48) | 1.01(0.65~1.49) | -2.2 (-2.62 ~ -1.78) |
| Japan | 10056.83(9212.75~10820.61) | 8934.92(7488.71~10076.4) | -0.11 | 6(5.5~6.45) | 2.67(2.35~2.95) | -2.75 (-2.88 ~ -2.62) |
| Jordan | 102.29(74.4~142.85) | 213.99(155.68~283.42) | 1.09 | 8.43(6.01~11.95) | 3.2(2.23~4.24) | -3.8 (-4.23 ~ -3.38) |
| Kazakhstan | 534.34(399.53~723.26) | 1723.74(1241.61~2336.56) | 2.23 | 4.14(3.08~5.51) | 9.39(6.87~12.37) | 3.19 (2.61 ~ 3.79) |
| Kenya | 1417.06(887.06~2484.63) | 3928.64(2772.53~5797.54) | 1.77 | 16.29(10.14~28.78) | 16.08(11.37~23.64) | 0.23 (-0.02 ~ 0.47) |
| Kiribati | 3.43(2.19~5.24) | 5.52(3.4~8.48) | 0.61 | 7.37(4.62~11.2) | 6.06(3.8~9.22) | -0.59 (-0.67 ~ -0.5) |
| Kuwait | 25.05(19.49~30.96) | 68.69(51.58~87.43) | 1.74 | 3.97(3.03~5) | 2.58(1.86~3.38) | -1.19 (-2.12 ~ -0.25) |
| Kyrgyzstan | 296.43(223.69~390.33) | 515.09(357.77~710.94) | 0.74 | 9.63(7.29~12.54) | 9.15(6.49~12.4) | -0.21 (-0.9 ~ 0.49) |
| Lao People's Democratic Republic | 301.45(185.55~476.62) | 359.92(237.55~557.29) | 0.19 | 13.29(7.92~21.53) | 7.04(4.68~10.51) | -2.28 (-2.39 ~ -2.16) |
| Latvia | 62.19(46.14~83.15) | 95.09(67.98~130.91) | 0.53 | 1.83(1.36~2.42) | 3.34(2.37~4.68) | 1.34 (0.65 ~ 2.03) |
| Lebanon | 184.24(122.22~247.29) | 297.27(214.3~388.15) | 0.61 | 9.37(6.3~12.38) | 4.65(3.35~6.09) | -2 (-2.18 ~ -1.83) |
| Lesotho | 89.75(47.93~171.35) | 140.26(95.34~199.98) | 0.56 | 10.41(5.64~20.28) | 11.71(7.96~16.33) | 0.94 (0.52 ~ 1.36) |
| Liberia | 132.36(91.48~182.33) | 211.09(138.67~319.29) | 0.59 | 11.22(7.69~15.5) | 8.44(5.56~12.6) | -1.17 (-1.34 ~ -1.01) |
| Libya | 187.56(102.13~389.09) | 413.99(241.52~687.4) | 1.21 | 10.22(5.33~21.51) | 8.15(4.67~13.53) | -0.49 (-0.85 ~ -0.13) |
| Lithuania | 132.5(103.68~163.52) | 260.96(201.34~329.13) | 0.97 | 3.06(2.42~3.76) | 6.26(4.76~7.83) | 2.62 (1.53 ~ 3.72) |
| Luxembourg | 19.23(13.87~26.03) | 16(11.58~21.59) | -0.17 | 3.78(2.73~5.1) | 1.58(1.14~2.13) | -2.75 (-2.88 ~ -2.61) |
| Madagascar | 703.72(492.48~1034.97) | 1356.26(872.9~2063.01) | 0.93 | 12.93(9.07~18.81) | 10.33(6.74~15.6) | -0.73 (-0.79 ~ -0.67) |
| Malawi | 738.39(533.9~1019.31) | 1305.89(961.18~1716.28) | 0.77 | 17.47(13~23.41) | 15.13(11.05~19.83) | -0.71 (-0.98 ~ -0.43) |
| Malaysia | 407.27(297.03~550.81) | 1115.11(788.33~1495.66) | 1.74 | 4.02(2.95~5.4) | 3.9(2.8~5.18) | -0.75 (-1.04 ~ -0.47) |
| Maldives | 7.4(4.9~10.18) | 8.98(6.42~12.65) | 0.21 | 7.81(5.16~10.95) | 2.27(1.66~3.03) | -4.35 (-4.53 ~ -4.18) |
| Mali | 467.86(303.03~666.11) | 847.72(603.17~1206.06) | 0.81 | 11.24(7.34~16.15) | 8.7(5.92~12.22) | -0.52 (-0.7 ~ -0.35) |
| Malta | 7.71(5.77~10.29) | 6.99(5.06~9.26) | -0.09 | 1.81(1.36~2.41) | 0.89(0.66~1.19) | -2.22 (-2.34 ~ -2.09) |
| Marshall Islands | 1.75(1.16~2.67) | 2.56(1.48~4.06) | 0.46 | 8.22(5.31~12.65) | 5.68(3.4~8.93) | -1.22 (-1.25 ~ -1.18) |
| Mauritania | 114.78(73.55~168.46) | 142.16(89.19~223.65) | 0.24 | 11.3(7.17~16.58) | 6.33(4~9.68) | -1.92 (-2.06 ~ -1.78) |
| Mauritius | 64.6(48.09~85.14) | 59.97(43.99~78.92) | -0.07 | 8(5.99~10.46) | 3.41(2.51~4.43) | -3.95 (-4.3 ~ -3.61) |
| Mexico | 8242.67(7596.54~8898.38) | 13117.13(11117.46~15265.12) | 0.59 | 16.96(15.57~18.37) | 9.96(8.43~11.55) | -2.1 (-2.23 ~ -1.98) |
| Micronesia (Federated States of) | 4.13(2.64~6.01) | 4.16(2.43~6.47) | 0.01 | 7.08(4.44~10.44) | 4.69(2.83~7.27) | -1.35 (-1.46 ~ -1.23) |
| Monaco | 1.18(0.82~1.67) | 1.35(0.95~1.86) | 0.14 | 2.07(1.44~2.9) | 1.78(1.19~2.52) | -0.45 (-0.55 ~ -0.35) |
| Mongolia | 173.89(124.96~233.11) | 219.57(160.15~287.55) | 0.26 | 15.96(11.47~21.35) | 9.19(6.83~12.2) | -1.52 (-2.01 ~ -1.03) |
| Montenegro | 9.41(6.34~13.92) | 11.42(7.43~16.32) | 0.21 | 1.44(0.98~2.11) | 1.24(0.8~1.74) | -0.37 (-0.51 ~ -0.24) |
| Morocco | 1120.89(762.83~1611.65) | 2098.11(1396.1~2914.98) | 0.87 | 8.25(5.67~11.92) | 6.57(4.47~8.87) | -0.67 (-0.72 ~ -0.63) |
| Mozambique | 432.45(319.08~620.71) | 863.58(575.86~1211.47) | 1 | 7.37(5.38~10.37) | 7.02(4.7~9.92) | 0.21 (0.02 ~ 0.4) |
| Myanmar | 3454.51(1999.46~5678.19) | 3908.58(2528.41~5527.37) | 0.13 | 12.5(7.41~20.32) | 7.17(4.74~10.09) | -2.07 (-2.17 ~ -1.96) |
| Namibia | 75.91(44.74~138.18) | 127.77(84.22~197.48) | 0.68 | 11.14(6.64~20.17) | 8.42(5.69~12.64) | -1.04 (-1.33 ~ -0.76) |
| Nauru | 0.47(0.27~0.71) | 0.42(0.19~0.68) | -0.11 | 7.66(4.53~11.4) | 5.63(2.57~8.81) | -1.04 (-1.21 ~ -0.88) |
| Nepal | 356.65(194.98~617.73) | 629.97(405.29~932.57) | 0.77 | 3.24(1.76~5.74) | 2.62(1.7~3.87) | -0.64 (-0.73 ~ -0.55) |
| Netherlands | 219.85(163.02~285.23) | 229.34(170.29~304.16) | 0.04 | 1.17(0.87~1.54) | 0.72(0.53~0.95) | -1.81 (-2.01 ~ -1.6) |
| New Zealand | 47.55(42.76~52.65) | 71.62(62.74~80.13) | 0.51 | 1.25(1.13~1.38) | 0.9(0.8~1) | -0.91 (-1.14 ~ -0.69) |
| Nicaragua | 151.26(117.39~188.84) | 386.16(285.33~510.95) | 1.55 | 8.65(6.71~10.8) | 7.19(5.33~9.56) | -0.3 (-0.51 ~ -0.1) |
| Niger | 426.59(277.76~714.03) | 926.19(544.83~1645.93) | 1.17 | 14.08(9.33~22.77) | 10.38(6.35~17.93) | -0.81 (-0.89 ~ -0.73) |
| Nigeria | 5615.09(3968.34~7999.69) | 6973.39(4745.07~9656.68) | 0.24 | 12.24(8.66~17.28) | 7.01(5.05~9.33) | -1.78 (-1.97 ~ -1.58) |
| Niue | 0.09(0.06~0.14) | 0.07(0.04~0.1) | -0.22 | 4.45(2.97~6.63) | 3.19(2.04~4.96) | -1.33 (-1.43 ~ -1.23) |
| North Macedonia | 37.6(26.04~51.92) | 46.89(30.68~67.42) | 0.25 | 1.89(1.32~2.57) | 1.42(0.94~2.03) | -0.95 (-1.2 ~ -0.69) |
| Northern Mariana Islands | 1.84(1.16~2.83) | 2.44(1.71~3.38) | 0.33 | 6.81(4.69~9.85) | 4.5(3.31~6.02) | -1.43 (-1.72 ~ -1.13) |
| Norway | 58.02(50.24~67.14) | 57.24(49.39~66.64) | -0.01 | 1.02(0.89~1.18) | 0.64(0.55~0.74) | -1.58 (-1.8 ~ -1.35) |
| Oman | 80.1(52.19~125.4) | 105.25(72.85~152.59) | 0.31 | 12.29(8.09~19.31) | 5.77(4.03~7.61) | -1.91 (-2.09 ~ -1.74) |
| Pakistan | 8853.54(4548.78~16650.05) | 16671.31(11975.12~22450.4) | 0.88 | 14.47(7.49~27.8) | 11.91(8.59~15.68) | -0.87 (-1.05 ~ -0.69) |
| Palau | 0.53(0.31~0.84) | 0.78(0.47~1.17) | 0.47 | 4.64(2.8~7.09) | 3.31(2.07~4.83) | -1 (-1.07 ~ -0.93) |
| Palestine | 103.04(60.91~178.09) | 131.13(97.11~167.06) | 0.27 | 13.26(7.9~23.11) | 6.09(4.53~7.72) | -2.47 (-2.67 ~ -2.28) |
| Panama | 73.58(58.01~89.15) | 134.66(96.01~179.36) | 0.83 | 4.76(3.75~5.75) | 3.05(2.17~4.06) | -1.42 (-1.53 ~ -1.31) |
| Papua New Guinea | 60.66(33.47~99.63) | 124.97(78.47~181.16) | 1.06 | 2.58(1.46~4.23) | 1.86(1.22~2.74) | -1.14 (-1.28 ~ -1) |
| Paraguay | 73.74(56.53~95.62) | 201.95(134.37~284.98) | 1.74 | 3.04(2.31~3.94) | 3.27(2.2~4.62) | 0.47 (0.34 ~ 0.59) |
| Peru | 364.5(248.94~526.13) | 600.47(380.6~873.28) | 0.65 | 2.82(1.88~4.11) | 1.76(1.1~2.57) | -1.97 (-2.28 ~ -1.66) |
| Philippines | 1929.82(1429~2823.63) | 4034.77(3218.72~5072.39) | 1.09 | 5.64(4.14~8.55) | 4.53(3.63~5.62) | -0.64 (-0.68 ~ -0.59) |
| Poland | 1350.81(1194.37~1546.5) | 2247.91(1921.97~2607.5) | 0.66 | 3.12(2.77~3.56) | 3.79(3.22~4.44) | 0.45 (0.2 ~ 0.7) |
| Portugal | 697.82(523.52~931.38) | 334.54(247.17~448.29) | -0.52 | 5.4(4.05~7.2) | 1.63(1.2~2.17) | -4.07 (-4.18 ~ -3.95) |
| Puerto Rico | 219.88(168.12~291) | 134.97(92.38~181.55) | -0.39 | 6.18(4.74~8.18) | 2.15(1.52~2.92) | -3.92 (-4.31 ~ -3.52) |
| Qatar | 16.05(11.79~21.94) | 56.32(38.87~82.28) | 2.51 | 18.45(12.99~25.25) | 7.39(5.1~10.1) | -3.18 (-3.97 ~ -2.38) |
| Republic of Korea | 2143.32(1482.42~3073.46) | 1198.54(850.2~1718.68) | -0.44 | 6.63(4.7~9.07) | 1.35(0.96~1.95) | -5.53 (-5.73 ~ -5.33) |
| Republic of Moldova | 807.85(592.39~1105.25) | 642.3(464.6~881.25) | -0.2 | 17.65(13.19~23.82) | 11.47(8.31~15.61) | -1.43 (-1.91 ~ -0.94) |
| Romania | 1624.53(1178.85~2227.94) | 1889.06(1346.24~2694.38) | 0.16 | 5.8(4.24~7.9) | 5.78(4.07~8.25) | -0.93 (-1.33 ~ -0.53) |
| Russian Federation | 2317.94(1951.01~2774.59) | 7026.01(5759.94~8543.15) | 2.03 | 1.29(1.1~1.53) | 3.4(2.73~4.18) | 3.02 (2.13 ~ 3.93) |
| Rwanda | 720.49(515.7~962.57) | 974.35(669.2~1429.41) | 0.35 | 23.29(16.98~30.84) | 14.18(9.89~20.14) | -2.41 (-2.71 ~ -2.11) |
| Saint Kitts and Nevis | 2.38(1.78~3.13) | 2.43(1.59~3.53) | 0.02 | 6.96(5.17~9.12) | 3.34(2.34~4.7) | -2.17 (-2.71 ~ -1.63) |
| Saint Lucia | 5.98(4.47~7.73) | 7.07(5.04~9.87) | 0.18 | 6.99(5.3~9.02) | 2.99(2.14~4.09) | -3.04 (-3.62 ~ -2.46) |
| Saint Vincent and the Grenadines | 2.91(2.19~3.81) | 4.07(2.96~5.55) | 0.4 | 4.1(3.08~5.4) | 2.9(2.12~3.88) | -1.33 (-1.86 ~ -0.8) |
| Samoa | 3.93(2.51~6.11) | 5.32(3.23~7.87) | 0.35 | 4.05(2.61~6.31) | 3.34(2.08~4.81) | -0.67 (-0.89 ~ -0.45) |
| San Marino | 1.06(0.74~1.45) | 1.01(0.61~1.51) | -0.05 | 3.14(2.22~4.32) | 1.4(0.81~2.14) | -1.76 (-2.06 ~ -1.46) |
| Sao Tome and Principe | 11.88(8.23~16.6) | 15.27(8.84~23.2) | 0.29 | 18.35(12.84~25.7) | 12.65(7.29~19.11) | -1.31 (-1.55 ~ -1.08) |
| Saudi Arabia | 1749.3(994.92~3341.9) | 2774.7(2038.12~3649.42) | 0.59 | 32.71(17.9~64.79) | 15.61(11.51~20.43) | -2.17 (-2.29 ~ -2.05) |
| Senegal | 327.61(225.45~481.43) | 554.34(336.8~812.97) | 0.69 | 9.54(6.54~13.86) | 6.6(3.96~9.57) | -1.05 (-1.23 ~ -0.87) |
| Serbia | 291.91(199.74~425.04) | 256.97(176.76~363.49) | -0.12 | 2.53(1.79~3.49) | 1.72(1.2~2.41) | -1.39 (-1.59 ~ -1.18) |
| Seychelles | 5.49(4.12~7.02) | 7.4(5.25~10.06) | 0.35 | 9.78(7.34~12.55) | 6.16(4.45~8.24) | -1.75 (-1.99 ~ -1.52) |
| Sierra Leone | 224.25(148.69~331.46) | 329.13(222~482.97) | 0.47 | 10.6(7.05~15.17) | 7.71(5.32~10.89) | -0.81 (-0.95 ~ -0.67) |
| Singapore | 61.09(45.71~77.57) | 37.79(27.92~50.13) | -0.38 | 2.5(1.91~3.19) | 0.44(0.33~0.58) | -5.25 (-5.51 ~ -4.99) |
| Slovakia | 307.37(220.28~422.21) | 273.61(188.31~379.17) | -0.11 | 5.3(3.79~7.35) | 3.17(2.15~4.4) | -1.27 (-1.49 ~ -1.06) |
| Slovenia | 124.94(87.34~178.28) | 72.16(50.13~100.31) | -0.42 | 5.13(3.59~7.3) | 1.79(1.26~2.49) | -3.77 (-4.23 ~ -3.31) |
| Solomon Islands | 12.07(6.69~20.8) | 30.85(19.4~46.45) | 1.56 | 7.15(4.19~11.95) | 6.94(4.58~10.27) | -0.01 (-0.1 ~ 0.08) |
| Somalia | 504.11(314.9~750.93) | 1109.79(663.73~1705.04) | 1.2 | 17.37(11.43~25.11) | 14.55(8.97~21.77) | -0.68 (-0.76 ~ -0.6) |
| South Africa | 1373.77(1113.24~1772.76) | 2180.11(1898.87~2513.36) | 0.59 | 5.76(4.55~7.65) | 4.41(3.82~5.05) | -1 (-1.47 ~ -0.53) |
| South Sudan | 423.91(284.41~657.69) | 645.3(420.95~934.26) | 0.52 | 15.69(10.61~23.71) | 14.66(9.76~20.99) | -0.41 (-0.54 ~ -0.28) |
| Spain | 2538.05(1948.62~3256.92) | 1398.15(1041.47~1776.06) | -0.45 | 5(3.86~6.34) | 1.55(1.15~2.01) | -3.69 (-3.78 ~ -3.61) |
| Sri Lanka | 1297.88(880.77~1905.65) | 957.54(585.55~1469.28) | -0.26 | 10.78(7.52~15.26) | 3.67(2.26~5.54) | -3.91 (-4.26 ~ -3.56) |
| Sudan | 1229.28(685.35~2281.11) | 1638.56(943.88~3002.91) | 0.33 | 14.1(7.77~27.16) | 8.7(5.12~16.33) | -1.6 (-1.63 ~ -1.57) |
| Suriname | 18.03(13.15~24.5) | 24.77(16.16~36.44) | 0.37 | 6.65(4.85~8.97) | 3.84(2.51~5.62) | -1.81 (-1.98 ~ -1.64) |
| Sweden | 168.08(145.28~194.39) | 178.6(145.56~215.66) | 0.06 | 1.33(1.14~1.55) | 0.93(0.77~1.12) | -0.47 (-0.7 ~ -0.24) |
| Switzerland | 164.52(120.57~217.41) | 143.94(103.68~192.66) | -0.13 | 1.77(1.28~2.39) | 0.88(0.63~1.18) | -2.35 (-2.63 ~ -2.06) |
| Syrian Arab Republic | 519.22(357.83~733.45) | 854.61(578.42~1210.12) | 0.65 | 10.6(7.07~15.37) | 7.67(5.19~10.63) | -1.09 (-1.21 ~ -0.96) |
| Taiwan (Province of China) | 1268.59(961.77~1651.56) | 1453.98(1071.64~1911.04) | 0.15 | 8.04(6.25~10.33) | 3.68(2.7~4.84) | -2.66 (-2.88 ~ -2.45) |
| Tajikistan | 271.24(197.78~352.82) | 439.35(305.16~595.58) | 0.62 | 9.22(6.81~11.95) | 6.52(4.63~8.79) | -1.54 (-1.92 ~ -1.16) |
| Thailand | 2456.59(1693.59~3498.46) | 5028.06(3326.11~7045.73) | 1.05 | 6.14(4.35~8.65) | 4.97(3.3~7.04) | -0.65 (-0.84 ~ -0.46) |
| Timor-Leste | 26.89(13.76~54.41) | 52.49(31.32~90.52) | 0.95 | 7.6(3.86~15.39) | 5.99(3.53~10.22) | -0.84 (-1.04 ~ -0.64) |
| Togo | 133.57(87.49~194.77) | 336.88(211.69~520.77) | 1.52 | 9.61(6.41~13.92) | 7.63(4.8~11.52) | -0.63 (-0.75 ~ -0.52) |
| Tokelau | 0.07(0.04~0.11) | 0.05(0.03~0.06) | -0.29 | 5.25(3.25~7.87) | 3.17(2.19~4.48) | -1.88 (-2 ~ -1.76) |
| Tonga | 4.46(2.94~6.61) | 4.49(2.79~6.87) | 0.01 | 7.45(4.92~11.05) | 5.36(3.34~8.17) | -0.96 (-1 ~ -0.91) |
| Trinidad and Tobago | 34.1(25.44~45.24) | 31.84(20.99~46.8) | -0.07 | 3.93(2.96~5.18) | 1.7(1.12~2.46) | -2.89 (-3.16 ~ -2.62) |
| Tunisia | 354.21(240.87~521.47) | 680.28(467.8~981.91) | 0.92 | 8.02(5.44~11.73) | 5.46(3.74~7.78) | -1.21 (-1.28 ~ -1.14) |
| Turkmenistan | 226.59(170.95~291.44) | 692.87(468.32~990.18) | 2.06 | 11.01(8.44~14.08) | 14.72(10.13~20.75) | 1 (0.75 ~ 1.26) |
| Tuvalu | 0.51(0.32~0.76) | 0.49(0.33~0.69) | -0.04 | 6.8(4.29~10.13) | 4.43(3.05~6.17) | -1.3 (-1.43 ~ -1.18) |
| T眉rkiye | 1604.55(1090.72~2246.25) | 2136.56(1458.81~2927.77) | 0.33 | 4.76(3.23~6.65) | 2.38(1.64~3.26) | -2.17 (-2.46 ~ -1.87) |
| Uganda | 881.69(620.4~1248.26) | 1665.53(1104.16~2397.01) | 0.89 | 13.01(9.24~18.07) | 9.81(6.35~14.46) | -1.4 (-1.62 ~ -1.17) |
| Ukraine | 1842.24(1558.18~2157.69) | 3670.44(2517.23~5050.56) | 0.99 | 2.65(2.26~3.08) | 5.84(4.04~8.15) | 2.31 (1.32 ~ 3.31) |
| United Arab Emirates | 40.91(28.75~60.42) | 151.23(104.26~220.54) | 2.7 | 9.71(6.9~13.77) | 5.76(3.74~8.92) | -0.09 (-0.63 ~ 0.46) |
| United Kingdom | 356.79(288.44~445.05) | 721.61(572.26~911.94) | 1.02 | 0.48(0.38~0.6) | 0.7(0.55~0.9) | 1.21 (0.59 ~ 1.83) |
| United Republic of Tanzania | 1422.23(1015.73~1879.19) | 2803.79(1945.92~3842.13) | 0.97 | 12.48(9.05~16.11) | 9.97(6.99~13.55) | -0.7 (-0.89 ~ -0.51) |
| United States Virgin Islands | 4.68(3.28~6.47) | 2.99(1.99~4.3) | -0.36 | 5.18(3.75~7.14) | 2.04(1.39~2.9) | -2.89 (-3.11 ~ -2.68) |
| United States of America | 13858.67(12668.76~15270.6) | 25280.56(22455.77~28319.96) | 0.82 | 4.67(4.26~5.14) | 4.94(4.43~5.48) | 0.31 (0.16 ~ 0.45) |
| Uruguay | 177.65(143.25~218.15) | 139.76(110~172.46) | -0.21 | 4.76(3.84~5.81) | 2.78(2.22~3.44) | -1.94 (-2.1 ~ -1.79) |
| Uzbekistan | 1155.97(868.1~1500.33) | 2311.17(1638.52~3130.23) | 1 | 9.41(7.05~12.06) | 7.54(5.59~10.01) | -1.08 (-1.51 ~ -0.66) |
| Vanuatu | 6.69(3.36~13.09) | 13.69(7.91~23.39) | 1.05 | 8.15(4~16.12) | 6.22(3.63~10.56) | -1 (-1.09 ~ -0.91) |
| Venezuela (Bolivarian Republic of) | 722.94(563.86~902.34) | 1366.04(916.66~2002.35) | 0.89 | 6.78(5.28~8.49) | 4.44(3~6.45) | -1.5 (-1.77 ~ -1.23) |
| Viet Nam | 4239.99(2678.06~6913.21) | 6417.13(4388.98~9033.44) | 0.51 | 10.65(6.68~17.4) | 6.46(4.45~8.82) | -1.63 (-1.69 ~ -1.57) |
| Yemen | 750.19(408.19~1398.3) | 1302.02(849.25~1892.87) | 0.74 | 16.82(9.05~32.87) | 10(6.51~15.11) | -2.07 (-2.23 ~ -1.91) |
| Zambia | 565.72(407.07~784.91) | 1108.69(655.18~1607.84) | 0.96 | 18.11(12.98~24.89) | 13.25(8.01~18.73) | -1.32 (-1.62 ~ -1.02) |
| Zimbabwe | 379.85(270.42~538.54) | 803.88(394~1232.17) | 1.12 | 9.16(6.65~12.74) | 10.29(5.25~15.59) | 0.74 (0.27 ~ 1.23) |
