# Supplementary material for: Global, regional, and national burden of chronic hepatitis C including cirrhosis from 1990 to 2021, alongside projections for the next 15 years
Source: Medicine (Baltimore). 2026 Oct 16;105(42):e51118. doi: 10.1097/MD.0000000000051118 (PMC13681144; doi:10.1097/MD.0000000000051118)
Supplement: Supplementary file 10 [file medi-105-e51118-s010.docx]

Supplementary Table S7. The number of DALYs cases and the age-standardized DALYs rate of chronic hepatitis C including cirrhosis in 1990 and 2021 at 204 countries and territories levels, and their trends from 1990 to 2021.

| location | Number of DALYs cases (95%UI) in 1990 | Number of DALYs cases (95%UI) in 2021 | 1990-2021 Percentage change(100%) | The age-standardized DALYs rate/100000 (95%UI) in 1990 | The age-standardized DALYs rate/100000 (95%UI) in 2021 | 1990-2021 EAPC (95%CI) |
| --- | --- | --- | --- | --- | --- | --- |
| Afghanistan | 43346.85(26040.42~64688.25) | 48537.07(27301.9~77671.45) | 0.12 | 608.94(377.58~896.53) | 386.49(225.2~584.22) | -1.72 (-1.9 ~ -1.53) |
| Albania | 1780.55(1221.77~2460.13) | 1385.89(915.87~2054.25) | -0.22 | 75.28(52.48~102.44) | 36.66(24.17~53.48) | -2.1 (-2.27 ~ -1.92) |
| Algeria | 22918.06(15927.36~33433) | 41732.06(29949.95~59221.66) | 0.82 | 178.62(128.14~247.53) | 114.43(84.35~158.18) | -1.38 (-1.42 ~ -1.34) |
| American Samoa | 38.79(24.96~55.72) | 48.69(31.36~70.28) | 0.26 | 117.22(76.63~167.67) | 93.05(61.76~132.62) | -0.8 (-1.06 ~ -0.55) |
| Andorra | 51.16(31.01~80.51) | 59.68(35.95~91.68) | 0.17 | 84.44(51.2~132.86) | 42.02(25.5~63.32) | -2.01 (-2.19 ~ -1.82) |
| Angola | 42108.95(29119.63~57937.66) | 80596.62(56158.16~112056.17) | 0.91 | 759.29(517.11~1042.37) | 459.02(325.46~636.08) | -1.57 (-1.66 ~ -1.48) |
| Antigua and Barbuda | 63.91(47.19~84.69) | 75.11(55.02~100.83) | 0.18 | 126.07(91.86~167.87) | 67.2(49.99~88.71) | -2.07 (-2.52 ~ -1.62) |
| Argentina | 66165.41(52564.09~80868.71) | 63878.95(51127.18~79746.32) | -0.03 | 204.79(163.48~249.56) | 120.68(96.9~150.7) | -1.15 (-1.42 ~ -0.88) |
| Armenia | 3740.43(2723.5~5058.09) | 4002.89(2896.26~5472.3) | 0.07 | 122.05(89.21~163.58) | 101.01(72.4~138.53) | 0.01 (-0.78 ~ 0.81) |
| Australia | 13485.23(10993.3~16202.24) | 18471.93(15029.54~22466.55) | 0.37 | 71.97(58.28~86.63) | 50.85(41.67~61.13) | -0.74 (-0.94 ~ -0.55) |
| Austria | 15022.23(10721.61~20840.56) | 7957.13(5766.04~10915.47) | -0.47 | 153.78(106.38~211.5) | 54.97(39.69~74.15) | -3.35 (-3.5 ~ -3.2) |
| Azerbaijan | 14021.65(10066.46~19166.53) | 19326.46(12823.74~27891.65) | 0.38 | 249.02(179.7~335.3) | 171.83(115.17~246.42) | -1.61 (-1.98 ~ -1.25) |
| Bahamas | 351.42(245.82~470.21) | 458.55(298.57~692.89) | 0.3 | 185.75(129.02~253.63) | 102.09(67.78~153.15) | -2.05 (-2.43 ~ -1.66) |
| Bahrain | 624.27(474.79~778.65) | 1628.16(1219.39~2226.27) | 1.61 | 333.97(249.68~412.29) | 164.83(123.54~215.8) | -3.09 (-3.52 ~ -2.65) |
| Bangladesh | 231481.98(166987.44~315578.93) | 237061.74(164605.68~327383.57) | 0.02 | 372.27(265.63~507.41) | 158.4(111.46~216.26) | -2.88 (-3.02 ~ -2.75) |
| Barbados | 234.72(170.66~317.89) | 180.28(116.57~269.68) | -0.23 | 93.68(66.4~129.06) | 39.96(26.21~60.09) | -2.96 (-3.22 ~ -2.7) |
| Belarus | 6363.86(4546.62~8647.58) | 16733.01(11241.58~23572.94) | 1.63 | 50.79(36.54~68.71) | 127.07(84.22~178.83) | 3.23 (1.84 ~ 4.65) |
| Belgium | 10722.77(8090.85~14243.07) | 9288.69(6845.65~12552.22) | -0.13 | 82.52(60.8~110.33) | 51.94(37.68~69.51) | -1.65 (-1.82 ~ -1.47) |
| Belize | 150.56(111.69~200.43) | 506.15(354.74~689.59) | 2.36 | 144.78(108.1~193.31) | 139.22(99.19~188.99) | -0.36 (-0.68 ~ -0.03) |
| Benin | 8538.99(5778.21~12546.71) | 16708.86(10701.39~25337.39) | 0.96 | 355.52(241.44~521.95) | 233.14(152.88~338.65) | -1.32 (-1.41 ~ -1.22) |
| Bermuda | 57.73(41.83~78.33) | 35.61(24.55~50.24) | -0.38 | 88.13(64.73~119.62) | 32.58(22.44~45.86) | -3.3 (-3.92 ~ -2.68) |
| Bhutan | 1023.86(557.93~1865.54) | 1365.97(831.58~2378.91) | 0.33 | 278.65(153.58~486.63) | 190.64(119.16~330.36) | -1.29 (-1.32 ~ -1.26) |
| Bolivia (Plurinational State of) | 4344.86(2251.82~7254.95) | 6944.18(3946.16~11548.01) | 0.6 | 113.3(58.07~188.34) | 69.07(39.16~114.89) | -1.79 (-1.89 ~ -1.69) |
| Bosnia and Herzegovina | 4975.2(3404.31~7156.26) | 2927.02(1795.63~4190.33) | -0.41 | 105.86(73.27~149.19) | 54.69(33.73~78.6) | -2.26 (-2.47 ~ -2.05) |
| Botswana | 2470.99(1346.14~4581.18) | 3217.78(2167.78~4731.69) | 0.3 | 346.41(194.35~617.02) | 163.51(113.81~231.13) | -2.59 (-2.86 ~ -2.31) |
| Brazil | 242198.55(220738.32~264202.91) | 281543.68(251531.56~310889.85) | 0.16 | 209.46(190.57~227.78) | 109.39(98.05~120.56) | -2.06 (-2.23 ~ -1.9) |
| Brunei Darussalam | 155.38(110.31~209.3) | 298.68(207.61~405.77) | 0.92 | 103.21(73.38~140.04) | 63.64(45.19~85.28) | -1.28 (-1.53 ~ -1.04) |
| Bulgaria | 11523.53(8114.43~16279.72) | 14450.63(9771.59~20198.84) | 0.25 | 99.24(70.35~140.29) | 136.09(90.81~190.16) | 0.94 (0.66 ~ 1.22) |
| Burkina Faso | 12740.2(8538.55~17832.92) | 23573.97(15666.52~34176.93) | 0.85 | 256.36(176.76~353.9) | 200.5(134.94~289.3) | -0.64 (-0.75 ~ -0.53) |
| Burundi | 16721.1(10976.01~23754.59) | 26270.44(17806.72~36878.98) | 0.57 | 580.04(381.12~827.95) | 376.99(257.12~522.62) | -1.82 (-1.98 ~ -1.67) |
| Cabo Verde | 508.47(338.99~812.61) | 838.98(549.32~1235.56) | 0.65 | 230.23(153.59~371.16) | 161.48(104.75~236.43) | -1.53 (-1.69 ~ -1.37) |
| Cambodia | 48117.7(31469.66~74770.24) | 69895.56(45382.85~99912.22) | 0.45 | 818.04(531.29~1323.94) | 470.11(305.18~670.15) | -2.01 (-2.09 ~ -1.93) |
| Cameroon | 16497.17(10424.61~23865.8) | 39420.19(23112.98~61121.37) | 1.39 | 293.69(185.82~419.49) | 216.2(128.57~332.41) | -0.85 (-1 ~ -0.7) |
| Canada | 39109.91(31704.76~46689.59) | 60125.6(49898.99~72995.36) | 0.54 | 124.98(100.96~148.61) | 104.76(87.38~126.61) | -0.42 (-0.58 ~ -0.26) |
| Central African Republic | 12561.85(8686.56~17600.19) | 24281.01(16009.19~35152.9) | 0.93 | 795.99(556.39~1116.36) | 716.94(489.41~1017.67) | -0.35 (-0.43 ~ -0.26) |
| Chad | 11860.22(7824.69~18442.25) | 29481.33(18177.25~45365.08) | 1.49 | 365.96(239.24~575.3) | 367.63(230.04~570.93) | 0.05 (-0.09 ~ 0.19) |
| Chile | 49527.88(39246.56~60774.5) | 43878.92(34365.03~54552.89) | -0.11 | 446.64(354.08~547.38) | 180.55(142.08~223.58) | -2.27 (-2.49 ~ -2.04) |
| China | 713821.66(566034.59~892770.11) | 481674.14(373067.68~629745.52) | -0.33 | 72.99(58.33~90.49) | 23.73(18.27~30.96) | -3.61 (-3.75 ~ -3.46) |
| Colombia | 19222.64(15192.07~23836.86) | 28904.52(21198.36~37751.33) | 0.5 | 89.47(70.51~111.33) | 52.54(38.78~68.24) | -2.24 (-2.39 ~ -2.08) |
| Comoros | 994.07(637.77~1529.59) | 1732.29(1204.11~2440.87) | 0.74 | 403.67(268.32~614.96) | 293.93(206.16~408.99) | -1.28 (-1.48 ~ -1.08) |
| Congo | 9412.28(6569.65~13027.06) | 17029.3(10748.76~25531.16) | 0.81 | 688.53(476.47~942.04) | 430.68(275.82~621.59) | -1.64 (-1.74 ~ -1.53) |
| Cook Islands | 3.32(2.07~5.04) | 2.68(1.6~4.12) | -0.19 | 21.34(13.42~32.37) | 12.5(7.36~19.56) | -1.76 (-2.07 ~ -1.46) |
| Costa Rica | 3614.93(2803.07~4471.96) | 7862.06(5872.08~10352.93) | 1.17 | 176.35(136.16~218.93) | 143.11(107.02~186.43) | -1.37 (-1.62 ~ -1.12) |
| Croatia | 10096.11(6939.99~14389.36) | 4769.53(3350.09~6630.92) | -0.53 | 160.98(110.74~228.01) | 67.76(47.25~95.36) | -2.92 (-3.14 ~ -2.69) |
| Cuba | 7478.49(5551.19~9822.26) | 12149.6(8390.38~17099.13) | 0.62 | 71.41(52.68~94.31) | 69.7(48.3~97.27) | -0.23 (-0.62 ~ 0.17) |
| Cyprus | 670.47(449.55~971.87) | 644.36(458.31~870.12) | -0.04 | 85.41(58.54~122.89) | 34.13(24.15~46.42) | -3.06 (-3.15 ~ -2.97) |
| Czechia | 9955.25(6920.35~14235.09) | 9312.9(6413.5~13268.92) | -0.06 | 78.8(54.22~111.34) | 56.9(38.24~82.05) | -0.8 (-0.99 ~ -0.62) |
| Côte d'Ivoire | 19960.61(13335.76~28827.6) | 37273.17(23660.88~54986.04) | 0.87 | 342.89(236.01~483.43) | 224.32(149.5~324.93) | -1.34 (-1.46 ~ -1.22) |
| Democratic People's Republic of Korea | 21383.06(13328.24~34370.64) | 27506.56(15383.16~46019.82) | 0.29 | 112.78(71.08~176.95) | 81(46.97~134.42) | -1.07 (-1.1 ~ -1.04) |
| Democratic Republic of the Congo | 105200.84(72839.62~145744.9) | 231244(147376.5~334484.77) | 1.2 | 504.36(349.95~693.9) | 430.23(281.35~624.48) | -0.37 (-0.45 ~ -0.3) |
| Denmark | 5683.05(3935.08~7805.74) | 4073.21(2922.95~5740.67) | -0.28 | 88.78(61.6~123.14) | 44.76(31.99~61.99) | -2.75 (-3.2 ~ -2.3) |
| Djibouti | 778.23(475.89~1408.05) | 2786.04(1652.85~4230.44) | 2.58 | 377.78(238.84~637.13) | 302.57(187.51~449.9) | -0.87 (-1.04 ~ -0.69) |
| Dominica | 67.34(49.97~88.96) | 59.81(39.28~86.74) | -0.11 | 118.28(86.84~157.86) | 72.2(48.23~102.87) | -1.68 (-1.93 ~ -1.44) |
| Dominican Republic | 11035.73(7848.36~14785.88) | 17225.97(9943.06~25679.05) | 0.56 | 245.04(175.68~324.79) | 161.63(93.47~239.87) | -1.24 (-1.47 ~ -1.01) |
| Ecuador | 5057.41(3398.67~7419.2) | 7176.01(4559.59~11021.95) | 0.42 | 78.61(53.69~113.02) | 42.37(27.06~65.52) | -1.87 (-2.12 ~ -1.63) |
| Egypt | 514454.54(423347.2~596213.05) | 632392.94(469249.02~808372.32) | 0.23 | 2043.51(1630.51~2397.35) | 976.16(737.55~1228.58) | -2 (-2.23 ~ -1.77) |
| El Salvador | 11564.96(8700.7~14366.66) | 15151.52(10461.97~20998.92) | 0.31 | 334.34(252.26~415.65) | 248.3(171.52~345.37) | -1.04 (-1.28 ~ -0.8) |
| Equatorial Guinea | 1620.47(995.08~2289.46) | 2168.61(1210.4~3541.87) | 0.34 | 651.34(398.97~906.2) | 255.75(140.07~411.05) | -3.47 (-3.81 ~ -3.13) |
| Eritrea | 11189.69(7422.43~15759.51) | 19595.62(12671.92~28475.83) | 0.75 | 657.13(454.86~914.56) | 470.78(310.56~661.68) | -1.19 (-1.26 ~ -1.12) |
| Estonia | 1361.49(971.08~1874.09) | 2176.35(1474.57~3031.9) | 0.6 | 72.54(51.96~100.5) | 120.07(80.87~168.05) | 1.01 (0.24 ~ 1.79) |
| Eswatini | 1453.81(906.59~2414.95) | 2679.61(1579.71~4030.72) | 0.84 | 376.83(236.69~615.42) | 335.35(204.68~498.7) | 0.01 (-0.53 ~ 0.56) |
| Ethiopia | 142788.66(89787.96~190652.22) | 168457.09(134753.41~205675.99) | 0.18 | 558.94(356.74~738.9) | 282.9(225.76~341.98) | -2.66 (-2.85 ~ -2.48) |
| Fiji | 547.86(348.66~833.75) | 606.69(389.22~902.28) | 0.11 | 98.62(63.98~149.68) | 66.31(42.4~97.73) | -1.34 (-1.49 ~ -1.2) |
| Finland | 5217.43(3680.76~7358.51) | 5829.29(4260.21~8085.6) | 0.12 | 82.53(57.9~116.35) | 69.26(48.92~96.38) | 0.13 (-0.37 ~ 0.64) |
| France | 81717.78(58601.41~111222.06) | 46248.96(33649.09~62703.5) | -0.43 | 117.4(82.66~161.85) | 45.21(32.38~61.72) | -3.07 (-3.2 ~ -2.93) |
| Gabon | 3972.05(2800.88~5296.8) | 5176.18(3238.43~7587.74) | 0.3 | 622.33(435.63~832.19) | 381.85(250.15~556.43) | -1.51 (-1.63 ~ -1.4) |
| Gambia | 1296.01(824.86~1965.52) | 2940.79(1835.88~4279.29) | 1.27 | 273.29(175.17~408.12) | 217.37(137.43~319.71) | -0.77 (-1.01 ~ -0.53) |
| Georgia | 12121.11(8863.11~16507.18) | 8059.85(5489.37~11097.58) | -0.34 | 196.25(143.16~266.29) | 165.41(111.71~227.54) | 0.12 (-0.24 ~ 0.48) |
| Germany | 136562.05(96895.03~185419.96) | 86445.24(63530.04~120806.35) | -0.37 | 128.14(90.69~175.42) | 61.57(43.8~84.65) | -2.83 (-2.99 ~ -2.66) |
| Ghana | 24890.45(16052.43~35729.3) | 47873.32(31089.03~68804.57) | 0.92 | 298.32(197.32~421.22) | 219.77(150.55~311.21) | -0.78 (-0.9 ~ -0.67) |
| Greece | 6756.82(5050.48~9150.12) | 5384.97(4049.43~7266.78) | -0.2 | 48.52(36.28~64.54) | 31.07(22.6~42.74) | -1.24 (-1.42 ~ -1.07) |
| Greenland | 111.86(84.46~140.81) | 118.88(82.82~157.82) | 0.06 | 213.58(163.91~267.21) | 161.55(114.61~218.04) | -0.63 (-0.72 ~ -0.54) |
| Grenada | 103.14(76.12~134.93) | 104.99(71.54~141.64) | 0.02 | 163.04(118.19~217.47) | 87.66(60.64~117.84) | -2.14 (-2.48 ~ -1.8) |
| Guam | 182.14(122.45~266.18) | 230.88(157.14~321.16) | 0.27 | 170.52(117.9~241.58) | 124.12(85.15~172.76) | -0.72 (-1 ~ -0.45) |
| Guatemala | 28267.93(22205.7~34863.77) | 61012.7(45576.3~79036.84) | 1.16 | 592.01(465.03~725.58) | 475.76(356.67~615.5) | -1.01 (-1.29 ~ -0.73) |
| Guinea | 11969.09(7971.24~18357.2) | 19609.33(12388.32~28978.29) | 0.64 | 320.11(213.75~488.42) | 267.15(172.5~394.06) | -0.4 (-0.51 ~ -0.29) |
| Guinea-Bissau | 2667.83(1687.78~4001.5) | 4116.87(2583.8~6230.58) | 0.54 | 518.24(331.81~775.71) | 364.54(235.79~534.76) | -0.99 (-1.1 ~ -0.89) |
| Guyana | 1790.86(1249.83~2407.42) | 1785.75(1155.19~2661.05) | 0 | 363.06(256.66~499.05) | 243.39(159.99~357.53) | -0.87 (-1.14 ~ -0.6) |
| Haiti | 15221.68(9533.17~22222.92) | 22925.33(13538.74~37002.28) | 0.51 | 384.18(242.98~557.69) | 245.58(148.92~386.55) | -1.38 (-1.45 ~ -1.32) |
| Honduras | 11458.72(8633.08~15120.02) | 23500.07(13399.7~37931.62) | 1.05 | 431.37(327.3~565.62) | 316.5(177.57~503.28) | -1.11 (-1.17 ~ -1.04) |
| Hungary | 34878.14(23205.61~49865.68) | 15242.53(10419.44~22107.55) | -0.56 | 268.76(179.03~387.57) | 99.99(67.75~145.02) | -4.85 (-5.35 ~ -4.34) |
| Iceland | 68.32(49.21~93.47) | 74.67(53.67~103.55) | 0.09 | 26.66(19~36.89) | 16.22(11.43~22.69) | -1.89 (-2.09 ~ -1.68) |
| India | 1174315.14(917038.85~1503192.56) | 1894750.06(1278047.35~2539841.14) | 0.61 | 188.64(147.44~243.74) | 139.04(93.65~185.47) | -0.91 (-1.07 ~ -0.76) |
| Indonesia | 899731.54(680740.72~1253378.78) | 1266654.19(1020553.09~1608138.36) | 0.41 | 708.17(531.9~997.16) | 468.08(374.94~581.93) | -1.32 (-1.41 ~ -1.23) |
| Iran (Islamic Republic of) | 27156.13(20080.97~38441.24) | 38345.55(30811.26~50661.37) | 0.41 | 93.14(69.56~131.31) | 45.22(36.8~59.03) | -2.19 (-2.29 ~ -2.1) |
| Iraq | 19667.12(13837.66~28377.87) | 37437.64(25132.41~52062.07) | 0.9 | 217.88(150.85~315.52) | 136.26(93.3~189.96) | -1.8 (-1.9 ~ -1.71) |
| Ireland | 1224.04(889.62~1650.36) | 1751.52(1242.34~2365.28) | 0.43 | 33.13(23.77~44.97) | 26.02(18.36~35.7) | -0.45 (-0.95 ~ 0.06) |
| Israel | 2540.2(1923.45~3362.07) | 2880.31(2127.13~3785.5) | 0.13 | 54.54(41.12~72.38) | 26.48(19.46~34.91) | -2.49 (-2.74 ~ -2.23) |
| Italy | 141788.9(129185.62~153172.84) | 63460.74(56205.87~71282.06) | -0.55 | 178.91(164.25~192.77) | 54.27(48.64~60.68) | -3.87 (-3.92 ~ -3.82) |
| Jamaica | 942.1(702.02~1262) | 938.76(598.18~1400.13) | 0 | 54.98(40.3~74.91) | 30.43(19.45~45.45) | -2.26 (-2.7 ~ -1.83) |
| Japan | 285426.04(263441.52~305002.66) | 183888.83(162786.06~203040.8) | -0.36 | 169.86(157.4~181.16) | 73.73(67.3~80.35) | -2.9 (-3 ~ -2.79) |
| Jordan | 3003.24(2167.5~4172.31) | 6146.66(4486.87~8254.98) | 1.05 | 197.12(143.14~273.94) | 72.94(52.9~96.49) | -3.88 (-4.3 ~ -3.46) |
| Kazakhstan | 16626.42(12039.3~22556.19) | 55865.15(38471.26~76988.99) | 2.36 | 120.35(88.87~162.19) | 283.12(198.92~388.47) | 3.15 (2.34 ~ 3.97) |
| Kenya | 47213.79(29666.59~80463.56) | 133446.8(93281.18~200318.98) | 1.83 | 453.63(281.42~799.49) | 445.55(314.73~659.06) | 0.22 (-0.07 ~ 0.5) |
| Kiribati | 142.15(92.09~214.42) | 226.82(137.64~352.18) | 0.6 | 270.23(171.75~413.28) | 221.81(138.1~338.49) | -0.61 (-0.68 ~ -0.53) |
| Kuwait | 837.85(644.82~1043.2) | 1895.38(1398.96~2477.13) | 1.26 | 100(78.06~124.39) | 54.65(40.51~70.39) | -1.75 (-2.68 ~ -0.8) |
| Kyrgyzstan | 9998.29(7353.25~13132.86) | 19626.44(13279.94~27082.11) | 0.96 | 306.29(227.14~403.03) | 321.75(221.7~440.43) | 0.03 (-0.71 ~ 0.77) |
| Lao People's Democratic Republic | 10872.51(6618.47~16633.16) | 12732.86(8129.16~20082.62) | 0.17 | 425.01(261.87~659.29) | 214.29(139.7~336.27) | -2.46 (-2.58 ~ -2.34) |
| Latvia | 2102.26(1516.52~2851.92) | 3248.62(2254.69~4577.14) | 0.55 | 64.38(46.34~87.49) | 126.19(85.67~178.19) | 1.49 (0.75 ~ 2.24) |
| Lebanon | 4755.05(3141.92~6521.62) | 5930.96(4405.04~7702.99) | 0.25 | 212.81(142.6~285.84) | 97.53(71.99~127.13) | -2.27 (-2.46 ~ -2.08) |
| Lesotho | 2852.98(1498.25~5332.71) | 5049.47(3352.62~7335.06) | 0.77 | 300.21(158.97~569.01) | 372.4(249.73~542.5) | 1.27 (0.8 ~ 1.74) |
| Liberia | 4317.91(2868.65~5887.37) | 7822.84(4972.34~11672.92) | 0.81 | 317.95(211.86~434.55) | 245.5(160.96~373.56) | -1.05 (-1.21 ~ -0.9) |
| Libya | 5181.71(2997.86~10235.22) | 11992.76(7248.29~19834.99) | 1.31 | 243.51(138.44~490.81) | 196.3(117.55~324.26) | -0.53 (-0.89 ~ -0.16) |
| Lithuania | 4671.54(3612.13~5775.76) | 8847.12(6696.73~11115.09) | 0.89 | 111.18(86.48~137.08) | 238.43(180.75~297.23) | 2.72 (1.6 ~ 3.86) |
| Luxembourg | 600.63(425.47~828.86) | 441.74(317.49~605.73) | -0.26 | 122.74(85.72~169.45) | 46.01(33.19~62.2) | -3.1 (-3.24 ~ -2.95) |
| Madagascar | 24271.95(16884.69~36084.87) | 51121(32571.82~78198.55) | 1.11 | 383.95(266.65~570.84) | 308.65(199.72~466.11) | -0.71 (-0.77 ~ -0.65) |
| Malawi | 26286.74(18490.41~37177.78) | 48272.05(34007.59~65765.71) | 0.84 | 519.83(375.48~725.03) | 455.88(334.46~605.61) | -0.68 (-0.97 ~ -0.39) |
| Malaysia | 13659.46(9522.45~19000.4) | 33859.2(23862.54~45672.57) | 1.48 | 120.07(86.53~164.08) | 109.13(76.76~146.48) | -0.88 (-1.09 ~ -0.66) |
| Maldives | 258.49(170.76~351) | 314.96(213.71~448.79) | 0.22 | 227.26(150.46~308.23) | 62.23(44.34~86.99) | -4.53 (-4.72 ~ -4.34) |
| Mali | 15911.93(10186.93~23103.43) | 30149.88(20796.24~42539.43) | 0.89 | 326.95(212.32~464.49) | 254.92(181.27~362.07) | -0.49 (-0.68 ~ -0.31) |
| Malta | 237.78(172.62~323.03) | 195.74(142.61~265.2) | -0.18 | 55.83(40.55~76.13) | 28.94(20.61~39.76) | -1.95 (-2.07 ~ -1.83) |
| Marshall Islands | 73.75(48.4~116.79) | 104.63(58.66~167.38) | 0.42 | 297.7(193.11~458.72) | 205.19(118.41~322.24) | -1.24 (-1.28 ~ -1.21) |
| Mauritania | 3656.4(2412.68~5447.75) | 4580.81(2810.25~7268.13) | 0.25 | 317.04(207.11~471.64) | 176.1(109.57~279.73) | -1.94 (-2.08 ~ -1.8) |
| Mauritius | 2341(1703.39~3121.61) | 1953.44(1382.6~2603.62) | -0.17 | 261.01(193.66~344.76) | 114.71(81.09~152.53) | -3.87 (-4.24 ~ -3.5) |
| Mexico | 306180.95(282651.24~329262.62) | 434928.71(368275.23~505726.9) | 0.42 | 561(516.83~604.47) | 318.8(270.37~369.54) | -2.29 (-2.45 ~ -2.13) |
| Micronesia (Federated States of) | 170.39(109.02~249.97) | 164.9(94.93~259.34) | -0.03 | 263.15(166.59~391.89) | 172.63(101.56~270.76) | -1.38 (-1.49 ~ -1.26) |
| Monaco | 32.28(22.26~45.36) | 35.83(24.06~51.22) | 0.11 | 66.71(45.09~93.87) | 58.49(37.03~86.24) | -0.4 (-0.51 ~ -0.28) |
| Mongolia | 5630.83(4029.93~7611.09) | 7447.65(5278.19~10232.21) | 0.32 | 457.75(326.49~615.2) | 257.81(188.75~341.39) | -1.65 (-2.09 ~ -1.2) |
| Montenegro | 333.73(222.35~485.93) | 355.41(229.22~511.69) | 0.06 | 50.59(33.96~73.24) | 40.53(26.31~58.28) | -0.6 (-0.77 ~ -0.43) |
| Morocco | 30745.94(21526.6~43618.5) | 53886.7(35628.03~77613.14) | 0.75 | 199.65(136.95~285.23) | 152.02(99.74~216.15) | -0.84 (-0.87 ~ -0.8) |
| Mozambique | 14376.57(10341.11~21085.68) | 32223.21(21249.23~45672.04) | 1.24 | 196.23(143.14~278.9) | 200(134.22~283.07) | 0.49 (0.29 ~ 0.7) |
| Myanmar | 138252.09(78464.97~232416.9) | 148846.01(95140.07~211333.09) | 0.08 | 465.23(266.51~777.57) | 262.03(170.25~372.35) | -2.11 (-2.22 ~ -2.01) |
| Namibia | 2605.39(1501.27~4812.22) | 4480.99(2706.5~7244.17) | 0.72 | 330.49(195.8~604.83) | 253.54(163.13~395.54) | -1.05 (-1.37 ~ -0.72) |
| Nauru | 19.77(11.14~29.74) | 17.6(7.64~28.41) | -0.11 | 278.69(159.81~418.29) | 205.72(91.78~331.47) | -1.02 (-1.21 ~ -0.84) |
| Nepal | 14075.95(7755.02~23954.21) | 22078.93(13922.56~33386.04) | 0.57 | 108.93(60.12~185.88) | 81.9(51.48~121.42) | -0.88 (-0.97 ~ -0.79) |
| Netherlands | 6784.76(4873~9156.9) | 5751.8(4227.18~7741) | -0.15 | 37.69(26.8~51.32) | 20.64(14.9~27.28) | -2.23 (-2.47 ~ -1.99) |
| New Zealand | 1394.7(1261.02~1538.76) | 1914.81(1705.14~2130.79) | 0.37 | 37.57(33.84~41.54) | 26.28(23.53~29.18) | -0.99 (-1.18 ~ -0.79) |
| Nicaragua | 5479.11(4210.45~6962.41) | 13594.21(9716~18034.2) | 1.48 | 273.07(209.07~341.94) | 233.93(168.54~310.95) | -0.26 (-0.47 ~ -0.06) |
| Niger | 15154.6(9740.05~25547.7) | 32682.74(18260.52~59050.11) | 1.16 | 403.47(262.1~676.96) | 296.1(172.65~526.59) | -0.83 (-0.92 ~ -0.74) |
| Nigeria | 185529.72(131467.89~266142.16) | 244610.27(156761.99~351804.3) | 0.32 | 351.29(246.55~505.25) | 196.69(132.8~273.46) | -1.86 (-2.04 ~ -1.68) |
| Niue | 3.24(2.07~4.9) | 2.24(1.43~3.45) | -0.31 | 159.18(99.88~244.16) | 113.66(72.26~171.38) | -1.43 (-1.54 ~ -1.31) |
| North Macedonia | 1265(854.55~1781.01) | 1403.99(894.66~2063.35) | 0.11 | 61.62(42.32~85.85) | 43.04(27.62~63.54) | -1.21 (-1.47 ~ -0.95) |
| Northern Mariana Islands | 78.9(47.48~123.09) | 84.05(57.01~119.62) | 0.07 | 222.99(144.97~337.41) | 143.14(100.44~199.11) | -1.5 (-1.81 ~ -1.2) |
| Norway | 1746.91(1513.88~2027.13) | 1533.3(1327.22~1793.75) | -0.12 | 33.72(28.92~39.5) | 19.1(16.46~22.42) | -1.91 (-2.16 ~ -1.66) |
| Oman | 2402.53(1548.38~3731.89) | 3265.25(2227.92~5012.42) | 0.36 | 298(194.62~465.78) | 129.09(89.46~181.01) | -2.21 (-2.35 ~ -2.07) |
| Pakistan | 321143.53(165606.86~589928.09) | 639581.13(450634.52~870483.47) | 0.99 | 454.5(236.34~844.81) | 368.98(264.55~496.95) | -0.94 (-1.14 ~ -0.74) |
| Palau | 21.25(11.93~33.59) | 29.69(17.46~44.44) | 0.4 | 166.07(95.45~262.17) | 124.49(74.08~183.58) | -0.83 (-0.9 ~ -0.76) |
| Palestine | 2516.68(1478.14~4282.6) | 3431.41(2565.18~4371.51) | 0.36 | 284.49(167.29~486.21) | 128.78(96.65~164.39) | -2.53 (-2.69 ~ -2.38) |
| Panama | 2287.47(1773.45~2778.52) | 3977.79(2833.78~5292.84) | 0.74 | 135.72(105.56~166.42) | 90.26(64.38~120.12) | -1.34 (-1.44 ~ -1.24) |
| Papua New Guinea | 2513.41(1393.08~4162.75) | 5091.58(3235.34~7439.66) | 1.03 | 92.46(51.46~149.89) | 64.73(41.03~93.11) | -1.25 (-1.4 ~ -1.1) |
| Paraguay | 2597.94(1924.8~3438.61) | 6724.64(4436.52~9551.77) | 1.59 | 97.12(73.17~128.01) | 103.31(68.41~147.55) | 0.42 (0.28 ~ 0.55) |
| Peru | 12375.31(8151.99~18403.87) | 17643.56(11163.18~26430.16) | 0.43 | 86.63(58.05~128.34) | 50(31.49~74.94) | -2.26 (-2.59 ~ -1.93) |
| Philippines | 74387.58(56573.07~104409.25) | 140126.74(110711.39~176727.08) | 0.88 | 181.66(135.47~262.58) | 141.81(112.79~177.93) | -0.82 (-0.86 ~ -0.77) |
| Poland | 41181(36273.62~47134.86) | 75132.52(63559.86~87817.12) | 0.82 | 95.77(84.81~109.76) | 137.05(115.66~161.17) | 0.9 (0.57 ~ 1.23) |
| Portugal | 21570.97(15577~29698.94) | 9030.95(6516.62~12184.72) | -0.58 | 175.61(124.55~241.87) | 51.05(36.47~70.42) | -4.23 (-4.4 ~ -4.05) |
| Puerto Rico | 6989.87(5052.11~9451.43) | 3414.82(2375.04~4666.68) | -0.51 | 195.61(140.29~263.95) | 64.44(43.88~89.19) | -4.16 (-4.58 ~ -3.74) |
| Qatar | 529.51(388.51~717.75) | 1925.73(1258.99~2840.19) | 2.64 | 379.58(269.51~521.92) | 149.77(105.46~212.3) | -3.09 (-3.79 ~ -2.39) |
| Republic of Korea | 75792.53(51269.27~111698.44) | 33001.27(22540.84~48953.86) | -0.56 | 201.69(139.07~290.91) | 38.9(26.51~57.56) | -5.57 (-5.77 ~ -5.37) |
| Republic of Moldova | 26337.1(18884.43~35836.49) | 20522.54(14418.53~28560.03) | -0.22 | 565.9(410.15~764.66) | 384.37(269.29~536.2) | -1.3 (-1.79 ~ -0.81) |
| Romania | 51540.52(36837.15~70924.62) | 54580.75(37875.29~78536.92) | 0.06 | 186.69(134.21~259.2) | 182.35(126.31~262.3) | -1.09 (-1.53 ~ -0.64) |
| Russian Federation | 73826.76(61926.55~89473.25) | 256603.8(203965.81~322433.44) | 2.48 | 41.32(34.8~49.95) | 131.21(102.77~166.15) | 3.57 (2.5 ~ 4.65) |
| Rwanda | 25842.41(18905.21~34588.34) | 34114.29(22426.78~52679.12) | 0.32 | 700.5(508.12~931.12) | 405.66(276.45~593.84) | -2.65 (-2.98 ~ -2.32) |
| Saint Kitts and Nevis | 69.32(51.01~91.21) | 74.39(48.7~108.75) | 0.07 | 215.71(155.95~288.2) | 95.49(63.35~136.69) | -2.52 (-3.13 ~ -1.91) |
| Saint Lucia | 187.15(136.3~246.72) | 210.13(143.09~301.57) | 0.12 | 207.23(153.02~274.44) | 89.46(61.67~124.87) | -2.87 (-3.4 ~ -2.34) |
| Saint Vincent and the Grenadines | 92.47(68.2~122.45) | 128.56(88.61~177.91) | 0.39 | 127.34(91.71~171.91) | 92.41(63.93~127.51) | -1.27 (-1.77 ~ -0.76) |
| Samoa | 152.47(94.59~239.32) | 200.14(117.35~300.76) | 0.31 | 142.44(88.26~221.5) | 117.2(69.74~174.35) | -0.66 (-0.9 ~ -0.42) |
| San Marino | 26.22(18.14~35.86) | 22.68(13.06~35.07) | -0.14 | 84.17(59.06~115.83) | 39.06(21.5~61.43) | -1.67 (-1.95 ~ -1.39) |
| Sao Tome and Principe | 368.84(251.83~515.44) | 522.24(302.94~804.53) | 0.42 | 524.17(359.77~740.91) | 358.01(208.2~544.78) | -1.41 (-1.67 ~ -1.15) |
| Saudi Arabia | 49860.38(28933.86~94311.99) | 92653.39(67234.4~123411.41) | 0.86 | 744.71(423.09~1420.38) | 356.7(264.67~468.97) | -2.13 (-2.25 ~ -2.01) |
| Senegal | 11095.11(7423.63~16584.09) | 19048.72(11445.08~28995.22) | 0.72 | 279.24(189.65~415.9) | 194.04(119.82~286.98) | -0.98 (-1.17 ~ -0.78) |
| Serbia | 9506.19(6383.44~13901.37) | 7315.2(5042.51~10439.48) | -0.23 | 80.82(55.05~114.71) | 53.49(35.82~74.96) | -1.5 (-1.71 ~ -1.29) |
| Seychelles | 181.87(133.08~237.3) | 243.06(166.53~332.97) | 0.34 | 321.14(233.81~421.15) | 191.09(133.49~256.86) | -1.96 (-2.21 ~ -1.72) |
| Sierra Leone | 7248.09(4640.83~11043.12) | 11839.19(7662.45~17714.33) | 0.63 | 301.27(193.14~455.25) | 228.78(152.86~335.65) | -0.63 (-0.77 ~ -0.49) |
| Singapore | 2041.78(1494.87~2613.74) | 1031.52(768.5~1399.57) | -0.49 | 75.56(55.83~96.98) | 12.03(8.98~16.09) | -5.56 (-5.83 ~ -5.29) |
| Slovakia | 10574.4(7116.26~14805.05) | 8619.3(5827.76~12250.75) | -0.18 | 185.14(124.24~260.29) | 105.93(70.75~151.18) | -1.39 (-1.62 ~ -1.17) |
| Slovenia | 4135.26(2782.57~5984.04) | 1848.32(1281.81~2584.98) | -0.55 | 172.41(115.63~247.57) | 52.11(36.31~71.92) | -4.27 (-4.75 ~ -3.79) |
| Solomon Islands | 491.08(271.27~868.33) | 1262.69(795.74~1902.16) | 1.57 | 251.27(141.36~436.68) | 244.61(156.16~369.61) | 0 (-0.08 ~ 0.07) |
| Somalia | 19204.3(11672.29~28767.61) | 42989.89(25131.21~67216.65) | 1.24 | 521.86(332.7~757.62) | 438.11(260.9~665.16) | -0.72 (-0.81 ~ -0.63) |
| South Africa | 53728.82(44352.09~65602.28) | 75905.41(65592.28~88899.43) | 0.41 | 197.99(162.39~249.04) | 138.27(120.27~160.02) | -1.28 (-1.82 ~ -0.74) |
| South Sudan | 13915.77(9201.93~23446.61) | 23085.09(14621.01~33786.13) | 0.66 | 457.75(305.09~741.22) | 431.74(284.11~620.28) | -0.38 (-0.53 ~ -0.22) |
| Spain | 73174.11(55688.55~92944.43) | 34779.62(25922.94~45954.19) | -0.52 | 154.09(115.75~197.93) | 44.11(32.8~58.7) | -3.97 (-4.06 ~ -3.88) |
| Sri Lanka | 47954.16(30912.22~71609.14) | 29450.03(17523.56~45527.95) | -0.39 | 347.73(230.52~519.62) | 112.36(67.76~173.24) | -4.29 (-4.73 ~ -3.86) |
| Sudan | 35956.65(20953.87~64629.29) | 48941.08(26990.27~90209.39) | 0.36 | 347.85(197.88~637.98) | 210.13(120.74~382.47) | -1.65 (-1.69 ~ -1.62) |
| Suriname | 602.18(425.74~848.4) | 785.07(509.09~1175.72) | 0.3 | 206.11(148.53~287.43) | 119.66(78.69~178.7) | -1.85 (-2.04 ~ -1.66) |
| Sweden | 4879.73(4190.72~5694.66) | 4311.32(3531.36~5192.21) | -0.12 | 42.58(36.47~50.38) | 25.78(21.01~31.18) | -0.91 (-1.15 ~ -0.68) |
| Switzerland | 5172.41(3646.06~7160.03) | 3693.14(2658.69~5024.19) | -0.29 | 58.97(41.29~82.1) | 25.27(18.38~33.75) | -2.82 (-3.08 ~ -2.56) |
| Syrian Arab Republic | 14753.94(10490.04~20694.14) | 22215.02(14765.38~32198.97) | 0.51 | 249.28(174.24~352.17) | 166.05(112.98~235.67) | -1.37 (-1.52 ~ -1.22) |
| Taiwan (Province of China) | 41541.67(31017.26~54669.87) | 41791.57(29802.15~56778.91) | 0.01 | 232.56(177.07~302.25) | 113.55(80.89~154.17) | -2.44 (-2.7 ~ -2.18) |
| Tajikistan | 9451.12(6854.35~12177.88) | 16480.9(10981.76~23408.88) | 0.74 | 291.14(207.78~374.37) | 206.99(142.83~279.91) | -1.63 (-1.96 ~ -1.31) |
| Thailand | 88281.06(59131.73~127724.02) | 162753.58(104294.86~236583.45) | 0.84 | 194.9(132.83~276.57) | 170.69(110.07~248.55) | -0.47 (-0.67 ~ -0.28) |
| Timor-Leste | 1049.35(544.78~2175.66) | 1712.1(1032.66~2930.16) | 0.63 | 237.52(121.88~478.56) | 179.99(107.83~311.06) | -1.01 (-1.25 ~ -0.76) |
| Togo | 4842.98(3137.23~7075.16) | 12410.27(7835.36~19128.6) | 1.56 | 282.91(183.85~412.97) | 230.82(145.8~354.82) | -0.52 (-0.65 ~ -0.39) |
| Tokelau | 2.37(1.47~3.7) | 1.6(1.05~2.28) | -0.32 | 182.55(112.7~280.97) | 114.38(75.39~163.87) | -1.86 (-2 ~ -1.71) |
| Tonga | 160.26(105.06~237.98) | 156.09(96.39~239.05) | -0.03 | 248.14(161.11~364.08) | 179.58(110.47~275.11) | -0.94 (-0.98 ~ -0.9) |
| Trinidad and Tobago | 1132.14(822.94~1536.85) | 1023.66(668.9~1517.18) | -0.1 | 121.17(88.79~163.68) | 56.14(36.39~83.68) | -2.67 (-2.94 ~ -2.4) |
| Tunisia | 9525.34(6649.7~13982.41) | 16443.84(10917.31~23946.38) | 0.73 | 182.28(125.59~269.4) | 123.32(83.13~178.2) | -1.25 (-1.3 ~ -1.2) |
| Turkmenistan | 7876.36(5776.45~10426.08) | 27477.28(17975.06~39767.76) | 2.49 | 340.92(253.81~448.92) | 545.8(362.87~783.19) | 1.61 (1.34 ~ 1.88) |
| Tuvalu | 19.59(12.23~28.98) | 18.41(12.42~25.98) | -0.06 | 245.91(154.23~363.2) | 160.23(108.19~226.64) | -1.31 (-1.43 ~ -1.2) |
| Türkiye | 46381.01(32810.35~63982.87) | 50901.5(34886.06~69900.18) | 0.1 | 121.02(85.34~168.95) | 53.83(37.24~73.05) | -2.67 (-2.92 ~ -2.42) |
| Uganda | 29506.53(21066.85~41315.35) | 61152.97(40911.21~88100.17) | 1.07 | 372.09(262.07~519.37) | 288.87(192.39~417.44) | -1.36 (-1.6 ~ -1.12) |
| Ukraine | 59532.2(50258.03~69425.3) | 140987.66(96677.85~198643.09) | 1.37 | 88.94(75.13~104.28) | 238.12(164.19~335.55) | 2.78 (1.65 ~ 3.92) |
| United Arab Emirates | 1388.95(964.03~2084.73) | 5225.44(3483.49~7484.93) | 2.76 | 229.83(161.71~335.46) | 113.54(76.77~170.22) | -1.07 (-1.53 ~ -0.62) |
| United Kingdom | 11869.38(9420.56~15140.27) | 22763.12(17607.69~29389.48) | 0.92 | 17.14(13.34~21.95) | 24.56(18.82~32.22) | 1.13 (0.45 ~ 1.81) |
| United Republic of Tanzania | 47130.05(34301.29~62353.35) | 96968.23(65990.95~135319.88) | 1.06 | 360.13(259.02~473.05) | 288.34(197.03~397.54) | -0.68 (-0.89 ~ -0.47) |
| United States Virgin Islands | 158.37(106.37~224.6) | 85.78(57.11~124.46) | -0.46 | 157.7(109.01~220.17) | 69.17(45.13~101.17) | -2.46 (-2.68 ~ -2.24) |
| United States of America | 449958.09(410695.65~492133.83) | 743847.7(665903.83~825760.66) | 0.65 | 157.38(143.4~172.42) | 160.14(143.77~177.72) | 0.16 (0.02 ~ 0.3) |
| Uruguay | 5089.08(4010.13~6264.06) | 3803.33(3004.99~4689.99) | -0.25 | 142.81(112.76~174.03) | 83.43(65.75~103.21) | -1.89 (-2.06 ~ -1.73) |
| Uzbekistan | 40140.21(29655.21~53382.21) | 87802.69(61060.99~119145.3) | 1.19 | 303.32(226.5~400.33) | 261.66(188.21~351.67) | -0.91 (-1.33 ~ -0.49) |
| Vanuatu | 277.62(137.07~535.56) | 556.78(319.3~943.21) | 1.01 | 293.88(147.01~575.33) | 226.6(130.65~386.48) | -0.99 (-1.08 ~ -0.9) |
| Venezuela (Bolivarian Republic of) | 24788.78(18997.3~30832.34) | 42925.76(27997.79~63488.63) | 0.73 | 208.13(160.77~261.49) | 137.41(90.25~202.35) | -1.47 (-1.74 ~ -1.2) |
| Viet Nam | 130980.91(81876.32~207524.77) | 198171.23(128832.6~279831.55) | 0.51 | 303.45(190.15~484.96) | 182.2(123.11~253.3) | -1.63 (-1.7 ~ -1.55) |
| Yemen | 22449.41(12686.83~41088.74) | 38019.68(24432.95~55997.14) | 0.69 | 407.32(222.8~756.2) | 234.44(152.1~342.11) | -2.16 (-2.32 ~ -2) |
| Zambia | 20005.77(14444.14~27476.16) | 41855.87(24801.94~62142.58) | 1.09 | 532.5(382.13~736.93) | 398.84(237.35~578.49) | -1.27 (-1.59 ~ -0.96) |
| Zimbabwe | 12220.04(8807.1~16932.79) | 29213.49(14158.33~45583.95) | 1.39 | 248.21(177.56~349.26) | 307.35(150.16~471.26) | 1.04 (0.48 ~ 1.6) |
