# Supplementary material for: Global, regional, and national burden of chronic hepatitis C including cirrhosis from 1990 to 2021, alongside projections for the next 15 years
Source: Medicine (Baltimore). 2026 Oct 16;105(42):e51118. doi: 10.1097/MD.0000000000051118 (PMC13681144; doi:10.1097/MD.0000000000051118)
Supplement: Supplementary file 11 [file medi-105-e51118-s011.docx]

Supplementary Table S9. Joinpoint regression analysis on age-standardized prevalence rates for chronic hepatitis C including cirrhosis at global level and 5 SDI regions from 1990 to 2021.

| location | Model | Segment | Segment Start | Segment End | APC | APC 95% LCL | APC 95% UCL | APC Significant | Test Statistic | P-Value | location | Start Obs | End Obs | AAPC | AAPC C.I. Low | AAPC C.I. High | Statistically Significant (0=No 1=Yes) | Test Statistic | P-Value |
| --- | --- | --- | --- | --- | --- | --- | --- | --- | --- | --- | --- | --- | --- | --- | --- | --- | --- | --- | --- |
| Global | 4 | 0 | 1990 | 1995 | -0.4056 | -0.551 | -0.26 | 1 | -5.8453 | 0.000015 | Global | 1990 | 2021 | -0.8039 | -0.8595 | -0.7483 | 1 | -28.2246 | 0 |
| Global | 4 | 1 | 1995 | 2000 | -2.4258 | -2.6242 | -2.2271 | 1 | -25.3551 | 0 |  |  |  |  |  |  |  |  |  |
| Global | 4 | 2 | 2000 | 2005 | -0.1144 | -0.3188 | 0.0905 | 0 | -1.1732 | 0.256011 |  |  |  |  |  |  |  |  |  |
| Global | 4 | 3 | 2005 | 2015 | -0.7827 | -0.8421 | -0.7233 | 1 | -27.5702 | 0 |  |  |  |  |  |  |  |  |  |
| Global | 4 | 4 | 2015 | 2021 | -0.3793 | -0.4957 | -0.2628 | 1 | -6.8298 | 0.000002 |  |  |  |  |  |  |  |  |  |
| High SDI | 4 | 0 | 1990 | 1996 | -0.2282 | -0.346 | -0.1103 | 1 | -4.0638 | 0.000729 | High SDI | 1990 | 2021 | -0.6415 | -0.7203 | -0.5626 | 1 | -15.9019 | 0 |
| High SDI | 4 | 1 | 1996 | 2001 | -1.3501 | -1.5636 | -1.1361 | 1 | -13.1807 | 0 |  |  |  |  |  |  |  |  |  |
| High SDI | 4 | 2 | 2001 | 2004 | -2.4931 | -3.1647 | -1.8168 | 1 | -7.674 | 0 |  |  |  |  |  |  |  |  |  |
| High SDI | 4 | 3 | 2004 | 2015 | -0.5794 | -0.6353 | -0.5234 | 1 | -21.6912 | 0 |  |  |  |  |  |  |  |  |  |
| High SDI | 4 | 4 | 2015 | 2021 | 0.3645 | 0.2363 | 0.4929 | 1 | 5.98 | 0.000012 |  |  |  |  |  |  |  |  |  |
| High-middle SDI | 4 | 0 | 1990 | 1995 | -0.0693 | -0.2796 | 0.1415 | 0 | -0.6909 | 0.498468 | High-middle SDI | 1990 | 2021 | -1.1261 | -1.2108 | -1.0412 | 1 | -25.8787 | 0 |
| High-middle SDI | 4 | 1 | 1995 | 2000 | -3.6284 | -3.9089 | -3.3471 | 1 | -26.6408 | 0 |  |  |  |  |  |  |  |  |  |
| High-middle SDI | 4 | 2 | 2000 | 2005 | -0.6625 | -0.9556 | -0.3685 | 1 | -4.7256 | 0.000169 |  |  |  |  |  |  |  |  |  |
| High-middle SDI | 4 | 3 | 2005 | 2010 | -1.6913 | -1.9822 | -1.3996 | 1 | -12.0941 | 0 |  |  |  |  |  |  |  |  |  |
| High-middle SDI | 4 | 4 | 2010 | 2021 | -0.4017 | -0.4664 | -0.337 | 1 | -13.013 | 0 |  |  |  |  |  |  |  |  |  |
| Low SDI | 5 | 0 | 1990 | 1994 | -0.3419 | -0.3914 | -0.2923 | 1 | -14.6738 | 0 | Low SDI | 1990 | 2021 | -0.7004 | -0.7183 | -0.6825 | 1 | -76.4001 | 0 |
| Low SDI | 5 | 1 | 1994 | 2005 | -0.2296 | -0.2416 | -0.2177 | 1 | -40.9415 | 0 |  |  |  |  |  |  |  |  |  |
| Low SDI | 5 | 2 | 2005 | 2010 | -1.1423 | -1.1899 | -1.0947 | 1 | -50.8741 | 0 |  |  |  |  |  |  |  |  |  |
| Low SDI | 5 | 3 | 2010 | 2014 | -1.5071 | -1.5824 | -1.4317 | 1 | -42.3212 | 0 |  |  |  |  |  |  |  |  |  |
| Low SDI | 5 | 4 | 2014 | 2019 | -0.9668 | -1.0155 | -0.9181 | 1 | -42.1151 | 0 |  |  |  |  |  |  |  |  |  |
| Low SDI | 5 | 5 | 2019 | 2021 | -0.6056 | -0.7631 | -0.4478 | 1 | -8.161 | 0.000001 |  |  |  |  |  |  |  |  |  |
| Low-middle SDI | 4 | 0 | 1990 | 1995 | -0.831 | -1.0212 | -0.6405 | 1 | -9.1327 | 0 | Low-middle SDI | 1990 | 2021 | -0.9825 | -1.0587 | -0.9062 | 1 | -25.1275 | 0 |
| Low-middle SDI | 4 | 1 | 1995 | 2000 | -1.8236 | -2.0806 | -1.5658 | 1 | -14.7475 | 0 |  |  |  |  |  |  |  |  |  |
| Low-middle SDI | 4 | 2 | 2000 | 2005 | 0.8209 | 0.5566 | 1.086 | 1 | 6.5422 | 0.000004 |  |  |  |  |  |  |  |  |  |
| Low-middle SDI | 4 | 3 | 2005 | 2010 | -0.6032 | -0.8644 | -0.3412 | 1 | -4.829 | 0.000135 |  |  |  |  |  |  |  |  |  |
| Low-middle SDI | 4 | 4 | 2010 | 2021 | -1.6496 | -1.7098 | -1.5893 | 1 | -57.0293 | 0 |  |  |  |  |  |  |  |  |  |
| Middle SDI | 4 | 0 | 1990 | 1995 | -0.7935 | -0.9973 | -0.5892 | 1 | -8.1383 | 0 | Middle SDI | 1990 | 2021 | -1.0972 | -1.1817 | -1.0126 | 1 | -25.2966 | 0 |
| Middle SDI | 4 | 1 | 1995 | 2000 | -4.1147 | -4.3928 | -3.8358 | 1 | -30.3887 | 0 |  |  |  |  |  |  |  |  |  |
| Middle SDI | 4 | 2 | 2000 | 2004 | -0.3178 | -0.7767 | 0.1432 | 0 | -1.4495 | 0.164396 |  |  |  |  |  |  |  |  |  |
| Middle SDI | 4 | 3 | 2004 | 2014 | -0.8149 | -0.899 | -0.7306 | 1 | -20.253 | 0 |  |  |  |  |  |  |  |  |  |
| Middle SDI | 4 | 4 | 2014 | 2021 | 0.035 | -0.0907 | 0.1609 | 0 | 0.5851 | 0.565732 |  |  |  |  |  |  |  |  |  |
