# Supplementary material for: Global, regional, and national burden of chronic hepatitis C including cirrhosis from 1990 to 2021, alongside projections for the next 15 years
Source: Medicine (Baltimore). 2026 Oct 16;105(42):e51118. doi: 10.1097/MD.0000000000051118 (PMC13681144; doi:10.1097/MD.0000000000051118)
Supplement: Supplementary file 12 [file medi-105-e51118-s012.docx]

Supplementary Table S10. Joinpoint regression analysis on age-standardized mortality rates for chronic hepatitis C including cirrhosis at global level and 5 SDI regions from 1990 to 2021.

| location | Model | Segment | Segment Start | Segment End | APC | APC 95% LCL | APC 95% UCL | APC Significant | Test Statistic | P-Value | location | Start Obs | End Obs | AAPC | AAPC C.I. Low | AAPC C.I. High | Statistically Significant (0=No 1=Yes) | Test Statistic | P-Value |
| --- | --- | --- | --- | --- | --- | --- | --- | --- | --- | --- | --- | --- | --- | --- | --- | --- | --- | --- | --- |
| Global | 4 | 0 | 1990 | 1995 | -0.5833 | -0.8525 | -0.3135 | 1 | -4.534 | 0.000257 | Global | 1990 | 2021 | -1.2213 | -1.3415 | -1.1008 | 1 | -19.764 | 0 |
| Global | 4 | 1 | 1995 | 2000 | -1.3723 | -1.7083 | -1.0351 | 1 | -8.5065 | 0 |  |  |  |  |  |  |  |  |  |
| Global | 4 | 2 | 2000 | 2003 | -0.2934 | -1.2629 | 0.6856 | 0 | -0.6318 | 0.535428 |  |  |  |  |  |  |  |  |  |
| Global | 4 | 3 | 2003 | 2008 | -1.064 | -1.3843 | -0.7427 | 1 | -6.9309 | 0.000002 |  |  |  |  |  |  |  |  |  |
| Global | 4 | 4 | 2008 | 2021 | -1.6802 | -1.7295 | -1.6307 | 1 | -70.8554 | 0 |  |  |  |  |  |  |  |  |  |
| High SDI | 5 | 0 | 1990 | 1994 | -0.3574 | -0.856 | 0.1437 | 0 | -1.5215 | 0.148943 | High SDI | 1990 | 2021 | -1.0682 | -1.2718 | -0.8642 | 1 | -10.2176 | 0 |
| High SDI | 5 | 1 | 1994 | 1997 | -1.7553 | -3.2075 | -0.2813 | 1 | -2.5347 | 0.022883 |  |  |  |  |  |  |  |  |  |
| High SDI | 5 | 2 | 1997 | 2010 | -0.9804 | -1.0648 | -0.896 | 1 | -24.6286 | 0 |  |  |  |  |  |  |  |  |  |
| High SDI | 5 | 3 | 2010 | 2015 | -0.4306 | -0.8679 | 0.0087 | 0 | -2.0895 | 0.054115 |  |  |  |  |  |  |  |  |  |
| High SDI | 5 | 4 | 2015 | 2019 | -2.5424 | -3.2479 | -1.8317 | 1 | -7.5545 | 0.000002 |  |  |  |  |  |  |  |  |  |
| High SDI | 5 | 5 | 2019 | 2021 | -0.6379 | -2.1754 | 0.9237 | 0 | -0.8747 | 0.395512 |  |  |  |  |  |  |  |  |  |
| High-middle SDI | 5 | 0 | 1990 | 1992 | -1.5092 | -4.5203 | 1.5968 | 0 | -1.044 | 0.313038 | High-middle SDI | 1990 | 2021 | -1.5792 | -1.9877 | -1.1691 | 1 | -7.502 | 0 |
| High-middle SDI | 5 | 1 | 1992 | 1995 | 1.2418 | -1.7747 | 4.3509 | 0 | 0.8696 | 0.3982 |  |  |  |  |  |  |  |  |  |
| High-middle SDI | 5 | 2 | 1995 | 1999 | -3.8013 | -5.2669 | -2.3131 | 1 | -5.3807 | 0.000076 |  |  |  |  |  |  |  |  |  |
| High-middle SDI | 5 | 3 | 1999 | 2005 | 1.4007 | 0.7019 | 2.1043 | 1 | 4.2872 | 0.000648 |  |  |  |  |  |  |  |  |  |
| High-middle SDI | 5 | 4 | 2005 | 2012 | -2.9599 | -3.4719 | -2.4451 | 1 | -12.1051 | 0 |  |  |  |  |  |  |  |  |  |
| High-middle SDI | 5 | 5 | 2012 | 2021 | -2.3953 | -2.6823 | -2.1074 | 1 | -17.548 | 0 |  |  |  |  |  |  |  |  |  |
| Low SDI | 5 | 0 | 1990 | 1995 | 0.1209 | -0.0118 | 0.2537 | 0 | 1.9423 | 0.071117 | Low SDI | 1990 | 2021 | -1.1955 | -1.2632 | -1.1277 | 1 | -34.3941 | 0 |
| Low SDI | 5 | 1 | 1995 | 2003 | -0.9909 | -1.0629 | -0.9188 | 1 | -29.1528 | 0 |  |  |  |  |  |  |  |  |  |
| Low SDI | 5 | 2 | 2003 | 2011 | -1.5819 | -1.6533 | -1.5105 | 1 | -46.8228 | 0 |  |  |  |  |  |  |  |  |  |
| Low SDI | 5 | 3 | 2011 | 2014 | -1.1856 | -1.7084 | -0.6601 | 1 | -4.7928 | 0.000237 |  |  |  |  |  |  |  |  |  |
| Low SDI | 5 | 4 | 2014 | 2018 | -2.1078 | -2.361 | -1.8539 | 1 | -17.5297 | 0 |  |  |  |  |  |  |  |  |  |
| Low SDI | 5 | 5 | 2018 | 2021 | -1.6738 | -1.9309 | -1.416 | 1 | -13.7429 | 0 |  |  |  |  |  |  |  |  |  |
| Low-middle SDI | 2 | 0 | 1990 | 2008 | -1.0879 | -1.1391 | -1.0368 | 1 | -43.6537 | 0 | Low-middle SDI | 1990 | 2021 | -1.5461 | -1.6126 | -1.4795 | 1 | -45.1773 | 0 |
| Low-middle SDI | 2 | 1 | 2008 | 2014 | -1.8591 | -2.1326 | -1.5849 | 1 | -13.88 | 0 |  |  |  |  |  |  |  |  |  |
| Low-middle SDI | 2 | 2 | 2014 | 2021 | -2.4484 | -2.6009 | -2.2957 | 1 | -32.7005 | 0 |  |  |  |  |  |  |  |  |  |
| Middle SDI | 3 | 0 | 1990 | 2001 | -1.5 | -1.6077 | -1.3922 | 1 | -28.7421 | 0 | Middle SDI | 1990 | 2021 | -1.5333 | -1.67 | -1.3965 | 1 | -21.8108 | 0 |
| Middle SDI | 3 | 1 | 2001 | 2004 | -1.0405 | -2.3041 | 0.2394 | 0 | -1.6927 | 0.105305 |  |  |  |  |  |  |  |  |  |
| Middle SDI | 3 | 2 | 2004 | 2018 | -1.7856 | -1.8486 | -1.7226 | 1 | -58.4429 | 0 |  |  |  |  |  |  |  |  |  |
| Middle SDI | 3 | 3 | 2018 | 2021 | -0.9666 | -1.6033 | -0.3257 | 1 | -3.1316 | 0.005042 |  |  |  |  |  |  |  |  |  |
