# Supplementary material for: Global, regional, and national burden of chronic hepatitis C including cirrhosis from 1990 to 2021, alongside projections for the next 15 years
Source: Medicine (Baltimore). 2026 Oct 16;105(42):e51118. doi: 10.1097/MD.0000000000051118 (PMC13681144; doi:10.1097/MD.0000000000051118)
Supplement: Supplementary file 13 [file medi-105-e51118-s013.docx]

Supplementary Table S11. Joinpoint regression analysis on age-standardized DALYs rates for chronic hepatitis C including cirrhosis at global level and 5 SDI regions from 1990 to 2021.

| location | Model | Segment | Segment Start | Segment End | APC | APC 95% LCL | APC 95% UCL | APC Significant | Test Statistic | P-Value | location | Start Obs | End Obs | AAPC | AAPC C.I. Low | AAPC C.I. High | Statistically Significant (0=No 1=Yes) | Test Statistic | P-Value |
| --- | --- | --- | --- | --- | --- | --- | --- | --- | --- | --- | --- | --- | --- | --- | --- | --- | --- | --- | --- |
| Global | 5 | 0 | 1990 | 1995 | -0.3316 | -0.4993 | -0.1637 | 1 | -4.2047 | 0.000766 | Global | 1990 | 2021 | -1.1129 | -1.2014 | -1.0243 | 1 | -24.4906 | 0 |
| Global | 5 | 1 | 1995 | 1998 | -1.632 | -2.2992 | -0.9603 | 1 | -5.1536 | 0.000118 |  |  |  |  |  |  |  |  |  |
| Global | 5 | 2 | 1998 | 2004 | -0.4654 | -0.6117 | -0.3189 | 1 | -6.761 | 0.000006 |  |  |  |  |  |  |  |  |  |
| Global | 5 | 3 | 2004 | 2008 | -0.9957 | -1.3125 | -0.6778 | 1 | -6.6531 | 0.000008 |  |  |  |  |  |  |  |  |  |
| Global | 5 | 4 | 2008 | 2019 | -1.7255 | -1.7728 | -1.6781 | 1 | -77.0044 | 0 |  |  |  |  |  |  |  |  |  |
| Global | 5 | 5 | 2019 | 2021 | -1.0679 | -1.6789 | -0.4531 | 1 | -3.6941 | 0.002165 |  |  |  |  |  |  |  |  |  |
| High SDI | 5 | 0 | 1990 | 1994 | -0.1315 | -0.9102 | 0.6534 | 0 | -0.3582 | 0.725183 | High SDI | 1990 | 2021 | -1.1673 | -1.4616 | -0.8722 | 1 | -7.7176 | 0 |
| High SDI | 5 | 1 | 1994 | 1997 | -2.0113 | -4.1842 | 0.2109 | 0 | -1.9312 | 0.072581 |  |  |  |  |  |  |  |  |  |
| High SDI | 5 | 2 | 1997 | 2001 | -0.4494 | -1.528 | 0.641 | 0 | -0.8813 | 0.392081 |  |  |  |  |  |  |  |  |  |
| High SDI | 5 | 3 | 2001 | 2005 | -1.6194 | -2.6732 | -0.5541 | 1 | -3.2312 | 0.005595 |  |  |  |  |  |  |  |  |  |
| High SDI | 5 | 4 | 2005 | 2015 | -0.8633 | -1.0495 | -0.6766 | 1 | -9.8265 | 0 |  |  |  |  |  |  |  |  |  |
| High SDI | 5 | 5 | 2015 | 2021 | -2.1068 | -2.4678 | -1.7444 | 1 | -12.2839 | 0 |  |  |  |  |  |  |  |  |  |
| High-middle SDI | 5 | 0 | 1990 | 1995 | 0.9191 | 0.1805 | 1.6632 | 1 | 2.6549 | 0.018015 | High-middle SDI | 1990 | 2021 | -1.3472 | -1.8381 | -0.8539 | 1 | -5.3293 | 0 |
| High-middle SDI | 5 | 1 | 1995 | 1999 | -3.6127 | -5.1905 | -2.0086 | 1 | -4.7517 | 0.000257 |  |  |  |  |  |  |  |  |  |
| High-middle SDI | 5 | 2 | 1999 | 2005 | 2.1023 | 1.3612 | 2.8489 | 1 | 6.0869 | 0.000021 |  |  |  |  |  |  |  |  |  |
| High-middle SDI | 5 | 3 | 2005 | 2008 | -1.8675 | -5.0793 | 1.4531 | 0 | -1.2074 | 0.245961 |  |  |  |  |  |  |  |  |  |
| High-middle SDI | 5 | 4 | 2008 | 2011 | -4.6194 | -7.7409 | -1.3924 | 1 | -3.0296 | 0.008447 |  |  |  |  |  |  |  |  |  |
| High-middle SDI | 5 | 5 | 2011 | 2021 | -2.4244 | -2.6878 | -2.1603 | 1 | -19.3524 | 0 |  |  |  |  |  |  |  |  |  |
| Low SDI | 5 | 0 | 1990 | 1995 | 0.1459 | 0.003 | 0.289 | 1 | 2.1757 | 0.04598 | Low SDI | 1990 | 2021 | -1.1747 | -1.243 | -1.1064 | 1 | -33.5298 | 0 |
| Low SDI | 5 | 1 | 1995 | 2000 | -0.7586 | -0.9583 | -0.5586 | 1 | -8.0599 | 0.000001 |  |  |  |  |  |  |  |  |  |
| Low SDI | 5 | 2 | 2000 | 2005 | -1.1857 | -1.3874 | -0.9835 | 1 | -12.4392 | 0 |  |  |  |  |  |  |  |  |  |
| Low SDI | 5 | 3 | 2005 | 2014 | -1.6401 | -1.7109 | -1.5692 | 1 | -48.9391 | 0 |  |  |  |  |  |  |  |  |  |
| Low SDI | 5 | 4 | 2014 | 2018 | -2.0332 | -2.3414 | -1.7242 | 1 | -13.8996 | 0 |  |  |  |  |  |  |  |  |  |
| Low SDI | 5 | 5 | 2018 | 2021 | -1.4853 | -1.787 | -1.1826 | 1 | -10.3989 | 0 |  |  |  |  |  |  |  |  |  |
| Low-middle SDI | 5 | 0 | 1990 | 1994 | -0.5296 | -0.884 | -0.174 | 1 | -3.1715 | 0.006323 | Low-middle SDI | 1990 | 2021 | -1.3241 | -1.4154 | -1.2327 | 1 | -28.2305 | 0 |
| Low-middle SDI | 5 | 1 | 1994 | 2000 | -0.9917 | -1.2012 | -0.7817 | 1 | -10.0272 | 0 |  |  |  |  |  |  |  |  |  |
| Low-middle SDI | 5 | 2 | 2000 | 2008 | -0.6924 | -0.8075 | -0.5772 | 1 | -12.7715 | 0 |  |  |  |  |  |  |  |  |  |
| Low-middle SDI | 5 | 3 | 2008 | 2013 | -1.7268 | -1.9779 | -1.475 | 1 | -14.5077 | 0 |  |  |  |  |  |  |  |  |  |
| Low-middle SDI | 5 | 4 | 2013 | 2019 | -2.5332 | -2.6869 | -2.3793 | 1 | -34.6562 | 0 |  |  |  |  |  |  |  |  |  |
| Low-middle SDI | 5 | 5 | 2019 | 2021 | -1.7613 | -2.5629 | -0.9531 | 1 | -4.6229 | 0.000332 |  |  |  |  |  |  |  |  |  |
| Middle SDI | 2 | 0 | 1990 | 2005 | -1.4433 | -1.501 | -1.3856 | 1 | -51.2393 | 0 | Middle SDI | 1990 | 2021 | -1.5134 | -1.5689 | -1.4578 | 1 | -52.9619 | 0 |
| Middle SDI | 2 | 1 | 2005 | 2015 | -1.8517 | -1.9554 | -1.7478 | 1 | -36.4714 | 0 |  |  |  |  |  |  |  |  |  |
| Middle SDI | 2 | 2 | 2015 | 2021 | -1.1228 | -1.3247 | -0.9206 | 1 | -11.4041 | 0 |  |  |  |  |  |  |  |  |  |
