# Supplementary material for: Global, regional, and national burden of chronic hepatitis C including cirrhosis from 1990 to 2021, alongside projections for the next 15 years
Source: Medicine (Baltimore). 2026 Oct 16;105(42):e51118. doi: 10.1097/MD.0000000000051118 (PMC13681144; doi:10.1097/MD.0000000000051118)
Supplement: Supplementary file 14 [file medi-105-e51118-s014.docx]

Supplementary Table S13. Frontier analysis based on SDI and age-standardized prevalence rate of chronic hepatitis C including cirrhosis at 204 countries and territories.

| location | The age-standardized prevalence rate/100000 in 2021 | SDI in 2021 | frontier | effective difference | trend |
| --- | --- | --- | --- | --- | --- |
| Afghanistan | 2697.34 | 0.34 | 724.23 | 1973.11 | Decrease |
| Albania | 919.99 | 0.71 | 498.20 | 421.79 | Decrease |
| Algeria | 1734.95 | 0.66 | 502.15 | 1232.79 | Decrease |
| American Samoa | 1365.91 | 0.72 | 491.25 | 874.66 | Increase |
| Andorra | 862.30 | 0.87 | 411.23 | 451.07 | Decrease |
| Angola | 4401.35 | 0.45 | 724.99 | 3676.36 | Decrease |
| Antigua and Barbuda | 1351.88 | 0.75 | 482.62 | 869.26 | Decrease |
| Argentina | 585.16 | 0.72 | 487.51 | 97.65 | Decrease |
| Armenia | 4492.50 | 0.70 | 501.66 | 3990.84 | Increase |
| Australia | 1263.71 | 0.84 | 453.63 | 810.08 | Decrease |
| Austria | 700.99 | 0.85 | 427.43 | 273.55 | Decrease |
| Azerbaijan | 5983.99 | 0.69 | 502.59 | 5481.40 | Decrease |
| Bahamas | 1382.74 | 0.81 | 459.23 | 923.50 | Decrease |
| Bahrain | 1599.53 | 0.75 | 483.01 | 1116.52 | Decrease |
| Bangladesh | 851.10 | 0.49 | 725.84 | 125.26 | Decrease |
| Barbados | 1299.27 | 0.75 | 483.25 | 816.02 | Decrease |
| Belarus | 2117.33 | 0.78 | 487.55 | 1629.78 | Increase |
| Belgium | 766.92 | 0.85 | 429.59 | 337.33 | Increase |
| Belize | 1752.71 | 0.61 | 554.89 | 1197.82 | Decrease |
| Benin | 3347.43 | 0.37 | 723.55 | 2623.88 | Decrease |
| Bermuda | 1109.13 | 0.82 | 459.48 | 649.65 | Decrease |
| Bhutan | 2217.06 | 0.47 | 724.96 | 1492.10 | Increase |
| Bolivia (Plurinational State of) | 1117.18 | 0.60 | 554.49 | 562.69 | Decrease |
| Bosnia and Herzegovina | 533.51 | 0.72 | 487.08 | 46.43 | Decrease |
| Botswana | 2017.04 | 0.64 | 518.93 | 1498.10 | Decrease |
| Brazil | 1958.80 | 0.65 | 507.68 | 1451.12 | Decrease |
| Brunei Darussalam | 1253.45 | 0.81 | 459.20 | 794.25 | Decrease |
| Bulgaria | 896.58 | 0.77 | 482.01 | 414.57 | Decrease |
| Burkina Faso | 4523.41 | 0.29 | 870.52 | 3652.89 | Decrease |
| Burundi | 4085.57 | 0.29 | 852.50 | 3233.07 | Decrease |
| Cabo Verde | 2668.08 | 0.53 | 723.72 | 1944.35 | Decrease |
| Cambodia | 4885.00 | 0.47 | 724.38 | 4160.63 | Decrease |
| Cameroon | 3670.91 | 0.48 | 724.81 | 2946.10 | Decrease |
| Canada | 565.97 | 0.87 | 363.95 | 202.02 | Decrease |
| Central African Republic | 3974.34 | 0.31 | 736.46 | 3237.88 | Decrease |
| Chad | 4486.02 | 0.24 | 993.06 | 3492.96 | Decrease |
| Chile | 932.22 | 0.77 | 482.29 | 449.93 | Decrease |
| China | 1260.41 | 0.72 | 490.90 | 769.51 | Decrease |
| Colombia | 1497.37 | 0.66 | 503.29 | 994.09 | Decrease |
| Comoros | 2261.85 | 0.48 | 723.82 | 1538.03 | Decrease |
| Congo | 3937.04 | 0.58 | 554.94 | 3382.09 | Decrease |
| Cook Islands | 1215.17 | 0.78 | 482.07 | 733.10 | Decrease |
| Costa Rica | 1588.94 | 0.70 | 501.73 | 1087.20 | Decrease |
| Croatia | 821.93 | 0.80 | 460.48 | 361.45 | Decrease |
| Cuba | 1391.18 | 0.67 | 504.51 | 886.67 | Decrease |
| Cyprus | 627.41 | 0.84 | 458.26 | 169.15 | Decrease |
| Czechia | 975.66 | 0.83 | 459.05 | 516.61 | Decrease |
| C么te d'Ivoire | 3078.79 | 0.43 | 727.39 | 2351.40 | Decrease |
| Democratic People's Republic of Korea | 2323.34 | 0.57 | 555.98 | 1767.36 | Increase |
| Democratic Republic of the Congo | 4508.05 | 0.38 | 723.61 | 3784.45 | Decrease |
| Denmark | 699.21 | 0.90 | 325.07 | 374.14 | Decrease |
| Djibouti | 2246.40 | 0.49 | 723.58 | 1522.82 | Decrease |
| Dominica | 1351.51 | 0.75 | 482.05 | 869.47 | Decrease |
| Dominican Republic | 1745.19 | 0.62 | 552.37 | 1192.82 | Decrease |
| Ecuador | 977.80 | 0.66 | 504.54 | 473.27 | Decrease |
| Egypt | 3456.12 | 0.61 | 553.85 | 2902.27 | Decrease |
| El Salvador | 1906.92 | 0.56 | 555.45 | 1351.48 | Decrease |
| Equatorial Guinea | 3260.23 | 0.66 | 505.01 | 2755.22 | Decrease |
| Eritrea | 2709.91 | 0.40 | 724.98 | 1984.93 | Decrease |
| Estonia | 1967.35 | 0.84 | 451.86 | 1515.49 | Increase |
| Eswatini | 2152.09 | 0.59 | 556.03 | 1596.06 | Decrease |
| Ethiopia | 2472.58 | 0.36 | 723.85 | 1748.73 | Decrease |
| Fiji | 1436.88 | 0.68 | 502.76 | 934.12 | Decrease |
| Finland | 740.76 | 0.86 | 417.80 | 322.96 | Increase |
| France | 573.75 | 0.84 | 455.47 | 118.28 | Decrease |
| Gabon | 4640.18 | 0.63 | 532.99 | 4107.19 | Decrease |
| Gambia | 2389.66 | 0.41 | 723.64 | 1666.02 | Decrease |
| Georgia | 2900.36 | 0.73 | 483.04 | 2417.32 | Decrease |
| Germany | 721.89 | 0.90 | 316.90 | 404.99 | Decrease |
| Ghana | 3765.69 | 0.56 | 556.02 | 3209.67 | Decrease |
| Greece | 547.32 | 0.79 | 462.43 | 84.90 | Increase |
| Greenland | 851.89 | 0.83 | 459.17 | 392.72 | Decrease |
| Grenada | 1486.99 | 0.67 | 501.57 | 985.42 | Decrease |
| Guam | 1376.69 | 0.80 | 460.03 | 916.66 | Increase |
| Guatemala | 2459.15 | 0.54 | 558.56 | 1900.59 | Decrease |
| Guinea | 3635.47 | 0.34 | 723.87 | 2911.59 | Decrease |
| Guinea-Bissau | 2615.43 | 0.35 | 726.01 | 1889.42 | Decrease |
| Guyana | 1920.90 | 0.65 | 510.49 | 1410.41 | Decrease |
| Haiti | 2475.05 | 0.45 | 725.73 | 1749.31 | Decrease |
| Honduras | 2314.45 | 0.51 | 724.15 | 1590.29 | Decrease |
| Hungary | 870.84 | 0.79 | 472.04 | 398.80 | Decrease |
| Iceland | 316.78 | 0.88 | 316.78 | 0.00 | Decrease |
| India | 1215.80 | 0.58 | 554.68 | 661.13 | Decrease |
| Indonesia | 2637.44 | 0.66 | 505.32 | 2132.11 | Decrease |
| Iran (Islamic Republic of) | 1039.36 | 0.70 | 501.52 | 537.83 | Decrease |
| Iraq | 2459.79 | 0.66 | 501.74 | 1958.04 | Decrease |
| Ireland | 647.36 | 0.87 | 361.30 | 286.06 | Decrease |
| Israel | 633.83 | 0.81 | 459.08 | 174.75 | Decrease |
| Italy | 1479.63 | 0.81 | 459.96 | 1019.67 | Decrease |
| Jamaica | 1334.44 | 0.68 | 501.49 | 832.95 | Decrease |
| Japan | 842.34 | 0.87 | 411.32 | 431.02 | Decrease |
| Jordan | 1818.29 | 0.73 | 490.63 | 1327.66 | Decrease |
| Kazakhstan | 3122.00 | 0.73 | 490.35 | 2631.64 | Increase |
| Kenya | 2402.42 | 0.52 | 723.86 | 1678.56 | Decrease |
| Kiribati | 2563.75 | 0.53 | 723.86 | 1839.89 | Increase |
| Kuwait | 1335.55 | 0.85 | 451.31 | 884.24 | Decrease |
| Kyrgyzstan | 6214.92 | 0.60 | 553.98 | 5660.94 | Increase |
| Lao People's Democratic Republic | 2509.47 | 0.49 | 723.83 | 1785.64 | Decrease |
| Latvia | 1539.69 | 0.83 | 459.19 | 1080.51 | Decrease |
| Lebanon | 1142.55 | 0.74 | 482.05 | 660.50 | Decrease |
| Lesotho | 2361.69 | 0.51 | 724.31 | 1637.38 | Increase |
| Liberia | 3524.79 | 0.35 | 725.01 | 2799.79 | Decrease |
| Libya | 1533.27 | 0.73 | 486.28 | 1046.99 | Decrease |
| Lithuania | 2194.28 | 0.86 | 424.33 | 1769.94 | Increase |
| Luxembourg | 728.61 | 0.88 | 349.92 | 378.69 | Decrease |
| Madagascar | 1822.85 | 0.40 | 723.58 | 1099.27 | Decrease |
| Malawi | 2692.20 | 0.38 | 724.33 | 1967.87 | Decrease |
| Malaysia | 2888.68 | 0.74 | 482.70 | 2405.99 | Decrease |
| Maldives | 2810.97 | 0.65 | 509.41 | 2301.56 | Decrease |
| Mali | 3505.67 | 0.27 | 916.61 | 2589.06 | Decrease |
| Malta | 682.46 | 0.80 | 459.32 | 223.14 | Decrease |
| Marshall Islands | 2335.05 | 0.57 | 555.49 | 1779.56 | Increase |
| Mauritania | 2772.03 | 0.50 | 726.00 | 2046.03 | Decrease |
| Mauritius | 3136.22 | 0.72 | 489.73 | 2646.49 | Decrease |
| Mexico | 1371.55 | 0.66 | 502.58 | 868.96 | Decrease |
| Micronesia (Federated States of) | 2241.90 | 0.59 | 556.34 | 1685.55 | Increase |
| Monaco | 755.93 | 0.91 | 324.72 | 431.22 | Increase |
| Mongolia | 8589.89 | 0.62 | 552.63 | 8037.27 | Decrease |
| Montenegro | 883.85 | 0.80 | 460.13 | 423.72 | Decrease |
| Morocco | 1856.36 | 0.56 | 555.08 | 1301.28 | Decrease |
| Mozambique | 2208.35 | 0.33 | 724.17 | 1484.18 | Decrease |
| Myanmar | 3268.64 | 0.53 | 627.35 | 2641.29 | Decrease |
| Namibia | 1957.51 | 0.62 | 551.54 | 1405.97 | Decrease |
| Nauru | 2089.72 | 0.63 | 547.95 | 1541.78 | Increase |
| Nepal | 880.93 | 0.43 | 725.46 | 155.47 | Decrease |
| Netherlands | 475.16 | 0.89 | 332.22 | 142.94 | Decrease |
| New Zealand | 1065.33 | 0.85 | 450.13 | 615.20 | Increase |
| Nicaragua | 2540.95 | 0.52 | 724.96 | 1815.99 | Decrease |
| Niger | 3996.76 | 0.17 | 3042.01 | 954.75 | Decrease |
| Nigeria | 3756.80 | 0.50 | 723.89 | 3032.91 | Decrease |
| Niue | 1480.98 | 0.73 | 490.03 | 990.95 | Decrease |
| North Macedonia | 919.60 | 0.75 | 484.38 | 435.22 | Decrease |
| Northern Mariana Islands | 1617.28 | 0.77 | 483.87 | 1133.40 | Increase |
| Norway | 432.70 | 0.92 | 356.30 | 76.39 | Decrease |
| Oman | 1576.90 | 0.77 | 482.01 | 1094.88 | Decrease |
| Pakistan | 2614.04 | 0.50 | 724.37 | 1889.67 | Decrease |
| Palau | 1723.12 | 0.75 | 482.18 | 1240.94 | Increase |
| Palestine | 1879.98 | 0.63 | 545.89 | 1334.09 | Decrease |
| Panama | 1377.05 | 0.71 | 497.07 | 879.99 | Decrease |
| Papua New Guinea | 2613.84 | 0.42 | 723.74 | 1890.10 | Decrease |
| Paraguay | 1854.73 | 0.64 | 536.23 | 1318.50 | Decrease |
| Peru | 955.52 | 0.66 | 504.16 | 451.35 | Decrease |
| Philippines | 2035.81 | 0.65 | 513.03 | 1522.78 | Decrease |
| Poland | 911.45 | 0.81 | 459.07 | 452.38 | Decrease |
| Portugal | 1173.80 | 0.74 | 482.81 | 690.99 | Increase |
| Puerto Rico | 1297.85 | 0.83 | 459.09 | 838.76 | Decrease |
| Qatar | 1515.14 | 0.85 | 448.66 | 1066.48 | Decrease |
| Republic of Korea | 870.27 | 0.89 | 319.34 | 550.94 | Decrease |
| Republic of Moldova | 2800.50 | 0.73 | 483.15 | 2317.35 | Decrease |
| Romania | 1022.15 | 0.77 | 482.57 | 539.58 | Decrease |
| Russian Federation | 2165.48 | 0.81 | 459.09 | 1706.39 | Increase |
| Rwanda | 1606.80 | 0.44 | 724.65 | 882.16 | Decrease |
| Saint Kitts and Nevis | 1394.82 | 0.75 | 482.87 | 911.95 | Decrease |
| Saint Lucia | 1497.63 | 0.67 | 505.76 | 991.88 | Decrease |
| Saint Vincent and the Grenadines | 1481.19 | 0.64 | 537.51 | 943.67 | Decrease |
| Samoa | 2237.87 | 0.59 | 554.73 | 1683.14 | Increase |
| San Marino | 699.24 | 0.89 | 389.52 | 309.71 | Increase |
| Sao Tome and Principe | 2988.78 | 0.51 | 727.45 | 2261.33 | Decrease |
| Saudi Arabia | 2821.26 | 0.82 | 459.19 | 2362.07 | Decrease |
| Senegal | 2334.10 | 0.41 | 727.36 | 1606.73 | Decrease |
| Serbia | 905.00 | 0.79 | 460.67 | 444.33 | Decrease |
| Seychelles | 2237.41 | 0.73 | 483.11 | 1754.31 | Decrease |
| Sierra Leone | 3316.72 | 0.36 | 723.85 | 2592.87 | Decrease |
| Singapore | 1284.09 | 0.86 | 429.28 | 854.81 | Increase |
| Slovakia | 949.72 | 0.81 | 459.85 | 489.87 | Decrease |
| Slovenia | 797.51 | 0.84 | 454.34 | 343.16 | Decrease |
| Solomon Islands | 2474.69 | 0.43 | 724.71 | 1749.98 | Increase |
| Somalia | 3386.71 | 0.08 | 3382.99 | 3.72 | Decrease |
| South Africa | 1688.96 | 0.68 | 501.77 | 1187.20 | Decrease |
| South Sudan | 2705.04 | 0.28 | 878.01 | 1827.03 | Decrease |
| Spain | 707.76 | 0.77 | 482.29 | 225.48 | Decrease |
| Sri Lanka | 2151.40 | 0.70 | 501.60 | 1649.80 | Decrease |
| Sudan | 2066.97 | 0.54 | 556.36 | 1510.61 | Decrease |
| Suriname | 1671.61 | 0.63 | 541.06 | 1130.55 | Decrease |
| Sweden | 461.50 | 0.89 | 317.78 | 143.72 | Decrease |
| Switzerland | 811.58 | 0.93 | 321.46 | 490.12 | Increase |
| Syrian Arab Republic | 1891.97 | 0.62 | 545.47 | 1346.50 | Decrease |
| Taiwan (Province of China) | 2726.09 | 0.87 | 329.13 | 2396.96 | Decrease |
| Tajikistan | 6485.90 | 0.54 | 555.58 | 5930.32 | Decrease |
| Thailand | 2032.52 | 0.68 | 502.09 | 1530.43 | Decrease |
| Timor-Leste | 2488.90 | 0.44 | 723.86 | 1765.04 | Decrease |
| Togo | 3150.20 | 0.41 | 724.93 | 2425.27 | Decrease |
| Tokelau | 1680.61 | 0.69 | 501.55 | 1179.06 | Decrease |
| Tonga | 2230.03 | 0.63 | 544.91 | 1685.12 | Increase |
| Trinidad and Tobago | 1347.97 | 0.77 | 482.09 | 865.88 | Decrease |
| Tunisia | 1793.92 | 0.68 | 502.22 | 1291.70 | Decrease |
| Turkey | 1008.90 | 0.71 | 494.73 | 514.17 | Decrease |
| Turkmenistan | 6547.41 | 0.68 | 501.71 | 6045.69 | Increase |
| Tuvalu | 2171.88 | 0.58 | 557.14 | 1614.73 | Increase |
| Uganda | 2974.37 | 0.42 | 724.40 | 2249.97 | Decrease |
| Ukraine | 3056.46 | 0.76 | 482.07 | 2574.39 | Increase |
| United Arab Emirates | 1353.43 | 0.85 | 445.57 | 907.86 | Decrease |
| United Kingdom | 794.26 | 0.86 | 418.45 | 375.81 | Decrease |
| United Republic of Tanzania | 3624.57 | 0.45 | 727.43 | 2897.14 | Decrease |
| United States Virgin Islands | 1350.10 | 0.82 | 459.18 | 890.92 | Decrease |
| United States of America | 904.83 | 0.86 | 420.23 | 484.59 | Decrease |
| Uruguay | 655.46 | 0.72 | 490.80 | 164.67 | Decrease |
| Uzbekistan | 6338.28 | 0.66 | 503.45 | 5834.83 | Increase |
| Vanuatu | 2531.11 | 0.47 | 724.31 | 1806.80 | Increase |
| Venezuela (Bolivarian Republic of) | 1513.99 | 0.60 | 554.86 | 959.13 | Decrease |
| Viet Nam | 2262.94 | 0.63 | 544.82 | 1718.12 | Decrease |
| Yemen | 2641.61 | 0.45 | 724.50 | 1917.11 | Decrease |
| Zambia | 2564.36 | 0.51 | 723.65 | 1840.71 | Decrease |
| Zimbabwe | 2493.53 | 0.47 | 723.87 | 1769.66 | Increase |
