# Supplementary material for: Global, regional, and national burden of chronic hepatitis C including cirrhosis from 1990 to 2021, alongside projections for the next 15 years
Source: Medicine (Baltimore). 2026 Oct 16;105(42):e51118. doi: 10.1097/MD.0000000000051118 (PMC13681144; doi:10.1097/MD.0000000000051118)
Supplement: Supplementary file 15 [file medi-105-e51118-s015.docx]

Supplementary Table S14. Frontier analysis based on SDI and age-standardized mortality rate of chronic hepatitis C including cirrhosis at 204 countries and territories.

| location | The age-standardized mortality rate/100000 in 2021 | SDI in 2021 | frontier | effective difference | trend |
| --- | --- | --- | --- | --- | --- |
| Afghanistan | 15.66 | 0.34 | 2.46 | 13.19 | Decrease |
| Albania | 1.25 | 0.71 | 0.32 | 0.94 | Decrease |
| Algeria | 5.41 | 0.66 | 0.43 | 4.99 | Decrease |
| American Samoa | 2.66 | 0.72 | 0.31 | 2.34 | Decrease |
| Andorra | 1.39 | 0.87 | 0.31 | 1.08 | Decrease |
| Angola | 14.68 | 0.45 | 1.87 | 12.81 | Decrease |
| Antigua and Barbuda | 2.46 | 0.75 | 0.31 | 2.15 | Decrease |
| Argentina | 4.15 | 0.72 | 0.31 | 3.84 | Decrease |
| Armenia | 3.71 | 0.70 | 0.33 | 3.38 | Decrease |
| Australia | 1.65 | 0.84 | 0.32 | 1.33 | Decrease |
| Austria | 1.86 | 0.85 | 0.31 | 1.54 | Decrease |
| Azerbaijan | 6.41 | 0.69 | 0.34 | 6.08 | Decrease |
| Bahamas | 3.15 | 0.81 | 0.32 | 2.84 | Decrease |
| Bahrain | 8.43 | 0.75 | 0.31 | 8.12 | Decrease |
| Bangladesh | 5.74 | 0.49 | 1.89 | 3.85 | Decrease |
| Barbados | 1.34 | 0.75 | 0.32 | 1.02 | Decrease |
| Belarus | 3.41 | 0.78 | 0.31 | 3.09 | Increase |
| Belgium | 1.72 | 0.85 | 0.31 | 1.40 | Decrease |
| Belize | 4.47 | 0.61 | 0.50 | 3.97 | Decrease |
| Benin | 7.91 | 0.37 | 1.98 | 5.92 | Decrease |
| Bermuda | 1.10 | 0.82 | 0.31 | 0.79 | Decrease |
| Bhutan | 6.38 | 0.47 | 1.86 | 4.52 | Decrease |
| Bolivia (Plurinational State of) | 2.52 | 0.60 | 0.48 | 2.03 | Decrease |
| Bosnia and Herzegovina | 1.80 | 0.72 | 0.31 | 1.49 | Decrease |
| Botswana | 5.51 | 0.64 | 0.45 | 5.06 | Decrease |
| Brazil | 3.32 | 0.65 | 0.40 | 2.93 | Decrease |
| Brunei Darussalam | 2.07 | 0.81 | 0.31 | 1.75 | Decrease |
| Bulgaria | 4.01 | 0.77 | 0.31 | 3.70 | Increase |
| Burkina Faso | 7.31 | 0.29 | 2.91 | 4.40 | Decrease |
| Burundi | 12.90 | 0.29 | 2.95 | 9.95 | Decrease |
| Cabo Verde | 5.53 | 0.53 | 1.65 | 3.87 | Decrease |
| Cambodia | 15.43 | 0.47 | 1.86 | 13.58 | Decrease |
| Cameroon | 7.16 | 0.48 | 1.87 | 5.29 | Decrease |
| Canada | 3.44 | 0.87 | 0.31 | 3.12 | Decrease |
| Central African Republic | 21.97 | 0.31 | 2.80 | 19.17 | Decrease |
| Chad | 12.49 | 0.24 | 3.17 | 9.31 | Decrease |
| Chile | 6.11 | 0.77 | 0.32 | 5.79 | Decrease |
| China | 0.82 | 0.72 | 0.31 | 0.50 | Decrease |
| Colombia | 1.94 | 0.66 | 0.39 | 1.55 | Decrease |
| Comoros | 10.12 | 0.48 | 1.89 | 8.23 | Decrease |
| Congo | 13.64 | 0.58 | 0.53 | 13.11 | Decrease |
| Cook Islands | 0.32 | 0.78 | 0.31 | 0.00 | Decrease |
| Costa Rica | 4.77 | 0.70 | 0.32 | 4.45 | Decrease |
| Croatia | 2.25 | 0.80 | 0.31 | 1.93 | Decrease |
| Cuba | 2.19 | 0.67 | 0.38 | 1.81 | Decrease |
| Cyprus | 1.19 | 0.84 | 0.31 | 0.88 | Decrease |
| Czechia | 1.73 | 0.83 | 0.31 | 1.41 | Decrease |
| C么te d'Ivoire | 7.49 | 0.43 | 1.88 | 5.61 | Decrease |
| Democratic People's Republic of Korea | 2.57 | 0.57 | 0.66 | 1.91 | Decrease |
| Democratic Republic of the Congo | 13.36 | 0.38 | 1.92 | 11.45 | Decrease |
| Denmark | 1.47 | 0.90 | 0.32 | 1.16 | Decrease |
| Djibouti | 10.42 | 0.49 | 1.89 | 8.53 | Decrease |
| Dominica | 2.45 | 0.75 | 0.32 | 2.13 | Decrease |
| Dominican Republic | 5.52 | 0.62 | 0.47 | 5.06 | Decrease |
| Ecuador | 1.56 | 0.66 | 0.39 | 1.16 | Decrease |
| Egypt | 45.20 | 0.61 | 0.47 | 44.73 | Decrease |
| El Salvador | 7.52 | 0.56 | 1.50 | 6.02 | Decrease |
| Equatorial Guinea | 8.02 | 0.66 | 0.39 | 7.63 | Decrease |
| Eritrea | 15.58 | 0.40 | 1.91 | 13.67 | Decrease |
| Estonia | 3.30 | 0.84 | 0.31 | 2.98 | Increase |
| Eswatini | 10.31 | 0.59 | 0.51 | 9.80 | Decrease |
| Ethiopia | 9.85 | 0.36 | 2.14 | 7.70 | Decrease |
| Fiji | 1.92 | 0.68 | 0.34 | 1.58 | Decrease |
| Finland | 2.13 | 0.86 | 0.32 | 1.81 | Decrease |
| France | 1.48 | 0.84 | 0.31 | 1.17 | Decrease |
| Gabon | 12.20 | 0.63 | 0.45 | 11.75 | Decrease |
| Gambia | 7.17 | 0.41 | 1.91 | 5.27 | Decrease |
| Georgia | 4.95 | 0.73 | 0.31 | 4.64 | Decrease |
| Germany | 2.00 | 0.90 | 0.31 | 1.69 | Decrease |
| Ghana | 7.94 | 0.56 | 0.59 | 7.35 | Decrease |
| Greece | 1.05 | 0.79 | 0.31 | 0.74 | Decrease |
| Greenland | 4.43 | 0.83 | 0.31 | 4.11 | Decrease |
| Grenada | 2.87 | 0.67 | 0.34 | 2.53 | Decrease |
| Guam | 3.40 | 0.80 | 0.31 | 3.09 | Decrease |
| Guatemala | 14.26 | 0.54 | 1.61 | 12.65 | Decrease |
| Guinea | 9.11 | 0.34 | 2.46 | 6.65 | Decrease |
| Guinea-Bissau | 11.79 | 0.35 | 2.38 | 9.41 | Decrease |
| Guyana | 7.29 | 0.65 | 0.41 | 6.88 | Decrease |
| Haiti | 8.20 | 0.45 | 1.88 | 6.32 | Decrease |
| Honduras | 10.80 | 0.51 | 1.72 | 9.08 | Decrease |
| Hungary | 3.08 | 0.79 | 0.31 | 2.77 | Decrease |
| Iceland | 0.48 | 0.88 | 0.31 | 0.17 | Decrease |
| India | 4.38 | 0.58 | 0.54 | 3.84 | Decrease |
| Indonesia | 17.61 | 0.66 | 0.41 | 17.20 | Decrease |
| Iran (Islamic Republic of) | 1.87 | 0.70 | 0.32 | 1.55 | Decrease |
| Iraq | 5.58 | 0.66 | 0.37 | 5.21 | Decrease |
| Ireland | 0.79 | 0.87 | 0.31 | 0.47 | Decrease |
| Israel | 0.94 | 0.81 | 0.31 | 0.63 | Decrease |
| Italy | 2.08 | 0.81 | 0.31 | 1.76 | Decrease |
| Jamaica | 1.01 | 0.68 | 0.34 | 0.67 | Decrease |
| Japan | 2.67 | 0.87 | 0.31 | 2.35 | Decrease |
| Jordan | 3.20 | 0.73 | 0.31 | 2.89 | Decrease |
| Kazakhstan | 9.39 | 0.73 | 0.31 | 9.08 | Increase |
| Kenya | 16.08 | 0.52 | 1.65 | 14.43 | Decrease |
| Kiribati | 6.06 | 0.53 | 1.83 | 4.23 | Decrease |
| Kuwait | 2.58 | 0.85 | 0.31 | 2.26 | Decrease |
| Kyrgyzstan | 9.15 | 0.60 | 0.48 | 8.67 | Decrease |
| Lao People's Democratic Republic | 7.04 | 0.49 | 1.86 | 5.18 | Decrease |
| Latvia | 3.34 | 0.83 | 0.31 | 3.02 | Increase |
| Lebanon | 4.65 | 0.74 | 0.31 | 4.34 | Decrease |
| Lesotho | 11.71 | 0.51 | 1.82 | 9.89 | Increase |
| Liberia | 8.44 | 0.35 | 2.37 | 6.08 | Decrease |
| Libya | 8.15 | 0.73 | 0.31 | 7.84 | Decrease |
| Lithuania | 6.26 | 0.86 | 0.31 | 5.95 | Increase |
| Luxembourg | 1.58 | 0.88 | 0.31 | 1.26 | Decrease |
| Madagascar | 10.33 | 0.40 | 1.92 | 8.42 | Decrease |
| Malawi | 15.13 | 0.38 | 1.92 | 13.21 | Decrease |
| Malaysia | 3.90 | 0.74 | 0.31 | 3.59 | Decrease |
| Maldives | 2.27 | 0.65 | 0.45 | 1.82 | Decrease |
| Mali | 8.70 | 0.27 | 3.03 | 5.67 | Decrease |
| Malta | 0.89 | 0.80 | 0.31 | 0.58 | Decrease |
| Marshall Islands | 5.68 | 0.57 | 0.54 | 5.14 | Decrease |
| Mauritania | 6.33 | 0.50 | 1.86 | 4.47 | Decrease |
| Mauritius | 3.41 | 0.72 | 0.32 | 3.10 | Decrease |
| Mexico | 9.96 | 0.66 | 0.37 | 9.58 | Decrease |
| Micronesia (Federated States of) | 4.69 | 0.59 | 0.52 | 4.18 | Decrease |
| Monaco | 1.78 | 0.91 | 0.31 | 1.47 | Decrease |
| Mongolia | 9.19 | 0.62 | 0.47 | 8.72 | Decrease |
| Montenegro | 1.24 | 0.80 | 0.31 | 0.92 | Decrease |
| Morocco | 6.57 | 0.56 | 1.49 | 5.08 | Decrease |
| Mozambique | 7.02 | 0.33 | 2.54 | 4.48 | Decrease |
| Myanmar | 7.17 | 0.53 | 1.61 | 5.56 | Decrease |
| Namibia | 8.42 | 0.62 | 0.46 | 7.96 | Decrease |
| Nauru | 5.63 | 0.63 | 0.46 | 5.17 | Decrease |
| Nepal | 2.62 | 0.43 | 1.86 | 0.76 | Decrease |
| Netherlands | 0.72 | 0.89 | 0.31 | 0.41 | Decrease |
| New Zealand | 0.90 | 0.85 | 0.31 | 0.59 | Decrease |
| Nicaragua | 7.19 | 0.52 | 1.66 | 5.53 | Decrease |
| Niger | 10.38 | 0.17 | 8.95 | 1.43 | Decrease |
| Nigeria | 7.01 | 0.50 | 1.82 | 5.20 | Decrease |
| Niue | 3.19 | 0.73 | 0.31 | 2.88 | Decrease |
| North Macedonia | 1.42 | 0.75 | 0.32 | 1.10 | Decrease |
| Northern Mariana Islands | 4.50 | 0.77 | 0.31 | 4.18 | Decrease |
| Norway | 0.64 | 0.92 | 0.31 | 0.32 | Decrease |
| Oman | 5.77 | 0.77 | 0.31 | 5.46 | Decrease |
| Pakistan | 11.91 | 0.50 | 1.81 | 10.10 | Decrease |
| Palau | 3.31 | 0.75 | 0.31 | 3.00 | Decrease |
| Palestine | 6.09 | 0.63 | 0.46 | 5.64 | Decrease |
| Panama | 3.05 | 0.71 | 0.32 | 2.73 | Decrease |
| Papua New Guinea | 1.86 | 0.42 | 1.86 | 0.00 | Decrease |
| Paraguay | 3.27 | 0.64 | 0.45 | 2.82 | Increase |
| Peru | 1.76 | 0.66 | 0.38 | 1.38 | Decrease |
| Philippines | 4.53 | 0.65 | 0.45 | 4.08 | Decrease |
| Poland | 3.79 | 0.81 | 0.31 | 3.48 | Increase |
| Portugal | 1.63 | 0.74 | 0.31 | 1.32 | Decrease |
| Puerto Rico | 2.15 | 0.83 | 0.31 | 1.83 | Decrease |
| Qatar | 7.39 | 0.85 | 0.31 | 7.07 | Decrease |
| Republic of Korea | 1.35 | 0.89 | 0.31 | 1.03 | Decrease |
| Republic of Moldova | 11.47 | 0.73 | 0.32 | 11.16 | Decrease |
| Romania | 5.78 | 0.77 | 0.31 | 5.46 | Decrease |
| Russian Federation | 3.40 | 0.81 | 0.31 | 3.08 | Increase |
| Rwanda | 14.18 | 0.44 | 1.87 | 12.31 | Decrease |
| Saint Kitts and Nevis | 3.34 | 0.75 | 0.31 | 3.02 | Decrease |
| Saint Lucia | 2.99 | 0.67 | 0.36 | 2.63 | Decrease |
| Saint Vincent and the Grenadines | 2.90 | 0.64 | 0.46 | 2.44 | Decrease |
| Samoa | 3.34 | 0.59 | 0.49 | 2.85 | Decrease |
| San Marino | 1.40 | 0.89 | 0.32 | 1.09 | Decrease |
| Sao Tome and Principe | 12.65 | 0.51 | 1.81 | 10.84 | Decrease |
| Saudi Arabia | 15.61 | 0.82 | 0.31 | 15.29 | Decrease |
| Senegal | 6.60 | 0.41 | 1.92 | 4.68 | Decrease |
| Serbia | 1.72 | 0.79 | 0.31 | 1.41 | Decrease |
| Seychelles | 6.16 | 0.73 | 0.31 | 5.84 | Decrease |
| Sierra Leone | 7.71 | 0.36 | 2.26 | 5.46 | Decrease |
| Singapore | 0.44 | 0.86 | 0.31 | 0.13 | Decrease |
| Slovakia | 3.17 | 0.81 | 0.31 | 2.85 | Decrease |
| Slovenia | 1.79 | 0.84 | 0.31 | 1.48 | Decrease |
| Solomon Islands | 6.94 | 0.43 | 1.86 | 5.07 | Decrease |
| Somalia | 14.55 | 0.08 | 14.55 | 0.00 | Decrease |
| South Africa | 4.41 | 0.68 | 0.34 | 4.07 | Decrease |
| South Sudan | 14.66 | 0.28 | 2.99 | 11.67 | Decrease |
| Spain | 1.55 | 0.77 | 0.32 | 1.23 | Decrease |
| Sri Lanka | 3.67 | 0.70 | 0.32 | 3.35 | Decrease |
| Sudan | 8.70 | 0.54 | 1.59 | 7.11 | Decrease |
| Suriname | 3.84 | 0.63 | 0.46 | 3.38 | Decrease |
| Sweden | 0.93 | 0.89 | 0.32 | 0.61 | Decrease |
| Switzerland | 0.88 | 0.93 | 0.31 | 0.56 | Decrease |
| Syrian Arab Republic | 7.67 | 0.62 | 0.46 | 7.21 | Decrease |
| Taiwan (Province of China) | 3.68 | 0.87 | 0.31 | 3.36 | Decrease |
| Tajikistan | 6.52 | 0.54 | 1.59 | 4.92 | Decrease |
| Thailand | 4.97 | 0.68 | 0.34 | 4.64 | Decrease |
| Timor-Leste | 5.99 | 0.44 | 1.86 | 4.13 | Decrease |
| Togo | 7.63 | 0.41 | 1.91 | 5.72 | Decrease |
| Tokelau | 3.17 | 0.69 | 0.34 | 2.83 | Decrease |
| Tonga | 5.36 | 0.63 | 0.46 | 4.90 | Decrease |
| Trinidad and Tobago | 1.70 | 0.77 | 0.32 | 1.38 | Decrease |
| Tunisia | 5.46 | 0.68 | 0.34 | 5.12 | Decrease |
| Turkey | 2.38 | 0.71 | 0.31 | 2.07 | Decrease |
| Turkmenistan | 14.72 | 0.68 | 0.34 | 14.38 | Increase |
| Tuvalu | 4.43 | 0.58 | 0.54 | 3.89 | Decrease |
| Uganda | 9.81 | 0.42 | 1.87 | 7.94 | Decrease |
| Ukraine | 5.84 | 0.76 | 0.31 | 5.52 | Increase |
| United Arab Emirates | 5.76 | 0.85 | 0.31 | 5.45 | Decrease |
| United Kingdom | 0.70 | 0.86 | 0.31 | 0.39 | Increase |
| United Republic of Tanzania | 9.97 | 0.45 | 1.89 | 8.07 | Decrease |
| United States Virgin Islands | 2.04 | 0.82 | 0.31 | 1.72 | Decrease |
| United States of America | 4.94 | 0.86 | 0.31 | 4.63 | Increase |
| Uruguay | 2.78 | 0.72 | 0.31 | 2.47 | Decrease |
| Uzbekistan | 7.54 | 0.66 | 0.39 | 7.16 | Decrease |
| Vanuatu | 6.22 | 0.47 | 1.90 | 4.32 | Decrease |
| Venezuela (Bolivarian Republic of) | 4.44 | 0.60 | 0.50 | 3.94 | Decrease |
| Viet Nam | 6.46 | 0.63 | 0.46 | 6.00 | Decrease |
| Yemen | 10.00 | 0.45 | 1.88 | 8.12 | Decrease |
| Zambia | 13.25 | 0.51 | 1.88 | 11.38 | Decrease |
| Zimbabwe | 10.29 | 0.47 | 1.90 | 8.38 | Increase |
