# Supplementary material for: Global, regional, and national burden of chronic hepatitis C including cirrhosis from 1990 to 2021, alongside projections for the next 15 years
Source: Medicine (Baltimore). 2026 Oct 16;105(42):e51118. doi: 10.1097/MD.0000000000051118 (PMC13681144; doi:10.1097/MD.0000000000051118)
Supplement: Supplementary file 16 [file medi-105-e51118-s016.docx]

Supplementary Table S15. Frontier analysis based on SDI and age-standardized DALYs rate of chronic hepatitis C including cirrhosis at 204 countries and territories.

| location | The age-standardized DALYs rate/100000 in 2021 | SDI in 2021 | frontier | effective difference | trend |
| --- | --- | --- | --- | --- | --- |
| Afghanistan | 386.49 | 0.34 | 88.49 | 298.00 | Decrease |
| Albania | 36.66 | 0.71 | 12.41 | 24.26 | Decrease |
| Algeria | 114.43 | 0.66 | 14.91 | 99.52 | Decrease |
| American Samoa | 93.05 | 0.72 | 12.41 | 80.64 | Decrease |
| Andorra | 42.02 | 0.87 | 12.29 | 29.72 | Decrease |
| Angola | 459.02 | 0.45 | 65.77 | 393.25 | Decrease |
| Antigua and Barbuda | 67.20 | 0.75 | 12.41 | 54.79 | Decrease |
| Argentina | 120.68 | 0.72 | 12.29 | 108.38 | Decrease |
| Armenia | 101.01 | 0.70 | 12.64 | 88.37 | Decrease |
| Australia | 50.85 | 0.84 | 12.29 | 38.57 | Decrease |
| Austria | 54.97 | 0.85 | 12.29 | 42.67 | Decrease |
| Azerbaijan | 171.83 | 0.69 | 12.92 | 158.91 | Decrease |
| Bahamas | 102.09 | 0.81 | 12.28 | 89.81 | Decrease |
| Bahrain | 164.83 | 0.75 | 12.29 | 152.53 | Decrease |
| Bangladesh | 158.40 | 0.49 | 60.82 | 97.59 | Decrease |
| Barbados | 39.96 | 0.75 | 12.29 | 27.67 | Decrease |
| Belarus | 127.07 | 0.78 | 12.28 | 114.79 | Increase |
| Belgium | 51.94 | 0.85 | 12.32 | 39.63 | Decrease |
| Belize | 139.22 | 0.61 | 18.29 | 120.93 | Decrease |
| Benin | 233.14 | 0.37 | 69.36 | 163.79 | Decrease |
| Bermuda | 32.58 | 0.82 | 12.30 | 20.27 | Decrease |
| Bhutan | 190.64 | 0.47 | 65.66 | 124.98 | Decrease |
| Bolivia (Plurinational State of) | 69.07 | 0.60 | 18.57 | 50.50 | Decrease |
| Bosnia and Herzegovina | 54.69 | 0.72 | 12.80 | 41.89 | Decrease |
| Botswana | 163.51 | 0.64 | 17.13 | 146.39 | Decrease |
| Brazil | 109.39 | 0.65 | 17.10 | 92.29 | Decrease |
| Brunei Darussalam | 63.64 | 0.81 | 12.30 | 51.35 | Decrease |
| Bulgaria | 136.09 | 0.77 | 12.34 | 123.76 | Increase |
| Burkina Faso | 200.50 | 0.29 | 99.86 | 100.64 | Decrease |
| Burundi | 376.99 | 0.29 | 96.52 | 280.47 | Decrease |
| Cabo Verde | 161.48 | 0.53 | 48.94 | 112.54 | Decrease |
| Cambodia | 470.11 | 0.47 | 66.25 | 403.86 | Decrease |
| Cameroon | 216.20 | 0.48 | 65.18 | 151.02 | Decrease |
| Canada | 104.76 | 0.87 | 12.16 | 92.60 | Decrease |
| Central African Republic | 716.94 | 0.31 | 91.64 | 625.29 | Decrease |
| Chad | 367.63 | 0.24 | 106.54 | 261.09 | Increase |
| Chile | 180.55 | 0.77 | 12.36 | 168.20 | Decrease |
| China | 23.73 | 0.72 | 12.32 | 11.41 | Decrease |
| Colombia | 52.54 | 0.66 | 16.44 | 36.10 | Decrease |
| Comoros | 293.93 | 0.48 | 64.71 | 229.22 | Decrease |
| Congo | 430.68 | 0.58 | 20.36 | 410.32 | Decrease |
| Cook Islands | 12.50 | 0.78 | 12.31 | 0.19 | Decrease |
| Costa Rica | 143.11 | 0.70 | 12.62 | 130.49 | Decrease |
| Croatia | 67.76 | 0.80 | 12.28 | 55.48 | Decrease |
| Cuba | 69.70 | 0.67 | 14.90 | 54.80 | Decrease |
| Cyprus | 34.13 | 0.84 | 12.30 | 21.84 | Decrease |
| Czechia | 56.90 | 0.83 | 12.30 | 44.60 | Decrease |
| C么te d'Ivoire | 224.32 | 0.43 | 65.25 | 159.07 | Decrease |
| Democratic People's Republic of Korea | 81.00 | 0.57 | 22.86 | 58.14 | Decrease |
| Democratic Republic of the Congo | 430.23 | 0.38 | 69.27 | 360.96 | Decrease |
| Denmark | 44.76 | 0.90 | 12.11 | 32.65 | Decrease |
| Djibouti | 302.57 | 0.49 | 63.61 | 238.96 | Decrease |
| Dominica | 72.20 | 0.75 | 12.44 | 59.76 | Decrease |
| Dominican Republic | 161.63 | 0.62 | 17.79 | 143.84 | Decrease |
| Ecuador | 42.37 | 0.66 | 14.81 | 27.55 | Decrease |
| Egypt | 976.16 | 0.61 | 19.69 | 956.47 | Decrease |
| El Salvador | 248.30 | 0.56 | 43.75 | 204.55 | Decrease |
| Equatorial Guinea | 255.75 | 0.66 | 14.86 | 240.89 | Decrease |
| Eritrea | 470.78 | 0.40 | 66.57 | 404.21 | Decrease |
| Estonia | 120.07 | 0.84 | 12.29 | 107.78 | Increase |
| Eswatini | 335.35 | 0.59 | 19.49 | 315.86 | Decrease |
| Ethiopia | 282.90 | 0.36 | 77.14 | 205.77 | Decrease |
| Fiji | 66.31 | 0.68 | 13.43 | 52.88 | Decrease |
| Finland | 69.26 | 0.86 | 12.29 | 56.98 | Decrease |
| France | 45.21 | 0.84 | 12.37 | 32.84 | Decrease |
| Gabon | 381.85 | 0.63 | 17.22 | 364.63 | Decrease |
| Gambia | 217.37 | 0.41 | 66.36 | 151.01 | Decrease |
| Georgia | 165.41 | 0.73 | 12.46 | 152.95 | Decrease |
| Germany | 61.57 | 0.90 | 12.17 | 49.39 | Decrease |
| Ghana | 219.77 | 0.56 | 25.90 | 193.87 | Decrease |
| Greece | 31.07 | 0.79 | 12.29 | 18.78 | Decrease |
| Greenland | 161.55 | 0.83 | 12.32 | 149.23 | Decrease |
| Grenada | 87.66 | 0.67 | 13.30 | 74.36 | Decrease |
| Guam | 124.12 | 0.80 | 12.34 | 111.79 | Decrease |
| Guatemala | 475.76 | 0.54 | 48.49 | 427.27 | Decrease |
| Guinea | 267.15 | 0.34 | 87.66 | 179.49 | Decrease |
| Guinea-Bissau | 364.54 | 0.35 | 84.36 | 280.18 | Decrease |
| Guyana | 243.39 | 0.65 | 15.99 | 227.40 | Decrease |
| Haiti | 245.58 | 0.45 | 64.99 | 180.59 | Decrease |
| Honduras | 316.50 | 0.51 | 54.96 | 261.54 | Decrease |
| Hungary | 99.99 | 0.79 | 12.37 | 87.62 | Decrease |
| Iceland | 16.22 | 0.88 | 12.06 | 4.17 | Decrease |
| India | 139.04 | 0.58 | 35.74 | 103.30 | Decrease |
| Indonesia | 468.08 | 0.66 | 15.79 | 452.29 | Decrease |
| Iran (Islamic Republic of) | 45.22 | 0.70 | 12.56 | 32.65 | Decrease |
| Iraq | 136.26 | 0.66 | 14.44 | 121.83 | Decrease |
| Ireland | 26.02 | 0.87 | 12.07 | 13.95 | Decrease |
| Israel | 26.48 | 0.81 | 12.37 | 14.11 | Decrease |
| Italy | 54.27 | 0.81 | 12.29 | 41.98 | Decrease |
| Jamaica | 30.43 | 0.68 | 13.02 | 17.41 | Decrease |
| Japan | 73.73 | 0.87 | 12.07 | 61.67 | Decrease |
| Jordan | 72.94 | 0.73 | 12.33 | 60.62 | Decrease |
| Kazakhstan | 283.12 | 0.73 | 12.29 | 270.83 | Increase |
| Kenya | 445.55 | 0.52 | 61.13 | 384.42 | Decrease |
| Kiribati | 221.81 | 0.53 | 58.82 | 162.99 | Decrease |
| Kuwait | 54.65 | 0.85 | 12.44 | 42.21 | Decrease |
| Kyrgyzstan | 321.75 | 0.60 | 18.52 | 303.22 | Increase |
| Lao People's Democratic Republic | 214.29 | 0.49 | 60.81 | 153.47 | Decrease |
| Latvia | 126.19 | 0.83 | 12.30 | 113.90 | Increase |
| Lebanon | 97.53 | 0.74 | 12.28 | 85.25 | Decrease |
| Lesotho | 372.40 | 0.51 | 57.05 | 315.35 | Increase |
| Liberia | 245.50 | 0.35 | 84.31 | 161.18 | Decrease |
| Libya | 196.30 | 0.73 | 12.28 | 184.02 | Decrease |
| Lithuania | 238.43 | 0.86 | 12.30 | 226.14 | Increase |
| Luxembourg | 46.01 | 0.88 | 12.04 | 33.98 | Decrease |
| Madagascar | 308.65 | 0.40 | 67.03 | 241.62 | Decrease |
| Malawi | 455.88 | 0.38 | 68.00 | 387.88 | Decrease |
| Malaysia | 109.13 | 0.74 | 12.29 | 96.84 | Decrease |
| Maldives | 62.23 | 0.65 | 15.64 | 46.59 | Decrease |
| Mali | 254.92 | 0.27 | 101.17 | 153.75 | Decrease |
| Malta | 28.94 | 0.80 | 12.29 | 16.65 | Decrease |
| Marshall Islands | 205.19 | 0.57 | 25.41 | 179.78 | Decrease |
| Mauritania | 176.10 | 0.50 | 62.48 | 113.62 | Decrease |
| Mauritius | 114.71 | 0.72 | 12.30 | 102.41 | Decrease |
| Mexico | 318.80 | 0.66 | 14.26 | 304.53 | Decrease |
| Micronesia (Federated States of) | 172.63 | 0.59 | 19.55 | 153.08 | Decrease |
| Monaco | 58.49 | 0.91 | 12.17 | 46.32 | Decrease |
| Mongolia | 257.81 | 0.62 | 18.54 | 239.27 | Decrease |
| Montenegro | 40.53 | 0.80 | 12.28 | 28.24 | Decrease |
| Morocco | 152.02 | 0.56 | 43.41 | 108.61 | Decrease |
| Mozambique | 200.00 | 0.33 | 90.39 | 109.61 | Increase |
| Myanmar | 262.03 | 0.53 | 49.40 | 212.63 | Decrease |
| Namibia | 253.54 | 0.62 | 18.22 | 235.31 | Decrease |
| Nauru | 205.72 | 0.63 | 17.58 | 188.14 | Decrease |
| Nepal | 81.90 | 0.43 | 65.88 | 16.02 | Decrease |
| Netherlands | 20.64 | 0.89 | 12.24 | 8.40 | Decrease |
| New Zealand | 26.28 | 0.85 | 12.34 | 13.94 | Decrease |
| Nicaragua | 233.93 | 0.52 | 50.17 | 183.75 | Decrease |
| Niger | 296.10 | 0.17 | 246.16 | 49.94 | Decrease |
| Nigeria | 196.69 | 0.50 | 58.85 | 137.85 | Decrease |
| Niue | 113.66 | 0.73 | 12.30 | 101.36 | Decrease |
| North Macedonia | 43.04 | 0.75 | 12.28 | 30.76 | Decrease |
| Northern Mariana Islands | 143.14 | 0.77 | 12.29 | 130.86 | Decrease |
| Norway | 19.10 | 0.92 | 12.05 | 7.05 | Decrease |
| Oman | 129.09 | 0.77 | 12.29 | 116.80 | Decrease |
| Pakistan | 368.98 | 0.50 | 60.23 | 308.75 | Decrease |
| Palau | 124.49 | 0.75 | 12.28 | 112.20 | Decrease |
| Palestine | 128.78 | 0.63 | 17.28 | 111.50 | Decrease |
| Panama | 90.26 | 0.71 | 12.47 | 77.79 | Decrease |
| Papua New Guinea | 64.73 | 0.42 | 64.73 | 0.00 | Decrease |
| Paraguay | 103.31 | 0.64 | 17.17 | 86.14 | Increase |
| Peru | 50.00 | 0.66 | 14.41 | 35.59 | Decrease |
| Philippines | 141.81 | 0.65 | 16.62 | 125.20 | Decrease |
| Poland | 137.05 | 0.81 | 12.49 | 124.57 | Increase |
| Portugal | 51.05 | 0.74 | 12.29 | 38.76 | Decrease |
| Puerto Rico | 64.44 | 0.83 | 12.31 | 52.13 | Decrease |
| Qatar | 149.77 | 0.85 | 12.28 | 137.49 | Decrease |
| Republic of Korea | 38.90 | 0.89 | 12.24 | 26.66 | Decrease |
| Republic of Moldova | 384.37 | 0.73 | 12.35 | 372.02 | Decrease |
| Romania | 182.35 | 0.77 | 12.40 | 169.95 | Decrease |
| Russian Federation | 131.21 | 0.81 | 12.34 | 118.87 | Increase |
| Rwanda | 405.66 | 0.44 | 65.80 | 339.86 | Decrease |
| Saint Kitts and Nevis | 95.49 | 0.75 | 12.30 | 83.20 | Decrease |
| Saint Lucia | 89.46 | 0.67 | 14.65 | 74.81 | Decrease |
| Saint Vincent and the Grenadines | 92.41 | 0.64 | 17.30 | 75.12 | Decrease |
| Samoa | 117.20 | 0.59 | 18.94 | 98.26 | Decrease |
| San Marino | 39.06 | 0.89 | 12.11 | 26.95 | Decrease |
| Sao Tome and Principe | 358.01 | 0.51 | 56.89 | 301.11 | Decrease |
| Saudi Arabia | 356.70 | 0.82 | 12.29 | 344.41 | Decrease |
| Senegal | 194.04 | 0.41 | 66.75 | 127.29 | Decrease |
| Serbia | 53.49 | 0.79 | 12.28 | 41.21 | Decrease |
| Seychelles | 191.09 | 0.73 | 12.34 | 178.76 | Decrease |
| Sierra Leone | 228.78 | 0.36 | 77.66 | 151.12 | Decrease |
| Singapore | 12.03 | 0.86 | 12.03 | 0.00 | Decrease |
| Slovakia | 105.93 | 0.81 | 12.28 | 93.65 | Decrease |
| Slovenia | 52.11 | 0.84 | 12.35 | 39.76 | Decrease |
| Solomon Islands | 244.61 | 0.43 | 65.04 | 179.57 | Decrease |
| Somalia | 438.11 | 0.08 | 438.11 | 0.00 | Decrease |
| South Africa | 138.27 | 0.68 | 13.45 | 124.82 | Decrease |
| South Sudan | 431.74 | 0.28 | 102.12 | 329.62 | Decrease |
| Spain | 44.11 | 0.77 | 12.42 | 31.69 | Decrease |
| Sri Lanka | 112.36 | 0.70 | 12.61 | 99.76 | Decrease |
| Sudan | 210.13 | 0.54 | 48.37 | 161.76 | Decrease |
| Suriname | 119.66 | 0.63 | 17.22 | 102.44 | Decrease |
| Sweden | 25.78 | 0.89 | 12.03 | 13.75 | Decrease |
| Switzerland | 25.27 | 0.93 | 12.16 | 13.12 | Decrease |
| Syrian Arab Republic | 166.05 | 0.62 | 17.46 | 148.59 | Decrease |
| Taiwan (Province of China) | 113.55 | 0.87 | 12.21 | 101.35 | Decrease |
| Tajikistan | 206.99 | 0.54 | 48.18 | 158.81 | Decrease |
| Thailand | 170.69 | 0.68 | 13.02 | 157.67 | Decrease |
| Timor-Leste | 179.99 | 0.44 | 65.76 | 114.22 | Decrease |
| Togo | 230.82 | 0.41 | 66.77 | 164.05 | Decrease |
| Tokelau | 114.38 | 0.69 | 13.02 | 101.36 | Decrease |
| Tonga | 179.58 | 0.63 | 17.65 | 161.93 | Decrease |
| Trinidad and Tobago | 56.14 | 0.77 | 12.30 | 43.84 | Decrease |
| Tunisia | 123.32 | 0.68 | 13.02 | 110.30 | Decrease |
| Turkey | 53.83 | 0.71 | 12.29 | 41.54 | Decrease |
| Turkmenistan | 545.80 | 0.68 | 13.07 | 532.72 | Increase |
| Tuvalu | 160.23 | 0.58 | 20.90 | 139.34 | Decrease |
| Uganda | 288.87 | 0.42 | 65.53 | 223.34 | Decrease |
| Ukraine | 238.12 | 0.76 | 12.45 | 225.67 | Increase |
| United Arab Emirates | 113.54 | 0.85 | 12.28 | 101.26 | Decrease |
| United Kingdom | 24.56 | 0.86 | 12.11 | 12.45 | Increase |
| United Republic of Tanzania | 288.34 | 0.45 | 65.25 | 223.10 | Decrease |
| United States Virgin Islands | 69.17 | 0.82 | 12.29 | 56.88 | Decrease |
| United States of America | 160.14 | 0.86 | 12.18 | 147.95 | Increase |
| Uruguay | 83.43 | 0.72 | 12.29 | 71.14 | Decrease |
| Uzbekistan | 261.66 | 0.66 | 13.88 | 247.78 | Decrease |
| Vanuatu | 226.60 | 0.47 | 65.05 | 161.55 | Decrease |
| Venezuela (Bolivarian Republic of) | 137.41 | 0.60 | 19.82 | 117.59 | Decrease |
| Viet Nam | 182.20 | 0.63 | 17.48 | 164.73 | Decrease |
| Yemen | 234.44 | 0.45 | 65.63 | 168.80 | Decrease |
| Zambia | 398.84 | 0.51 | 55.62 | 343.22 | Decrease |
| Zimbabwe | 307.35 | 0.47 | 64.94 | 242.41 | Increase |
