# Supplementary material for: Global, regional, and national burden of chronic hepatitis C including cirrhosis from 1990 to 2021, alongside projections for the next 15 years
Source: Medicine (Baltimore). 2026 Oct 16;105(42):e51118. doi: 10.1097/MD.0000000000051118 (PMC13681144; doi:10.1097/MD.0000000000051118)
Supplement: Supplementary file 17 [file medi-105-e51118-s017.docx]

Supplementary Table S17. Prediction of global prevalence of chronic hepatitis C including cirrhosis trend from 2022 to 2036.

| year | Age-standardized prevalence rate | sd | 0.025Q | 0.975Q | Number of prevalence cases | sd | 0.025Q | 0.975Q |
| --- | --- | --- | --- | --- | --- | --- | --- | --- |
| 2022 | 1687.11 | 16.49 | 1654.79 | 1719.43 | 135748478.87 | 1306586.98 | 133187615.45 | 138309342.29 |
| 2023 | 1685.21 | 26.70 | 1632.88 | 1737.55 | 136910782.24 | 2147531.86 | 132701697.15 | 141119867.33 |
| 2024 | 1683.56 | 39.60 | 1605.94 | 1761.18 | 138065634.47 | 3222240.03 | 131750160.06 | 144381108.87 |
| 2025 | 1682.18 | 54.52 | 1575.31 | 1789.04 | 139212812.67 | 4481542.23 | 130429151.30 | 147996474.04 |
| 2026 | 1680.81 | 71.14 | 1541.38 | 1820.24 | 140330609.87 | 5902960.41 | 128761020.07 | 151900199.66 |
| 2027 | 1679.26 | 89.27 | 1504.30 | 1854.22 | 141402485.28 | 7473607.35 | 126754484.04 | 156050486.52 |
| 2028 | 1677.76 | 108.76 | 1464.60 | 1890.92 | 142448576.00 | 9183458.72 | 124449327.66 | 160447824.33 |
| 2029 | 1676.50 | 129.51 | 1422.67 | 1930.33 | 143485164.79 | 11026147.69 | 121874312.42 | 165096017.15 |
| 2030 | 1675.51 | 151.45 | 1378.67 | 1972.35 | 144513240.74 | 12996911.11 | 119039763.05 | 169986718.43 |
| 2031 | 1674.55 | 174.52 | 1332.50 | 2016.59 | 145513009.47 | 15090776.03 | 115935631.95 | 175090387.00 |
| 2032 | 1673.42 | 198.64 | 1284.09 | 2062.74 | 146467786.64 | 17303172.66 | 112554191.42 | 180381381.86 |
| 2033 | 1672.34 | 223.78 | 1233.74 | 2110.94 | 147395954.93 | 19631334.69 | 108919245.98 | 185872663.89 |
| 2034 | 1671.49 | 249.92 | 1181.67 | 2161.32 | 148313942.31 | 22073977.26 | 105049741.88 | 191578142.74 |
| 2035 | 1670.89 | 277.03 | 1127.93 | 2213.85 | 149223544.93 | 24629229.22 | 100951142.69 | 197495947.17 |
| 2036 | 1670.31 | 305.05 | 1072.42 | 2268.21 | 150105557.60 | 27292511.07 | 96613218.86 | 203597896.34 |
