# Supplementary material for: Global, regional, and national burden of chronic hepatitis C including cirrhosis from 1990 to 2021, alongside projections for the next 15 years
Source: Medicine (Baltimore). 2026 Oct 16;105(42):e51118. doi: 10.1097/MD.0000000000051118 (PMC13681144; doi:10.1097/MD.0000000000051118)
Supplement: Supplementary file 18 [file medi-105-e51118-s018.docx]

Supplementary Table S18. Prediction of global deaths of chronic hepatitis C including cirrhosis trend from 2022 to 2036.

| year | Age-standardized mortality rate | sd | 0.025Q | 0.975Q | Number of deaths cases | sd | 0.025Q | 0.975Q |
| --- | --- | --- | --- | --- | --- | --- | --- | --- |
| 2022 | 4.26 | 0.04 | 4.18 | 4.34 | 342907.92 | 3237.29 | 336562.95 | 349252.89 |
| 2023 | 4.21 | 0.06 | 4.09 | 4.33 | 342141.33 | 4979.38 | 332381.92 | 351900.73 |
| 2024 | 4.16 | 0.09 | 3.99 | 4.33 | 341242.22 | 7175.74 | 327178.03 | 355306.41 |
| 2025 | 4.11 | 0.12 | 3.88 | 4.34 | 340200.76 | 9702.32 | 321184.57 | 359216.95 |
| 2026 | 4.06 | 0.15 | 3.77 | 4.36 | 339100.12 | 12510.32 | 314580.34 | 363619.90 |
| 2027 | 4.02 | 0.18 | 3.65 | 4.38 | 337993.77 | 15579.11 | 307459.28 | 368528.27 |
| 2028 | 3.97 | 0.22 | 3.53 | 4.40 | 336853.20 | 18857.71 | 299892.78 | 373813.63 |
| 2029 | 3.92 | 0.26 | 3.41 | 4.43 | 335556.13 | 22308.68 | 291831.92 | 379280.34 |
| 2030 | 3.88 | 0.30 | 3.29 | 4.47 | 334125.14 | 25913.98 | 283334.68 | 384915.61 |
| 2031 | 3.83 | 0.34 | 3.16 | 4.50 | 332656.70 | 29675.06 | 274494.64 | 390818.75 |
| 2032 | 3.79 | 0.38 | 3.03 | 4.54 | 331182.84 | 33596.09 | 265335.71 | 397029.96 |
| 2033 | 3.74 | 0.43 | 2.91 | 4.58 | 329639.29 | 37643.40 | 255859.58 | 403419.00 |
| 2034 | 3.70 | 0.47 | 2.78 | 4.62 | 327927.47 | 41785.34 | 246029.70 | 409825.23 |
| 2035 | 3.66 | 0.52 | 2.64 | 4.67 | 326083.88 | 46011.93 | 235902.16 | 416265.61 |
| 2036 | 3.61 | 0.56 | 2.51 | 4.71 | 324201.65 | 50332.46 | 225551.83 | 422851.47 |
