# Supplementary material for: Global, regional, and national burden of chronic hepatitis C including cirrhosis from 1990 to 2021, alongside projections for the next 15 years
Source: Medicine (Baltimore). 2026 Oct 16;105(42):e51118. doi: 10.1097/MD.0000000000051118 (PMC13681144; doi:10.1097/MD.0000000000051118)
Supplement: Supplementary file 19 [file medi-105-e51118-s019.docx]

Supplementary Table S19. Prediction of global DALYs of chronic hepatitis C including cirrhosis trend from 2022 to 2036.

| year | Age-standardized DALYs rate | sd | 0.025Q | 0.975Q | Number of DALYs cases | sd | 0.025Q | 0.975Q |
| --- | --- | --- | --- | --- | --- | --- | --- | --- |
| 2022 | 138.10 | 1.71 | 134.76 | 141.44 | 11110749.24 | 137226.96 | 10841789.34 | 11379709.14 |
| 2023 | 136.51 | 2.34 | 131.93 | 141.09 | 11088209.70 | 189883.38 | 10716045.11 | 11460374.30 |
| 2024 | 134.93 | 3.16 | 128.73 | 141.12 | 11062291.38 | 259034.57 | 10554592.96 | 11569989.81 |
| 2025 | 133.35 | 4.11 | 125.29 | 141.41 | 11032164.41 | 340338.44 | 10365113.32 | 11699215.50 |
| 2026 | 131.77 | 5.17 | 121.64 | 141.91 | 10997473.16 | 431407.73 | 10151929.54 | 11843016.78 |
| 2027 | 130.22 | 6.31 | 117.86 | 142.58 | 10959899.39 | 530865.15 | 9919422.81 | 12000375.98 |
| 2028 | 128.68 | 7.51 | 113.97 | 143.40 | 10919840.15 | 637205.99 | 9670939.35 | 12168740.95 |
| 2029 | 127.16 | 8.76 | 109.99 | 144.33 | 10876248.48 | 749402.90 | 9407445.77 | 12345051.18 |
| 2030 | 125.63 | 10.06 | 105.92 | 145.34 | 10828399.15 | 866778.82 | 9129543.88 | 12527254.43 |
| 2031 | 124.09 | 11.39 | 101.77 | 146.41 | 10774594.79 | 988770.69 | 8836639.84 | 12712549.74 |
| 2032 | 122.54 | 12.75 | 97.55 | 147.53 | 10716164.36 | 1115091.01 | 8530626.14 | 12901702.57 |
| 2033 | 120.99 | 14.14 | 93.28 | 148.71 | 10654284.56 | 1245049.34 | 8214032.69 | 13094536.43 |
| 2034 | 119.45 | 15.55 | 88.98 | 149.92 | 10588738.12 | 1378043.18 | 7887823.11 | 13289653.12 |
| 2035 | 117.91 | 16.97 | 84.65 | 151.16 | 10518759.33 | 1513560.54 | 7552235.19 | 13485283.47 |
| 2036 | 116.32 | 18.39 | 80.28 | 152.37 | 10441446.86 | 1650848.25 | 7205843.74 | 13677049.97 |
